# Integrated bioinformatics analysis for the identification of idiopathic pulmonary fibrosis–related genes and potential therapeutic drugs

**DOI:** 10.1186/s12890-023-02678-z

**Published:** 2023-10-04

**Authors:** Zhenzhen Zhang, Qingzhou Guan, Yange Tian, Xuejie Shao, Peng Zhao, Lidong Huang, Jiansheng Li

**Affiliations:** 1 Academy of Chinese Medical Sciences, Henan University of Chinese Medicine, Zhengzhou, 450046 China; 2 Henan Key Laboratory of Chinese Medicine for Respiratory Disease, Collaborative Innovation Center for Chinese Medicine and Respiratory Diseases Co-Constructed By Henan Province and Education Ministry of P.R. China, Henan University of Chinese Medicine, Zhengzhou, 450046 China; 3 Department of Respiratory Diseases, The First Affiliated Hospital of Henan University of Chinese Medicine, Zhengzhou, 450000 China

**Keywords:** Idiopathic pulmonary fibrosis, Gene expression, Differentially expressed genes, ceRNA network, Traditional Chinese medicine

## Abstract

**Objective:**

The pathogenesis of idiopathic pulmonary fibrosis (IPF) remains unclear. We sought to identify IPF-related genes that may participate in the pathogenesis and predict potential targeted traditional Chinese medicines (TCMs).

**Methods:**

Using IPF gene-expression data, Wilcoxon rank-sum tests were performed to identify differentially expressed genes (DEGs). Protein–protein interaction (PPI) networks, hub genes, and competitive endogenous RNA (ceRNA) networks were constructed or identified by Cytoscape. Quantitative polymerase chain reaction (qPCR) experiments in TGF-β1-induced human fetal lung (HFL) fibroblast cells and a pulmonary fibrosis mouse model verified gene reliability. The SymMap database predicted potential TCMs targeting IPF. The reliability of TCMs was verified in TGF-β1-induced MRC-5 cells.

**Materials:**

Multiple gene-expression profile data of normal lung and IPF tissues were downloaded from the Gene Expression Omnibus database. HFL fibroblast cells and MRC-5 cells were purchased from Wuhan Procell Life Science and Technology Co., Ltd. (Wuhan, China). C57BL/12 mice were purchased from Beijing Vital River Laboratory Animal Technology Co., Ltd. (Beijing, China).

**Results:**

In datasets GSE134692 and GSE15197, DEGs were identified using Wilcoxon rank-sum tests (both *p* < 0.05). Among them, 1885 DEGs were commonly identified, and 87% (1640 genes) had identical dysregulation directions (binomial test, *p* < 1.00E-16). A PPI network with 1623 nodes and 8159 edges was constructed, and 18 hub genes were identified using the Analyze Network plugin in Cytoscape. Of 18 genes, *CAV1*, *PECAM1*, *BMP4*, *VEGFA*, *FYN*, *SPP1*, and *COL1A1* were further validated in the GeneCards database and independent dataset GSE24206. ceRNA networks of *VEGFA*, *SPP1*, and *COL1A1* were constructed. The genes were verified by qPCR in samples of TGF-β1-induced HFL fibroblast cells and pulmonary fibrosis mice. Finally, Sea Buckthorn and Gnaphalium Affine were predicted as potential TCMs for IPF. The TCMs were verified by qPCR in TGF-β1-induced MRC-5 cells.

**Conclusion:**

This analysis strategy may be useful for elucidating novel mechanisms underlying IPF at the transcriptome level. The identified hub genes may play key roles in IPF pathogenesis and therapy.

**Supplementary Information:**

The online version contains supplementary material available at 10.1186/s12890-023-02678-z.

## Introduction

Idiopathic pulmonary fibrosis (IPF), an unexplained progressive and fatal interstitial lung disease, is the most common form of idiopathic interstitial pneumonia and predominantly affects the health of men and smokers [1]. In recent years, evidence has suggested that the morbidity and mortality rates of IPF patients are increasing [2]. IPF primarily occurs in middle-aged or elderly people with a median age of approximately 65 years at the time of diagnosis, and the median survival time is 3–5 years following diagnosis [3, 4]. In addition, people with IPF usually experience increasingly debilitating symptoms, including cough, dyspnea, fatigue, and weight loss. The physical, psychological, and socio-economic burdens of IPF are substantial [5, 6], yet treatment options for patients with IPF remain limited. Lung transplantation is a fundamental treatment that clearly improves survival in carefully selected patients, but the restricted supply of donor organs and the limitations of chronic allograft rejection mean that only a few patients can receive this intervention [7]. Two drugs, pirfenidone and nintedanib, are also commonly used for IPF and can slow down the progression of pulmonary fibrosis and reduce mortality. However, their effects are not ideal, and they trigger complications like nausea, diarrhea, dyspepsia, and rash [8, 9].

The etiology of IPF remains unclear. Numerous pathophysiological factors have been implied in the genesis and progression of the disease, but most are not yet fully elucidated [10]. The underlying pathomechanisms of IPF, with its complex immunological and inflammatory processes and external impacts, have been the focus of recent research. Smoking, pneumotoxic medications, and the inhalation of dusts are well-known risk factors [11]. Moreover, genetic factors play a vital role in the pathogenesis of IPF [12]. For example, in patients with IPF, *GDF15* messenger RNA (mRNA) expression in lung tissue is significantly increased and correlates with pulmonary function [13]. Son et al. [14] suggested that *ROR2* expression correlates with the development and severity of IPF. *LAMA1* mRNA expression was significantly increased in lung tissue obtained from IPF patients and was identified to be a genetic modifier of transforming growth factor TGF-β1 effector responses that significantly affect the development of pulmonary fibrosis [15]. Therefore, it is of great significance to explore the pathogenesis of IPF at the gene level.

In recent years, high-throughput genetic testing technology has become increasingly advanced, and many transcriptomics features have become more common in disease research [16–18]. In addition, competitive endogenous RNA (ceRNA) regulatory networks have been found to be involved in the transcription and regulation of various disease-related genes in a variety of studies [19, 20]. By combining high-throughput microarray/RNA-sequencing data and bioinformatics analysis algorithms, pathogenic genes can be identified, providing some guidance for pathogenesis research and the clinical treatment of these diseases.

In this study, we analyzed the gene-expression profile data of IPF from the Gene Expression Omnibus (GEO) database and identified differentially expressed genes (DEGs). A protein–protein interaction (PPI) network was constructed based on the STRING database (https://www.string-db.org/), and the Analyze Network plugin for Cytoscape (Institute for Systems Biology, Seattle, WA, USA) was applied to verify hub genes. The above hub genes were then optimized using the GeneCards database. Finally, an independent GEO dataset was employed as a validation group to further validate the hub genes. Furthermore, target microRNAs (miRNAs) of identified hub genes were predicted, a co-expressed network was constructed by Cytoscape, and ceRNA networks were constructed based on the prediction results of long non-coding RNAs (lncRNAs). Traditional Chinese medicine (TCM) plays a significant part in treating IPF, boasting strengths like hypotoxicity and multi-level and multi-target actions [9]. The potential therapeutic effect of TCM for IPF was predicted by SymMap database. Our findings provide evidence for the mechanisms of disease development in IPF at the transcriptome level and explore potential hub genes for the treatment of IPF.

## Materials and methods

### Data acquisition and identification of DEGs

First, the keywords “idiopathic pulmonary fibrosis”, “tissue”, “gene expression”, “biopsy or surgical resection” and “homo sapiens” were searched in the GEO database (https://www.ncbi.nlm.nih.gov/geo/) [21], and the samples from lung transplant patient were excluded in this study. Then, a total of 81 IPF and 62 lung normal samples from 6 datasets were found. Among the above datasets, GSE134692 (including 46 IPF and 26 normal samples) and GSE15197 (including 8 IPF and 13 normal samples) has the largest sample size, as shown in Table 1. Thus, these two datasets were downloaded on January 4, 2021 for the following high-throughput analysis. Processed data detected by microarray (Agilent Technologies, Santa Clara, CA, USA) and an RNA-sequencing platform (Illumina, San Diego, CA, USA) were directly downloaded. The probe identifier was mapped to the Entrez Gene identifier with the corresponding platform file for the array-based data, while, for the sequencing-based data, gene symbols were mapped to the Entrez Gene identifier with the Database for Annotation, Visualization and Integrated Discovery (DAVID) database (accessed on 28 January 2021). Then, for the data from public databases, the Wilcoxon rank-sum test was used to identify DEGs (*p* < 0.05).

**Table 1:** Normal lung and IPF tissue data used in this study

GEO Acc	Platform	Normal	IPF
GSE15197	Agilent GPL6480	13	8
GSE134692^a^	Illumina HiSeq 2000 GPL16791	26	46

^a^Denotes the RNA sequencing data

### PPI network construction and hub gene identification

A PPI network was constructed based on the genes of interest using information from the STRING database (https://www.string-db.org/) (accessed on 30 August 2021) with a combined score of  > 0.4 points. Then, the interaction information was downloaded and Cytoscape (version 3.8.2) was used to visualize the PPI network. Degree is an important analytical parameter in network analysis, which represents the number of genes that directly interact with the node gene in the network. The greater the degree value of a node gene was, the more genes that could directly interact with the node gene, and the degree value is also commonly used to identify hub genes [22–25].

Based on information from the DAVID database (https://david.ncifcrf.gov/) (accessed on 8 October 2021), Gene Ontology (GO) [26, 27] and Kyoto Encyclopedia of Genes and Genomes (KEGG) [28–30] pathway enrichment analyses were used to explore the function of hub genes, and the threshold was set as a false-discovery rate (FDR) of  < 0.05. GeneCards is a comprehensive database of human genes, providing concise genomic, proteomic, transcriptional, genetic, and functional information on all known and predicted human genes. Information mainly includes relationships that point to disease, gene expression and gene function, etc. [31]. Then, the hub genes were further optimized and validated in the GeneCards database (https://www.genecards.org/) (accessed on 18 October 2021) and an independent GEO dataset (GSE24206).

### Prediction of target miRNAs and the construction of ceRNA networks

Five online miRNA databases (namely miRDIP, the Encyclopedia of RNA Interactomes [ENCORI], TargetScan, DIANA-micro T, and miRWalk) (accessed on 19 October 2021) were used to predict the target miRNAs of genes of interest with the default parameters [20, 32]. In this study, if a gene (such as gene A) could be targeted by a certain miRNA in at least four of the five databases, then it was defined as the target miRNA of gene A. Based on the miRNA predict result of the interested genes by the five miRNA databases, the miRNA–mRNA pairs were obtained. Then, those gene pairs were imported in Cytoscape software, and the visualization results of miRNA-mRNA co-expressed network was generated.

ENCORI (https://starbase.sysu.edu.cn/index.php) (accessed on 26 October 2021) was used to predict lncRNAs that interacted with the miRNAs of interest using the following screening criteria: mammalian, human h19 genome, strict stringency (≥ 5) of CLIP-Data, and the presence or absence of degradome data [33]. The ceRNA networks of mRNAs, miRNAs, and lncRNAs were constructed using Cytoscape.

### Herbal medicines

Sea Buckthorn was purchased from Tongrentang (Beijing, China) (No.221109004) and Gnaphalium Affine was purchased from KANGMEI PHARMACEUTICAL CO..LTD. (Guangdong, China) (No.3014258). The proportion of Chinese medicine and water is 100 g: 1200 ml water boil to 100 ml for decocting medicine, and then use EYELA freeze-drying machine FDU-2110 (TOKYO RIKAKIKAI CO..LTD., SER.NO. 12211071) to freeze dry into powder, and finally mix into 25 mg/ml concentration.

### Cell culture

Human fetal lung fibroblasts cells were purchased from Wuhan Procell Life Science and Technology Co., Ltd. (Wuhan, China). HFL fibroblast cells were cultured in Ham's F-12 K medium containing 10% fetal bovine serum and 1% penicillin–streptomycin solution at 37 °C and 5% CO_2_. Then, the cultured cells were divided into a control group and a model group. A pulmonary fibrosis cell model was established through HFL fibroblast cells induced by TGF-β1 (5 ng/mL) to activate fibroblasts, while the control group was not treated. The changes in HFL cells induced by TGF-β1 are similar to the pathological changes in human pulmonary fibrotic disease, suggesting that a TGF-β1-induced fibroblast activation model has been successfully established, which has also been used as a model for interstitial pneumonia in other studies [34, 35]. After 6 h of induction, two groups of cells were harvested for quantitative polymerase chain reaction (qPCR) experiments.

MRC-5 cells were purchased from Wuhan Procell Life Science and Technology Co., Ltd. (Wuhan, China) (No. CL-0161). The cells were cultured in medium containing procell MEM + 5% double antibody + 10% lonsera in a 5% CO_2_ incubator at 37 ℃. MRC-5 cells were activated by TGF-β1 (5 ng/mL) to construct the pulmonary fibrosis cell model [36] and divided into control group, model group, Sea Buckthorn group, and Gnaphalium Affine group. The treatment groups were given Sea Buckthorn and Gnaphalium Affine (25 mg/mL), respectively. After 3 ~ 4 h of drug action, TGF-β1 was added to induce for 6 h except the control group. The cells were collected by QIAzol, and the mRNA expressions of *VEGFA*, *SPP1* and *COL1A1* were detected by qPCR experiments.

### Animal experiment

Six C57BL/12 mice (6–8 weeks, 18–22 g) were purchased from Beijing Vital River Laboratory Animal Technology Co., Ltd. (Beijing, China). This experiment was approved by the Ethics Committee of Laboratory Animal Welfare of the Henan University of Chinese Medicine (DWLL202203014). The mice were randomly divided into a model group and control group; then, the model group mice were anesthetized with 1% pentobarbital sodium (50 mg/kg), and pulmonary fibrosis mice were established by injection with bleomycin (5 mg/kg) through an endotracheal drip [35]. The mice in the control group were dripped with the same volume of sterile saline. On day 28, the mice in each group were killed, and their lung tissues (50 mg) were harvested for qPCR experimentation.

### Quantitative real-time polymerase chain reaction

Total RNA was extracted using QIAzol (Qiagen, Hilden, Germany) according to the manufacturer's instructions. Reverse transcription was performed using HiScript® II Q RT SuperMix (Vazyme, Nanjing, China). The complementary DNA after reverse transcription were amplified using a real-time fluorescent qPCR instrument. *GAPDH* and *MUBB* were used as housekeeping genes in HFL fibroblast cells and mice lung tissue for qPCR experiments, respectively (the primers are shown in Tables 2 and 3). The 2^−ΔΔCT^ method was used to assess the gene-expression level. Finally, statistical analysis was performed by one-way analysis of variance testing in SPSS version 22.0 (IBM Corporation, Armonk, NY, USA).

**Table 2:** Primers for human genes of interest

Gene Symbol		Primer sequence (5’–3’)
*VEGFA*	F	GAGGGCAGAATCATCACGAAG
	R	TGTGCTGTAGGAAGCTCATCTCTC
*COL1A1*	F	GAGGGCCAAGACGAAGACATC
	R	CAGATCACGTCATCGCACAAC
*SPP1*	F	GGAGTTGAATGGTGCATACAAGG
	R	CCACGGCTGTCCCAATCAG
*GAPDH*	F	GGAGCGAGATCCCTCCAAAAT
	R	GGCTGTTGTCATACTTCTCATGG

**Table 3:** Primers for mouse genes of interest

Gene Symbol		Primer sequence (5’–3’)
*Vegfa*	F	GATCTGCTCCCTCCCTCTACA
	R	TTGACCCTTTCCCTTTCCTCG
*Spp1*	F	AGCAAGAAACTCTTCCAAGCAA
	R	GTGAGATTCGTCAGATTCATCCG
*Mubb*	F	TGGCTATTAATTATTCGGTCTGCA
	R	GCAAGTGGCTAGAGTGCAGAGTAA

### Prediction of potential TCMs based on the SymMap database

SymMap (http://www.symmap.org/) (accessed on 29 December 2021) integrates TCM with modern medicine through both internal molecular mechanisms and external symptom mapping, thus providing a massive amount of information on herbs/ingredients, targets, and the clinical symptoms and diseases, they are being used to treat for drug-screening efforts. In order to obtain the more reliable TCMs prediction results, we choose a stricter statistical threshold (FDR < 0.01) [37]. Moreover, when all the genes of interest could be targeted by a single herb, this herb was defined as a target medicine of IPF.

## Results

### Identification of DEGs

For the 46 IPF and 26 normal tissue samples from GSE134692, 7550 DEGs were identified using Wilcoxon rank-sum tests (*p* < 0.05). Similarly, for the eight IPF and 13 normal tissue samples from GSE15197, 3557 DEGs were identified. Among those DEGs, 1885 DEGs were commonly identified, and 87% (1640 genes, including 940 up-regulated and 700 down-regulated genes) had identical dysregulation directions, which could not occur by chance (binomial test, *p* < 1.00E-16).

### PPI network construction and hub gene identification

Based on the 1640 DEGs, a PPI network composed of 1623 nodes and 8159 edges was constructed (interaction score > 0.4 points). Then, the network was visualized by Cytoscape, and the Analyze Network plugin in Cytoscape was used to identify hub genes. In this study, most genes have higher degree values, as shown in Table 4. Thus, a relatively strict degree threshold (≥ 100) was set to identify hub genes. Finally, 18 genes (including *IGF1, SPP1, BMP4, CDH2, PECAM1, ITGA2B, CDH5, KDR, SOX2, TJP1, FYN, BDNF, EGF, VEGFA, DLG4, KRAS, CAV1*, and *COL1A1*) were identified, as shown both in Table 4 and in Table S1 in the Supplementary Material.

**Table 4:** The degree value of gene in the network analysis

Gene Symbol	Degree
*VEGFA*	250
*EGF*	214
*KRAS*	198
*PECAM1*	154
*KDR*	152
*CAV1*	146
*FYN*	142
*BDNF*	142
*DLG4*	140
*COL1A1*	136
*IGF1*	128
*CDH2*	124
*SOX2*	122
*BMP4*	116
*CDH5*	116
*ITGA2B*	104
*TJP1*	102
*SPP1*	102
*BMP2*	96
*COL1A2*	96
*RUNX2*	94
*WNT3A*	94
*CRK*	94
*CSF2*	90
*COL3A1*	90
*NFKBIA*	88
*CAT*	88
*PRKCA*	88
*ARRB1*	86
*HSPG2*	86

This table only lists the degree of the top 30 genes due to textual limitation

### GO term and KEGG pathway enrichment analyses

**Fig. 1:**
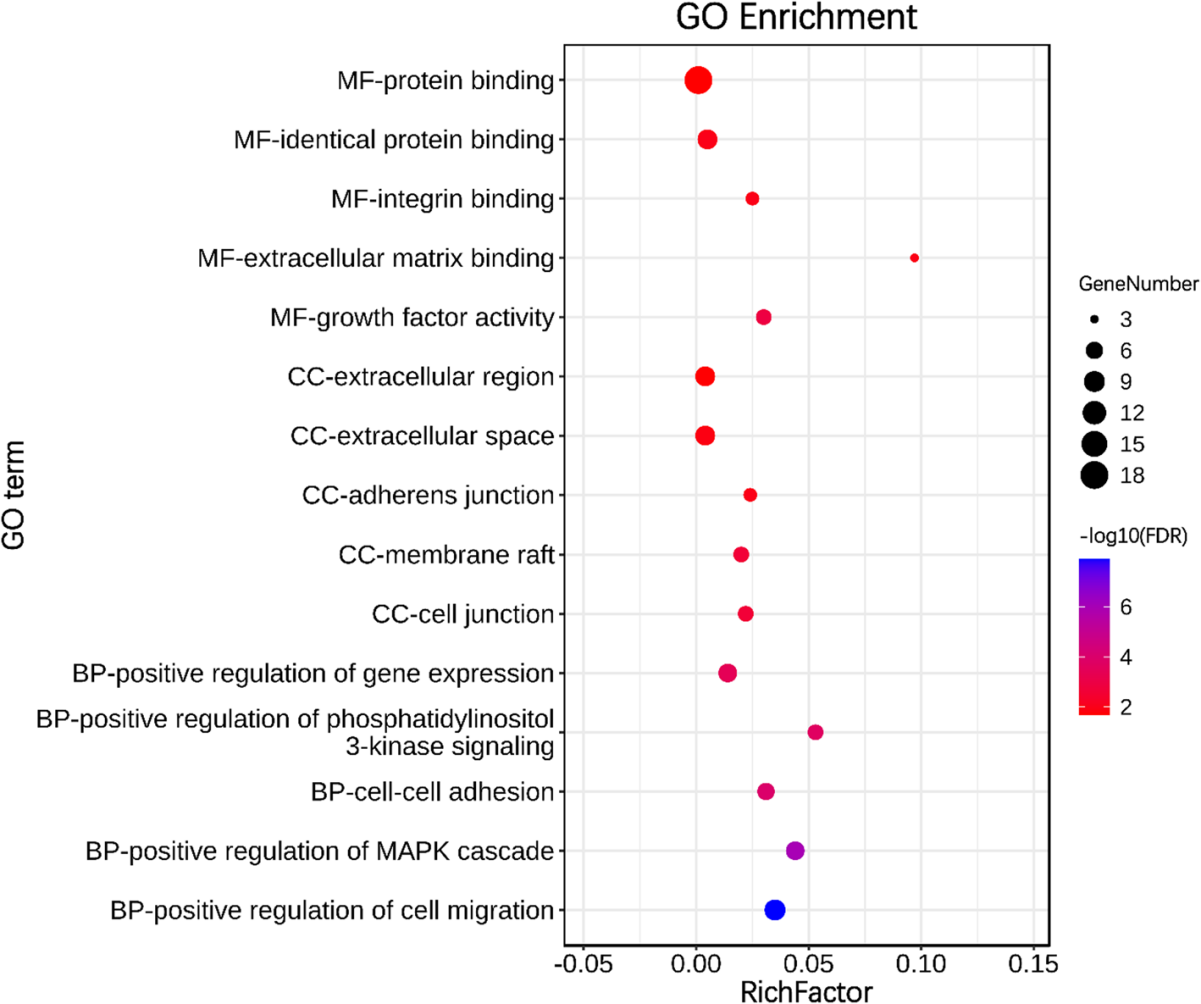
GO enrichment analyses of 18 hub genes. The top 5 terms in each GO category (MF, CC, and BP). All significant GO terms were performed using the DAVID online tool with the cutoff criterion of FDR < 0.05. The color of each bubble represents the FDR for that term, with blue indicating greater significance. The rich factor refers to the proportion of enriched genes for each term

**Fig. 2:**
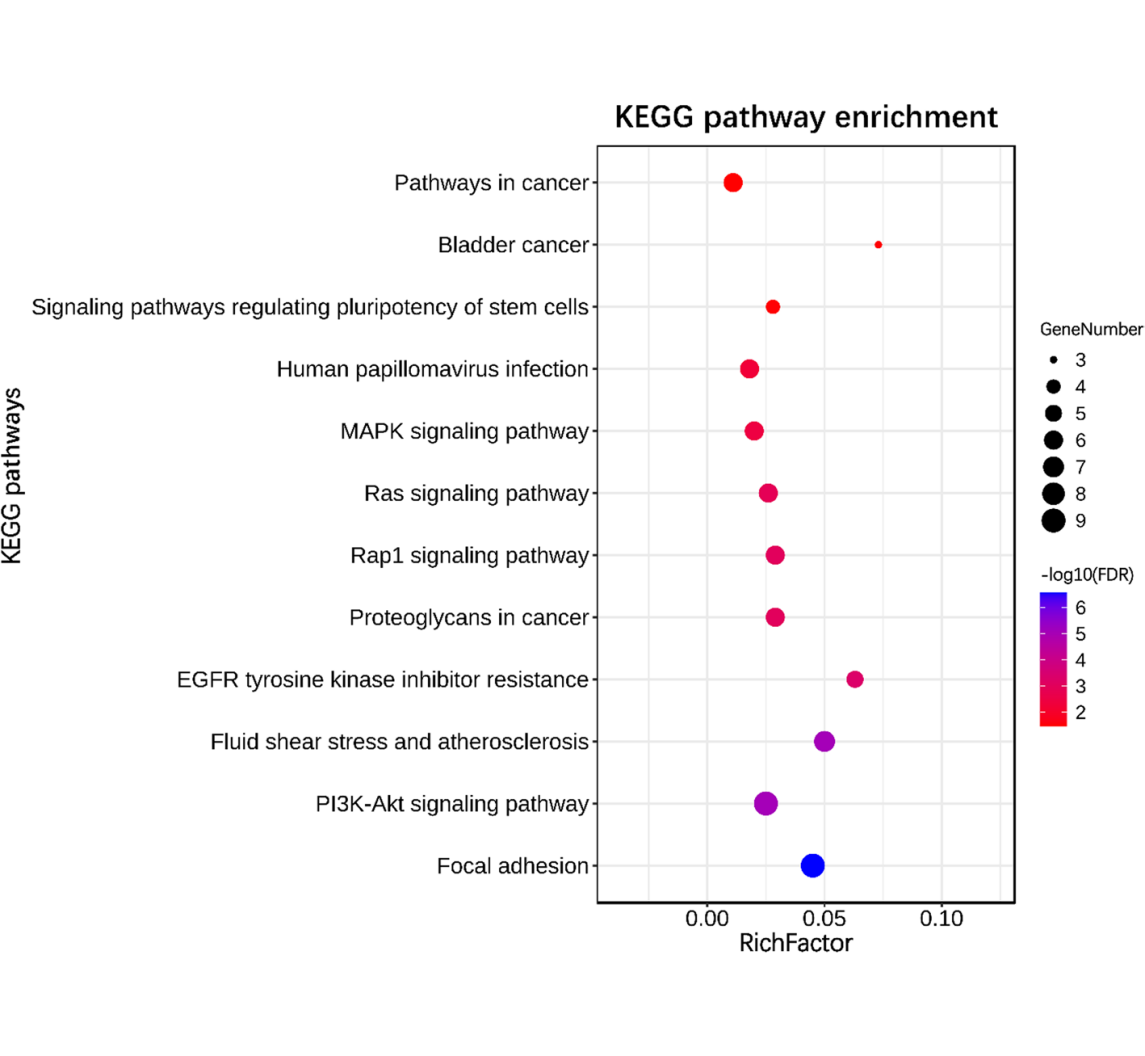
KEGG enrichment analyses of 18 hub genes. All significant KEGG pathways were performed using the DAVID online tool with the cutoff criterion of FDR < 0.05. The color of each bubble represents the FDR for that pathway, with blue indicating greater significance. The rich factor refers to the proportion of enriched genes for each pathway

GO and KEGG analyses of the 18 hub genes were performed using the DAVID database. The GO functional analysis was divided into the following three parts: biological processes (BPs), molecular functions (MFs), and cell components (CCs). The results were considered statistically significant if FDR < 0.05. The top five most significant GO terms and KEGG pathways are shown in Fig. 1 and Fig. 2. And the complete list of enriched GO terms and KEGG pathways were shown in supplementary Table S2 and Table S3.

The GO analysis results revealed that some of these pathways were reported to be related with IPF. For example, in BPs, positive regulation of cell migration, positive regulation of MAPK cascade and cell–cell adhesion are related to the pathogenesis of IPF. Pulmonary fibrosis is typically characterized by activated fibroblast proliferation and migration, and its abnormality is a major concern for treating pulmonary fibrosis [38]. Studies have shown that TGF-β1 plays a central role in the occurrence and development of IPF, and it may promote the fibrotic process of IPF through MAPK signaling pathway [39]. A genome-wide association study of susceptibility to IPF showed that cell–cell adhesion was associated with increased sensitivity to IPF [40]. IPF is characterized primarily by excessive deposition of extracellular matrix (ECM) proteins by activated lung fibroblasts and myofibroblasts, resulting in reduced gas exchange and impaired pulmonary function [41], therefore extracellular space, extracellular region in CCs and extracellular matrix binding in MFs are related to the pathogenesis of IPF. Some enriched KEGG pathways, such as PI3K-Akt signaling pathway and Ras signaling pathway were reported to be related with IPF. Studies showed that the PI3K/Akt/mTOR axis are implicated in fibrosis, the pan-PI3K/mTOR inhibition are currently under clinical evaluation for IPF [42]. Studies have shown that RAB6, a member of the RAS family, and its knockout could inhibit pulmonary fibrosis, oxidative stress, and AEC2 cell death in PM2.5-damaged mice [43].

### Optimization and verification of the hub genes

By searching the 18 candidate hub genes in GeneCards database, we found that *CAV1* and *PECAM1* were specifically expressed in lung tissues, *BMP4* and *VEGFA* were related to angiogenesis, *FYN* and *SPP1* were related to immunity or inflammation, and *COL1A1* was closely associated with pulmonary fibrosis. Thus, *CAV1, PECAM1, BMP4, VEGFA, FYN, SPP1*, and *COL1A1* genes were selected for further investigation.

To further test the reliability of the above seven genes, they were validated in an independent GEO dataset (GSE24206) in which eight IPF and six normal tissue samples were included. The expression levels of *BMP4*, *COL1A1,* and *SPP1* were increased and those of *PECAM1, FYN, VEGFA*, and *CAV1* were decreased in IPF tissues compared to normal tissues in the GEO dataset GSE24206, and all of these genes exhibited dysregulation directions identical to those in the training dataset. These results proved that the aforementioned seven genes were reliable and deserving of further study.

### Target miRNA prediction and ceRNA network construction

**Fig. 3:**
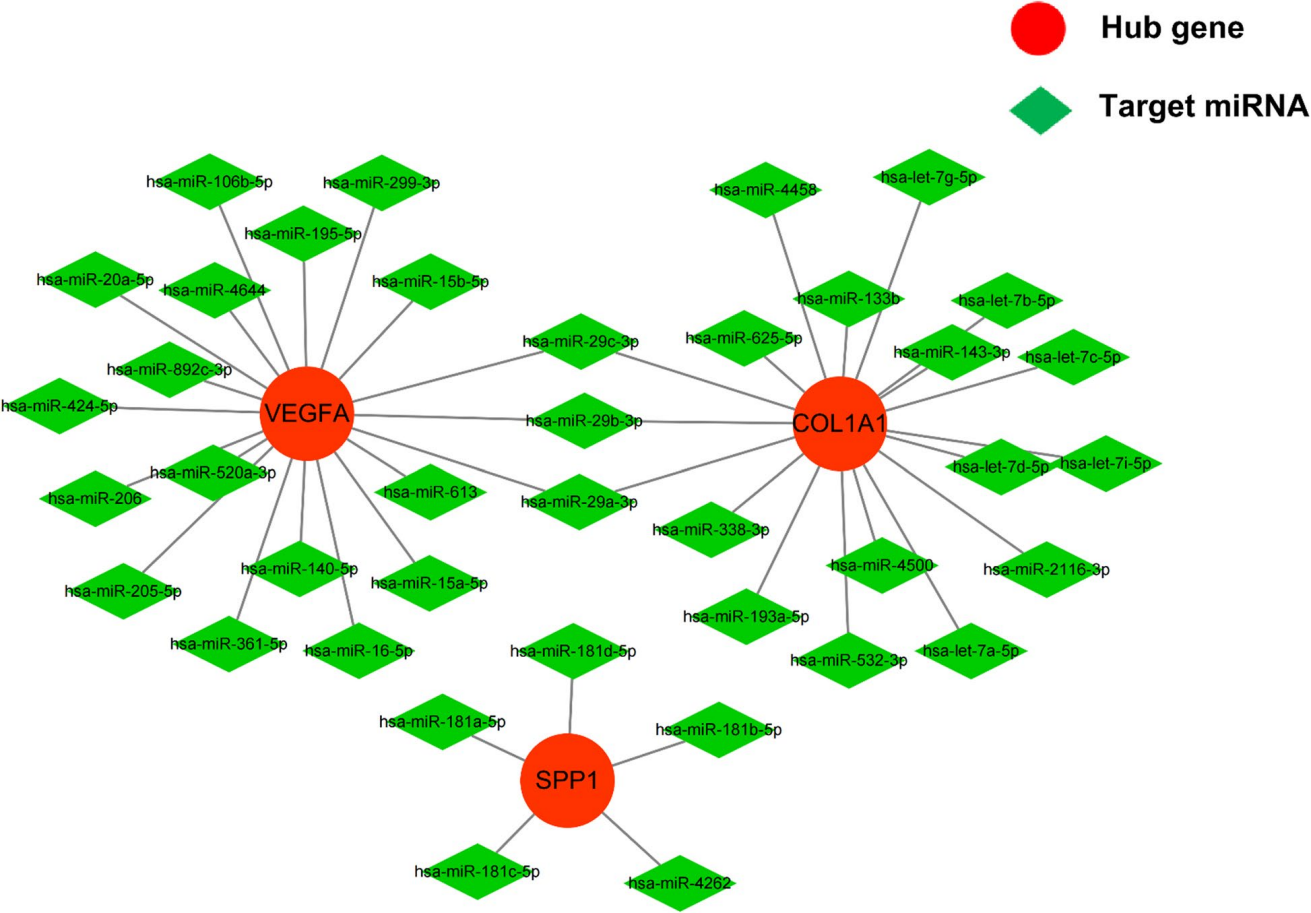
The co-expressed network of mRNAs and target miRNAs. The miRNA–mRNA co-expressed network was constructed by Cytoscape, and included 42 nodes, 42 edges. Red circles represent the hub genes, while green diamonds represent miRNAs

Five online miRNA databases were used to predict the target miRNAs of the seven genes of interest. For each gene, the target miRNA of the five miRNA databases was obtained, as shown in Tables S4–S10 in the Supplementary Material. When a particular miRNA could target a gene of interest in at least four databases, the miRNA was defined as the target of this gene. Finally, 39 target miRNAs of three genes (*VEGFA, COL1A1*, and *SPP1*) and 42 miRNA–mRNA pairs were obtained. An interaction network of mRNAs and miRNAs, including 42 nodes and 42 edges, was constructed by Cytoscape, as shown in Fig. 3.

**Fig. 4:**
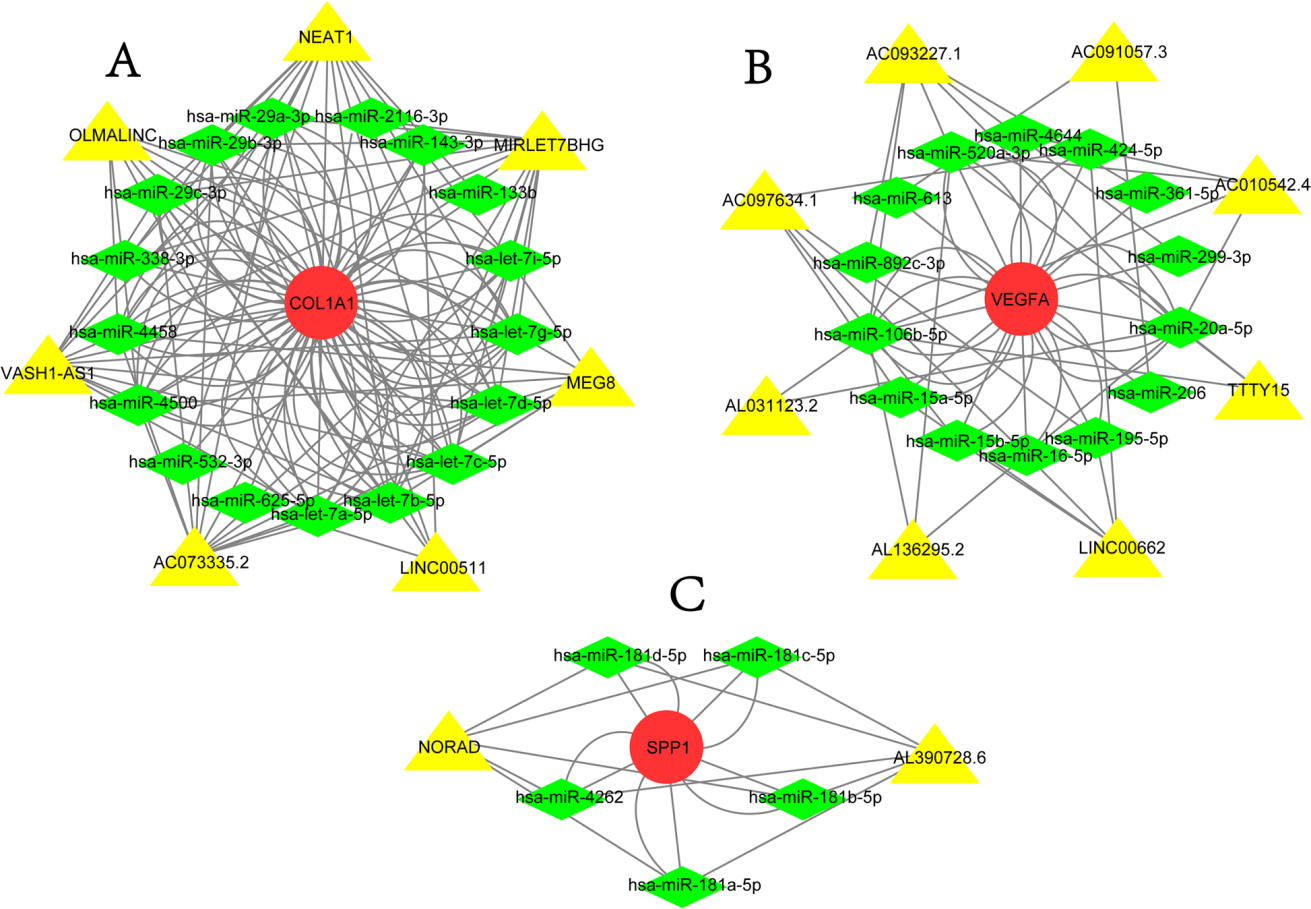
The ceRNA networks of (**A**) *COL1A1* (25 nodes and 142 edges), (**B**) *VEGFA* (23 nodes and 60 edges), and (**C**) *SPP1* (8 nodes and 20 edges). Red circles represent the hub genes, green diamonds represent miRNAs, and yellow triangles represent lncRNAs

It is well known that miRNAs can bind mRNAs and induce gene silencing, further reducing the expression level of a gene, whereas lncRNAs can combine miRNA response elements and increase the expression level of the corresponding gene. This interaction between RNAs is known as a ceRNA network [19]. We also used the ENCORI database to predict the lncRNAs that interact with the selected miRNAs, and the results of ceRNA network are shown in Fig. 4.

### *COL1A1*,* SPP1*, and *VEGFA* were verified in qPCR

**Fig. 5:**
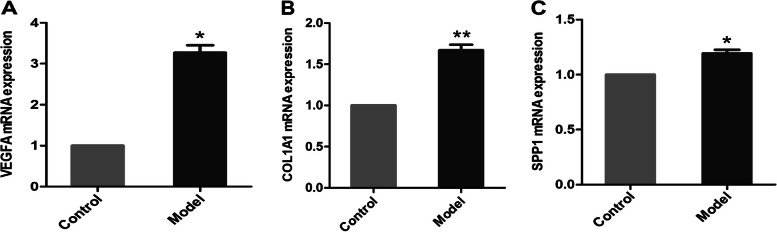
The mRNA expression levels of (**A**) *VEGFA*, (**B**) *COL1A1*, and (**C**) *SPP1* in the HFL fibroblast cells model group and control group (*n* = 3 for each group). The mean ± standard deviation format is used for the expression of values. Compared to the control group, * denotes *p* < 0.05 and ** denotes *p* < 0.01

As shown in Fig. 5, for TGF-β1-induced HFL fibroblast cells, compared to the control group, the expression levels of *COL1A1* and *SPP1* in the model group were significantly up-regulated (*p* < 0.01 and* p* < 0.05, respectively), displaying an identical trend to the results of our data analysis. Compared to the control group, the expression level of *VEGFA* in the model group was also up-regulated (*p* < 0.05); however, the direction of this gene was opposite that found during data analysis in this study, which might be ascribed to the dual role of *VEGFA* in IPF [44].

**Fig. 6:**
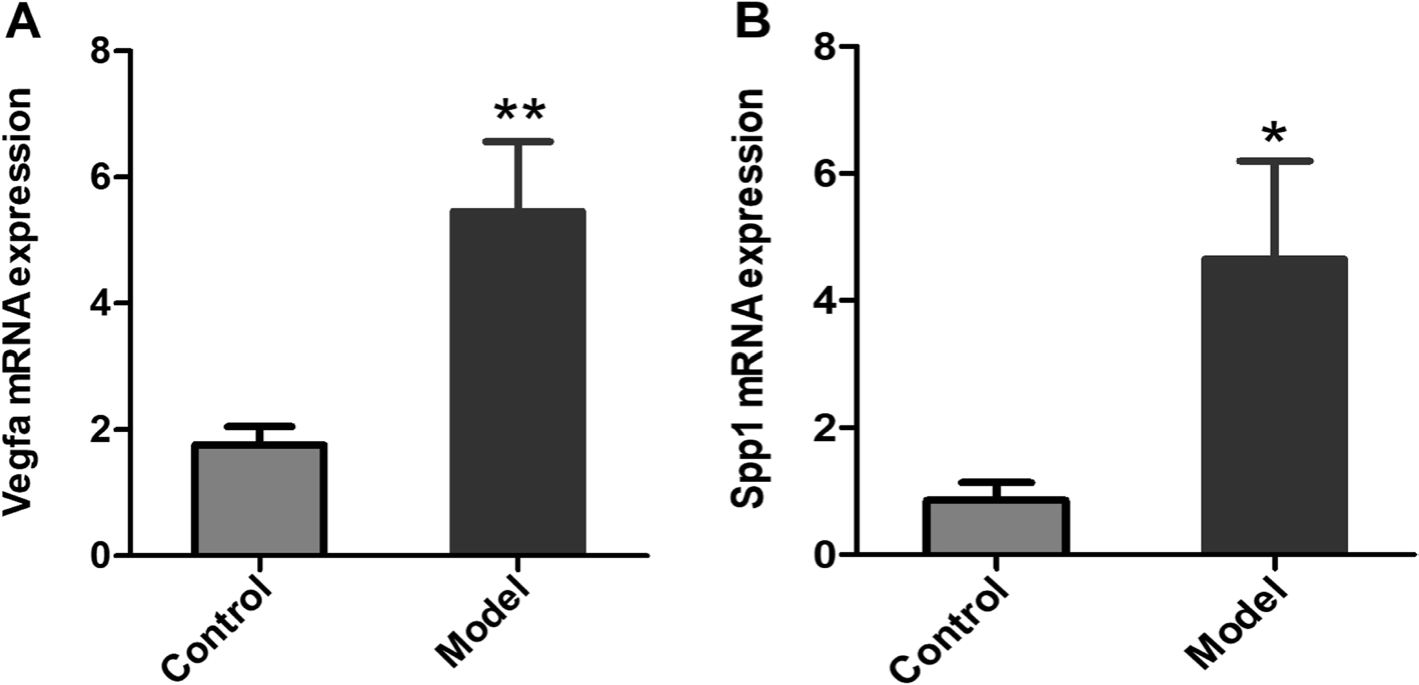
The mRNA expression levels of (**A**) *Vegfa* and (**B**) *Spp1* in the mouse model group and control group (*n* = 3 for each group). Compared to the control group, * denotes *p* < 0.05 and ** denotes *p* < 0.01

For pulmonary fibrosis mice, compared to the control group, the expression of *Spp1* in the model group was significantly increased (*p* < 0.05), consistent with the results of data analysis and HFL fibroblast cell qPCR experimentation. Compared to the control group, the expression of *Vegfa* in the model group was also significantly increased (*p* < 0.01), which was also consistent with the results of HFL fibroblast cell qPCR experimentation. And the results are shown in Fig. 6.

### Prediction of potential therapeutic TCM results

**Fig. 7:**
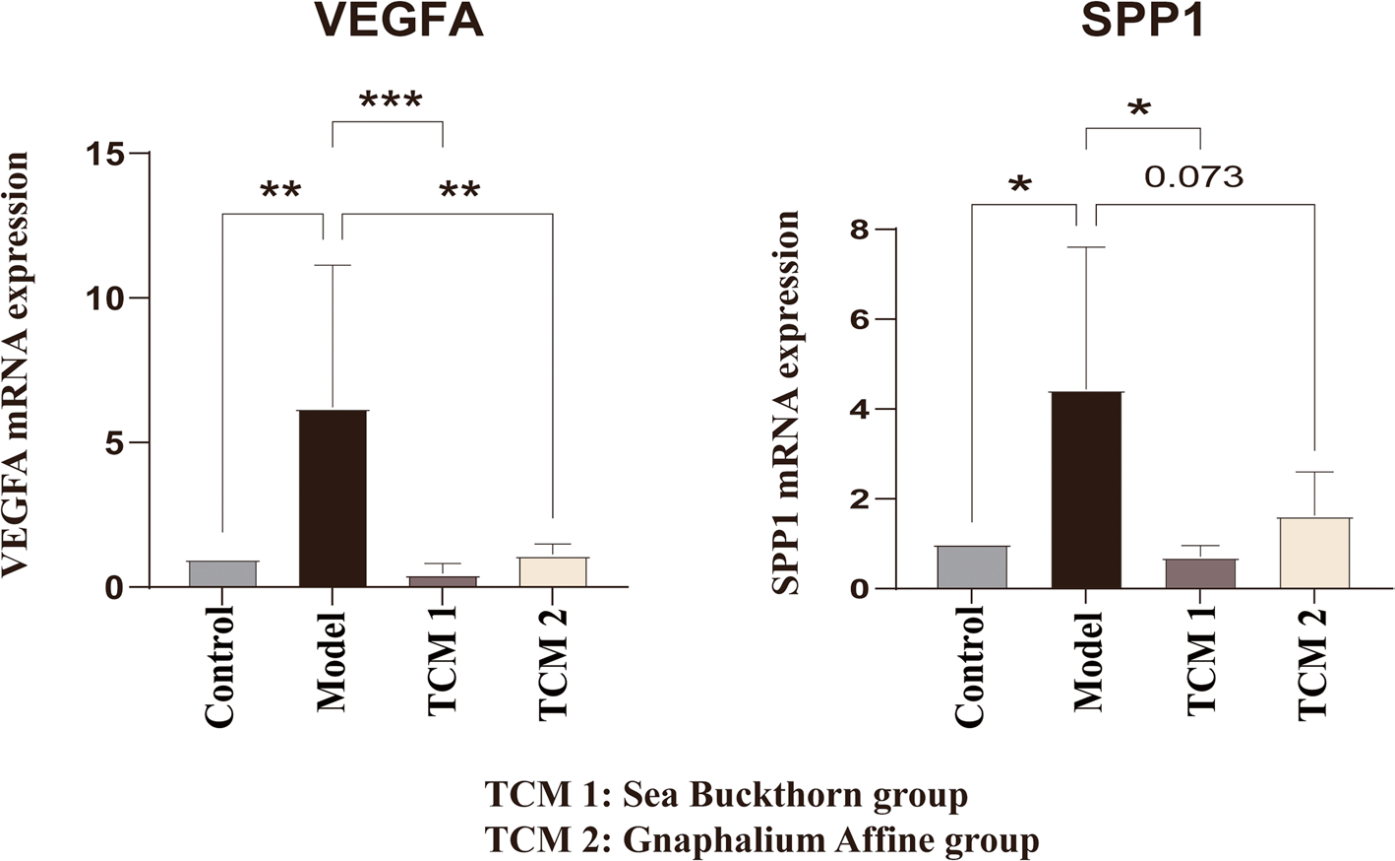
The mRNA expression levels of *VEGFA*, *SPP1* in the MRC-5 cells control group, model group, TCM1 group (Sea Buckthorn group) and TCM2 group (Gnaphalium Affine group) (*n* = 3 for each group). The mean ± standard deviation format is used for the expression of values. * denotes *p* < 0.05, ** denotes *p* < 0.001, *** denotes *p* < 0.0001

TCMs have a unique advantage in the present situation of treating IPF. Potential therapeutic TCMs that target hub genes were predicted using the SymMap database [9]. Three key genes were mapped to screen potential TCMs for IPF under the condition of FDR < 0.01, and some TCMs were identified, as shown in Tables S11–S13 in the Supplementary Material. In this study, if all three genes could be targeted by a single herb, then this herb was defined as a target medicine of IPF. Then, Sea Buckthorn and Gnaphalium Affine were predicted to be potential therapeutic TCMs for IPF, and the intervention mechanism of these herbs on the hub genes were verified in TGF-β1-induced MRC-5 cells. The qPCR result showed that the expression level of *VEGFA* and *SPP1* was significantly up-regulated in the model group (*p* < 0.001 and* p* < 0.05, respectively), which was identical with the result of TGF-β1-induced HFL cell and animal experiment. After the intervention of Sea Buckthorn, the direction of *VEGFA* and *SPP1* were significantly reversed (*p* < 0.05). Similarly, compared with the model group, after the intervention of Gnaphalium Affine, the direction of *VEGFA* was significantly reduced (*p* < 0.001), while the direction of *SPP1* had a decline trend with weak statistical significance (*p* = 0.073), as shown in Fig. 7. The above results indicated that Sea Buckthorn and Gnaphalium Affine have a certain curative effect in the treatment of IPF.

## Discussion

Based on multiple gene-expression data from human lung tissues, we successfully identified DEGs by comparing IPF tissues with normal lung tissues. Then, following PPI network and Cytoscape software analyses, 18 hub genes were identified. Among those genes, we found that *CAV1* and *PECAM1* were specifically expressed in lung tissues, *BMP4* and *VEGFA* were related to angiogenesis, *FYN* and *SPP1* were related to immunity or inflammation, and *COL1A1* was closely associated with pulmonary fibrosis according to the GeneCards database. All of these seven genes were well-verified in the independent GEO dataset GSE24206 (Wilcoxon rank-sum test, *p* < 0.05).

miRNAs are single-stranded, endogenous and non-coding RNAs that function as negative regulators of gene expression at the post-transcriptional level, which could fully or partially bind with complementary sequences in the 3'UTR of the target mRNA, thereby degrading the mRNA or inhibiting its translation [45]. Previous study showed that there was about 10% of miRNAs differ between IPF and control lungs for miRNA microarrays data, miRNAs and the identified potential target genes may contribute to understanding the complex transcriptional programs of pulmonary fibrosis [46]. The network of miRNAs-mRNAs is involved in many important pathways, and there is an urgent need to elucidate the exact mechanisms, which may provide a new approach to identify gene functions and mechanisms involved in IPF pathogenesis [47]. As an endogenous competitive RNA, lncRNA plays an important role in lung disease, tumor, etc. Studies have found that lncRNA could regulate the binding of miRNA and its target mRNA, and is an emerging key regulator of different cellular processes [48, 49]. Therefore, the ceRNA network of lncRNA-miRNA-mRNA was constructed to explore the pathogenesis of IPF. Among the aforementioned seven genes, the ceRNA networks of *SPP1, COL1A1*, and *VEGFA* were constructed, and the three genes were identified as IPF hub genes.

The identified hub genes were verified by qPCR in samples of TGF-β1-induced HFL fibroblast cells and pulmonary fibrosis mice. Finally, Sea Buckthorn and Gnaphalium Affine were predicted to be potential therapeutic TCMs that target IPF hub genes by the SymMap database, and the intervention mechanism of these herbs were verified by qPCR in TGF-β1-induced MRC-5 cells. We found that these herbs could reverse the expression level of hub gene *VEGFA* and *SPP1* in TGF-β1-induced MRC-5 cells. These results showed that the hub genes and predicted herbs have higher reliability and might play vital role in IPF. Further investigation of these genes and TCMs might provide ideas for the research of IPF mechanism and the intervention of TCMs in IPF patient treatment, and perhaps also serve for clinical scenario in the future.

Studies have shown that *SPP1, COL1A1*, and *VEGFA* are closely related to IPF. *SPP1* is a multifunctional protein that plays a vital part in cell–ECM interactions and the pathogenesis of IPF; its silencing is very useful in the treatment of IPF [50]. Moreover, *SPP1* has been shown to have both pro-inflammatory and pro-fibrotic properties, and the *SPP1* gene-expression level was found to increase in mouse models of pulmonary fibrosis and patients with IPF [51]. Lung fibrosis is characterized by the deposition of ECM proteins, including *COL1A1*, combined with collagen I as the most abundant ECM component in both normal and fibrotic conditions. Collagen I has a triple helix structure that arises from two α-1 and one α-2 chains, which are the products of the *COL1A1* and *COL1A2* genes, respectively; down-regulation of *COL1A1* alleviates the accumulation of ECM and then inhibits pulmonary fibrosis [52–54]. *VEGFA* is a growth factor that is active in angiogenesis and endothelial cell growth [55, 56]. Dysregulation of *VEGFA* bioavailability is associated with the progression of lung injury/fibrosis [57]. Several clinical studies have documented a reparative effect of *VEGFA* in the lungs, and it has also been shown that *VEGFA* has a protective effect against the development of excessive pulmonary fibrosis caused by lung injury [44, 58]. Thus, *SPP1*, *COL1A1*, and *VEGFA* play crucial roles in the occurrence and development of IPF and deserve further study.

Compared to control samples, we found that the expression levels of *COL1A1, SPP1,* and *VEGFA* were significantly up-regulated in TGF-β1-induced HFL fibroblast cells. Similarly, the expression levels of *Vegfa* and *Spp1* were significantly increased in the pulmonary fibrosis mouse model. The results of *SPP1* were identical in our data analysis, cell experiments, and mouse experiments. In the cell and mouse experiments, consistent results for *VEGFA* were obtained, and the dysregulated direction of this gene was significantly opposite that in the data analysis, which might due to the fact that *VEGFA* has a dual role in the occurrence and development of IPF [44]. *COL1A1* showed the same results in our data analysis and cell experiments, and there was no statistically significant finding in the mouse experiments.

Studies have demonstrated that Seabuckthorn Wuwei Pulvis (SWP) could improve lung inflammation and chronic bronchitis in mice, and it could also alleviate chronic obstructive pulmonary disease in rats, improve lung function and inhibit inflammatory response through intestinal microbiota short-chain fatty acid axis [59]. A clinical study suggested that Sea Buckthorn could reduce the levels of collagen types III and IV in patients with liver cirrhosis and is an effective drug for the prevention and treatment of liver fibrosis [60]. Other studies have shown that it may suppress the synthesis of collagen and other ECM components [61, 62]. Gnaphalium Affine is an important TCM, and it was used to treat asthma, hyperuricemia, rheumatic arthritis, antitussive, expectorant and cardiovascular in folk medicine due its anti-inflammatory and anti-oxidant activity [63, 64], which might also work for IPF. Some species of Gnaphalium Affine are used as folk medicines in Mexico to treat various respiratory diseases, such as grippe, fever, asthma, cough, cold, bronchitis, expectoration, and bronchial affections [65]. GnafC is derived from Gnaphalium Affine. When adding ascorbate to the culture, gnafC enhanced the expression of the *ALPase* and *MMP13* genes from the early stage of differentiation, leading to maturation of the collagenous ECM [66]. IPF is a chronic, progressive, and ultimately fatal interstitial lung disease characterized by intensive ECM accumulation; therefore, Sea Buckthorn and Gnaphalium Affine have a certain therapeutic effect in IPF from the perspective of ECM management [67].

Our study has certain limitations. Relatively lax thresholds (*p* < 0.05) were used in DEG analysis of IPF data from a public database because DEGs are not recognized in GSE15197 when strict thresholds (FDR < 0.05) are used. However, in the DEGs of the two datasets (GSE15197 and GSE134692) with *p* < 0.05, it was found that 1885 genes were common, and 87% of these genes (1640) had the same dysregulation direction, which would not happen by chance (binomial test, *p* < 1.00E-16). The results indicate that the DEGs identified at the 0.05 threshold were reliable and the cutoff used was reasonable. Currently, there are few IPF samples of high-throughput data available in public databases. In future research, with greater accumulation of IPF data from public databases, an improved algorithm or the analysis of more IPF data should be considered.

## Conclusions

We identified three genes of interest (*SPP1*, *COL1A1*, and *VEGFA*) by bioinformatics analysis that were verified by qPCR in samples of TGF-β1-induced HFL fibroblast cells and pulmonary fibrosis mice. Based on these genes, Sea Buckthorn and Gnaphalium Affine were predicted as potential therapeutic TCMs for IPF, qPCR results showed that these herbs could reverse the expression level of hub gene *VEGFA* and *SPP1* in TGF-β1-induced MRC-5 cells. Further experiments are needed to verify the key genes and related Chinese medicines in the future.

### Supplementary Information

**Additional file 1: Table S1.** The analyze network results of 1640 DEGs. **Table S2.** GO terms of the 18 hub genes. **Table S3.** KEGG pathways of the 18 hub genes. **Table S4.** Target microRNAs of *SPP1* based on five online miRNA databases. **Table S5.** Target microRNAs of *VEGFA* based on five online miRNA databases. **Table S6.** Target microRNAs of *COL1A1* based on five online miRNA databases. **Table S7.** Target microRNAs of *CAV1* based on five online miRNA databases. **Table S8.** Target microRNAs of *PECAM1* based on five online miRNA databases. **Table S9.** Target microRNAs of *BMP4* based on five online miRNA databases. **Table S10.** Target microRNAs of *FYN* based on five online miRNA databases. **Table S11.** Traditional Chinese medicine prediction results of *COL1A1*. **Table S12.** Traditional Chinese medicine prediction results of *VEGFA*. **Table S13.** Traditional Chinese medicine prediction results of *SPP1*.

## Data Availability

The datasets presented in this study can be found in online repositories (https://www.ncbi.nlm.nih.gov/). The names of the repository/repositories and accession number(s) can be found in the article.
