# Supplementary material for: Integrated bioinformatics analysis for the identification of idiopathic pulmonary fibrosis–related genes and potential therapeutic drugs
Source: BMC Pulm Med. 2023 Oct 4;23:373. doi: 10.1186/s12890-023-02678-z (PMC10552267; doi:10.1186/s12890-023-02678-z)
Supplement: Supplementary file 1 — Additional file 1: Table S1. The analyze network results of 1640 DEGs. Table S2. GO terms of the 18 hub genes. Table S3. KEGG pathways of the 18 hub genes. Table S4. Target microRNAs of SPP1 based on five online miRNA databases. Table S5. Target microRNAs of VEGFA based on five online miRNA databases. Table S6. Target microRNAs of COL1A1 based on five online miRNA databases. Table S7. Target microRNAs of CAV1 based on five online miRNA databases. Table S8. Target microRNAs of PECAM1 based on five online miRNA databases. Table S9. Target microRNAs of BMP4 based on five online miRNA databases. Table S10. Target microRNAs of FYN based on five online miRNA databases. Table S11. Traditional Chinese medicine prediction results of COL1A1. Table S12. Traditional Chinese medicine prediction results of VEGFA. Table S13. Traditional Chinese medicine prediction results of SPP1. [file 12890_2023_2678_MOESM1_ESM.zip › Supplementary Tables/Supplementary Table1.docx]

**Table S1 The analyze network results of 1640 DEGs**

| Gene Symbol | Degree |
| --- | --- |
| *VEGFA* | 250 |
| *EGF* | 214 |
| *KRAS* | 198 |
| *PECAM1* | 154 |
| *KDR* | 152 |
| *CAV1* | 146 |
| *BDNF* | 142 |
| *FYN* | 142 |
| *DLG4* | 140 |
| *COL1A1* | 136 |
| *IGF1* | 128 |
| *CDH2* | 124 |
| *SOX2* | 122 |
| *BMP4* | 116 |
| *CDH5* | 116 |
| *ITGA2B* | 104 |
| *TJP1* | 102 |
| *SPP1* | 102 |
| *COL1A2* | 96 |
| *BMP2* | 96 |
| *WNT3A* | 94 |
| *RUNX2* | 94 |
| *CRK* | 94 |
| *COL3A1* | 90 |
| *CSF2* | 90 |
| *CAT* | 88 |
| *PRKCA* | 88 |
| *NFKBIA* | 88 |
| *HSPG2* | 86 |
| *ARRB1* | 86 |
| *FCGR3A* | 84 |
| *TEK* | 84 |
| *FLT1* | 84 |
| *POSTN* | 82 |
| *CLU* | 82 |
| *MAP2K1* | 82 |
| *CTNND1* | 82 |
| *EFNB2* | 80 |
| *COL18A1* | 80 |
| *SNCA* | 80 |
| *GLI1* | 78 |
| *GRIA1* | 78 |
| *ARL13B* | 78 |
| *CCDC40* | 78 |
| *RAB5A* | 76 |
| *TLN1* | 76 |
| *CUL1* | 76 |
| *DNAH1* | 76 |
| *PSEN1* | 74 |
| *THBS2* | 74 |
| *ACAN* | 72 |
| *LDLR* | 72 |
| *NGFR* | 72 |
| *DNAI2* | 72 |
| *SPAG6* | 72 |
| *CD274* | 72 |
| *OCLN* | 70 |
| *TIMP3* | 70 |
| *IFT172* | 70 |
| *YES1* | 70 |
| *LEF1* | 68 |
| *EFHC1* | 68 |
| *ITGB4* | 68 |
| *RUVBL1* | 66 |
| *MPO* | 66 |
| *TP53BP1* | 66 |
| *DNAI1* | 66 |
| *DNALI1* | 66 |
| *PIK3R2* | 66 |
| *FGA* | 64 |
| *FOXJ1* | 64 |
| *SPAG17* | 64 |
| *ENKUR* | 64 |
| *CHEK2* | 64 |
| *CFAP45* | 64 |
| *PROM1* | 62 |
| *MMP1* | 62 |
| *MYH11* | 62 |
| *DNAH9* | 62 |
| *TTC18* | 62 |
| *SLC2A1* | 60 |
| *MMP14* | 60 |
| *TTN* | 60 |
| *DRC1* | 60 |
| *CUL3* | 60 |
| *CCDC65* | 60 |
| *TAGLN* | 58 |
| *FBN1* | 58 |
| *DNM1* | 58 |
| *PPP2CB* | 58 |
| *COL5A2* | 58 |
| *GATA3* | 58 |
| *COL4A3* | 58 |
| *ADAM17* | 56 |
| *SLC6A1* | 56 |
| *PACRG* | 56 |
| *RSPH1* | 56 |
| *MMP7* | 54 |
| *GPC1* | 54 |
| *SCD* | 54 |
| *NOTCH4* | 54 |
| *DLL1* | 54 |
| *ARMC3* | 54 |
| *SH3GL2* | 54 |
| *MAPK9* | 54 |
| *RSPH4A* | 54 |
| *RSPH9* | 54 |
| *TPM2* | 52 |
| *KRT5* | 52 |
| *TEKT1* | 52 |
| *CHUK* | 52 |
| *ADAMTS5* | 50 |
| *ACTG2* | 50 |
| *MYLK* | 50 |
| *SMAD7* | 50 |
| *NRG1* | 50 |
| *MME* | 50 |
| *WNT4* | 50 |
| *TLR3* | 50 |
| *NME5* | 50 |
| *GAP43* | 50 |
| *SYNJ1* | 50 |
| *IFT46* | 50 |
| *RIBC2* | 50 |
| *GATA2* | 50 |
| *COL4A6* | 50 |
| *MSN* | 50 |
| *EFNB3* | 50 |
| *HMGCR* | 48 |
| *CTSK* | 48 |
| *ITSN1* | 48 |
| *TBK1* | 48 |
| *WDR90* | 48 |
| *EPHB2* | 48 |
| *CETN2* | 48 |
| *CC2D2A* | 48 |
| *CCDC113* | 48 |
| *MUC5B* | 48 |
| *NTN1* | 48 |
| *GAB1* | 48 |
| *DNAH6* | 48 |
| *PSMD11* | 48 |
| *ABCB1* | 46 |
| *CD55* | 46 |
| *COL15A1* | 46 |
| *CLTCL1* | 46 |
| *NEDD4* | 46 |
| *IRF3* | 46 |
| *SPTBN1* | 46 |
| *AQP4* | 46 |
| *ITGA7* | 46 |
| *RAP1A* | 46 |
| *CEP164* | 46 |
| *RPGRIP1L* | 46 |
| *CD47* | 46 |
| *DNAH2* | 46 |
| *PSMD12* | 46 |
| *PRF1* | 46 |
| *CLUAP1* | 44 |
| *COMP* | 44 |
| *GP1BA* | 44 |
| *AGT* | 44 |
| *RAD9A* | 44 |
| *SPTBN4* | 44 |
| *EFHC2* | 44 |
| *MARK3* | 44 |
| *ESR2* | 44 |
| *USP14* | 44 |
| *TEKT2* | 44 |
| *PSMD6* | 44 |
| *PSMA6* | 44 |
| *CAMP* | 42 |
| *PLS3* | 42 |
| *GSN* | 42 |
| *S100A8* | 42 |
| *PLD1* | 42 |
| *IFT27* | 42 |
| *VAMP3* | 42 |
| *GAB2* | 42 |
| *BBS5* | 42 |
| *CFAP43* | 42 |
| *PPP2R5A* | 42 |
| *SCN1A* | 42 |
| *EFNA5* | 42 |
| *WDR16* | 42 |
| *CCDC135* | 42 |
| *TP73* | 42 |
| *HSPB1* | 42 |
| *FLNC* | 42 |
| *CCNO* | 42 |
| *RAD23B* | 42 |
| *COL7A1* | 42 |
| *SIX1* | 40 |
| *SCGB1A1* | 40 |
| *LCN2* | 40 |
| *ZMYND10* | 40 |
| *NR0B2* | 40 |
| *B9D2* | 40 |
| *B9D1* | 40 |
| *NPHP1* | 40 |
| *DPYSL2* | 40 |
| *KAT2A* | 40 |
| *HMGCS1* | 40 |
| *TCF3* | 40 |
| *SEMA6A* | 40 |
| *TAB2* | 40 |
| *DNAAF1* | 40 |
| *DNAH3* | 40 |
| *HLA-E* | 40 |
| *VANGL1* | 40 |
| *LEPREL2* | 40 |
| *ATP12A* | 38 |
| *TOP1* | 38 |
| *EPHX2* | 38 |
| *SLC27A2* | 38 |
| *EHMT2* | 38 |
| *MYL12A* | 38 |
| *BMPR2* | 38 |
| *KL* | 38 |
| *COL17A1* | 38 |
| *COL16A1* | 38 |
| *RAPGEF4* | 38 |
| *TLR8* | 38 |
| *LDB3* | 38 |
| *AQP1* | 38 |
| *VAPA* | 38 |
| *CBY1* | 38 |
| *TCTN1* | 38 |
| *NOS2* | 38 |
| *MAP1B* | 38 |
| *MUC16* | 38 |
| *NRXN3* | 38 |
| *USP25* | 38 |
| *CD36* | 38 |
| *DNAAF3* | 38 |
| *KLRD1* | 38 |
| *DLG2* | 38 |
| *PTPRZ1* | 36 |
| *GFM1* | 36 |
| *CALML4* | 36 |
| *VIP* | 36 |
| *AGPAT1* | 36 |
| *LRP2* | 36 |
| *PPP3R1* | 36 |
| *NUP98* | 36 |
| *ATP6V1A* | 36 |
| *SMO* | 36 |
| *IFT22* | 36 |
| *TMEM216* | 36 |
| *TTC21A* | 36 |
| *CFAP61* | 36 |
| *MUC4* | 36 |
| *SERPINF1* | 36 |
| *CFH* | 36 |
| *SFRP2* | 36 |
| *WNT5B* | 36 |
| *TP63* | 36 |
| *TEKT4* | 36 |
| *PCSK9* | 36 |
| *FIGF* | 36 |
| *CD27* | 36 |
| *NCR1* | 36 |
| *CNN1* | 34 |
| *HSPA4L* | 34 |
| *ACVRL1* | 34 |
| *CFP* | 34 |
| *PF4* | 34 |
| *ADRB2* | 34 |
| *FGF14* | 34 |
| *APOH* | 34 |
| *POLR2C* | 34 |
| *FTO* | 34 |
| *TRPV4* | 34 |
| *WDR63* | 34 |
| *IQCD* | 34 |
| *KDM7A* | 34 |
| *IFT43* | 34 |
| *WNT9A* | 34 |
| *EYA1* | 34 |
| *PPFIA1* | 34 |
| *TRADD* | 34 |
| *HLA-C* | 34 |
| *PGF* | 34 |
| *FERMT2* | 34 |
| *ITGA11* | 34 |
| *PGK1* | 32 |
| *EPAS1* | 32 |
| *TRPC3* | 32 |
| *CALML6* | 32 |
| *PRKCE* | 32 |
| *SPTBN2* | 32 |
| *CEP41* | 32 |
| *NGEF* | 32 |
| *HSPB11* | 32 |
| *ODF2* | 32 |
| *WDR66* | 32 |
| *MORN5* | 32 |
| *MYCBPAP* | 32 |
| *QKI* | 32 |
| *ITPR3* | 32 |
| *MUC20* | 32 |
| *GCNT1* | 32 |
| *DBN1* | 32 |
| *NTF4* | 32 |
| *MNS1* | 32 |
| *CYP51A1* | 32 |
| *CFB* | 32 |
| *GP6* | 32 |
| *CCDC146* | 32 |
| *TEKT3* | 32 |
| *GNLY* | 32 |
| *CDH3* | 32 |
| *FZD7* | 32 |
| *RNF8* | 32 |
| *EPHB3* | 32 |
| *KIF3C* | 32 |
| *COL14A1* | 32 |
| *SULF1* | 32 |
| *UBD* | 32 |
| *SLC6A4* | 30 |
| *B3GNT3* | 30 |
| *THBS4* | 30 |
| *HTRA1* | 30 |
| *LMOD1* | 30 |
| *PRDX4* | 30 |
| *PSENEN* | 30 |
| *BTC* | 30 |
| *ASS1* | 30 |
| *AGER* | 30 |
| *S100A12* | 30 |
| *PPAP2C* | 30 |
| *AQP5* | 30 |
| *AIM2* | 30 |
| *PLA2G2A* | 30 |
| *ST6GAL1* | 30 |
| *TMEM231* | 30 |
| *ARL3* | 30 |
| *CCDC96* | 30 |
| *PPP6C* | 30 |
| *STAM* | 30 |
| *SLC1A1* | 30 |
| *TFF3* | 30 |
| *GFRA1* | 30 |
| *SPRY1* | 30 |
| *DYNLRB2* | 30 |
| *MSMO1* | 30 |
| *CCDC11* | 30 |
| *DNM3* | 30 |
| *CADM1* | 30 |
| *CDH13* | 30 |
| *ETF1* | 30 |
| *COL8A2* | 30 |
| *PRKCQ* | 30 |
| *LTBP1* | 30 |
| *FADS1* | 30 |
| *SPAG16* | 30 |
| *ASPN* | 28 |
| *ACSL4* | 28 |
| *ELOVL6* | 28 |
| *MYL12B* | 28 |
| *ADCY2* | 28 |
| *ARHGEF12* | 28 |
| *VPS26A* | 28 |
| *LPIN2* | 28 |
| *CCNA1* | 28 |
| *DHX15* | 28 |
| *DYNC1LI1* | 28 |
| *TCTN3* | 28 |
| *INSIG1* | 28 |
| *PPA1* | 28 |
| *WDPCP* | 28 |
| *RCOR1* | 28 |
| *GPC5* | 28 |
| *CTHRC1* | 28 |
| *UBFD1* | 28 |
| *CCDC37* | 28 |
| *PTPRJ* | 28 |
| *CCDC108* | 28 |
| *S1PR1* | 28 |
| *NDEL1* | 28 |
| *CCL21* | 28 |
| *IGFBP7* | 28 |
| *HLA-G* | 28 |
| *PTPRB* | 28 |
| *NEURL1B* | 28 |
| *PSMD5* | 28 |
| *MMP11* | 26 |
| *PLA2G12B* | 26 |
| *MYL6B* | 26 |
| *IL6R* | 26 |
| *NRG4* | 26 |
| *IGFBP5* | 26 |
| *HHIP* | 26 |
| *SH3GL3* | 26 |
| *RETN* | 26 |
| *CP* | 26 |
| *GPX2* | 26 |
| *FBN2* | 26 |
| *COG7* | 26 |
| *RRAS2* | 26 |
| *KLC1* | 26 |
| *ATF6* | 26 |
| *GPT2* | 26 |
| *TRIM32* | 26 |
| *CDH19* | 26 |
| *CERS2* | 26 |
| *RYR3* | 26 |
| *GRIA3* | 26 |
| *HSD17B7* | 26 |
| *IDI1* | 26 |
| *CYP24A1* | 26 |
| *CCRL2* | 26 |
| *KIF26B* | 26 |
| *GAS2L2* | 26 |
| *FKBP10* | 26 |
| *PVR* | 26 |
| *EGFL7* | 26 |
| *DDB2* | 26 |
| *COL22A1* | 26 |
| *FZD8* | 26 |
| *TUBB1* | 26 |
| *RAB27A* | 26 |
| *OTUB2* | 26 |
| *MBIP* | 26 |
| *STX3* | 26 |
| *IQSEC2* | 24 |
| *ACAD10* | 24 |
| *MMP10* | 24 |
| *DBI* | 24 |
| *ALOX15* | 24 |
| *NRG2* | 24 |
| *ADAMTS6* | 24 |
| *RAD51B* | 24 |
| *PDE1A* | 24 |
| *NECAP1* | 24 |
| *AGTR2* | 24 |
| *CAV2* | 24 |
| *STOM* | 24 |
| *NR3C2* | 24 |
| *RSAD2* | 24 |
| *DPCD* | 24 |
| *CRY1* | 24 |
| *GPX7* | 24 |
| *ERN2* | 24 |
| *PDIA4* | 24 |
| *C8B* | 24 |
| *FBF1* | 24 |
| *LRRC23* | 24 |
| *UNC13D* | 24 |
| *SPAG9* | 24 |
| *EFCAB1* | 24 |
| *IL17RD* | 24 |
| *LIG1* | 24 |
| *HS6ST1* | 24 |
| *GLIS3* | 24 |
| *PLXNA4* | 24 |
| *CSF3R* | 24 |
| *BEST1* | 24 |
| *RALA* | 24 |
| *PPP4R4* | 24 |
| *BPIFA1* | 24 |
| *EPN3* | 24 |
| *EPB41L3* | 24 |
| *L3MBTL1* | 24 |
| *PIH1D2* | 24 |
| *HIST3H2A* | 24 |
| *PPBP* | 24 |
| *PKP2* | 24 |
| *PKP1* | 24 |
| *RIBC1* | 24 |
| *RNF168* | 24 |
| *DOT1L* | 24 |
| *USP2* | 24 |
| *EFCAB11* | 24 |
| *EFNA4* | 24 |
| *CYP3A5* | 22 |
| *DPYD* | 22 |
| *HTR2A* | 22 |
| *FABP5* | 22 |
| *ADAMTS8* | 22 |
| *AGPAT9* | 22 |
| *F12* | 22 |
| *ZNF274* | 22 |
| *KCNN3* | 22 |
| *GALNT13* | 22 |
| *LPCAT1* | 22 |
| *EDNRB* | 22 |
| *SNAPC4* | 22 |
| *ANKRD45* | 22 |
| *ARCN1* | 22 |
| *PPIC* | 22 |
| *SIL1* | 22 |
| *MAT1A* | 22 |
| *LIN7A* | 22 |
| *GPM6A* | 22 |
| *ATP1A2* | 22 |
| *RGMA* | 22 |
| *C6orf165* | 22 |
| *SASH1* | 22 |
| *PPP2R3B* | 22 |
| *CDKN2B* | 22 |
| *FUT2* | 22 |
| *HS3ST1* | 22 |
| *TNNI2* | 22 |
| *PEX13* | 22 |
| *GGT6* | 22 |
| *PCSK1* | 22 |
| *DCLK1* | 22 |
| *LINGO1* | 22 |
| *EYA3* | 22 |
| *F11* | 22 |
| *LMO7* | 22 |
| *SNTN* | 22 |
| *SPRED2* | 22 |
| *FANK1* | 22 |
| *IQCG* | 22 |
| *KRT6A* | 22 |
| *DDX47* | 22 |
| *TRAF5* | 22 |
| *DLG5* | 22 |
| *EIF2S2* | 22 |
| *ELOVL5* | 22 |
| *IRAK2* | 22 |
| *FDXR* | 22 |
| *GAMT* | 22 |
| *DIO2* | 22 |
| *USP15* | 22 |
| *LPHN2* | 22 |
| *SPRY4* | 22 |
| *SLC6A14* | 22 |
| *PTDSS1* | 20 |
| *CAP2* | 20 |
| *PDE2A* | 20 |
| *ADRB1* | 20 |
| *CHRM2* | 20 |
| *RBKS* | 20 |
| *BCOR* | 20 |
| *TTLL1* | 20 |
| *NPNT* | 20 |
| *GPD1L* | 20 |
| *DGKE* | 20 |
| *LPCAT4* | 20 |
| *AK8* | 20 |
| *ALDH3A1* | 20 |
| *GLUD1* | 20 |
| *EMP2* | 20 |
| *CNOT2* | 20 |
| *PLXNB1* | 20 |
| *BVES* | 20 |
| *RAB36* | 20 |
| *RILP* | 20 |
| *NME9* | 20 |
| *CCDC170* | 20 |
| *CAPSL* | 20 |
| *C9orf116* | 20 |
| *ASF1A* | 20 |
| *ATG3* | 20 |
| *TMEM107* | 20 |
| *C11orf70* | 20 |
| *BAAT* | 20 |
| *CROT* | 20 |
| *SOSTDC1* | 20 |
| *FOXF1* | 20 |
| *LBR* | 20 |
| *TMPRSS6* | 20 |
| *CABLES1* | 20 |
| *GPR126* | 20 |
| *TCTE1* | 20 |
| *NUDT3* | 20 |
| *PLAA* | 20 |
| *KRT17* | 20 |
| *STX11* | 20 |
| *ICAM2* | 20 |
| *CD58* | 20 |
| *GALNT18* | 20 |
| *CD300LF* | 20 |
| *GP5* | 20 |
| *RAPGEF2* | 20 |
| *TARS* | 20 |
| *DKK2* | 20 |
| *STXBP6* | 20 |
| *PRMT8* | 20 |
| *KIF18A* | 20 |
| *KLRF1* | 20 |
| *TLE1* | 20 |
| *FAM216B* | 18 |
| *SLC7A11* | 18 |
| *EPHA10* | 18 |
| *NDRG4* | 18 |
| *CRABP2* | 18 |
| *XYLT2* | 18 |
| *ETFA* | 18 |
| *SPA17* | 18 |
| *SETD3* | 18 |
| *INO80* | 18 |
| *MYO1B* | 18 |
| *GRASP* | 18 |
| *UPB1* | 18 |
| *GIPR* | 18 |
| *ASRGL1* | 18 |
| *MTRF1L* | 18 |
| *SORD* | 18 |
| *SFTPD* | 18 |
| *BSCL2* | 18 |
| *PIGA* | 18 |
| *SLC39A8* | 18 |
| *CLEC7A* | 18 |
| *LRRFIP1* | 18 |
| *GSTA1* | 18 |
| *GSTA2* | 18 |
| *DQX1* | 18 |
| *ALOX5AP* | 18 |
| *PPP1R1B* | 18 |
| *TPPP3* | 18 |
| *DNER* | 18 |
| *KIAA0556* | 18 |
| *LRRC46* | 18 |
| *ING3* | 18 |
| *PC* | 18 |
| *CEBPG* | 18 |
| *CFLAR* | 18 |
| *FUT1* | 18 |
| *IGFBP2* | 18 |
| *SMOC1* | 18 |
| *SALL4* | 18 |
| *KIF9* | 18 |
| *KPNA6* | 18 |
| *C6* | 18 |
| *CEACAM1* | 18 |
| *C9orf117* | 18 |
| *CACNB3* | 18 |
| *CEACAM8* | 18 |
| *SGCG* | 18 |
| *NFE2* | 18 |
| *CCHCR1* | 18 |
| *CDC7* | 18 |
| *CDHR3* | 18 |
| *LTBP3* | 18 |
| *ESAM* | 18 |
| *FLI1* | 18 |
| *LEMD3* | 18 |
| *CUBN* | 18 |
| *LAMC3* | 18 |
| *NPR1* | 18 |
| *USP18* | 18 |
| *MDK* | 18 |
| *SLC3A2* | 18 |
| *ST6GALNAC1* | 18 |
| *KIAA2012* | 18 |
| *LRRC71* | 16 |
| *SLC35A1* | 16 |
| *CFAP46* | 16 |
| *CILP* | 16 |
| *CPT1C* | 16 |
| *BMPER* | 16 |
| *SPON2* | 16 |
| *SCGB3A1* | 16 |
| *ARHGAP6* | 16 |
| *NT5C3A* | 16 |
| *AGBL5* | 16 |
| *RAP1GDS1* | 16 |
| *PROK2* | 16 |
| *STOX1* | 16 |
| *RAP2C* | 16 |
| *AK4* | 16 |
| *STEAP3* | 16 |
| *GPX8* | 16 |
| *F2RL2* | 16 |
| *GLS* | 16 |
| *ANAPC10* | 16 |
| *KATNAL2* | 16 |
| *SRD5A2* | 16 |
| *CA4* | 16 |
| *SLC14A1* | 16 |
| *HOPX* | 16 |
| *SFTPA1* | 16 |
| *MYO10* | 16 |
| *ARHGEF25* | 16 |
| *ARL8B* | 16 |
| *ARMC2* | 16 |
| *ARPP19* | 16 |
| *MAST2* | 16 |
| *ASIC1* | 16 |
| *PIEZO1* | 16 |
| *STOML3* | 16 |
| *SAE1* | 16 |
| *FKBP2* | 16 |
| *CADM3* | 16 |
| *CRAT* | 16 |
| *CNEP1R1* | 16 |
| *NPTX2* | 16 |
| *NRGN* | 16 |
| *OXTR* | 16 |
| *MAGED1* | 16 |
| *PAX9* | 16 |
| *BPHL* | 16 |
| *MYOM1* | 16 |
| *C14orf1* | 16 |
| *KPNA1* | 16 |
| *CABYR* | 16 |
| *CACNA2D3* | 16 |
| *CACNG6* | 16 |
| *SCN3A* | 16 |
| *PVRL3* | 16 |
| *HMG20B* | 16 |
| *ZFYVE9* | 16 |
| *CROCC* | 16 |
| *5-Sep* | 16 |
| *MORN3* | 16 |
| *USP44* | 16 |
| *RRP15* | 16 |
| *LRRK1* | 16 |
| *EMR1* | 16 |
| *PTPRM* | 16 |
| *MYLIP* | 16 |
| *DSC3* | 16 |
| *PPIP5K1* | 16 |
| *CLCA2* | 16 |
| *TRIM29* | 16 |
| *PLAGL1* | 16 |
| *IGFBP4* | 16 |
| *VIPR2* | 16 |
| *EPB41L5* | 16 |
| *FBXO15* | 16 |
| *THOC2* | 16 |
| *IQSEC1* | 16 |
| *GSPT1* | 16 |
| *SCN9A* | 16 |
| *KIF6* | 16 |
| *NDUFA8* | 16 |
| *GALNT6* | 16 |
| *TMC7* | 16 |
| *MFNG* | 14 |
| *B3GALNT1* | 14 |
| *SLC22A4* | 14 |
| *PPOX* | 14 |
| *TNN* | 14 |
| *ACPP* | 14 |
| *PSCA* | 14 |
| *IZUMO1* | 14 |
| *SRRM3* | 14 |
| *GCH1* | 14 |
| *ADSSL1* | 14 |
| *AFAP1* | 14 |
| *DPYS* | 14 |
| *TSPAN7* | 14 |
| *AFG3L2* | 14 |
| *TTL* | 14 |
| *PADI4* | 14 |
| *SGMS2* | 14 |
| *NAT1* | 14 |
| *UBAP1* | 14 |
| *C14orf79* | 14 |
| *IL13RA2* | 14 |
| *UBAC1* | 14 |
| *FLVCR2* | 14 |
| *PLA2G5* | 14 |
| *PRKCH* | 14 |
| *MTRR* | 14 |
| *RBM17* | 14 |
| *GRPEL2* | 14 |
| *API5* | 14 |
| *BCL11A* | 14 |
| *PHYHIP* | 14 |
| *RAB6B* | 14 |
| *RAB34* | 14 |
| *PLEKHM1* | 14 |
| *TBC1D15* | 14 |
| *SSR4* | 14 |
| *FAM81B* | 14 |
| *DTX2* | 14 |
| *TPD52L1* | 14 |
| *CBX6* | 14 |
| *UFC1* | 14 |
| *FBXO24* | 14 |
| *LRGUK* | 14 |
| *RS1* | 14 |
| *PLN* | 14 |
| *HS6ST2* | 14 |
| *B3GNT9* | 14 |
| *BCAS1* | 14 |
| *KANSL2* | 14 |
| *CLIC6* | 14 |
| *BIN2* | 14 |
| *TBX1* | 14 |
| *MEOX1* | 14 |
| *OSR2* | 14 |
| *BOC* | 14 |
| *TNFRSF19* | 14 |
| *C16orf93* | 14 |
| *FUZ* | 14 |
| *CCDC173* | 14 |
| *JPH1* | 14 |
| *TES* | 14 |
| *SGCE* | 14 |
| *CFAP57* | 14 |
| *COLEC10* | 14 |
| *DOC2A* | 14 |
| *NGDN* | 14 |
| *PAQR5* | 14 |
| *TNFSF15* | 14 |
| *CDH26* | 14 |
| *PDZD4* | 14 |
| *KIF19* | 14 |
| *SLC1A4* | 14 |
| *SGMS1* | 14 |
| *CYP2F1* | 14 |
| *STK4* | 14 |
| *FANCG* | 14 |
| *CXCL6* | 14 |
| *TCEB3* | 14 |
| *CYB5R4* | 14 |
| *CYP46A1* | 14 |
| *TRPV6* | 14 |
| *DES* | 14 |
| *TNIK* | 14 |
| *PIGC* | 14 |
| *HPD* | 14 |
| *PDHX* | 14 |
| *SYNPO2* | 14 |
| *SHC4* | 14 |
| *GALNT5* | 14 |
| *TSPAN6* | 14 |
| *VIPR1* | 14 |
| *IFIT5* | 14 |
| *SIAH1* | 14 |
| *SH3RF1* | 14 |
| *TP53BP2* | 14 |
| *A4GALT* | 12 |
| *B3GNTL1* | 12 |
| *SH3BP5* | 12 |
| *RUNDC3B* | 12 |
| *ABCC5* | 12 |
| *FBXO32* | 12 |
| *FABP6* | 12 |
| *MYRIP* | 12 |
| *BACE2* | 12 |
| *TMEM59L* | 12 |
| *PGM2L1* | 12 |
| *AMPD3* | 12 |
| *DMGDH* | 12 |
| *RARRES1* | 12 |
| *TTLL10* | 12 |
| *CRTAC1* | 12 |
| *DAK* | 12 |
| *MRVI1* | 12 |
| *TMPRSS4* | 12 |
| *GIMAP5* | 12 |
| *ALAS1* | 12 |
| *HPGD* | 12 |
| *TBXAS1* | 12 |
| *ANKHD1* | 12 |
| *ANKRD1* | 12 |
| *DNAJA4* | 12 |
| *SERPINA5* | 12 |
| *AQR* | 12 |
| *POLR2D* | 12 |
| *UXS1* | 12 |
| *ARID3B* | 12 |
| *LGR6* | 12 |
| *VWA3A* | 12 |
| *FAM183A* | 12 |
| *WDR73* | 12 |
| *DERL3* | 12 |
| *MAP1A* | 12 |
| *KCNJ15* | 12 |
| *FNIP2* | 12 |
| *CIAPIN1* | 12 |
| *BACH2* | 12 |
| *CTSH* | 12 |
| *BAI2* | 12 |
| *BCAS3* | 12 |
| *PIP5K1B* | 12 |
| *CLIC5* | 12 |
| *VWCE* | 12 |
| *BPIFB1* | 12 |
| *TMEM45A* | 12 |
| *DLC1* | 12 |
| *SFI1* | 12 |
| *CCDC81* | 12 |
| *WSCD2* | 12 |
| *C6orf25* | 12 |
| *CCDC176* | 12 |
| *FCN3* | 12 |
| *SYTL2* | 12 |
| *RBM24* | 12 |
| *SELT* | 12 |
| *CIB1* | 12 |
| *REEP5* | 12 |
| *TTC23* | 12 |
| *NDN* | 12 |
| *ZNF365* | 12 |
| *ESPNL* | 12 |
| *LRRC56* | 12 |
| *RABL2A* | 12 |
| *SYTL1* | 12 |
| *CRACR2A* | 12 |
| *CXCL14* | 12 |
| *LILRA5* | 12 |
| *MCEMP1* | 12 |
| *TRAIP* | 12 |
| *DSEL* | 12 |
| *PLVAP* | 12 |
| *ELOVL2* | 12 |
| *DEGS1* | 12 |
| *PPM1D* | 12 |
| *TDP1* | 12 |
| *CHST7* | 12 |
| *SLC24A4* | 12 |
| *GOLM1* | 12 |
| *CPNE8* | 12 |
| *KRT15* | 12 |
| *PNMAL1* | 12 |
| *NBEA* | 12 |
| *SENP5* | 12 |
| *PARD6B* | 12 |
| *GXYLT1* | 12 |
| *DNAJC12* | 12 |
| *PNMA1* | 12 |
| *METTL10* | 12 |
| *DUSP7* | 12 |
| *TMEM2* | 12 |
| *MID1* | 12 |
| *PCMT1* | 12 |
| *PDSS1* | 12 |
| *ETV1* | 12 |
| *SCEL* | 12 |
| *GRIP2* | 12 |
| *SRGAP3* | 12 |
| *HHAT* | 12 |
| *NOVA2* | 12 |
| *SELENBP1* | 12 |
| *SCG5* | 12 |
| *SNCAIP* | 12 |
| *SLCO2A1* | 10 |
| *LTB4R2* | 10 |
| *ACOXL* | 10 |
| *HYAL1* | 10 |
| *CHST6* | 10 |
| *FKBP11* | 10 |
| *SLC45A3* | 10 |
| *ACSM3* | 10 |
| *SPAG8* | 10 |
| *CLEC14A* | 10 |
| *TSPAN12* | 10 |
| *TMEM132C* | 10 |
| *TENM4* | 10 |
| *FAM179A* | 10 |
| *LCA5L* | 10 |
| *ADRA1A* | 10 |
| *SLC6A8* | 10 |
| *NCKAP5* | 10 |
| *DGKD* | 10 |
| *PPAPDC1B* | 10 |
| *CASP4* | 10 |
| *CASP5* | 10 |
| *TMEM14A* | 10 |
| *PPARGC1B* | 10 |
| *POU2F3* | 10 |
| *AMT* | 10 |
| *PCGF5* | 10 |
| *STAC2* | 10 |
| *TRIM55* | 10 |
| *SLC4A11* | 10 |
| *HPCAL4* | 10 |
| *FAM107A* | 10 |
| *KCNJ16* | 10 |
| *PITPNM1* | 10 |
| *PRPF40B* | 10 |
| *ARHGEF10* | 10 |
| *ARHGEF19* | 10 |
| *RNF128* | 10 |
| *ELMOD1* | 10 |
| *IQCE* | 10 |
| *OTUD1* | 10 |
| *RIF1* | 10 |
| *PSD* | 10 |
| *SGK2* | 10 |
| *SLC25A12* | 10 |
| *ENC1* | 10 |
| *ATP5S* | 10 |
| *CWF19L1* | 10 |
| *RRAGC* | 10 |
| *CCDC85A* | 10 |
| *CCDC180* | 10 |
| *POU2AF1* | 10 |
| *WWC3* | 10 |
| *USP32* | 10 |
| *STRA6* | 10 |
| *GRIN3B* | 10 |
| *CPEB1* | 10 |
| *PRSS12* | 10 |
| *MYRF* | 10 |
| *PPP1R32* | 10 |
| *CCDC184* | 10 |
| *MESP1* | 10 |
| *POMGNT2* | 10 |
| *ODF2L* | 10 |
| *SUSD2* | 10 |
| *C16orf45* | 10 |
| *GSE1* | 10 |
| *TNS4* | 10 |
| *SAMD11* | 10 |
| *C1orf192* | 10 |
| *C7orf57* | 10 |
| *C9orf9* | 10 |
| *CCDC181* | 10 |
| *CCDC17* | 10 |
| *SLC4A3* | 10 |
| *10-Mar* | 10 |
| *TCFL5* | 10 |
| *MYO7B* | 10 |
| *TFPI* | 10 |
| *FAM92B* | 10 |
| *PRUNE2* | 10 |
| *STARD3NL* | 10 |
| *DLEC1* | 10 |
| *SLC38A11* | 10 |
| *CCDC74B* | 10 |
| *CCDC78* | 10 |
| *CCDC8* | 10 |
| *CCDC24* | 10 |
| *CCDC60* | 10 |
| *CKMT1A* | 10 |
| *PIGT* | 10 |
| *PCDH19* | 10 |
| *CEP131* | 10 |
| *SPAG4* | 10 |
| *MORN1* | 10 |
| *SPEF1* | 10 |
| *VSTM2L* | 10 |
| *CNTN6* | 10 |
| *COBLL1* | 10 |
| *CPAMD8* | 10 |
| *EFS* | 10 |
| *FBXL2* | 10 |
| *FRMPD2* | 10 |
| *PCDH9* | 10 |
| *KLHL13* | 10 |
| *MPPE1* | 10 |
| *MAK* | 10 |
| *RRN3* | 10 |
| *MRPS30* | 10 |
| *TNNC1* | 10 |
| *DESI2* | 10 |
| *RAET1E* | 10 |
| *SELM* | 10 |
| *SIAE* | 10 |
| *DYDC2* | 10 |
| *IQCK* | 10 |
| *MDH1B* | 10 |
| *ETNK2* | 10 |
| *SIX4* | 10 |
| *EYA2* | 10 |
| *FAM102A* | 10 |
| *LRRN1* | 10 |
| *PTPRT* | 10 |
| *SPATA18* | 10 |
| *MST4* | 10 |
| *RTN4RL1* | 10 |
| *MAP6* | 10 |
| *SLC26A9* | 10 |
| *SPOCK2* | 10 |
| *PLCH2* | 10 |
| *STRIP2* | 10 |
| *TMEM126B* | 10 |
| *MAP3K12* | 10 |
| *MCOLN3* | 10 |
| *SYT8* | 10 |
| *AATK* | 8 |
| *DENND4A* | 8 |
| *SLC22A1* | 8 |
| *GLT1D1* | 8 |
| *CREB3L4* | 8 |
| *ACRBP* | 8 |
| *DPY19L2* | 8 |
| *PAPLN* | 8 |
| *ANXA3* | 8 |
| *DNAJC22* | 8 |
| *NUDT2* | 8 |
| *AGBL2* | 8 |
| *EVA1A* | 8 |
| *HMGB3* | 8 |
| *LPPR3* | 8 |
| *AGTPBP1* | 8 |
| *RCHY1* | 8 |
| *NEK7* | 8 |
| *AKR7A2* | 8 |
| *BCO2* | 8 |
| *CUL9* | 8 |
| *ANKFN1* | 8 |
| *BZRAP1* | 8 |
| *ANKRD40* | 8 |
| *SAR1A* | 8 |
| *SAA2* | 8 |
| *CMTM5* | 8 |
| *SDE2* | 8 |
| *RHOV* | 8 |
| *DTX3* | 8 |
| *VPS37D* | 8 |
| *TXNDC15* | 8 |
| *NPM2* | 8 |
| *RAB21* | 8 |
| *TMEM190* | 8 |
| *ASB2* | 8 |
| *RIMKLA* | 8 |
| *ASXL3* | 8 |
| *ATP11A* | 8 |
| *C14orf37* | 8 |
| *LHPP* | 8 |
| *RSPH10B* | 8 |
| *ISM1* | 8 |
| *PLLP* | 8 |
| *TRIM58* | 8 |
| *RDH5* | 8 |
| *GABRP* | 8 |
| *CHRDL2* | 8 |
| *DALRD3* | 8 |
| *BTBD9* | 8 |
| *FCGBP* | 8 |
| *KIAA1751* | 8 |
| *MRPL14* | 8 |
| *RAMP1* | 8 |
| *PFN4* | 8 |
| *CASKIN2* | 8 |
| *MFSD8* | 8 |
| *CCDC148* | 8 |
| *FAM83D* | 8 |
| *FAM168A* | 8 |
| *JDP2* | 8 |
| *CEP68* | 8 |
| *RMI2* | 8 |
| *OSBPL11* | 8 |
| *TMEM165* | 8 |
| *REEP6* | 8 |
| *SBDS* | 8 |
| *LNX2* | 8 |
| *CLMP* | 8 |
| *UBXN10* | 8 |
| *TSPAN1* | 8 |
| *RGCC* | 8 |
| *PATL1* | 8 |
| *PCYOX1L* | 8 |
| *HOXD1* | 8 |
| *HEPH* | 8 |
| *HECW2* | 8 |
| *TSPAN8* | 8 |
| *NGFRAP1* | 8 |
| *KCNN2* | 8 |
| *RHOF* | 8 |
| *FBXW9* | 8 |
| *EBF3* | 8 |
| *FBXO16* | 8 |
| *KLHL42* | 8 |
| *WSB1* | 8 |
| *FAM81A* | 8 |
| *PAPSS2* | 8 |
| *RAB9B* | 8 |
| *TMEM26* | 8 |
| *SRCIN1* | 8 |
| *PTCRA* | 8 |
| *TMEM132A* | 8 |
| *DPH3* | 8 |
| *RTKN2* | 8 |
| *DTX3L* | 8 |
| *EAF1* | 8 |
| *EFCAB6* | 8 |
| *PRRT3* | 8 |
| *SH2D3C* | 8 |
| *MEGF8* | 8 |
| *KCNMB2* | 8 |
| *PRDM4* | 8 |
| *MAP3K19* | 8 |
| *PNOC* | 8 |
| *FHDC1* | 8 |
| *MTHFSD* | 8 |
| *SCHIP1* | 8 |
| *GYG2* | 8 |
| *KCNH3* | 8 |
| *NUP188* | 8 |
| *PTPRN2* | 8 |
| *ZC3H12C* | 8 |
| *KHDRBS3* | 8 |
| *LRIG3* | 8 |
| *TMEM98* | 8 |
| *TTC29* | 8 |
| *TSPAN13* | 8 |
| *SLITRK6* | 8 |
| *WDR20* | 8 |
| *PRRG1* | 8 |
| *PPP1R13B* | 8 |
| *CYP3A7* | 6 |
| *ANKRD23* | 6 |
| *CHRAC1* | 6 |
| *NET1* | 6 |
| *TMEM100* | 6 |
| *PLEKHS1* | 6 |
| *HSD11B1L* | 6 |
| *AFF2* | 6 |
| *RXFP1* | 6 |
| *AHNAK2* | 6 |
| *AIG1* | 6 |
| *GIMAP6* | 6 |
| *NLRP12* | 6 |
| *AKR7A3* | 6 |
| *LRTOMT* | 6 |
| *ZFP91* | 6 |
| *CAPS* | 6 |
| *ARHGAP31* | 6 |
| *ODAM* | 6 |
| *PRX* | 6 |
| *GPR116* | 6 |
| *CDCA7L* | 6 |
| *ARL8A* | 6 |
| *PITHD1* | 6 |
| *GPR64* | 6 |
| *LEPREL4* | 6 |
| *TMEM150C* | 6 |
| *KCND1* | 6 |
| *DRAM1* | 6 |
| *ATG4A* | 6 |
| *OSTM1* | 6 |
| *CWH43* | 6 |
| *HAUS2* | 6 |
| *OSTF1* | 6 |
| *MGAT4A* | 6 |
| *FGD5* | 6 |
| *TTC39C* | 6 |
| *SCRG1* | 6 |
| *FAM35A* | 6 |
| *PNRC2* | 6 |
| *MPPED2* | 6 |
| *METRN* | 6 |
| *GATS* | 6 |
| *BIK* | 6 |
| *INHBB* | 6 |
| *TMEM8B* | 6 |
| *KCNRG* | 6 |
| *REEP2* | 6 |
| *ZBBX* | 6 |
| *CMBL* | 6 |
| *C16orf74* | 6 |
| *WDR93* | 6 |
| *NDUFAF4* | 6 |
| *CCDC59* | 6 |
| *C1orf158* | 6 |
| *LAMP5* | 6 |
| *FAM198B* | 6 |
| *PROCA1* | 6 |
| *CALCRL* | 6 |
| *DFNA5* | 6 |
| *PEX11G* | 6 |
| *CBLL1* | 6 |
| *DZIP1L* | 6 |
| *GLB1L* | 6 |
| *SPATA17* | 6 |
| *SHE* | 6 |
| *LRRC17* | 6 |
| *CCDC136* | 6 |
| *CCDC178* | 6 |
| *HAGHL* | 6 |
| *CCDC88C* | 6 |
| *MOAP1* | 6 |
| *TMIGD2* | 6 |
| *HHLA2* | 6 |
| *PCDH12* | 6 |
| *TOX* | 6 |
| *GGA2* | 6 |
| *GOLGA7* | 6 |
| *TMEM79* | 6 |
| *DCST2* | 6 |
| *YBX2* | 6 |
| *PDZD8* | 6 |
| *PEAK1* | 6 |
| *TMEM243* | 6 |
| *FBXO31* | 6 |
| *SH2D4A* | 6 |
| *HSD17B6* | 6 |
| *RPL39L* | 6 |
| *LRP2BP* | 6 |
| *SULT1A2* | 6 |
| *SFTA2* | 6 |
| *KIAA1598* | 6 |
| *DZIP3* | 6 |
| *NECAB1* | 6 |
| *SYCP2L* | 6 |
| *PPP1R36* | 6 |
| *PLEKHA6* | 6 |
| *NELL2* | 6 |
| *FAM189A1* | 6 |
| *FAM83F* | 6 |
| *RASL11B* | 6 |
| *SPIRE2* | 6 |
| *MBD2* | 6 |
| *N4BP1* | 6 |
| *HECA* | 6 |
| *LRRC49* | 6 |
| *SLC15A4* | 6 |
| *LRRC18* | 6 |
| *NPR3* | 6 |
| *GYLTL1B* | 6 |
| *RNF114* | 6 |
| *RTP4* | 6 |
| *INPP5K* | 6 |
| *KCNS2* | 6 |
| *SPRYD7* | 6 |
| *KIAA1467* | 6 |
| *NAT14* | 6 |
| *S100A2* | 6 |
| *TTC19* | 6 |
| *MAP4K2* | 6 |
| *TMEM163* | 6 |
| *PCSK1N* | 6 |
| *MYZAP* | 6 |
| *NXF3* | 6 |
| *TSPAN19* | 6 |
| *PHACTR1* | 6 |
| *PSD3* | 6 |
| *PTMA* | 6 |
| *RNF169* | 6 |
| *SLC35D2* | 6 |
| *SYT15* | 6 |
| *CARD6* | 4 |
| *SPEG* | 4 |
| *ADAM23* | 4 |
| *RALGAPB* | 4 |
| *TMEM41B* | 4 |
| *ALG1L* | 4 |
| *ARAP2* | 4 |
| *TAPT1* | 4 |
| *CHRM3* | 4 |
| *ARHGAP29* | 4 |
| *TTC16* | 4 |
| *ARL6IP6* | 4 |
| *NEK11* | 4 |
| *RGS22* | 4 |
| *TSPYL4* | 4 |
| *2-Mar* | 4 |
| *FLRT2* | 4 |
| *PITPNM2* | 4 |
| *BCL2L13* | 4 |
| *STARD5* | 4 |
| *BTNL9* | 4 |
| *C10orf76* | 4 |
| *KIAA1377* | 4 |
| *GPR135* | 4 |
| *C6orf223* | 4 |
| *C17orf89* | 4 |
| *C17orf97* | 4 |
| *C1orf110* | 4 |
| *C9orf24* | 4 |
| *DENND6B* | 4 |
| *RHEBL1* | 4 |
| *CCBE1* | 4 |
| *SCAI* | 4 |
| *CCDC61* | 4 |
| *CCDC89* | 4 |
| *CCDC97* | 4 |
| *CCL16* | 4 |
| *CD101* | 4 |
| *FCRL5* | 4 |
| *SLC44A4* | 4 |
| *CD300E* | 4 |
| *KIAA1804* | 4 |
| *KLF17* | 4 |
| *CDKN2C* | 4 |
| *IGSF9* | 4 |
| *CHCHD7* | 4 |
| *ST6GALNAC5* | 4 |
| *CHST9* | 4 |
| *ZNF655* | 4 |
| *CLDND1* | 4 |
| *PON3* | 4 |
| *SSFA2* | 4 |
| *MYCT1* | 4 |
| *OSGIN2* | 4 |
| *OSGIN1* | 4 |
| *IQCC* | 4 |
| *CPXM1* | 4 |
| *ZBTB18* | 4 |
| *LMCD1* | 4 |
| *GPRC5A* | 4 |
| *CTXN1* | 4 |
| *KCTD1* | 4 |
| *CYB5D2* | 4 |
| *DENND4C* | 4 |
| *SSTR1* | 4 |
| *PYROXD2* | 4 |
| *DPCR1* | 4 |
| *DPEP1* | 4 |
| *DPF3* | 4 |
| *FAM169A* | 4 |
| *SLC25A16* | 4 |
| *DTD2* | 4 |
| *MEX3B* | 4 |
| *STYXL1* | 4 |
| *SCAMP5* | 4 |
| *PRR29* | 4 |
| *ESM1* | 4 |
| *WWC2* | 4 |
| *SOBP* | 4 |
| *FAM177A1* | 4 |
| *TRAPPC13* | 4 |
| *FAM64A* | 4 |
| *FAM83A* | 4 |
| *FAM98C* | 4 |
| *ZFHX2* | 4 |
| *KIAA1407* | 4 |
| *POLN* | 4 |
| *FGFBP2* | 4 |
| *SPIRE1* | 4 |
| *PTGFRN* | 4 |
| *SYNGR1* | 4 |
| *VTCN1* | 4 |
| *RAB26* | 4 |
| *WFDC2* | 4 |
| *HIST3H2BB* | 4 |
| *TRIM45* | 4 |
| *HPCAL1* | 4 |
| *WDR38* | 4 |
| *TMEM9B* | 4 |
| *LURAP1* | 4 |
| *STYK1* | 4 |
| *MTMR9* | 4 |
| *P4HTM* | 4 |
| *LOC728763* | 4 |
| *LONRF3* | 4 |
| *LYNX1* | 4 |
| *LYPD1* | 4 |
| *PLEKHF1* | 4 |
| *MS4A8* | 4 |
| *NUDT10* | 4 |
| *SULT1C4* | 4 |
| *PRR15* | 4 |
| *TMEM194A* | 4 |
| *SAMD5* | 4 |
| *SERPINI2* | 4 |
| *WDR49* | 4 |
| *SLC35G2* | 4 |
| *SLC39A11* | 4 |
| *ZDHHC1* | 4 |
| *TRIM6* | 4 |
| *USP12* | 4 |
| *ABHD14A* | 2 |
| *SERHL2* | 2 |
| *CRLF1* | 2 |
| *MAGED4B* | 2 |
| *AMIGO2* | 2 |
| *CDC14B* | 2 |
| *ANGPTL7* | 2 |
| *FAM154B* | 2 |
| *ANKRD29* | 2 |
| *APCDD1* | 2 |
| *ARHGAP39* | 2 |
| *ZBTB43* | 2 |
| *PNMA3* | 2 |
| *BEND5* | 2 |
| *BEX1* | 2 |
| *BMF* | 2 |
| *BNC1* | 2 |
| *SLC52A1* | 2 |
| *VWA3B* | 2 |
| *GALNS* | 2 |
| *C11orf16* | 2 |
| *C11orf48* | 2 |
| *C20orf195* | 2 |
| *C11orf74* | 2 |
| *C12orf49* | 2 |
| *SOWAHC* | 2 |
| *MPV17L* | 2 |
| *C16orf59* | 2 |
| *TMEM139* | 2 |
| *FAM118A* | 2 |
| *C18orf25* | 2 |
| *C1orf198* | 2 |
| *C1orf228* | 2 |
| *NRBP2* | 2 |
| *C20orf85* | 2 |
| *C22orf15* | 2 |
| *C22orf23* | 2 |
| *C6orf118* | 2 |
| *C9orf135* | 2 |
| *CA13* | 2 |
| *SNX22* | 2 |
| *EFHB* | 2 |
| *EFCAB12* | 2 |
| *FAIM* | 2 |
| *ZNF595* | 2 |
| *LAMP3* | 2 |
| *CCS* | 2 |
| *CCSER2* | 2 |
| *FHOD3* | 2 |
| *CDHR4* | 2 |
| *LRRC43* | 2 |
| *DNPH1* | 2 |
| *CHI3L2* | 2 |
| *ZNF18* | 2 |
| *LRRC27* | 2 |
| *CNTN3* | 2 |
| *CPNE5* | 2 |
| *MEI1* | 2 |
| *ZBED2* | 2 |
| *VILL* | 2 |
| *PA1* | 2 |
| *DPY19L3* | 2 |
| *FAM104B* | 2 |
| *SLAIN1* | 2 |
| *FAM216A* | 2 |
| *NHS* | 2 |
| *METAP2* | 2 |
| *ERGIC3* | 2 |
| *TRERF1* | 2 |
| *FAM172A* | 2 |
| *FAM89A* | 2 |
| *SCARA3* | 2 |
| *ITLN2* | 2 |
| *FEM1C* | 2 |
| *FRY* | 2 |
| *IL15RA* | 2 |
| *GPIHBP1* | 2 |
| *GPR115* | 2 |
| *HKR1* | 2 |
| *GPR157* | 2 |
| *STBD1* | 2 |
| *H2AFY2* | 2 |
| *MOK* | 2 |
| *PTP4A2* | 2 |
| *TTC23L* | 2 |
| *PCDH7* | 2 |
| *WNK2* | 2 |
| *KIAA1324* | 2 |
| *LRRN4* | 2 |
| *KRBA1* | 2 |
| *NMNAT2* | 2 |
| *MARK1* | 2 |
| *MDP1* | 2 |
| *MID1IP1* | 2 |
| *ZNF330* | 2 |
| *MTMR12* | 2 |
| *ZNF544* | 2 |
| *WBP1* | 2 |
| *SSBP4* | 2 |
| *NSG1* | 2 |
| *SLC19A3* | 2 |
| *SCAPER* | 2 |
| *PODNL1* | 2 |
| *TSNAXIP1* | 2 |
| *STMND1* | 2 |
| *RNF13* | 2 |
| *RNF150* | 2 |
| *SERTM1* | 2 |
| *SH3RF2* | 2 |
| *SLC5A9* | 2 |
| *TRABD2B* | 2 |
| *ZSCAN4* | 2 |
| *TOR4A* | 2 |
| *TSHZ2* | 2 |
| *TNFAIP8L1* | 2 |
| *ZBTB48* | 2 |
| *TRIM2* | 2 |

Note. Degree denotes number of nodes connected to other nodes.
