# Supplementary material for: Integrated bioinformatics analysis for the identification of idiopathic pulmonary fibrosis–related genes and potential therapeutic drugs
Source: BMC Pulm Med. 2023 Oct 4;23:373. doi: 10.1186/s12890-023-02678-z (PMC10552267; doi:10.1186/s12890-023-02678-z)
Supplement: Supplementary file 1 — Additional file 1: Table S1. The analyze network results of 1640 DEGs. Table S2. GO terms of the 18 hub genes. Table S3. KEGG pathways of the 18 hub genes. Table S4. Target microRNAs of SPP1 based on five online miRNA databases. Table S5. Target microRNAs of VEGFA based on five online miRNA databases. Table S6. Target microRNAs of COL1A1 based on five online miRNA databases. Table S7. Target microRNAs of CAV1 based on five online miRNA databases. Table S8. Target microRNAs of PECAM1 based on five online miRNA databases. Table S9. Target microRNAs of BMP4 based on five online miRNA databases. Table S10. Target microRNAs of FYN based on five online miRNA databases. Table S11. Traditional Chinese medicine prediction results of COL1A1. Table S12. Traditional Chinese medicine prediction results of VEGFA. Table S13. Traditional Chinese medicine prediction results of SPP1. [file 12890_2023_2678_MOESM1_ESM.zip › Supplementary Tables/Supplementary Table13.docx]

**Table S13 Traditional Chinese medicine prediction results of *SPP1***

| Gene Symbol | Herb | FDR |
| --- | --- | --- |
| *SPP1* | Sea Buckthorn | 1.20E-03 |
| *SPP1* | rhizome of Gaint Knotweed | 3.96E-03 |
| *SPP1* | Gnaphalium Affine | 4.47E-03 |
| *SPP1* | Pumpkin seed and husks | 7.97E-03 |

Notes. FDR denotes false discovery rate.
