# Supplementary material for: Integrated bioinformatics analysis for the identification of idiopathic pulmonary fibrosis–related genes and potential therapeutic drugs
Source: BMC Pulm Med. 2023 Oct 4;23:373. doi: 10.1186/s12890-023-02678-z (PMC10552267; doi:10.1186/s12890-023-02678-z)
Supplement: Supplementary file 1 — Additional file 1: Table S1. The analyze network results of 1640 DEGs. Table S2. GO terms of the 18 hub genes. Table S3. KEGG pathways of the 18 hub genes. Table S4. Target microRNAs of SPP1 based on five online miRNA databases. Table S5. Target microRNAs of VEGFA based on five online miRNA databases. Table S6. Target microRNAs of COL1A1 based on five online miRNA databases. Table S7. Target microRNAs of CAV1 based on five online miRNA databases. Table S8. Target microRNAs of PECAM1 based on five online miRNA databases. Table S9. Target microRNAs of BMP4 based on five online miRNA databases. Table S10. Target microRNAs of FYN based on five online miRNA databases. Table S11. Traditional Chinese medicine prediction results of COL1A1. Table S12. Traditional Chinese medicine prediction results of VEGFA. Table S13. Traditional Chinese medicine prediction results of SPP1. [file 12890_2023_2678_MOESM1_ESM.zip › Supplementary Tables/Supplementary Table2.docx]

**Table S2 GO terms of the 18 hub genes**

| GO terms |  | FDR |
| --- | --- | --- |
| Biological Processes (BPs) | positive regulation of cell migration | 1.27E-08 |
|  | positive regulation of MAPK cascade | 1.04E-06 |
|  | cell-cell adhesion | 1.08E-04 |
|  | positive regulation of phosphatidylinositol 3-kinase signaling | 1.92E-04 |
|  | positive regulation of gene expression | 3.92E-04 |
|  | platelet degranulation | 3.92E-04 |
|  | positive regulation of cell proliferation | 3.92E-04 |
|  | positive regulation of endothelial cell migration | 1.88E-03 |
|  | positive regulation of protein phosphorylation | 1.88E-03 |
|  | positive regulation of endothelial cell proliferation | 2.27E-03 |
|  | angiogenesis | 3.18E-03 |
|  | extracellular matrix organization | 3.56E-03 |
|  | osteoblast differentiation | 6.81E-03 |
|  | negative regulation of gene expression | 6.81E-03 |
|  | branching morphogenesis of an epithelial tube | 7.84E-03 |
|  | positive regulation of transcription, DNA-templated | 8.50E-03 |
|  | cellular protein metabolic process | 1.09E-02 |
|  | maintenance of permeability of blood-brain barrier | 1.20E-02 |
|  | cell-cell adhesion via plasma-membrane adhesion molecules | 1.62E-02 |
|  | ERK1 and ERK2 cascade | 1.62E-02 |
|  | cell-cell junction assembly | 1.63E-02 |
|  | leukocyte migration | 1.84E-02 |
|  | positive regulation of BMP signaling pathway | 1.98E-02 |
|  | positive regulation of ERK1 and ERK2 cascade | 2.59E-02 |
|  | negative regulation of apoptotic process | 2.64E-02 |
|  | vasculogenesis | 3.31E-02 |
|  | positive regulation of epithelial cell proliferation | 3.36E-02 |
|  | cellular response to transforming growth factor beta stimulus | 3.36E-02 |
|  | vascular endothelial growth factor receptor signaling pathway | 3.36E-02 |
|  | positive regulation of MAP kinase activity | 4.63E-02 |
|  | MAPK cascade | 4.63E-02 |
| Cell Components (CCs) | cell junction | 2.30E-03 |
|  | membrane raft | 2.30E-03 |
|  | adherens junction | 1.12E-02 |
|  | extracellular space | 1.33E-02 |
|  | extracellular region | 1.94E-02 |
|  | extrinsic component of cytoplasmic side of plasma membrane | 1.94E-02 |
|  | platelet alpha granule lumen | 1.94E-02 |
|  | endoplasmic reticulum lumen | 2.41E-02 |
|  | plasma membrane | 2.86E-02 |
|  | focal adhesion | 4.73E-02 |
| Molecular Functions (MFs) | growth factor activity | 1.28E-03 |
|  | extracellular matrix binding | 1.08E-02 |
|  | integrin binding | 1.08E-02 |
|  | identical protein binding | 1.08E-02 |
|  | protein binding | 2.04E-02 |
|  | cadherin binding | 4.40E-02 |
|  | protein phosphatase binding | 4.40E-02 |

Notes. FDR denotes false discovery rate.
