# Supplementary material for: Integrated bioinformatics analysis for the identification of idiopathic pulmonary fibrosis–related genes and potential therapeutic drugs
Source: BMC Pulm Med. 2023 Oct 4;23:373. doi: 10.1186/s12890-023-02678-z (PMC10552267; doi:10.1186/s12890-023-02678-z)
Supplement: Supplementary file 1 — Additional file 1: Table S1. The analyze network results of 1640 DEGs. Table S2. GO terms of the 18 hub genes. Table S3. KEGG pathways of the 18 hub genes. Table S4. Target microRNAs of SPP1 based on five online miRNA databases. Table S5. Target microRNAs of VEGFA based on five online miRNA databases. Table S6. Target microRNAs of COL1A1 based on five online miRNA databases. Table S7. Target microRNAs of CAV1 based on five online miRNA databases. Table S8. Target microRNAs of PECAM1 based on five online miRNA databases. Table S9. Target microRNAs of BMP4 based on five online miRNA databases. Table S10. Target microRNAs of FYN based on five online miRNA databases. Table S11. Traditional Chinese medicine prediction results of COL1A1. Table S12. Traditional Chinese medicine prediction results of VEGFA. Table S13. Traditional Chinese medicine prediction results of SPP1. [file 12890_2023_2678_MOESM1_ESM.zip › Supplementary Tables/Supplementary Table4.docx]

**Table S4 Target microRNAs of *SPP1* based on five online miRNA databases**

| Gene Symbol | microRNA | Database |
| --- | --- | --- |
| *SPP1* | hsa-miR-181c-5p | mirDIP |
| *SPP1* | hsa-miR-4753-3p | mirDIP |
| *SPP1* | hsa-miR-4262 | mirDIP |
| *SPP1* | hsa-miR-5003-5p | mirDIP |
| *SPP1* | hsa-miR-4251 | mirDIP |
| *SPP1* | hsa-miR-17-5p | ENCORI |
| *SPP1* | hsa-miR-20a-5p | ENCORI |
| *SPP1* | hsa-miR-33a-5p | ENCORI |
| *SPP1* | hsa-miR-93-5p | ENCORI |
| *SPP1* | hsa-miR-106a-5p | ENCORI |
| *SPP1* | hsa-miR-10a-5p | ENCORI |
| *SPP1* | hsa-miR-10b-5p | ENCORI |
| *SPP1* | hsa-miR-181a-5p | ENCORI |
| *SPP1* | hsa-miR-181b-5p | ENCORI |
| *SPP1* | hsa-miR-181c-5p | ENCORI |
| *SPP1* | hsa-miR-125b-5p | ENCORI |
| *SPP1* | hsa-miR-142-5p | ENCORI |
| *SPP1* | hsa-miR-125a-5p | ENCORI |
| *SPP1* | hsa-miR-186-5p | ENCORI |
| *SPP1* | hsa-miR-106b-5p | ENCORI |
| *SPP1* | hsa-miR-376c-3p | ENCORI |
| *SPP1* | hsa-miR-377-3p | ENCORI |
| *SPP1* | hsa-miR-323a-3p | ENCORI |
| *SPP1* | hsa-miR-20b-5p | ENCORI |
| *SPP1* | hsa-miR-433-3p | ENCORI |
| *SPP1* | hsa-miR-409-3p | ENCORI |
| *SPP1* | hsa-miR-489-3p | ENCORI |
| *SPP1* | hsa-miR-495-3p | ENCORI |
| *SPP1* | hsa-miR-181d-5p | ENCORI |
| *SPP1* | hsa-miR-498 | ENCORI |
| *SPP1* | hsa-miR-520a-5p | ENCORI |
| *SPP1* | hsa-miR-525-5p | ENCORI |
| *SPP1* | hsa-miR-519d-3p | ENCORI |
| *SPP1* | hsa-miR-520g-3p | ENCORI |
| *SPP1* | hsa-miR-520h | ENCORI |
| *SPP1* | hsa-miR-577 | ENCORI |
| *SPP1* | hsa-miR-579-3p | ENCORI |
| *SPP1* | hsa-miR-580-3p | ENCORI |
| *SPP1* | hsa-miR-582-5p | ENCORI |
| *SPP1* | hsa-miR-33b-5p | ENCORI |
| *SPP1* | hsa-miR-656-3p | ENCORI |
| *SPP1* | hsa-miR-542-3p | ENCORI |
| *SPP1* | hsa-miR-140-3p | ENCORI |
| *SPP1* | hsa-miR-371a-5p | ENCORI |
| *SPP1* | hsa-miR-340-5p | ENCORI |
| *SPP1* | hsa-miR-556-3p | ENCORI |
| *SPP1* | hsa-miR-450b-5p | ENCORI |
| *SPP1* | hsa-miR-888-5p | ENCORI |
| *SPP1* | hsa-miR-944 | ENCORI |
| *SPP1* | hsa-miR-513b-5p | ENCORI |
| *SPP1* | hsa-miR-1179 | ENCORI |
| *SPP1* | hsa-miR-1321 | ENCORI |
| *SPP1* | hsa-miR-3163 | ENCORI |
| *SPP1* | hsa-miR-4262 | ENCORI |
| *SPP1* | hsa-miR-4637 | ENCORI |
| *SPP1* | hsa-miR-4739 | ENCORI |
| *SPP1* | hsa-miR-5688 | ENCORI |
| *SPP1* | hsa-miR-345-3p | ENCORI |
| *SPP1* | hsa-miR-4262 | TargetScan |
| *SPP1* | hsa-miR-181c-5p | TargetScan |
| *SPP1* | hsa-miR-181a-5p | TargetScan |
| *SPP1* | hsa-miR-181d-5p | TargetScan |
| *SPP1* | hsa-miR-181b-5p | TargetScan |
| *SPP1* | hsa-miR-5003-5p | DIANA-micro T |
| *SPP1* | hsa-miR-181c-5p | DIANA-micro T |
| *SPP1* | hsa-miR-3529-3p | DIANA-micro T |
| *SPP1* | hsa-miR-181a-5p | DIANA-micro T |
| *SPP1* | hsa-miR-4753-3p | DIANA-micro T |
| *SPP1* | hsa-miR-4482-3p | DIANA-micro T |
| *SPP1* | hsa-miR-181d-5p | DIANA-micro T |
| *SPP1* | hsa-miR-181b-5p | DIANA-micro T |
| *SPP1* | hsa-miR-7-2-3p | DIANA-micro T |
| *SPP1* | hsa-miR-7-1-3p | DIANA-micro T |
| *SPP1* | hsa-miR-4659a-3p | DIANA-micro T |
| *SPP1* | hsa-miR-4659b-3p | DIANA-micro T |
| *SPP1* | hsa-miR-513a-3p | DIANA-micro T |
| *SPP1* | hsa-miR-513c-3p | DIANA-micro T |
| *SPP1* | hsa-miR-4262 | DIANA-micro T |
| *SPP1* | hsa-miR-3065-5p | DIANA-micro T |
| *SPP1* | hsa-miR-4270 | DIANA-micro T |
| *SPP1* | hsa-miR-4251 | DIANA-micro T |
| *SPP1* | hsa-miR-2117 | DIANA-micro T |
| *SPP1* | hsa-miR-302f | DIANA-micro T |
| *SPP1* | hsa-miR-495-3p | DIANA-micro T |
| *SPP1* | hsa-miR-6732-3p | DIANA-micro T |
| *SPP1* | hsa-miR-6885-3p | DIANA-micro T |
| *SPP1* | hsa-miR-4272 | DIANA-micro T |
| *SPP1* | hsa-miR-5688 | DIANA-micro T |
| *SPP1* | hsa-miR-577 | DIANA-micro T |
| *SPP1* | hsa-miR-4441 | DIANA-micro T |
| *SPP1* | hsa-miR-4786-3p | DIANA-micro T |
| *SPP1* | hsa-miR-664a-3p | DIANA-micro T |
| *SPP1* | hsa-miR-3606-3p | DIANA-micro T |
| *SPP1* | hsa-miR-3163 | DIANA-micro T |
| *SPP1* | hsa-miR-5583-5p | DIANA-micro T |
| *SPP1* | hsa-miR-3613-3p | DIANA-micro T |
| *SPP1* | hsa-miR-3129-3p | DIANA-micro T |
| *SPP1* | hsa-miR-3180-5p | DIANA-micro T |
| *SPP1* | hsa-miR-153-5p | DIANA-micro T |
| *SPP1* | hsa-miR-4712-3p | DIANA-micro T |
| *SPP1* | hsa-miR-6731-3p | DIANA-micro T |
| *SPP1* | hsa-miR-130b-5p | DIANA-micro T |
| *SPP1* | hsa-miR-7154-5p | DIANA-micro T |
| *SPP1* | hsa-miR-6809-3p | DIANA-micro T |
| *SPP1* | hsa-miR-1179 | DIANA-micro T |
| *SPP1* | hsa-miR-4789-5p | DIANA-micro T |
| *SPP1* | hsa-miR-4517 | DIANA-micro T |
| *SPP1* | hsa-miR-5095 | DIANA-micro T |
| *SPP1* | hsa-miR-127-5p | DIANA-micro T |
| *SPP1* | hsa-miR-1287-5p | DIANA-micro T |
| *SPP1* | hsa-miR-6733-5p | DIANA-micro T |
| *SPP1* | hsa-miR-548n | DIANA-micro T |
| *SPP1* | hsa-miR-6875-3p | DIANA-micro T |
| *SPP1* | hsa-miR-4307 | DIANA-micro T |
| *SPP1* | hsa-miR-7159-3p | DIANA-micro T |
| *SPP1* | hsa-let-7a-2-3p | miRWalk |
| *SPP1* | hsa-let-7b-5p | miRWalk |
| *SPP1* | hsa-let-7c-5p | miRWalk |
| *SPP1* | hsa-let-7d-5p | miRWalk |
| *SPP1* | hsa-let-7e-5p | miRWalk |
| *SPP1* | hsa-let-7e-3p | miRWalk |
| *SPP1* | hsa-miR-15a-3p | miRWalk |
| *SPP1* | hsa-miR-19a-3p | miRWalk |
| *SPP1* | hsa-miR-19b-1-5p | miRWalk |
| *SPP1* | hsa-miR-19b-2-5p | miRWalk |
| *SPP1* | hsa-miR-21-5p | miRWalk |
| *SPP1* | hsa-miR-21-3p | miRWalk |
| *SPP1* | hsa-miR-23a-5p | miRWalk |
| *SPP1* | hsa-miR-24-1-5p | miRWalk |
| *SPP1* | hsa-miR-24-3p | miRWalk |
| *SPP1* | hsa-miR-25-5p | miRWalk |
| *SPP1* | hsa-miR-25-3p | miRWalk |
| *SPP1* | hsa-miR-26b-3p | miRWalk |
| *SPP1* | hsa-miR-27a-5p | miRWalk |
| *SPP1* | hsa-miR-27a-3p | miRWalk |
| *SPP1* | hsa-miR-92a-1-5p | miRWalk |
| *SPP1* | hsa-miR-92a-3p | miRWalk |
| *SPP1* | hsa-miR-92a-2-5p | miRWalk |
| *SPP1* | hsa-miR-96-5p | miRWalk |
| *SPP1* | hsa-miR-99a-3p | miRWalk |
| *SPP1* | hsa-miR-100-3p | miRWalk |
| *SPP1* | hsa-miR-29b-1-5p | miRWalk |
| *SPP1* | hsa-miR-29b-2-5p | miRWalk |
| *SPP1* | hsa-miR-103a-2-5p | miRWalk |
| *SPP1* | hsa-miR-105-5p | miRWalk |
| *SPP1* | hsa-miR-107 | miRWalk |
| *SPP1* | hsa-miR-197-5p | miRWalk |
| *SPP1* | hsa-miR-198 | miRWalk |
| *SPP1* | hsa-miR-208a-5p | miRWalk |
| *SPP1* | hsa-miR-129-5p | miRWalk |
| *SPP1* | hsa-miR-30c-2-3p | miRWalk |
| *SPP1* | hsa-miR-139-3p | miRWalk |
| *SPP1* | hsa-miR-181a-5p | miRWalk |
| *SPP1* | hsa-miR-181a-2-3p | miRWalk |
| *SPP1* | hsa-miR-181b-5p | miRWalk |
| *SPP1* | hsa-miR-181c-5p | miRWalk |
| *SPP1* | hsa-miR-182-5p | miRWalk |
| *SPP1* | hsa-miR-183-5p | miRWalk |
| *SPP1* | hsa-miR-187-3p | miRWalk |
| *SPP1* | hsa-miR-199b-5p | miRWalk |
| *SPP1* | hsa-miR-204-3p | miRWalk |
| *SPP1* | hsa-miR-205-3p | miRWalk |
| *SPP1* | hsa-miR-210-3p | miRWalk |
| *SPP1* | hsa-miR-211-5p | miRWalk |
| *SPP1* | hsa-miR-211-3p | miRWalk |
| *SPP1* | hsa-miR-214-5p | miRWalk |
| *SPP1* | hsa-miR-215-3p | miRWalk |
| *SPP1* | hsa-miR-216a-5p | miRWalk |
| *SPP1* | hsa-miR-216a-3p | miRWalk |
| *SPP1* | hsa-miR-217-5p | miRWalk |
| *SPP1* | hsa-miR-218-1-3p | miRWalk |
| *SPP1* | hsa-miR-218-2-3p | miRWalk |
| *SPP1* | hsa-miR-219a-1-3p | miRWalk |
| *SPP1* | hsa-miR-221-3p | miRWalk |
| *SPP1* | hsa-miR-200b-5p | miRWalk |
| *SPP1* | hsa-let-7g-3p | miRWalk |
| *SPP1* | hsa-let-7i-3p | miRWalk |
| *SPP1* | hsa-miR-23b-5p | miRWalk |
| *SPP1* | hsa-miR-27b-5p | miRWalk |
| *SPP1* | hsa-miR-27b-3p | miRWalk |
| *SPP1* | hsa-miR-30b-3p | miRWalk |
| *SPP1* | hsa-miR-124-5p | miRWalk |
| *SPP1* | hsa-miR-124-3p | miRWalk |
| *SPP1* | hsa-miR-125b-1-3p | miRWalk |
| *SPP1* | hsa-miR-128-1-5p | miRWalk |
| *SPP1* | hsa-miR-130a-5p | miRWalk |
| *SPP1* | hsa-miR-132-5p | miRWalk |
| *SPP1* | hsa-miR-132-3p | miRWalk |
| *SPP1* | hsa-miR-135a-3p | miRWalk |
| *SPP1* | hsa-miR-135a-2-3p | miRWalk |
| *SPP1* | hsa-miR-137-5p | miRWalk |
| *SPP1* | hsa-miR-138-5p | miRWalk |
| *SPP1* | hsa-miR-143-5p | miRWalk |
| *SPP1* | hsa-miR-145-3p | miRWalk |
| *SPP1* | hsa-miR-191-3p | miRWalk |
| *SPP1* | hsa-miR-125a-3p | miRWalk |
| *SPP1* | hsa-miR-127-3p | miRWalk |
| *SPP1* | hsa-miR-134-5p | miRWalk |
| *SPP1* | hsa-miR-134-3p | miRWalk |
| *SPP1* | hsa-miR-136-3p | miRWalk |
| *SPP1* | hsa-miR-149-5p | miRWalk |
| *SPP1* | hsa-miR-149-3p | miRWalk |
| *SPP1* | hsa-miR-150-3p | miRWalk |
| *SPP1* | hsa-miR-154-5p | miRWalk |
| *SPP1* | hsa-miR-154-3p | miRWalk |
| *SPP1* | hsa-miR-184 | miRWalk |
| *SPP1* | hsa-miR-185-5p | miRWalk |
| *SPP1* | hsa-miR-185-3p | miRWalk |
| *SPP1* | hsa-miR-193a-5p | miRWalk |
| *SPP1* | hsa-miR-193a-3p | miRWalk |
| *SPP1* | hsa-miR-320a-5p | miRWalk |
| *SPP1* | hsa-miR-320a-3p | miRWalk |
| *SPP1* | hsa-miR-194-3p | miRWalk |
| *SPP1* | hsa-miR-29c-5p | miRWalk |
| *SPP1* | hsa-miR-29c-3p | miRWalk |
| *SPP1* | hsa-miR-30c-1-3p | miRWalk |
| *SPP1* | hsa-miR-200a-3p | miRWalk |
| *SPP1* | hsa-miR-302a-3p | miRWalk |
| *SPP1* | hsa-miR-101-2-5p | miRWalk |
| *SPP1* | hsa-miR-219a-2-3p | miRWalk |
| *SPP1* | hsa-miR-34b-5p | miRWalk |
| *SPP1* | hsa-miR-34c-5p | miRWalk |
| *SPP1* | hsa-miR-34c-3p | miRWalk |
| *SPP1* | hsa-miR-99b-3p | miRWalk |
| *SPP1* | hsa-miR-296-3p | miRWalk |
| *SPP1* | hsa-miR-130b-5p | miRWalk |
| *SPP1* | hsa-miR-361-5p | miRWalk |
| *SPP1* | hsa-miR-362-5p | miRWalk |
| *SPP1* | hsa-miR-365a-5p | miRWalk |
| *SPP1* | hsa-miR-365b-5p | miRWalk |
| *SPP1* | hsa-miR-302c-3p | miRWalk |
| *SPP1* | hsa-miR-302d-3p | miRWalk |
| *SPP1* | hsa-miR-371a-5p | miRWalk |
| *SPP1* | hsa-miR-372-3p | miRWalk |
| *SPP1* | hsa-miR-377-5p | miRWalk |
| *SPP1* | hsa-miR-378a-5p | miRWalk |
| *SPP1* | hsa-miR-380-5p | miRWalk |
| *SPP1* | hsa-miR-382-5p | miRWalk |
| *SPP1* | hsa-miR-383-5p | miRWalk |
| *SPP1* | hsa-miR-383-3p | miRWalk |
| *SPP1* | hsa-miR-330-5p | miRWalk |
| *SPP1* | hsa-miR-328-5p | miRWalk |
| *SPP1* | hsa-miR-323a-5p | miRWalk |
| *SPP1* | hsa-miR-323a-3p | miRWalk |
| *SPP1* | hsa-miR-151a-5p | miRWalk |
| *SPP1* | hsa-miR-331-5p | miRWalk |
| *SPP1* | hsa-miR-324-3p | miRWalk |
| *SPP1* | hsa-miR-338-3p | miRWalk |
| *SPP1* | hsa-miR-339-5p | miRWalk |
| *SPP1* | hsa-miR-133b | miRWalk |
| *SPP1* | hsa-miR-345-5p | miRWalk |
| *SPP1* | hsa-miR-345-3p | miRWalk |
| *SPP1* | hsa-miR-346 | miRWalk |
| *SPP1* | hsa-miR-196b-3p | miRWalk |
| *SPP1* | hsa-miR-422a | miRWalk |
| *SPP1* | hsa-miR-423-5p | miRWalk |
| *SPP1* | hsa-miR-423-3p | miRWalk |
| *SPP1* | hsa-miR-425-5p | miRWalk |
| *SPP1* | hsa-miR-20b-3p | miRWalk |
| *SPP1* | hsa-miR-431-5p | miRWalk |
| *SPP1* | hsa-miR-431-3p | miRWalk |
| *SPP1* | hsa-miR-433-3p | miRWalk |
| *SPP1* | hsa-miR-329-5p | miRWalk |
| *SPP1* | hsa-miR-412-5p | miRWalk |
| *SPP1* | hsa-miR-410-5p | miRWalk |
| *SPP1* | hsa-miR-410-3p | miRWalk |
| *SPP1* | hsa-miR-483-3p | miRWalk |
| *SPP1* | hsa-miR-484 | miRWalk |
| *SPP1* | hsa-miR-485-5p | miRWalk |
| *SPP1* | hsa-miR-485-3p | miRWalk |
| *SPP1* | hsa-miR-487a-5p | miRWalk |
| *SPP1* | hsa-miR-487a-3p | miRWalk |
| *SPP1* | hsa-miR-491-5p | miRWalk |
| *SPP1* | hsa-miR-511-5p | miRWalk |
| *SPP1* | hsa-miR-146b-3p | miRWalk |
| *SPP1* | hsa-miR-202-3p | miRWalk |
| *SPP1* | hsa-miR-493-3p | miRWalk |
| *SPP1* | hsa-miR-432-5p | miRWalk |
| *SPP1* | hsa-miR-494-5p | miRWalk |
| *SPP1* | hsa-miR-193b-5p | miRWalk |
| *SPP1* | hsa-miR-497-3p | miRWalk |
| *SPP1* | hsa-miR-181d-5p | miRWalk |
| *SPP1* | hsa-miR-512-5p | miRWalk |
| *SPP1* | hsa-miR-498-5p | miRWalk |
| *SPP1* | hsa-miR-515-3p | miRWalk |
| *SPP1* | hsa-miR-519e-3p | miRWalk |
| *SPP1* | hsa-miR-519c-5p | miRWalk |
| *SPP1* | hsa-miR-519b-5p | miRWalk |
| *SPP1* | hsa-miR-525-5p | miRWalk |
| *SPP1* | hsa-miR-525-3p | miRWalk |
| *SPP1* | hsa-miR-523-5p | miRWalk |
| *SPP1* | hsa-miR-518f-5p | miRWalk |
| *SPP1* | hsa-miR-518f-3p | miRWalk |
| *SPP1* | hsa-miR-526a-5p | miRWalk |
| *SPP1* | hsa-miR-520c-5p | miRWalk |
| *SPP1* | hsa-miR-518c-5p | miRWalk |
| *SPP1* | hsa-miR-518c-3p | miRWalk |
| *SPP1* | hsa-miR-524-3p | miRWalk |
| *SPP1* | hsa-miR-519d-5p | miRWalk |
| *SPP1* | hsa-miR-520d-3p | miRWalk |
| *SPP1* | hsa-miR-516b-5p | miRWalk |
| *SPP1* | hsa-miR-516b-3p | miRWalk |
| *SPP1* | hsa-miR-518e-5p | miRWalk |
| *SPP1* | hsa-miR-518e-3p | miRWalk |
| *SPP1* | hsa-miR-518a-3p | miRWalk |
| *SPP1* | hsa-miR-518d-5p | miRWalk |
| *SPP1* | hsa-miR-522-5p | miRWalk |
| *SPP1* | hsa-miR-519a-5p | miRWalk |
| *SPP1* | hsa-miR-516a-3p | miRWalk |
| *SPP1* | hsa-miR-500a-5p | miRWalk |
| *SPP1* | hsa-miR-500a-3p | miRWalk |
| *SPP1* | hsa-miR-502-5p | miRWalk |
| *SPP1* | hsa-miR-502-3p | miRWalk |
| *SPP1* | hsa-miR-503-3p | miRWalk |
| *SPP1* | hsa-miR-504-5p | miRWalk |
| *SPP1* | hsa-miR-504-3p | miRWalk |
| *SPP1* | hsa-miR-513a-5p | miRWalk |
| *SPP1* | hsa-miR-508-5p | miRWalk |
| *SPP1* | hsa-miR-509-3p | miRWalk |
| *SPP1* | hsa-miR-510-5p | miRWalk |
| *SPP1* | hsa-miR-455-3p | miRWalk |
| *SPP1* | hsa-miR-487b-3p | miRWalk |
| *SPP1* | hsa-miR-552-5p | miRWalk |
| *SPP1* | hsa-miR-552-3p | miRWalk |
| *SPP1* | hsa-miR-92b-3p | miRWalk |
| *SPP1* | hsa-miR-557 | miRWalk |
| *SPP1* | hsa-miR-558 | miRWalk |
| *SPP1* | hsa-miR-564 | miRWalk |
| *SPP1* | hsa-miR-551b-5p | miRWalk |
| *SPP1* | hsa-miR-569 | miRWalk |
| *SPP1* | hsa-miR-571 | miRWalk |
| *SPP1* | hsa-miR-572 | miRWalk |
| *SPP1* | hsa-miR-579-5p | miRWalk |
| *SPP1* | hsa-miR-580-5p | miRWalk |
| *SPP1* | hsa-miR-584-5p | miRWalk |
| *SPP1* | hsa-miR-587 | miRWalk |
| *SPP1* | hsa-miR-588 | miRWalk |
| *SPP1* | hsa-miR-589-3p | miRWalk |
| *SPP1* | hsa-miR-550a-5p | miRWalk |
| *SPP1* | hsa-miR-550a-3p | miRWalk |
| *SPP1* | hsa-miR-593-3p | miRWalk |
| *SPP1* | hsa-miR-596 | miRWalk |
| *SPP1* | hsa-miR-597-3p | miRWalk |
| *SPP1* | hsa-miR-598-5p | miRWalk |
| *SPP1* | hsa-miR-598-3p | miRWalk |
| *SPP1* | hsa-miR-599 | miRWalk |
| *SPP1* | hsa-miR-601 | miRWalk |
| *SPP1* | hsa-miR-602 | miRWalk |
| *SPP1* | hsa-miR-603 | miRWalk |
| *SPP1* | hsa-miR-604 | miRWalk |
| *SPP1* | hsa-miR-605-5p | miRWalk |
| *SPP1* | hsa-miR-605-3p | miRWalk |
| *SPP1* | hsa-miR-608 | miRWalk |
| *SPP1* | hsa-miR-609 | miRWalk |
| *SPP1* | hsa-miR-612 | miRWalk |
| *SPP1* | hsa-miR-615-3p | miRWalk |
| *SPP1* | hsa-miR-616-3p | miRWalk |
| *SPP1* | hsa-miR-619-5p | miRWalk |
| *SPP1* | hsa-miR-619-3p | miRWalk |
| *SPP1* | hsa-miR-621 | miRWalk |
| *SPP1* | hsa-miR-622 | miRWalk |
| *SPP1* | hsa-miR-623 | miRWalk |
| *SPP1* | hsa-miR-629-5p | miRWalk |
| *SPP1* | hsa-miR-33b-3p | miRWalk |
| *SPP1* | hsa-miR-632 | miRWalk |
| *SPP1* | hsa-miR-634 | miRWalk |
| *SPP1* | hsa-miR-637 | miRWalk |
| *SPP1* | hsa-miR-642a-3p | miRWalk |
| *SPP1* | hsa-miR-643 | miRWalk |
| *SPP1* | hsa-miR-646 | miRWalk |
| *SPP1* | hsa-miR-647 | miRWalk |
| *SPP1* | hsa-miR-648 | miRWalk |
| *SPP1* | hsa-miR-649 | miRWalk |
| *SPP1* | hsa-miR-650 | miRWalk |
| *SPP1* | hsa-miR-652-5p | miRWalk |
| *SPP1* | hsa-miR-652-3p | miRWalk |
| *SPP1* | hsa-miR-661 | miRWalk |
| *SPP1* | hsa-miR-662 | miRWalk |
| *SPP1* | hsa-miR-663a | miRWalk |
| *SPP1* | hsa-miR-449b-3p | miRWalk |
| *SPP1* | hsa-miR-654-5p | miRWalk |
| *SPP1* | hsa-miR-656-5p | miRWalk |
| *SPP1* | hsa-miR-549a-5p | miRWalk |
| *SPP1* | hsa-miR-657 | miRWalk |
| *SPP1* | hsa-miR-658 | miRWalk |
| *SPP1* | hsa-miR-659-5p | miRWalk |
| *SPP1* | hsa-miR-659-3p | miRWalk |
| *SPP1* | hsa-miR-660-3p | miRWalk |
| *SPP1* | hsa-miR-542-5p | miRWalk |
| *SPP1* | hsa-miR-758-5p | miRWalk |
| *SPP1* | hsa-miR-758-3p | miRWalk |
| *SPP1* | hsa-miR-671-5p | miRWalk |
| *SPP1* | hsa-miR-671-3p | miRWalk |
| *SPP1* | hsa-miR-668-5p | miRWalk |
| *SPP1* | hsa-miR-668-3p | miRWalk |
| *SPP1* | hsa-miR-550a-3-5p | miRWalk |
| *SPP1* | hsa-miR-767-5p | miRWalk |
| *SPP1* | hsa-miR-1224-5p | miRWalk |
| *SPP1* | hsa-miR-320b | miRWalk |
| *SPP1* | hsa-miR-320c | miRWalk |
| *SPP1* | hsa-miR-1296-5p | miRWalk |
| *SPP1* | hsa-miR-1296-3p | miRWalk |
| *SPP1* | hsa-miR-1468-5p | miRWalk |
| *SPP1* | hsa-miR-1271-3p | miRWalk |
| *SPP1* | hsa-miR-1301-3p | miRWalk |
| *SPP1* | hsa-miR-1185-2-3p | miRWalk |
| *SPP1* | hsa-miR-449c-5p | miRWalk |
| *SPP1* | hsa-miR-449c-3p | miRWalk |
| *SPP1* | hsa-miR-769-5p | miRWalk |
| *SPP1* | hsa-miR-769-3p | miRWalk |
| *SPP1* | hsa-miR-766-5p | miRWalk |
| *SPP1* | hsa-miR-762 | miRWalk |
| *SPP1* | hsa-miR-670-5p | miRWalk |
| *SPP1* | hsa-miR-1298-5p | miRWalk |
| *SPP1* | hsa-miR-1298-3p | miRWalk |
| *SPP1* | hsa-miR-2113 | miRWalk |
| *SPP1* | hsa-miR-761 | miRWalk |
| *SPP1* | hsa-miR-765 | miRWalk |
| *SPP1* | hsa-miR-770-5p | miRWalk |
| *SPP1* | hsa-miR-675-5p | miRWalk |
| *SPP1* | hsa-miR-298 | miRWalk |
| *SPP1* | hsa-miR-891a-5p | miRWalk |
| *SPP1* | hsa-miR-891a-3p | miRWalk |
| *SPP1* | hsa-miR-892a | miRWalk |
| *SPP1* | hsa-miR-874-5p | miRWalk |
| *SPP1* | hsa-miR-874-3p | miRWalk |
| *SPP1* | hsa-miR-891b | miRWalk |
| *SPP1* | hsa-miR-892b | miRWalk |
| *SPP1* | hsa-miR-541-5p | miRWalk |
| *SPP1* | hsa-miR-541-3p | miRWalk |
| *SPP1* | hsa-miR-889-5p | miRWalk |
| *SPP1* | hsa-miR-875-3p | miRWalk |
| *SPP1* | hsa-miR-190b-3p | miRWalk |
| *SPP1* | hsa-miR-744-5p | miRWalk |
| *SPP1* | hsa-miR-885-3p | miRWalk |
| *SPP1* | hsa-miR-877-5p | miRWalk |
| *SPP1* | hsa-miR-665 | miRWalk |
| *SPP1* | hsa-miR-374b-3p | miRWalk |
| *SPP1* | hsa-miR-760 | miRWalk |
| *SPP1* | hsa-miR-301b-5p | miRWalk |
| *SPP1* | hsa-miR-920 | miRWalk |
| *SPP1* | hsa-miR-921 | miRWalk |
| *SPP1* | hsa-miR-924 | miRWalk |
| *SPP1* | hsa-miR-933 | miRWalk |
| *SPP1* | hsa-miR-934 | miRWalk |
| *SPP1* | hsa-miR-937-5p | miRWalk |
| *SPP1* | hsa-miR-937-3p | miRWalk |
| *SPP1* | hsa-miR-939-5p | miRWalk |
| *SPP1* | hsa-miR-941 | miRWalk |
| *SPP1* | hsa-miR-942-5p | miRWalk |
| *SPP1* | hsa-miR-1178-5p | miRWalk |
| *SPP1* | hsa-miR-1178-3p | miRWalk |
| *SPP1* | hsa-miR-1179 | miRWalk |
| *SPP1* | hsa-miR-1180-3p | miRWalk |
| *SPP1* | hsa-miR-1182 | miRWalk |
| *SPP1* | hsa-miR-1183 | miRWalk |
| *SPP1* | hsa-miR-1184 | miRWalk |
| *SPP1* | hsa-miR-1225-5p | miRWalk |
| *SPP1* | hsa-miR-1226-5p | miRWalk |
| *SPP1* | hsa-miR-1227-5p | miRWalk |
| *SPP1* | hsa-miR-1228-5p | miRWalk |
| *SPP1* | hsa-miR-1228-3p | miRWalk |
| *SPP1* | hsa-miR-1229-5p | miRWalk |
| *SPP1* | hsa-miR-1229-3p | miRWalk |
| *SPP1* | hsa-miR-1231 | miRWalk |
| *SPP1* | hsa-miR-1233-5p | miRWalk |
| *SPP1* | hsa-miR-1233-3p | miRWalk |
| *SPP1* | hsa-miR-1234-3p | miRWalk |
| *SPP1* | hsa-miR-1237-5p | miRWalk |
| *SPP1* | hsa-miR-1238-5p | miRWalk |
| *SPP1* | hsa-miR-1238-3p | miRWalk |
| *SPP1* | hsa-miR-1200 | miRWalk |
| *SPP1* | hsa-miR-1203 | miRWalk |
| *SPP1* | hsa-miR-663b | miRWalk |
| *SPP1* | hsa-miR-1204 | miRWalk |
| *SPP1* | hsa-miR-1207-5p | miRWalk |
| *SPP1* | hsa-miR-1207-3p | miRWalk |
| *SPP1* | hsa-miR-1285-3p | miRWalk |
| *SPP1* | hsa-miR-1287-5p | miRWalk |
| *SPP1* | hsa-miR-1289 | miRWalk |
| *SPP1* | hsa-miR-1290 | miRWalk |
| *SPP1* | hsa-miR-1291 | miRWalk |
| *SPP1* | hsa-miR-1293 | miRWalk |
| *SPP1* | hsa-miR-1294 | miRWalk |
| *SPP1* | hsa-miR-1295a | miRWalk |
| *SPP1* | hsa-miR-1299 | miRWalk |
| *SPP1* | hsa-miR-548l | miRWalk |
| *SPP1* | hsa-miR-1304-3p | miRWalk |
| *SPP1* | hsa-miR-1305 | miRWalk |
| *SPP1* | hsa-miR-1243 | miRWalk |
| *SPP1* | hsa-miR-1244 | miRWalk |
| *SPP1* | hsa-miR-1249-5p | miRWalk |
| *SPP1* | hsa-miR-1250-5p | miRWalk |
| *SPP1* | hsa-miR-1251-3p | miRWalk |
| *SPP1* | hsa-miR-1253 | miRWalk |
| *SPP1* | hsa-miR-548g-5p | miRWalk |
| *SPP1* | hsa-miR-1263 | miRWalk |
| *SPP1* | hsa-miR-1265 | miRWalk |
| *SPP1* | hsa-miR-548o-3p | miRWalk |
| *SPP1* | hsa-miR-1266-5p | miRWalk |
| *SPP1* | hsa-miR-1266-3p | miRWalk |
| *SPP1* | hsa-miR-1268a | miRWalk |
| *SPP1* | hsa-miR-1269a | miRWalk |
| *SPP1* | hsa-miR-1270 | miRWalk |
| *SPP1* | hsa-miR-548h-3p | miRWalk |
| *SPP1* | hsa-miR-1275 | miRWalk |
| *SPP1* | hsa-miR-1281 | miRWalk |
| *SPP1* | hsa-miR-1282 | miRWalk |
| *SPP1* | hsa-miR-1288-5p | miRWalk |
| *SPP1* | hsa-miR-1288-3p | miRWalk |
| *SPP1* | hsa-miR-1292-5p | miRWalk |
| *SPP1* | hsa-miR-1292-3p | miRWalk |
| *SPP1* | hsa-miR-1306-3p | miRWalk |
| *SPP1* | hsa-miR-1307-3p | miRWalk |
| *SPP1* | hsa-miR-513c-5p | miRWalk |
| *SPP1* | hsa-miR-1321 | miRWalk |
| *SPP1* | hsa-miR-1322 | miRWalk |
| *SPP1* | hsa-miR-1197 | miRWalk |
| *SPP1* | hsa-miR-1537-5p | miRWalk |
| *SPP1* | hsa-miR-1538 | miRWalk |
| *SPP1* | hsa-miR-1539 | miRWalk |
| *SPP1* | hsa-miR-103b | miRWalk |
| *SPP1* | hsa-miR-1827 | miRWalk |
| *SPP1* | hsa-miR-1908-5p | miRWalk |
| *SPP1* | hsa-miR-1909-3p | miRWalk |
| *SPP1* | hsa-miR-1910-5p | miRWalk |
| *SPP1* | hsa-miR-1910-3p | miRWalk |
| *SPP1* | hsa-miR-1912-5p | miRWalk |
| *SPP1* | hsa-miR-1913 | miRWalk |
| *SPP1* | hsa-miR-1914-5p | miRWalk |
| *SPP1* | hsa-miR-1914-3p | miRWalk |
| *SPP1* | hsa-miR-1915-5p | miRWalk |
| *SPP1* | hsa-miR-1976 | miRWalk |
| *SPP1* | hsa-miR-2110 | miRWalk |
| *SPP1* | hsa-miR-2115-3p | miRWalk |
| *SPP1* | hsa-miR-548q | miRWalk |
| *SPP1* | hsa-miR-2276-5p | miRWalk |
| *SPP1* | hsa-miR-2276-3p | miRWalk |
| *SPP1* | hsa-miR-2277-5p | miRWalk |
| *SPP1* | hsa-miR-2277-3p | miRWalk |
| *SPP1* | hsa-miR-2278 | miRWalk |
| *SPP1* | hsa-miR-2681-5p | miRWalk |
| *SPP1* | hsa-miR-2682-5p | miRWalk |
| *SPP1* | hsa-miR-2682-3p | miRWalk |
| *SPP1* | hsa-miR-718 | miRWalk |
| *SPP1* | hsa-miR-2861 | miRWalk |
| *SPP1* | hsa-miR-3120-5p | miRWalk |
| *SPP1* | hsa-miR-3122 | miRWalk |
| *SPP1* | hsa-miR-3124-5p | miRWalk |
| *SPP1* | hsa-miR-3125 | miRWalk |
| *SPP1* | hsa-miR-3126-3p | miRWalk |
| *SPP1* | hsa-miR-3127-5p | miRWalk |
| *SPP1* | hsa-miR-3127-3p | miRWalk |
| *SPP1* | hsa-miR-3130-5p | miRWalk |
| *SPP1* | hsa-miR-3130-3p | miRWalk |
| *SPP1* | hsa-miR-3131 | miRWalk |
| *SPP1* | hsa-miR-378b | miRWalk |
| *SPP1* | hsa-miR-3135a | miRWalk |
| *SPP1* | hsa-miR-544b | miRWalk |
| *SPP1* | hsa-miR-3137 | miRWalk |
| *SPP1* | hsa-miR-3138 | miRWalk |
| *SPP1* | hsa-miR-3139 | miRWalk |
| *SPP1* | hsa-miR-3140-3p | miRWalk |
| *SPP1* | hsa-miR-548t-5p | miRWalk |
| *SPP1* | hsa-miR-548t-3p | miRWalk |
| *SPP1* | hsa-miR-3141 | miRWalk |
| *SPP1* | hsa-miR-3144-5p | miRWalk |
| *SPP1* | hsa-miR-3147 | miRWalk |
| *SPP1* | hsa-miR-3150a-3p | miRWalk |
| *SPP1* | hsa-miR-3151-5p | miRWalk |
| *SPP1* | hsa-miR-3151-3p | miRWalk |
| *SPP1* | hsa-miR-3153 | miRWalk |
| *SPP1* | hsa-miR-3074-5p | miRWalk |
| *SPP1* | hsa-miR-3154 | miRWalk |
| *SPP1* | hsa-miR-3155a | miRWalk |
| *SPP1* | hsa-miR-3156-5p | miRWalk |
| *SPP1* | hsa-miR-3156-3p | miRWalk |
| *SPP1* | hsa-miR-3157-5p | miRWalk |
| *SPP1* | hsa-miR-3157-3p | miRWalk |
| *SPP1* | hsa-miR-3158-3p | miRWalk |
| *SPP1* | hsa-miR-3159 | miRWalk |
| *SPP1* | hsa-miR-3162-5p | miRWalk |
| *SPP1* | hsa-miR-3165 | miRWalk |
| *SPP1* | hsa-miR-1260b | miRWalk |
| *SPP1* | hsa-miR-3169 | miRWalk |
| *SPP1* | hsa-miR-3173-5p | miRWalk |
| *SPP1* | hsa-miR-1193 | miRWalk |
| *SPP1* | hsa-miR-323b-5p | miRWalk |
| *SPP1* | hsa-miR-3175 | miRWalk |
| *SPP1* | hsa-miR-3176 | miRWalk |
| *SPP1* | hsa-miR-3177-3p | miRWalk |
| *SPP1* | hsa-miR-3178 | miRWalk |
| *SPP1* | hsa-miR-3180-5p | miRWalk |
| *SPP1* | hsa-miR-3180-3p | miRWalk |
| *SPP1* | hsa-miR-3181 | miRWalk |
| *SPP1* | hsa-miR-3183 | miRWalk |
| *SPP1* | hsa-miR-3184-5p | miRWalk |
| *SPP1* | hsa-miR-3184-3p | miRWalk |
| *SPP1* | hsa-miR-3185 | miRWalk |
| *SPP1* | hsa-miR-3186-5p | miRWalk |
| *SPP1* | hsa-miR-3186-3p | miRWalk |
| *SPP1* | hsa-miR-3187-5p | miRWalk |
| *SPP1* | hsa-miR-3187-3p | miRWalk |
| *SPP1* | hsa-miR-3188 | miRWalk |
| *SPP1* | hsa-miR-3189-5p | miRWalk |
| *SPP1* | hsa-miR-3189-3p | miRWalk |
| *SPP1* | hsa-miR-320e | miRWalk |
| *SPP1* | hsa-miR-3190-3p | miRWalk |
| *SPP1* | hsa-miR-3191-5p | miRWalk |
| *SPP1* | hsa-miR-3191-3p | miRWalk |
| *SPP1* | hsa-miR-3192-5p | miRWalk |
| *SPP1* | hsa-miR-3193 | miRWalk |
| *SPP1* | hsa-miR-3194-3p | miRWalk |
| *SPP1* | hsa-miR-3196 | miRWalk |
| *SPP1* | hsa-miR-548x-5p | miRWalk |
| *SPP1* | hsa-miR-3197 | miRWalk |
| *SPP1* | hsa-miR-3198 | miRWalk |
| *SPP1* | hsa-miR-3199 | miRWalk |
| *SPP1* | hsa-miR-3200-5p | miRWalk |
| *SPP1* | hsa-miR-3200-3p | miRWalk |
| *SPP1* | hsa-miR-514b-5p | miRWalk |
| *SPP1* | hsa-miR-4296 | miRWalk |
| *SPP1* | hsa-miR-4297 | miRWalk |
| *SPP1* | hsa-miR-4294 | miRWalk |
| *SPP1* | hsa-miR-4298 | miRWalk |
| *SPP1* | hsa-miR-4304 | miRWalk |
| *SPP1* | hsa-miR-4302 | miRWalk |
| *SPP1* | hsa-miR-4309 | miRWalk |
| *SPP1* | hsa-miR-4308 | miRWalk |
| *SPP1* | hsa-miR-4310 | miRWalk |
| *SPP1* | hsa-miR-4312 | miRWalk |
| *SPP1* | hsa-miR-4316 | miRWalk |
| *SPP1* | hsa-miR-4318 | miRWalk |
| *SPP1* | hsa-miR-4320 | miRWalk |
| *SPP1* | hsa-miR-4322 | miRWalk |
| *SPP1* | hsa-miR-4321 | miRWalk |
| *SPP1* | hsa-miR-4323 | miRWalk |
| *SPP1* | hsa-miR-4256 | miRWalk |
| *SPP1* | hsa-miR-4259 | miRWalk |
| *SPP1* | hsa-miR-4260 | miRWalk |
| *SPP1* | hsa-miR-4253 | miRWalk |
| *SPP1* | hsa-miR-4326 | miRWalk |
| *SPP1* | hsa-miR-4327 | miRWalk |
| *SPP1* | hsa-miR-4265 | miRWalk |
| *SPP1* | hsa-miR-4266 | miRWalk |
| *SPP1* | hsa-miR-2355-3p | miRWalk |
| *SPP1* | hsa-miR-4270 | miRWalk |
| *SPP1* | hsa-miR-4281 | miRWalk |
| *SPP1* | hsa-miR-4278 | miRWalk |
| *SPP1* | hsa-miR-4280 | miRWalk |
| *SPP1* | hsa-miR-4285 | miRWalk |
| *SPP1* | hsa-miR-500b-5p | miRWalk |
| *SPP1* | hsa-miR-3605-5p | miRWalk |
| *SPP1* | hsa-miR-3612 | miRWalk |
| *SPP1* | hsa-miR-3614-5p | miRWalk |
| *SPP1* | hsa-miR-3614-3p | miRWalk |
| *SPP1* | hsa-miR-3615 | miRWalk |
| *SPP1* | hsa-miR-3616-3p | miRWalk |
| *SPP1* | hsa-miR-3619-5p | miRWalk |
| *SPP1* | hsa-miR-3619-3p | miRWalk |
| *SPP1* | hsa-miR-3620-5p | miRWalk |
| *SPP1* | hsa-miR-3621 | miRWalk |
| *SPP1* | hsa-miR-3622a-3p | miRWalk |
| *SPP1* | hsa-miR-3622b-5p | miRWalk |
| *SPP1* | hsa-miR-3648 | miRWalk |
| *SPP1* | hsa-miR-3649 | miRWalk |
| *SPP1* | hsa-miR-3652 | miRWalk |
| *SPP1* | hsa-miR-3654 | miRWalk |
| *SPP1* | hsa-miR-3655 | miRWalk |
| *SPP1* | hsa-miR-3659 | miRWalk |
| *SPP1* | hsa-miR-3661 | miRWalk |
| *SPP1* | hsa-miR-3663-5p | miRWalk |
| *SPP1* | hsa-miR-3663-3p | miRWalk |
| *SPP1* | hsa-miR-3664-3p | miRWalk |
| *SPP1* | hsa-miR-3665 | miRWalk |
| *SPP1* | hsa-miR-3667-3p | miRWalk |
| *SPP1* | hsa-miR-3670 | miRWalk |
| *SPP1* | hsa-miR-3675-5p | miRWalk |
| *SPP1* | hsa-miR-3677-5p | miRWalk |
| *SPP1* | hsa-miR-3677-3p | miRWalk |
| *SPP1* | hsa-miR-3678-5p | miRWalk |
| *SPP1* | hsa-miR-3679-5p | miRWalk |
| *SPP1* | hsa-miR-3680-3p | miRWalk |
| *SPP1* | hsa-miR-3681-5p | miRWalk |
| *SPP1* | hsa-miR-3689a-5p | miRWalk |
| *SPP1* | hsa-miR-3689a-3p | miRWalk |
| *SPP1* | hsa-miR-3691-3p | miRWalk |
| *SPP1* | hsa-miR-3692-5p | miRWalk |
| *SPP1* | hsa-miR-3692-3p | miRWalk |
| *SPP1* | hsa-miR-3713 | miRWalk |
| *SPP1* | hsa-miR-3180 | miRWalk |
| *SPP1* | hsa-miR-3907 | miRWalk |
| *SPP1* | hsa-miR-3689b-5p | miRWalk |
| *SPP1* | hsa-miR-3911 | miRWalk |
| *SPP1* | hsa-miR-3912-5p | miRWalk |
| *SPP1* | hsa-miR-3916 | miRWalk |
| *SPP1* | hsa-miR-3917 | miRWalk |
| *SPP1* | hsa-miR-3918 | miRWalk |
| *SPP1* | hsa-miR-3922-5p | miRWalk |
| *SPP1* | hsa-miR-3922-3p | miRWalk |
| *SPP1* | hsa-miR-3924 | miRWalk |
| *SPP1* | hsa-miR-676-5p | miRWalk |
| *SPP1* | hsa-miR-676-3p | miRWalk |
| *SPP1* | hsa-miR-3928-5p | miRWalk |
| *SPP1* | hsa-miR-3928-3p | miRWalk |
| *SPP1* | hsa-miR-3929 | miRWalk |
| *SPP1* | hsa-miR-3934-5p | miRWalk |
| *SPP1* | hsa-miR-3934-3p | miRWalk |
| *SPP1* | hsa-miR-3935 | miRWalk |
| *SPP1* | hsa-miR-3936 | miRWalk |
| *SPP1* | hsa-miR-3937 | miRWalk |
| *SPP1* | hsa-miR-3940-5p | miRWalk |
| *SPP1* | hsa-miR-3942-5p | miRWalk |
| *SPP1* | hsa-miR-3944-5p | miRWalk |
| *SPP1* | hsa-miR-3944-3p | miRWalk |
| *SPP1* | hsa-miR-3945 | miRWalk |
| *SPP1* | hsa-miR-642b-3p | miRWalk |
| *SPP1* | hsa-miR-550b-2-5p | miRWalk |
| *SPP1* | hsa-miR-548z | miRWalk |
| *SPP1* | hsa-miR-548aa | miRWalk |
| *SPP1* | hsa-miR-1268b | miRWalk |
| *SPP1* | hsa-miR-378e | miRWalk |
| *SPP1* | hsa-miR-4418 | miRWalk |
| *SPP1* | hsa-miR-4420 | miRWalk |
| *SPP1* | hsa-miR-4421 | miRWalk |
| *SPP1* | hsa-miR-4422 | miRWalk |
| *SPP1* | hsa-miR-378g | miRWalk |
| *SPP1* | hsa-miR-4425 | miRWalk |
| *SPP1* | hsa-miR-4429 | miRWalk |
| *SPP1* | hsa-miR-4430 | miRWalk |
| *SPP1* | hsa-miR-4433a-3p | miRWalk |
| *SPP1* | hsa-miR-4435 | miRWalk |
| *SPP1* | hsa-miR-4436a | miRWalk |
| *SPP1* | hsa-miR-4437 | miRWalk |
| *SPP1* | hsa-miR-4439 | miRWalk |
| *SPP1* | hsa-miR-4440 | miRWalk |
| *SPP1* | hsa-miR-4443 | miRWalk |
| *SPP1* | hsa-miR-4446-3p | miRWalk |
| *SPP1* | hsa-miR-4447 | miRWalk |
| *SPP1* | hsa-miR-4448 | miRWalk |
| *SPP1* | hsa-miR-548ag | miRWalk |
| *SPP1* | hsa-miR-4453 | miRWalk |
| *SPP1* | hsa-miR-4457 | miRWalk |
| *SPP1* | hsa-miR-4460 | miRWalk |
| *SPP1* | hsa-miR-378h | miRWalk |
| *SPP1* | hsa-miR-3135b | miRWalk |
| *SPP1* | hsa-miR-4462 | miRWalk |
| *SPP1* | hsa-miR-4463 | miRWalk |
| *SPP1* | hsa-miR-548aj-5p | miRWalk |
| *SPP1* | hsa-miR-4466 | miRWalk |
| *SPP1* | hsa-miR-4467 | miRWalk |
| *SPP1* | hsa-miR-4470 | miRWalk |
| *SPP1* | hsa-miR-4472 | miRWalk |
| *SPP1* | hsa-miR-4474-3p | miRWalk |
| *SPP1* | hsa-miR-4476 | miRWalk |
| *SPP1* | hsa-miR-4478 | miRWalk |
| *SPP1* | hsa-miR-3689e | miRWalk |
| *SPP1* | hsa-miR-4479 | miRWalk |
| *SPP1* | hsa-miR-4481 | miRWalk |
| *SPP1* | hsa-miR-4482-5p | miRWalk |
| *SPP1* | hsa-miR-4483 | miRWalk |
| *SPP1* | hsa-miR-4485-3p | miRWalk |
| *SPP1* | hsa-miR-4486 | miRWalk |
| *SPP1* | hsa-miR-4487 | miRWalk |
| *SPP1* | hsa-miR-4488 | miRWalk |
| *SPP1* | hsa-miR-4489 | miRWalk |
| *SPP1* | hsa-miR-4491 | miRWalk |
| *SPP1* | hsa-miR-4492 | miRWalk |
| *SPP1* | hsa-miR-4495 | miRWalk |
| *SPP1* | hsa-miR-4496 | miRWalk |
| *SPP1* | hsa-miR-4497 | miRWalk |
| *SPP1* | hsa-miR-4498 | miRWalk |
| *SPP1* | hsa-miR-4501 | miRWalk |
| *SPP1* | hsa-miR-4502 | miRWalk |
| *SPP1* | hsa-miR-4505 | miRWalk |
| *SPP1* | hsa-miR-4506 | miRWalk |
| *SPP1* | hsa-miR-2392 | miRWalk |
| *SPP1* | hsa-miR-4507 | miRWalk |
| *SPP1* | hsa-miR-4508 | miRWalk |
| *SPP1* | hsa-miR-4510 | miRWalk |
| *SPP1* | hsa-miR-4513 | miRWalk |
| *SPP1* | hsa-miR-4514 | miRWalk |
| *SPP1* | hsa-miR-4515 | miRWalk |
| *SPP1* | hsa-miR-4516 | miRWalk |
| *SPP1* | hsa-miR-4518 | miRWalk |
| *SPP1* | hsa-miR-4521 | miRWalk |
| *SPP1* | hsa-miR-1269b | miRWalk |
| *SPP1* | hsa-miR-4523 | miRWalk |
| *SPP1* | hsa-miR-4525 | miRWalk |
| *SPP1* | hsa-miR-4526 | miRWalk |
| *SPP1* | hsa-miR-4527 | miRWalk |
| *SPP1* | hsa-miR-4528 | miRWalk |
| *SPP1* | hsa-miR-4529-5p | miRWalk |
| *SPP1* | hsa-miR-4529-3p | miRWalk |
| *SPP1* | hsa-miR-4531 | miRWalk |
| *SPP1* | hsa-miR-4533 | miRWalk |
| *SPP1* | hsa-miR-4534 | miRWalk |
| *SPP1* | hsa-miR-378i | miRWalk |
| *SPP1* | hsa-miR-4535 | miRWalk |
| *SPP1* | hsa-miR-1587 | miRWalk |
| *SPP1* | hsa-miR-4538 | miRWalk |
| *SPP1* | hsa-miR-4539 | miRWalk |
| *SPP1* | hsa-miR-4540 | miRWalk |
| *SPP1* | hsa-miR-3960 | miRWalk |
| *SPP1* | hsa-miR-3975 | miRWalk |
| *SPP1* | hsa-miR-3978 | miRWalk |
| *SPP1* | hsa-miR-4632-5p | miRWalk |
| *SPP1* | hsa-miR-4632-3p | miRWalk |
| *SPP1* | hsa-miR-4634 | miRWalk |
| *SPP1* | hsa-miR-4638-5p | miRWalk |
| *SPP1* | hsa-miR-4638-3p | miRWalk |
| *SPP1* | hsa-miR-4639-5p | miRWalk |
| *SPP1* | hsa-miR-4639-3p | miRWalk |
| *SPP1* | hsa-miR-4640-5p | miRWalk |
| *SPP1* | hsa-miR-4640-3p | miRWalk |
| *SPP1* | hsa-miR-4641 | miRWalk |
| *SPP1* | hsa-miR-4646-5p | miRWalk |
| *SPP1* | hsa-miR-4647 | miRWalk |
| *SPP1* | hsa-miR-4648 | miRWalk |
| *SPP1* | hsa-miR-4649-5p | miRWalk |
| *SPP1* | hsa-miR-4649-3p | miRWalk |
| *SPP1* | hsa-miR-4651 | miRWalk |
| *SPP1* | hsa-miR-4653-5p | miRWalk |
| *SPP1* | hsa-miR-4653-3p | miRWalk |
| *SPP1* | hsa-miR-4654 | miRWalk |
| *SPP1* | hsa-miR-4655-5p | miRWalk |
| *SPP1* | hsa-miR-4655-3p | miRWalk |
| *SPP1* | hsa-miR-4656 | miRWalk |
| *SPP1* | hsa-miR-4657 | miRWalk |
| *SPP1* | hsa-miR-4658 | miRWalk |
| *SPP1* | hsa-miR-4659a-3p | miRWalk |
| *SPP1* | hsa-miR-4660 | miRWalk |
| *SPP1* | hsa-miR-4659b-3p | miRWalk |
| *SPP1* | hsa-miR-4663 | miRWalk |
| *SPP1* | hsa-miR-4664-3p | miRWalk |
| *SPP1* | hsa-miR-4665-5p | miRWalk |
| *SPP1* | hsa-miR-4665-3p | miRWalk |
| *SPP1* | hsa-miR-4667-5p | miRWalk |
| *SPP1* | hsa-miR-4667-3p | miRWalk |
| *SPP1* | hsa-miR-219b-3p | miRWalk |
| *SPP1* | hsa-miR-4671-5p | miRWalk |
| *SPP1* | hsa-miR-4671-3p | miRWalk |
| *SPP1* | hsa-miR-4672 | miRWalk |
| *SPP1* | hsa-miR-4673 | miRWalk |
| *SPP1* | hsa-miR-4674 | miRWalk |
| *SPP1* | hsa-miR-4675 | miRWalk |
| *SPP1* | hsa-miR-4676-5p | miRWalk |
| *SPP1* | hsa-miR-4677-5p | miRWalk |
| *SPP1* | hsa-miR-4682 | miRWalk |
| *SPP1* | hsa-miR-4684-3p | miRWalk |
| *SPP1* | hsa-miR-4685-5p | miRWalk |
| *SPP1* | hsa-miR-4685-3p | miRWalk |
| *SPP1* | hsa-miR-4686 | miRWalk |
| *SPP1* | hsa-miR-4687-5p | miRWalk |
| *SPP1* | hsa-miR-4687-3p | miRWalk |
| *SPP1* | hsa-miR-1343-5p | miRWalk |
| *SPP1* | hsa-miR-4688 | miRWalk |
| *SPP1* | hsa-miR-4689 | miRWalk |
| *SPP1* | hsa-miR-4690-5p | miRWalk |
| *SPP1* | hsa-miR-4690-3p | miRWalk |
| *SPP1* | hsa-miR-4691-5p | miRWalk |
| *SPP1* | hsa-miR-4691-3p | miRWalk |
| *SPP1* | hsa-miR-4692 | miRWalk |
| *SPP1* | hsa-miR-4693-5p | miRWalk |
| *SPP1* | hsa-miR-4693-3p | miRWalk |
| *SPP1* | hsa-miR-4695-5p | miRWalk |
| *SPP1* | hsa-miR-4695-3p | miRWalk |
| *SPP1* | hsa-miR-4697-5p | miRWalk |
| *SPP1* | hsa-miR-4699-5p | miRWalk |
| *SPP1* | hsa-miR-4699-3p | miRWalk |
| *SPP1* | hsa-miR-4700-5p | miRWalk |
| *SPP1* | hsa-miR-4701-5p | miRWalk |
| *SPP1* | hsa-miR-4701-3p | miRWalk |
| *SPP1* | hsa-miR-4705 | miRWalk |
| *SPP1* | hsa-miR-4706 | miRWalk |
| *SPP1* | hsa-miR-4707-5p | miRWalk |
| *SPP1* | hsa-miR-4708-5p | miRWalk |
| *SPP1* | hsa-miR-4709-3p | miRWalk |
| *SPP1* | hsa-miR-4710 | miRWalk |
| *SPP1* | hsa-miR-4711-3p | miRWalk |
| *SPP1* | hsa-miR-4713-3p | miRWalk |
| *SPP1* | hsa-miR-4714-5p | miRWalk |
| *SPP1* | hsa-miR-4714-3p | miRWalk |
| *SPP1* | hsa-miR-4715-5p | miRWalk |
| *SPP1* | hsa-miR-4716-3p | miRWalk |
| *SPP1* | hsa-miR-3529-5p | miRWalk |
| *SPP1* | hsa-miR-4717-5p | miRWalk |
| *SPP1* | hsa-miR-4722-5p | miRWalk |
| *SPP1* | hsa-miR-4722-3p | miRWalk |
| *SPP1* | hsa-miR-4723-5p | miRWalk |
| *SPP1* | hsa-miR-4723-3p | miRWalk |
| *SPP1* | hsa-miR-451b | miRWalk |
| *SPP1* | hsa-miR-4724-3p | miRWalk |
| *SPP1* | hsa-miR-4725-5p | miRWalk |
| *SPP1* | hsa-miR-4725-3p | miRWalk |
| *SPP1* | hsa-miR-4726-5p | miRWalk |
| *SPP1* | hsa-miR-4726-3p | miRWalk |
| *SPP1* | hsa-miR-4727-5p | miRWalk |
| *SPP1* | hsa-miR-4727-3p | miRWalk |
| *SPP1* | hsa-miR-4728-3p | miRWalk |
| *SPP1* | hsa-miR-4731-5p | miRWalk |
| *SPP1* | hsa-miR-4732-5p | miRWalk |
| *SPP1* | hsa-miR-4732-3p | miRWalk |
| *SPP1* | hsa-miR-4733-3p | miRWalk |
| *SPP1* | hsa-miR-4734 | miRWalk |
| *SPP1* | hsa-miR-4736 | miRWalk |
| *SPP1* | hsa-miR-3064-5p | miRWalk |
| *SPP1* | hsa-miR-3064-3p | miRWalk |
| *SPP1* | hsa-miR-4738-5p | miRWalk |
| *SPP1* | hsa-miR-4738-3p | miRWalk |
| *SPP1* | hsa-miR-4739 | miRWalk |
| *SPP1* | hsa-miR-4740-3p | miRWalk |
| *SPP1* | hsa-miR-4741 | miRWalk |
| *SPP1* | hsa-miR-4742-3p | miRWalk |
| *SPP1* | hsa-miR-4743-5p | miRWalk |
| *SPP1* | hsa-miR-4743-3p | miRWalk |
| *SPP1* | hsa-miR-4745-5p | miRWalk |
| *SPP1* | hsa-miR-4745-3p | miRWalk |
| *SPP1* | hsa-miR-4746-5p | miRWalk |
| *SPP1* | hsa-miR-4746-3p | miRWalk |
| *SPP1* | hsa-miR-4747-5p | miRWalk |
| *SPP1* | hsa-miR-4747-3p | miRWalk |
| *SPP1* | hsa-miR-4748 | miRWalk |
| *SPP1* | hsa-miR-4749-5p | miRWalk |
| *SPP1* | hsa-miR-4749-3p | miRWalk |
| *SPP1* | hsa-miR-4750-5p | miRWalk |
| *SPP1* | hsa-miR-4752 | miRWalk |
| *SPP1* | hsa-miR-4754 | miRWalk |
| *SPP1* | hsa-miR-499b-5p | miRWalk |
| *SPP1* | hsa-miR-499b-3p | miRWalk |
| *SPP1* | hsa-miR-4756-5p | miRWalk |
| *SPP1* | hsa-miR-4756-3p | miRWalk |
| *SPP1* | hsa-miR-4757-5p | miRWalk |
| *SPP1* | hsa-miR-4757-3p | miRWalk |
| *SPP1* | hsa-miR-4758-5p | miRWalk |
| *SPP1* | hsa-miR-4758-3p | miRWalk |
| *SPP1* | hsa-miR-4761-5p | miRWalk |
| *SPP1* | hsa-miR-4761-3p | miRWalk |
| *SPP1* | hsa-miR-4763-5p | miRWalk |
| *SPP1* | hsa-miR-4763-3p | miRWalk |
| *SPP1* | hsa-miR-4764-3p | miRWalk |
| *SPP1* | hsa-miR-4765 | miRWalk |
| *SPP1* | hsa-miR-4767 | miRWalk |
| *SPP1* | hsa-miR-4768-3p | miRWalk |
| *SPP1* | hsa-miR-4769-5p | miRWalk |
| *SPP1* | hsa-miR-4769-3p | miRWalk |
| *SPP1* | hsa-miR-4772-3p | miRWalk |
| *SPP1* | hsa-miR-4774-5p | miRWalk |
| *SPP1* | hsa-miR-4776-5p | miRWalk |
| *SPP1* | hsa-miR-4776-3p | miRWalk |
| *SPP1* | hsa-miR-4778-3p | miRWalk |
| *SPP1* | hsa-miR-4779 | miRWalk |
| *SPP1* | hsa-miR-4436b-3p | miRWalk |
| *SPP1* | hsa-miR-4781-5p | miRWalk |
| *SPP1* | hsa-miR-4783-5p | miRWalk |
| *SPP1* | hsa-miR-4783-3p | miRWalk |
| *SPP1* | hsa-miR-4784 | miRWalk |
| *SPP1* | hsa-miR-4785 | miRWalk |
| *SPP1* | hsa-miR-2467-5p | miRWalk |
| *SPP1* | hsa-miR-4786-5p | miRWalk |
| *SPP1* | hsa-miR-4786-3p | miRWalk |
| *SPP1* | hsa-miR-4787-5p | miRWalk |
| *SPP1* | hsa-miR-4790-3p | miRWalk |
| *SPP1* | hsa-miR-4793-5p | miRWalk |
| *SPP1* | hsa-miR-4793-3p | miRWalk |
| *SPP1* | hsa-miR-4796-5p | miRWalk |
| *SPP1* | hsa-miR-4800-3p | miRWalk |
| *SPP1* | hsa-miR-4802-3p | miRWalk |
| *SPP1* | hsa-miR-4999-5p | miRWalk |
| *SPP1* | hsa-miR-5001-5p | miRWalk |
| *SPP1* | hsa-miR-5001-3p | miRWalk |
| *SPP1* | hsa-miR-5004-5p | miRWalk |
| *SPP1* | hsa-miR-5004-3p | miRWalk |
| *SPP1* | hsa-miR-5006-5p | miRWalk |
| *SPP1* | hsa-miR-5006-3p | miRWalk |
| *SPP1* | hsa-miR-5007-5p | miRWalk |
| *SPP1* | hsa-miR-5008-5p | miRWalk |
| *SPP1* | hsa-miR-5008-3p | miRWalk |
| *SPP1* | hsa-miR-5009-5p | miRWalk |
| *SPP1* | hsa-miR-5010-5p | miRWalk |
| *SPP1* | hsa-miR-5047 | miRWalk |
| *SPP1* | hsa-miR-5087 | miRWalk |
| *SPP1* | hsa-miR-5088-5p | miRWalk |
| *SPP1* | hsa-miR-5089-3p | miRWalk |
| *SPP1* | hsa-miR-5090 | miRWalk |
| *SPP1* | hsa-miR-5092 | miRWalk |
| *SPP1* | hsa-miR-5093 | miRWalk |
| *SPP1* | hsa-miR-5186 | miRWalk |
| *SPP1* | hsa-miR-5189-5p | miRWalk |
| *SPP1* | hsa-miR-5190 | miRWalk |
| *SPP1* | hsa-miR-5192 | miRWalk |
| *SPP1* | hsa-miR-5194 | miRWalk |
| *SPP1* | hsa-miR-5195-3p | miRWalk |
| *SPP1* | hsa-miR-5196-5p | miRWalk |
| *SPP1* | hsa-miR-5197-3p | miRWalk |
| *SPP1* | hsa-miR-5571-3p | miRWalk |
| *SPP1* | hsa-miR-5572 | miRWalk |
| *SPP1* | hsa-miR-664b-5p | miRWalk |
| *SPP1* | hsa-miR-5580-5p | miRWalk |
| *SPP1* | hsa-miR-548at-5p | miRWalk |
| *SPP1* | hsa-miR-5584-3p | miRWalk |
| *SPP1* | hsa-miR-1295b-3p | miRWalk |
| *SPP1* | hsa-miR-5588-5p | miRWalk |
| *SPP1* | hsa-miR-5589-5p | miRWalk |
| *SPP1* | hsa-miR-5680 | miRWalk |
| *SPP1* | hsa-miR-5685 | miRWalk |
| *SPP1* | hsa-miR-5691 | miRWalk |
| *SPP1* | hsa-miR-5692a | miRWalk |
| *SPP1* | hsa-miR-5693 | miRWalk |
| *SPP1* | hsa-miR-5694 | miRWalk |
| *SPP1* | hsa-miR-5696 | miRWalk |
| *SPP1* | hsa-miR-5698 | miRWalk |
| *SPP1* | hsa-miR-5699-3p | miRWalk |
| *SPP1* | hsa-miR-5705 | miRWalk |
| *SPP1* | hsa-miR-5707 | miRWalk |
| *SPP1* | hsa-miR-5708 | miRWalk |
| *SPP1* | hsa-miR-5787 | miRWalk |
| *SPP1* | hsa-miR-1199-5p | miRWalk |
| *SPP1* | hsa-miR-6068 | miRWalk |
| *SPP1* | hsa-miR-6069 | miRWalk |
| *SPP1* | hsa-miR-6072 | miRWalk |
| *SPP1* | hsa-miR-6073 | miRWalk |
| *SPP1* | hsa-miR-6075 | miRWalk |
| *SPP1* | hsa-miR-6077 | miRWalk |
| *SPP1* | hsa-miR-6078 | miRWalk |
| *SPP1* | hsa-miR-6083 | miRWalk |
| *SPP1* | hsa-miR-6084 | miRWalk |
| *SPP1* | hsa-miR-6085 | miRWalk |
| *SPP1* | hsa-miR-6086 | miRWalk |
| *SPP1* | hsa-miR-6088 | miRWalk |
| *SPP1* | hsa-miR-6089 | miRWalk |
| *SPP1* | hsa-miR-6090 | miRWalk |
| *SPP1* | hsa-miR-6127 | miRWalk |
| *SPP1* | hsa-miR-6129 | miRWalk |
| *SPP1* | hsa-miR-6131 | miRWalk |
| *SPP1* | hsa-miR-6132 | miRWalk |
| *SPP1* | hsa-miR-6133 | miRWalk |
| *SPP1* | hsa-miR-6165 | miRWalk |
| *SPP1* | hsa-miR-6499-5p | miRWalk |
| *SPP1* | hsa-miR-548az-3p | miRWalk |
| *SPP1* | hsa-miR-6501-5p | miRWalk |
| *SPP1* | hsa-miR-6501-3p | miRWalk |
| *SPP1* | hsa-miR-6503-3p | miRWalk |
| *SPP1* | hsa-miR-6504-5p | miRWalk |
| *SPP1* | hsa-miR-6506-5p | miRWalk |
| *SPP1* | hsa-miR-6507-3p | miRWalk |
| *SPP1* | hsa-miR-6508-3p | miRWalk |
| *SPP1* | hsa-miR-6510-5p | miRWalk |
| *SPP1* | hsa-miR-6511a-5p | miRWalk |
| *SPP1* | hsa-miR-6511a-3p | miRWalk |
| *SPP1* | hsa-miR-6513-5p | miRWalk |
| *SPP1* | hsa-miR-6513-3p | miRWalk |
| *SPP1* | hsa-miR-6514-5p | miRWalk |
| *SPP1* | hsa-miR-6515-5p | miRWalk |
| *SPP1* | hsa-miR-6715b-5p | miRWalk |
| *SPP1* | hsa-miR-6715b-3p | miRWalk |
| *SPP1* | hsa-miR-6716-5p | miRWalk |
| *SPP1* | hsa-miR-6717-5p | miRWalk |
| *SPP1* | hsa-miR-6511b-5p | miRWalk |
| *SPP1* | hsa-miR-6511b-3p | miRWalk |
| *SPP1* | hsa-miR-6718-5p | miRWalk |
| *SPP1* | hsa-miR-6720-5p | miRWalk |
| *SPP1* | hsa-miR-6720-3p | miRWalk |
| *SPP1* | hsa-miR-6721-5p | miRWalk |
| *SPP1* | hsa-miR-6722-3p | miRWalk |
| *SPP1* | hsa-miR-6724-5p | miRWalk |
| *SPP1* | hsa-miR-892c-5p | miRWalk |
| *SPP1* | hsa-miR-6726-5p | miRWalk |
| *SPP1* | hsa-miR-6726-3p | miRWalk |
| *SPP1* | hsa-miR-6727-5p | miRWalk |
| *SPP1* | hsa-miR-6727-3p | miRWalk |
| *SPP1* | hsa-miR-6728-5p | miRWalk |
| *SPP1* | hsa-miR-6728-3p | miRWalk |
| *SPP1* | hsa-miR-6729-5p | miRWalk |
| *SPP1* | hsa-miR-6730-5p | miRWalk |
| *SPP1* | hsa-miR-6730-3p | miRWalk |
| *SPP1* | hsa-miR-6732-5p | miRWalk |
| *SPP1* | hsa-miR-6732-3p | miRWalk |
| *SPP1* | hsa-miR-6734-5p | miRWalk |
| *SPP1* | hsa-miR-6735-5p | miRWalk |
| *SPP1* | hsa-miR-6736-5p | miRWalk |
| *SPP1* | hsa-miR-6736-3p | miRWalk |
| *SPP1* | hsa-miR-6737-5p | miRWalk |
| *SPP1* | hsa-miR-6737-3p | miRWalk |
| *SPP1* | hsa-miR-6739-3p | miRWalk |
| *SPP1* | hsa-miR-6740-5p | miRWalk |
| *SPP1* | hsa-miR-6741-5p | miRWalk |
| *SPP1* | hsa-miR-6742-5p | miRWalk |
| *SPP1* | hsa-miR-6742-3p | miRWalk |
| *SPP1* | hsa-miR-6743-5p | miRWalk |
| *SPP1* | hsa-miR-6744-3p | miRWalk |
| *SPP1* | hsa-miR-6745 | miRWalk |
| *SPP1* | hsa-miR-6746-5p | miRWalk |
| *SPP1* | hsa-miR-6746-3p | miRWalk |
| *SPP1* | hsa-miR-6747-5p | miRWalk |
| *SPP1* | hsa-miR-6747-3p | miRWalk |
| *SPP1* | hsa-miR-6748-5p | miRWalk |
| *SPP1* | hsa-miR-6748-3p | miRWalk |
| *SPP1* | hsa-miR-6749-5p | miRWalk |
| *SPP1* | hsa-miR-6749-3p | miRWalk |
| *SPP1* | hsa-miR-6750-5p | miRWalk |
| *SPP1* | hsa-miR-6751-5p | miRWalk |
| *SPP1* | hsa-miR-6752-5p | miRWalk |
| *SPP1* | hsa-miR-6753-5p | miRWalk |
| *SPP1* | hsa-miR-6753-3p | miRWalk |
| *SPP1* | hsa-miR-6754-5p | miRWalk |
| *SPP1* | hsa-miR-6754-3p | miRWalk |
| *SPP1* | hsa-miR-6756-5p | miRWalk |
| *SPP1* | hsa-miR-6756-3p | miRWalk |
| *SPP1* | hsa-miR-6757-5p | miRWalk |
| *SPP1* | hsa-miR-6759-5p | miRWalk |
| *SPP1* | hsa-miR-6760-3p | miRWalk |
| *SPP1* | hsa-miR-6761-5p | miRWalk |
| *SPP1* | hsa-miR-6761-3p | miRWalk |
| *SPP1* | hsa-miR-6762-5p | miRWalk |
| *SPP1* | hsa-miR-6762-3p | miRWalk |
| *SPP1* | hsa-miR-6763-5p | miRWalk |
| *SPP1* | hsa-miR-6764-5p | miRWalk |
| *SPP1* | hsa-miR-6764-3p | miRWalk |
| *SPP1* | hsa-miR-6765-5p | miRWalk |
| *SPP1* | hsa-miR-6765-3p | miRWalk |
| *SPP1* | hsa-miR-6766-5p | miRWalk |
| *SPP1* | hsa-miR-6766-3p | miRWalk |
| *SPP1* | hsa-miR-6767-5p | miRWalk |
| *SPP1* | hsa-miR-6768-3p | miRWalk |
| *SPP1* | hsa-miR-6769a-5p | miRWalk |
| *SPP1* | hsa-miR-6769a-3p | miRWalk |
| *SPP1* | hsa-miR-6770-5p | miRWalk |
| *SPP1* | hsa-miR-6770-3p | miRWalk |
| *SPP1* | hsa-miR-6771-5p | miRWalk |
| *SPP1* | hsa-miR-6772-5p | miRWalk |
| *SPP1* | hsa-miR-6772-3p | miRWalk |
| *SPP1* | hsa-miR-6773-3p | miRWalk |
| *SPP1* | hsa-miR-6774-5p | miRWalk |
| *SPP1* | hsa-miR-6774-3p | miRWalk |
| *SPP1* | hsa-miR-6775-5p | miRWalk |
| *SPP1* | hsa-miR-6776-5p | miRWalk |
| *SPP1* | hsa-miR-6776-3p | miRWalk |
| *SPP1* | hsa-miR-6778-5p | miRWalk |
| *SPP1* | hsa-miR-6779-5p | miRWalk |
| *SPP1* | hsa-miR-6779-3p | miRWalk |
| *SPP1* | hsa-miR-6780a-5p | miRWalk |
| *SPP1* | hsa-miR-6780a-3p | miRWalk |
| *SPP1* | hsa-miR-6781-5p | miRWalk |
| *SPP1* | hsa-miR-6781-3p | miRWalk |
| *SPP1* | hsa-miR-6782-5p | miRWalk |
| *SPP1* | hsa-miR-6782-3p | miRWalk |
| *SPP1* | hsa-miR-6784-5p | miRWalk |
| *SPP1* | hsa-miR-6785-5p | miRWalk |
| *SPP1* | hsa-miR-6786-5p | miRWalk |
| *SPP1* | hsa-miR-6786-3p | miRWalk |
| *SPP1* | hsa-miR-6787-5p | miRWalk |
| *SPP1* | hsa-miR-6787-3p | miRWalk |
| *SPP1* | hsa-miR-6788-5p | miRWalk |
| *SPP1* | hsa-miR-6789-5p | miRWalk |
| *SPP1* | hsa-miR-6789-3p | miRWalk |
| *SPP1* | hsa-miR-6790-5p | miRWalk |
| *SPP1* | hsa-miR-6790-3p | miRWalk |
| *SPP1* | hsa-miR-6791-5p | miRWalk |
| *SPP1* | hsa-miR-6791-3p | miRWalk |
| *SPP1* | hsa-miR-6792-5p | miRWalk |
| *SPP1* | hsa-miR-6793-5p | miRWalk |
| *SPP1* | hsa-miR-6794-5p | miRWalk |
| *SPP1* | hsa-miR-6794-3p | miRWalk |
| *SPP1* | hsa-miR-6795-5p | miRWalk |
| *SPP1* | hsa-miR-6796-5p | miRWalk |
| *SPP1* | hsa-miR-6796-3p | miRWalk |
| *SPP1* | hsa-miR-6797-5p | miRWalk |
| *SPP1* | hsa-miR-6797-3p | miRWalk |
| *SPP1* | hsa-miR-6798-5p | miRWalk |
| *SPP1* | hsa-miR-6799-5p | miRWalk |
| *SPP1* | hsa-miR-6800-5p | miRWalk |
| *SPP1* | hsa-miR-6801-5p | miRWalk |
| *SPP1* | hsa-miR-6802-5p | miRWalk |
| *SPP1* | hsa-miR-6803-5p | miRWalk |
| *SPP1* | hsa-miR-6805-5p | miRWalk |
| *SPP1* | hsa-miR-6805-3p | miRWalk |
| *SPP1* | hsa-miR-6806-5p | miRWalk |
| *SPP1* | hsa-miR-6808-5p | miRWalk |
| *SPP1* | hsa-miR-6810-5p | miRWalk |
| *SPP1* | hsa-miR-6811-3p | miRWalk |
| *SPP1* | hsa-miR-6812-5p | miRWalk |
| *SPP1* | hsa-miR-6813-5p | miRWalk |
| *SPP1* | hsa-miR-6813-3p | miRWalk |
| *SPP1* | hsa-miR-6814-3p | miRWalk |
| *SPP1* | hsa-miR-6815-5p | miRWalk |
| *SPP1* | hsa-miR-6816-5p | miRWalk |
| *SPP1* | hsa-miR-6817-5p | miRWalk |
| *SPP1* | hsa-miR-6820-5p | miRWalk |
| *SPP1* | hsa-miR-6820-3p | miRWalk |
| *SPP1* | hsa-miR-6821-5p | miRWalk |
| *SPP1* | hsa-miR-6821-3p | miRWalk |
| *SPP1* | hsa-miR-6822-5p | miRWalk |
| *SPP1* | hsa-miR-6822-3p | miRWalk |
| *SPP1* | hsa-miR-6823-5p | miRWalk |
| *SPP1* | hsa-miR-6824-5p | miRWalk |
| *SPP1* | hsa-miR-6825-5p | miRWalk |
| *SPP1* | hsa-miR-6825-3p | miRWalk |
| *SPP1* | hsa-miR-6826-3p | miRWalk |
| *SPP1* | hsa-miR-6827-5p | miRWalk |
| *SPP1* | hsa-miR-6829-5p | miRWalk |
| *SPP1* | hsa-miR-6829-3p | miRWalk |
| *SPP1* | hsa-miR-6830-5p | miRWalk |
| *SPP1* | hsa-miR-6830-3p | miRWalk |
| *SPP1* | hsa-miR-6831-5p | miRWalk |
| *SPP1* | hsa-miR-6834-5p | miRWalk |
| *SPP1* | hsa-miR-6835-5p | miRWalk |
| *SPP1* | hsa-miR-6780b-5p | miRWalk |
| *SPP1* | hsa-miR-6836-5p | miRWalk |
| *SPP1* | hsa-miR-6836-3p | miRWalk |
| *SPP1* | hsa-miR-6837-5p | miRWalk |
| *SPP1* | hsa-miR-6838-5p | miRWalk |
| *SPP1* | hsa-miR-6838-3p | miRWalk |
| *SPP1* | hsa-miR-6839-3p | miRWalk |
| *SPP1* | hsa-miR-6841-3p | miRWalk |
| *SPP1* | hsa-miR-6842-5p | miRWalk |
| *SPP1* | hsa-miR-6842-3p | miRWalk |
| *SPP1* | hsa-miR-6845-5p | miRWalk |
| *SPP1* | hsa-miR-6846-5p | miRWalk |
| *SPP1* | hsa-miR-6847-5p | miRWalk |
| *SPP1* | hsa-miR-6847-3p | miRWalk |
| *SPP1* | hsa-miR-6848-5p | miRWalk |
| *SPP1* | hsa-miR-6848-3p | miRWalk |
| *SPP1* | hsa-miR-6850-3p | miRWalk |
| *SPP1* | hsa-miR-6851-5p | miRWalk |
| *SPP1* | hsa-miR-6852-5p | miRWalk |
| *SPP1* | hsa-miR-6853-5p | miRWalk |
| *SPP1* | hsa-miR-6854-5p | miRWalk |
| *SPP1* | hsa-miR-6854-3p | miRWalk |
| *SPP1* | hsa-miR-6855-5p | miRWalk |
| *SPP1* | hsa-miR-6855-3p | miRWalk |
| *SPP1* | hsa-miR-6856-5p | miRWalk |
| *SPP1* | hsa-miR-6857-5p | miRWalk |
| *SPP1* | hsa-miR-6858-5p | miRWalk |
| *SPP1* | hsa-miR-6858-3p | miRWalk |
| *SPP1* | hsa-miR-6859-5p | miRWalk |
| *SPP1* | hsa-miR-6859-3p | miRWalk |
| *SPP1* | hsa-miR-6769b-5p | miRWalk |
| *SPP1* | hsa-miR-6769b-3p | miRWalk |
| *SPP1* | hsa-miR-6860 | miRWalk |
| *SPP1* | hsa-miR-6861-5p | miRWalk |
| *SPP1* | hsa-miR-6862-5p | miRWalk |
| *SPP1* | hsa-miR-6862-3p | miRWalk |
| *SPP1* | hsa-miR-6863 | miRWalk |
| *SPP1* | hsa-miR-6864-3p | miRWalk |
| *SPP1* | hsa-miR-6865-5p | miRWalk |
| *SPP1* | hsa-miR-6866-5p | miRWalk |
| *SPP1* | hsa-miR-6868-5p | miRWalk |
| *SPP1* | hsa-miR-6868-3p | miRWalk |
| *SPP1* | hsa-miR-6869-5p | miRWalk |
| *SPP1* | hsa-miR-6869-3p | miRWalk |
| *SPP1* | hsa-miR-6870-5p | miRWalk |
| *SPP1* | hsa-miR-6871-5p | miRWalk |
| *SPP1* | hsa-miR-6872-5p | miRWalk |
| *SPP1* | hsa-miR-6872-3p | miRWalk |
| *SPP1* | hsa-miR-6873-3p | miRWalk |
| *SPP1* | hsa-miR-6874-3p | miRWalk |
| *SPP1* | hsa-miR-6876-3p | miRWalk |
| *SPP1* | hsa-miR-6877-5p | miRWalk |
| *SPP1* | hsa-miR-6877-3p | miRWalk |
| *SPP1* | hsa-miR-6878-5p | miRWalk |
| *SPP1* | hsa-miR-6878-3p | miRWalk |
| *SPP1* | hsa-miR-6879-5p | miRWalk |
| *SPP1* | hsa-miR-6879-3p | miRWalk |
| *SPP1* | hsa-miR-6880-5p | miRWalk |
| *SPP1* | hsa-miR-6881-5p | miRWalk |
| *SPP1* | hsa-miR-6881-3p | miRWalk |
| *SPP1* | hsa-miR-6882-3p | miRWalk |
| *SPP1* | hsa-miR-6883-5p | miRWalk |
| *SPP1* | hsa-miR-6884-5p | miRWalk |
| *SPP1* | hsa-miR-6884-3p | miRWalk |
| *SPP1* | hsa-miR-6885-5p | miRWalk |
| *SPP1* | hsa-miR-6885-3p | miRWalk |
| *SPP1* | hsa-miR-6886-5p | miRWalk |
| *SPP1* | hsa-miR-6886-3p | miRWalk |
| *SPP1* | hsa-miR-6887-5p | miRWalk |
| *SPP1* | hsa-miR-6889-5p | miRWalk |
| *SPP1* | hsa-miR-6890-5p | miRWalk |
| *SPP1* | hsa-miR-6891-5p | miRWalk |
| *SPP1* | hsa-miR-6893-5p | miRWalk |
| *SPP1* | hsa-miR-6894-5p | miRWalk |
| *SPP1* | hsa-miR-6894-3p | miRWalk |
| *SPP1* | hsa-miR-6895-5p | miRWalk |
| *SPP1* | hsa-miR-6895-3p | miRWalk |
| *SPP1* | hsa-miR-7106-5p | miRWalk |
| *SPP1* | hsa-miR-7106-3p | miRWalk |
| *SPP1* | hsa-miR-7107-5p | miRWalk |
| *SPP1* | hsa-miR-7107-3p | miRWalk |
| *SPP1* | hsa-miR-7108-5p | miRWalk |
| *SPP1* | hsa-miR-7109-5p | miRWalk |
| *SPP1* | hsa-miR-7109-3p | miRWalk |
| *SPP1* | hsa-miR-7110-5p | miRWalk |
| *SPP1* | hsa-miR-7111-5p | miRWalk |
| *SPP1* | hsa-miR-7112-5p | miRWalk |
| *SPP1* | hsa-miR-7112-3p | miRWalk |
| *SPP1* | hsa-miR-7114-5p | miRWalk |
| *SPP1* | hsa-miR-7150 | miRWalk |
| *SPP1* | hsa-miR-7151-3p | miRWalk |
| *SPP1* | hsa-miR-7152-3p | miRWalk |
| *SPP1* | hsa-miR-7154-3p | miRWalk |
| *SPP1* | hsa-miR-7155-5p | miRWalk |
| *SPP1* | hsa-miR-7156-5p | miRWalk |
| *SPP1* | hsa-miR-7156-3p | miRWalk |
| *SPP1* | hsa-miR-7157-5p | miRWalk |
| *SPP1* | hsa-miR-7157-3p | miRWalk |
| *SPP1* | hsa-miR-7158-5p | miRWalk |
| *SPP1* | hsa-miR-7158-3p | miRWalk |
| *SPP1* | hsa-miR-7161-5p | miRWalk |
| *SPP1* | hsa-miR-7160-5p | miRWalk |
| *SPP1* | hsa-miR-7162-5p | miRWalk |
| *SPP1* | hsa-miR-7162-3p | miRWalk |
| *SPP1* | hsa-miR-7515 | miRWalk |
| *SPP1* | hsa-miR-7703 | miRWalk |
| *SPP1* | hsa-miR-7704 | miRWalk |
| *SPP1* | hsa-miR-7706 | miRWalk |
| *SPP1* | hsa-miR-4433b-3p | miRWalk |
| *SPP1* | hsa-miR-1273h-5p | miRWalk |
| *SPP1* | hsa-miR-7844-5p | miRWalk |
| *SPP1* | hsa-miR-7845-5p | miRWalk |
| *SPP1* | hsa-miR-7846-3p | miRWalk |
| *SPP1* | hsa-miR-7847-3p | miRWalk |
| *SPP1* | hsa-miR-7848-3p | miRWalk |
| *SPP1* | hsa-miR-7850-5p | miRWalk |
| *SPP1* | hsa-miR-7851-3p | miRWalk |
| *SPP1* | hsa-miR-7854-3p | miRWalk |
| *SPP1* | hsa-miR-7856-5p | miRWalk |
| *SPP1* | hsa-miR-7974 | miRWalk |
| *SPP1* | hsa-miR-7978 | miRWalk |
| *SPP1* | hsa-miR-8052 | miRWalk |
| *SPP1* | hsa-miR-8053 | miRWalk |
| *SPP1* | hsa-miR-8060 | miRWalk |
| *SPP1* | hsa-miR-8068 | miRWalk |
| *SPP1* | hsa-miR-8069 | miRWalk |
| *SPP1* | hsa-miR-8070 | miRWalk |
| *SPP1* | hsa-miR-8072 | miRWalk |
| *SPP1* | hsa-miR-8073 | miRWalk |
| *SPP1* | hsa-miR-8075 | miRWalk |
| *SPP1* | hsa-miR-8077 | miRWalk |
| *SPP1* | hsa-miR-8078 | miRWalk |
| *SPP1* | hsa-miR-8080 | miRWalk |
| *SPP1* | hsa-miR-8082 | miRWalk |
| *SPP1* | hsa-miR-8083 | miRWalk |
| *SPP1* | hsa-miR-8085 | miRWalk |
| *SPP1* | hsa-miR-8086 | miRWalk |
| *SPP1* | hsa-miR-8087 | miRWalk |
| *SPP1* | hsa-miR-8088 | miRWalk |
| *SPP1* | hsa-miR-8089 | miRWalk |
| *SPP1* | hsa-miR-9500 | miRWalk |
| *SPP1* | hsa-miR-9718 | miRWalk |
| *SPP1* | hsa-miR-9899 | miRWalk |
| *SPP1* | hsa-miR-9901 | miRWalk |
| *SPP1* | hsa-miR-9902 | miRWalk |
| *SPP1* | hsa-miR-9903 | miRWalk |
| *SPP1* | hsa-miR-1843 | miRWalk |
| *SPP1* | hsa-miR-10392-5p | miRWalk |
| *SPP1* | hsa-miR-10392-3p | miRWalk |
| *SPP1* | hsa-miR-10394-5p | miRWalk |
| *SPP1* | hsa-miR-10394-3p | miRWalk |
| *SPP1* | hsa-miR-10396a-5p | miRWalk |
| *SPP1* | hsa-miR-10397-5p | miRWalk |
| *SPP1* | hsa-miR-10397-3p | miRWalk |
| *SPP1* | hsa-miR-10398-3p | miRWalk |
| *SPP1* | hsa-miR-10400-5p | miRWalk |
| *SPP1* | hsa-miR-10400-3p | miRWalk |
| *SPP1* | hsa-miR-10401-5p | miRWalk |
| *SPP1* | hsa-miR-10401-3p | miRWalk |
| *SPP1* | hsa-miR-10526-3p | miRWalk |
| *SPP1* | hsa-miR-11181-3p | miRWalk |
| *SPP1* | hsa-miR-11399 | miRWalk |
| *SPP1* | hsa-miR-11400 | miRWalk |
| *SPP1* | hsa-miR-11401 | miRWalk |
| *SPP1* | hsa-miR-3059-5p | miRWalk |
| *SPP1* | hsa-miR-3059-3p | miRWalk |
| *SPP1* | hsa-miR-3085-5p | miRWalk |
| *SPP1* | hsa-miR-3085-3p | miRWalk |
| *SPP1* | hsa-miR-6529-5p | miRWalk |
| *SPP1* | hsa-miR-9851-5p | miRWalk |
| *SPP1* | hsa-miR-9851-3p | miRWalk |
| *SPP1* | hsa-miR-12114 | miRWalk |
| *SPP1* | hsa-miR-12115 | miRWalk |
| *SPP1* | hsa-miR-12116 | miRWalk |
| *SPP1* | hsa-miR-12117 | miRWalk |
| *SPP1* | hsa-miR-12118 | miRWalk |
| *SPP1* | hsa-miR-12119 | miRWalk |
| *SPP1* | hsa-miR-12120 | miRWalk |
| *SPP1* | hsa-miR-12121 | miRWalk |
| *SPP1* | hsa-miR-12125 | miRWalk |
| *SPP1* | hsa-miR-12128 | miRWalk |
| *SPP1* | hsa-miR-12129 | miRWalk |
| *SPP1* | hsa-miR-12133 | miRWalk |
| *SPP1* | hsa-miR-16-1-3p | miRWalk |
| *SPP1* | hsa-miR-101-3p | miRWalk |
| *SPP1* | hsa-miR-103a-3p | miRWalk |
| *SPP1* | hsa-miR-199a-5p | miRWalk |
| *SPP1* | hsa-miR-205-5p | miRWalk |
| *SPP1* | hsa-miR-224-5p | miRWalk |
| *SPP1* | hsa-miR-15b-5p | miRWalk |
| *SPP1* | hsa-miR-9-5p | miRWalk |
| *SPP1* | hsa-miR-127-5p | miRWalk |
| *SPP1* | hsa-miR-138-1-3p | miRWalk |
| *SPP1* | hsa-miR-128-2-5p | miRWalk |
| *SPP1* | hsa-miR-378a-3p | miRWalk |
| *SPP1* | hsa-miR-342-5p | miRWalk |
| *SPP1* | hsa-miR-135b-3p | miRWalk |
| *SPP1* | hsa-miR-148b-5p | miRWalk |
| *SPP1* | hsa-miR-324-5p | miRWalk |
| *SPP1* | hsa-miR-489-5p | miRWalk |
| *SPP1* | hsa-miR-432-3p | miRWalk |
| *SPP1* | hsa-miR-497-5p | miRWalk |
| *SPP1* | hsa-miR-512-3p | miRWalk |
| *SPP1* | hsa-miR-520g-5p | miRWalk |
| *SPP1* | hsa-miR-516a-5p | miRWalk |
| *SPP1* | hsa-miR-450a-2-3p | miRWalk |
| *SPP1* | hsa-miR-505-5p | miRWalk |
| *SPP1* | hsa-miR-532-5p | miRWalk |
| *SPP1* | hsa-miR-614 | miRWalk |
| *SPP1* | hsa-miR-630 | miRWalk |
| *SPP1* | hsa-miR-1301-5p | miRWalk |
| *SPP1* | hsa-miR-766-3p | miRWalk |
| *SPP1* | hsa-miR-890 | miRWalk |
| *SPP1* | hsa-miR-1181 | miRWalk |
| *SPP1* | hsa-miR-1236-5p | miRWalk |
| *SPP1* | hsa-miR-1236-3p | miRWalk |
| *SPP1* | hsa-miR-1202 | miRWalk |
| *SPP1* | hsa-miR-1304-5p | miRWalk |
| *SPP1* | hsa-miR-1247-3p | miRWalk |
| *SPP1* | hsa-miR-548p | miRWalk |
| *SPP1* | hsa-miR-320d | miRWalk |
| *SPP1* | hsa-miR-1912-3p | miRWalk |
| *SPP1* | hsa-miR-1915-3p | miRWalk |
| *SPP1* | hsa-miR-1972 | miRWalk |
| *SPP1* | hsa-miR-2115-5p | miRWalk |
| *SPP1* | hsa-miR-3136-5p | miRWalk |
| *SPP1* | hsa-miR-548u | miRWalk |
| *SPP1* | hsa-miR-3148 | miRWalk |
| *SPP1* | hsa-miR-3170 | miRWalk |
| *SPP1* | hsa-miR-3173-3p | miRWalk |
| *SPP1* | hsa-miR-4306 | miRWalk |
| *SPP1* | hsa-miR-4319 | miRWalk |
| *SPP1* | hsa-miR-4257 | miRWalk |
| *SPP1* | hsa-miR-4269 | miRWalk |
| *SPP1* | hsa-miR-4263 | miRWalk |
| *SPP1* | hsa-miR-4264 | miRWalk |
| *SPP1* | hsa-miR-4283 | miRWalk |
| *SPP1* | hsa-miR-4292 | miRWalk |
| *SPP1* | hsa-miR-4289 | miRWalk |
| *SPP1* | hsa-miR-4291 | miRWalk |
| *SPP1* | hsa-miR-3605-3p | miRWalk |
| *SPP1* | hsa-miR-3617-3p | miRWalk |
| *SPP1* | hsa-miR-3622a-5p | miRWalk |
| *SPP1* | hsa-miR-3650 | miRWalk |
| *SPP1* | hsa-miR-3682-3p | miRWalk |
| *SPP1* | hsa-miR-3691-5p | miRWalk |
| *SPP1* | hsa-miR-3689b-3p | miRWalk |
| *SPP1* | hsa-miR-3909 | miRWalk |
| *SPP1* | hsa-miR-3921 | miRWalk |
| *SPP1* | hsa-miR-3943 | miRWalk |
| *SPP1* | hsa-miR-4446-5p | miRWalk |
| *SPP1* | hsa-miR-4450 | miRWalk |
| *SPP1* | hsa-miR-4475 | miRWalk |
| *SPP1* | hsa-miR-3689c | miRWalk |
| *SPP1* | hsa-miR-3689d | miRWalk |
| *SPP1* | hsa-miR-4482-3p | miRWalk |
| *SPP1* | hsa-miR-4652-5p | miRWalk |
| *SPP1* | hsa-miR-4661-5p | miRWalk |
| *SPP1* | hsa-miR-4661-3p | miRWalk |
| *SPP1* | hsa-miR-4676-3p | miRWalk |
| *SPP1* | hsa-miR-4683 | miRWalk |
| *SPP1* | hsa-miR-4697-3p | miRWalk |
| *SPP1* | hsa-miR-4700-3p | miRWalk |
| *SPP1* | hsa-miR-4711-5p | miRWalk |
| *SPP1* | hsa-miR-4713-5p | miRWalk |
| *SPP1* | hsa-miR-4717-3p | miRWalk |
| *SPP1* | hsa-miR-4721 | miRWalk |
| *SPP1* | hsa-miR-4728-5p | miRWalk |
| *SPP1* | hsa-miR-4740-5p | miRWalk |
| *SPP1* | hsa-miR-4755-3p | miRWalk |
| *SPP1* | hsa-miR-4777-5p | miRWalk |
| *SPP1* | hsa-miR-2467-3p | miRWalk |
| *SPP1* | hsa-miR-4794 | miRWalk |
| *SPP1* | hsa-miR-5002-5p | miRWalk |
| *SPP1* | hsa-miR-5011-3p | miRWalk |
| *SPP1* | hsa-miR-5582-3p | miRWalk |
| *SPP1* | hsa-miR-5584-5p | miRWalk |
| *SPP1* | hsa-miR-5586-3p | miRWalk |
| *SPP1* | hsa-miR-5591-3p | miRWalk |
| *SPP1* | hsa-miR-5687 | miRWalk |
| *SPP1* | hsa-miR-4666b | miRWalk |
| *SPP1* | hsa-miR-6071 | miRWalk |
| *SPP1* | hsa-miR-6125 | miRWalk |
| *SPP1* | hsa-miR-6503-5p | miRWalk |
| *SPP1* | hsa-miR-6504-3p | miRWalk |
| *SPP1* | hsa-miR-6505-5p | miRWalk |
| *SPP1* | hsa-miR-6731-5p | miRWalk |
| *SPP1* | hsa-miR-6735-3p | miRWalk |
| *SPP1* | hsa-miR-6777-5p | miRWalk |
| *SPP1* | hsa-miR-6799-3p | miRWalk |
| *SPP1* | hsa-miR-6835-3p | miRWalk |
| *SPP1* | hsa-miR-6850-5p | miRWalk |
| *SPP1* | hsa-miR-6871-3p | miRWalk |
| *SPP1* | hsa-miR-6875-5p | miRWalk |
| *SPP1* | hsa-miR-6880-3p | miRWalk |
| *SPP1* | hsa-miR-6888-5p | miRWalk |
| *SPP1* | hsa-miR-6892-3p | miRWalk |
| *SPP1* | hsa-miR-7113-5p | miRWalk |
| *SPP1* | hsa-miR-7153-5p | miRWalk |
| *SPP1* | hsa-miR-7154-5p | miRWalk |
| *SPP1* | hsa-miR-7702 | miRWalk |
| *SPP1* | hsa-miR-8058 | miRWalk |
| *SPP1* | hsa-miR-8059 | miRWalk |
| *SPP1* | hsa-miR-8071 | miRWalk |
| *SPP1* | hsa-miR-9986 | miRWalk |
| *SPP1* | hsa-miR-10226 | miRWalk |
| *SPP1* | hsa-miR-10395-3p | miRWalk |
| *SPP1* | hsa-miR-10398-5p | miRWalk |
| *SPP1* | hsa-miR-10396b-5p | miRWalk |
| *SPP1* | hsa-miR-12127 | miRWalk |
| *SPP1* | hsa-miR-30d-3p | miRWalk |
| *SPP1* | hsa-miR-302d-5p | miRWalk |
| *SPP1* | hsa-miR-503-5p | miRWalk |
| *SPP1* | hsa-miR-611 | miRWalk |
| *SPP1* | hsa-miR-641 | miRWalk |
| *SPP1* | hsa-miR-3155b | miRWalk |
| *SPP1* | hsa-miR-4530 | miRWalk |
| *SPP1* | hsa-miR-4681 | miRWalk |
| *SPP1* | hsa-miR-4751 | miRWalk |
| *SPP1* | hsa-miR-5000-3p | miRWalk |
| *SPP1* | hsa-miR-5187-3p | miRWalk |
| *SPP1* | hsa-miR-1295b-5p | miRWalk |
| *SPP1* | hsa-miR-6074 | miRWalk |
| *SPP1* | hsa-miR-6807-3p | miRWalk |
| *SPP1* | hsa-miR-6819-5p | miRWalk |
| *SPP1* | hsa-miR-6849-5p | miRWalk |
| *SPP1* | hsa-miR-6873-5p | miRWalk |
| *SPP1* | hsa-miR-6876-5p | miRWalk |
| *SPP1* | hsa-miR-7843-5p | miRWalk |
| *SPP1* | hsa-miR-101-5p | miRWalk |
| *SPP1* | hsa-miR-192-3p | miRWalk |
| *SPP1* | hsa-miR-194-5p | miRWalk |
| *SPP1* | hsa-miR-106b-3p | miRWalk |
| *SPP1* | hsa-miR-370-3p | miRWalk |
| *SPP1* | hsa-miR-375-3p | miRWalk |
| *SPP1* | hsa-miR-412-3p | miRWalk |
| *SPP1* | hsa-miR-492 | miRWalk |
| *SPP1* | hsa-miR-520a-3p | miRWalk |
| *SPP1* | hsa-miR-505-3p | miRWalk |
| *SPP1* | hsa-miR-513a-3p | miRWalk |
| *SPP1* | hsa-miR-575 | miRWalk |
| *SPP1* | hsa-miR-639 | miRWalk |
| *SPP1* | hsa-miR-301b-3p | miRWalk |
| *SPP1* | hsa-miR-1205 | miRWalk |
| *SPP1* | hsa-miR-1258 | miRWalk |
| *SPP1* | hsa-miR-1260a | miRWalk |
| *SPP1* | hsa-miR-2116-3p | miRWalk |
| *SPP1* | hsa-miR-3152-3p | miRWalk |
| *SPP1* | hsa-miR-323b-3p | miRWalk |
| *SPP1* | hsa-miR-3194-5p | miRWalk |
| *SPP1* | hsa-miR-3202 | miRWalk |
| *SPP1* | hsa-miR-4258 | miRWalk |
| *SPP1* | hsa-miR-3672 | miRWalk |
| *SPP1* | hsa-miR-4512 | miRWalk |
| *SPP1* | hsa-miR-4524a-3p | miRWalk |
| *SPP1* | hsa-miR-4633-5p | miRWalk |
| *SPP1* | hsa-miR-4730 | miRWalk |
| *SPP1* | hsa-miR-4735-5p | miRWalk |
| *SPP1* | hsa-miR-4737 | miRWalk |
| *SPP1* | hsa-miR-4766-3p | miRWalk |
| *SPP1* | hsa-miR-4780 | miRWalk |
| *SPP1* | hsa-miR-4781-3p | miRWalk |
| *SPP1* | hsa-miR-5195-5p | miRWalk |
| *SPP1* | hsa-miR-5704 | miRWalk |
| *SPP1* | hsa-miR-6130 | miRWalk |
| *SPP1* | hsa-miR-548az-5p | miRWalk |
| *SPP1* | hsa-miR-6514-3p | miRWalk |
| *SPP1* | hsa-miR-6757-3p | miRWalk |
| *SPP1* | hsa-miR-6760-5p | miRWalk |
| *SPP1* | hsa-miR-6767-3p | miRWalk |
| *SPP1* | hsa-miR-6804-5p | miRWalk |
| *SPP1* | hsa-miR-6811-5p | miRWalk |
| *SPP1* | hsa-miR-6818-5p | miRWalk |
| *SPP1* | hsa-miR-6819-3p | miRWalk |
| *SPP1* | hsa-miR-6824-3p | miRWalk |
| *SPP1* | hsa-miR-6843-3p | miRWalk |
| *SPP1* | hsa-miR-7160-3p | miRWalk |
| *SPP1* | hsa-miR-7705 | miRWalk |
| *SPP1* | hsa-miR-8057 | miRWalk |
| *SPP1* | hsa-miR-17-3p | miRWalk |
| *SPP1* | hsa-miR-18a-5p | miRWalk |
| *SPP1* | hsa-miR-31-5p | miRWalk |
| *SPP1* | hsa-miR-33a-5p | miRWalk |
| *SPP1* | hsa-miR-95-3p | miRWalk |
| *SPP1* | hsa-miR-103a-1-5p | miRWalk |
| *SPP1* | hsa-miR-16-2-3p | miRWalk |
| *SPP1* | hsa-miR-196a-5p | miRWalk |
| *SPP1* | hsa-miR-203a-5p | miRWalk |
| *SPP1* | hsa-let-7i-5p | miRWalk |
| *SPP1* | hsa-miR-140-3p | miRWalk |
| *SPP1* | hsa-miR-142-3p | miRWalk |
| *SPP1* | hsa-miR-191-5p | miRWalk |
| *SPP1* | hsa-miR-188-5p | miRWalk |
| *SPP1* | hsa-miR-296-5p | miRWalk |
| *SPP1* | hsa-miR-361-3p | miRWalk |
| *SPP1* | hsa-miR-373-3p | miRWalk |
| *SPP1* | hsa-miR-196b-5p | miRWalk |
| *SPP1* | hsa-miR-18b-5p | miRWalk |
| *SPP1* | hsa-miR-409-3p | miRWalk |
| *SPP1* | hsa-miR-486-3p | miRWalk |
| *SPP1* | hsa-miR-490-5p | miRWalk |
| *SPP1* | hsa-miR-490-3p | miRWalk |
| *SPP1* | hsa-miR-193b-3p | miRWalk |
| *SPP1* | hsa-miR-181d-3p | miRWalk |
| *SPP1* | hsa-miR-508-3p | miRWalk |
| *SPP1* | hsa-miR-532-3p | miRWalk |
| *SPP1* | hsa-miR-455-5p | miRWalk |
| *SPP1* | hsa-miR-570-5p | miRWalk |
| *SPP1* | hsa-miR-593-5p | miRWalk |
| *SPP1* | hsa-miR-595 | miRWalk |
| *SPP1* | hsa-miR-610 | miRWalk |
| *SPP1* | hsa-miR-617 | miRWalk |
| *SPP1* | hsa-miR-644a | miRWalk |
| *SPP1* | hsa-miR-645 | miRWalk |
| *SPP1* | hsa-miR-449b-5p | miRWalk |
| *SPP1* | hsa-miR-151b | miRWalk |
| *SPP1* | hsa-miR-300 | miRWalk |
| *SPP1* | hsa-miR-147b-3p | miRWalk |
| *SPP1* | hsa-miR-543 | miRWalk |
| *SPP1* | hsa-miR-940 | miRWalk |
| *SPP1* | hsa-miR-1225-3p | miRWalk |
| *SPP1* | hsa-miR-1245a | miRWalk |
| *SPP1* | hsa-miR-1825 | miRWalk |
| *SPP1* | hsa-miR-3126-5p | miRWalk |
| *SPP1* | hsa-miR-3166 | miRWalk |
| *SPP1* | hsa-miR-3192-3p | miRWalk |
| *SPP1* | hsa-miR-4299 | miRWalk |
| *SPP1* | hsa-miR-4271 | miRWalk |
| *SPP1* | hsa-miR-4274 | miRWalk |
| *SPP1* | hsa-miR-23c | miRWalk |
| *SPP1* | hsa-miR-3622b-3p | miRWalk |
| *SPP1* | hsa-miR-3667-5p | miRWalk |
| *SPP1* | hsa-miR-3714 | miRWalk |
| *SPP1* | hsa-miR-3150b-5p | miRWalk |
| *SPP1* | hsa-miR-4423-5p | miRWalk |
| *SPP1* | hsa-miR-548ah-5p | miRWalk |
| *SPP1* | hsa-miR-4452 | miRWalk |
| *SPP1* | hsa-miR-4456 | miRWalk |
| *SPP1* | hsa-miR-548ai | miRWalk |
| *SPP1* | hsa-miR-4465 | miRWalk |
| *SPP1* | hsa-miR-4469 | miRWalk |
| *SPP1* | hsa-miR-4520-5p | miRWalk |
| *SPP1* | hsa-miR-4522 | miRWalk |
| *SPP1* | hsa-miR-4642 | miRWalk |
| *SPP1* | hsa-miR-4664-5p | miRWalk |
| *SPP1* | hsa-miR-4669 | miRWalk |
| *SPP1* | hsa-miR-4703-3p | miRWalk |
| *SPP1* | hsa-miR-4707-3p | miRWalk |
| *SPP1* | hsa-miR-4708-3p | miRWalk |
| *SPP1* | hsa-miR-4729 | miRWalk |
| *SPP1* | hsa-miR-4731-3p | miRWalk |
| *SPP1* | hsa-miR-371b-3p | miRWalk |
| *SPP1* | hsa-miR-5003-5p | miRWalk |
| *SPP1* | hsa-miR-5003-3p | miRWalk |
| *SPP1* | hsa-miR-5089-5p | miRWalk |
| *SPP1* | hsa-miR-5587-3p | miRWalk |
| *SPP1* | hsa-miR-5699-5p | miRWalk |
| *SPP1* | hsa-miR-6081 | miRWalk |
| *SPP1* | hsa-miR-6128 | miRWalk |
| *SPP1* | hsa-miR-6134 | miRWalk |
| *SPP1* | hsa-miR-6508-5p | miRWalk |
| *SPP1* | hsa-miR-6509-5p | miRWalk |
| *SPP1* | hsa-miR-6719-3p | miRWalk |
| *SPP1* | hsa-miR-6738-3p | miRWalk |
| *SPP1* | hsa-miR-6758-5p | miRWalk |
| *SPP1* | hsa-miR-6768-5p | miRWalk |
| *SPP1* | hsa-miR-6777-3p | miRWalk |
| *SPP1* | hsa-miR-6840-5p | miRWalk |
| *SPP1* | hsa-miR-1273h-3p | miRWalk |
| *SPP1* | hsa-miR-7855-5p | miRWalk |
| *SPP1* | hsa-miR-8079 | miRWalk |
| *SPP1* | hsa-miR-10396a-3p | miRWalk |
| *SPP1* | hsa-miR-10396b-3p | miRWalk |
| *SPP1* | hsa-miR-10522-5p | miRWalk |
