# Supplementary material for: Integrated bioinformatics analysis for the identification of idiopathic pulmonary fibrosis–related genes and potential therapeutic drugs
Source: BMC Pulm Med. 2023 Oct 4;23:373. doi: 10.1186/s12890-023-02678-z (PMC10552267; doi:10.1186/s12890-023-02678-z)
Supplement: Supplementary file 1 — Additional file 1: Table S1. The analyze network results of 1640 DEGs. Table S2. GO terms of the 18 hub genes. Table S3. KEGG pathways of the 18 hub genes. Table S4. Target microRNAs of SPP1 based on five online miRNA databases. Table S5. Target microRNAs of VEGFA based on five online miRNA databases. Table S6. Target microRNAs of COL1A1 based on five online miRNA databases. Table S7. Target microRNAs of CAV1 based on five online miRNA databases. Table S8. Target microRNAs of PECAM1 based on five online miRNA databases. Table S9. Target microRNAs of BMP4 based on five online miRNA databases. Table S10. Target microRNAs of FYN based on five online miRNA databases. Table S11. Traditional Chinese medicine prediction results of COL1A1. Table S12. Traditional Chinese medicine prediction results of VEGFA. Table S13. Traditional Chinese medicine prediction results of SPP1. [file 12890_2023_2678_MOESM1_ESM.zip › Supplementary Tables/Supplementary Table5.docx]

**Table S5 Target microRNAs of *VEGFA* based on five online miRNA databases**

| Gene Symbol | microRNA | Database |
| --- | --- | --- |
| *VEGFA* | hsa-miR-195-5p | mirDIP |
| *VEGFA* | hsa-miR-15b-5p | mirDIP |
| *VEGFA* | hsa-miR-424-5p | mirDIP |
| *VEGFA* | hsa-miR-16-5p | mirDIP |
| *VEGFA* | hsa-miR-29b-3p | mirDIP |
| *VEGFA* | hsa-miR-29a-3p | mirDIP |
| *VEGFA* | hsa-miR-29c-3p | mirDIP |
| *VEGFA* | hsa-miR-15a-5p | mirDIP |
| *VEGFA* | hsa-miR-299-3p | mirDIP |
| *VEGFA* | hsa-miR-5011-5p | mirDIP |
| *VEGFA* | hsa-miR-140-5p | mirDIP |
| *VEGFA* | hsa-miR-185-5p | mirDIP |
| *VEGFA* | hsa-miR-206 | mirDIP |
| *VEGFA* | hsa-miR-205-5p | mirDIP |
| *VEGFA* | hsa-miR-1277-5p | mirDIP |
| *VEGFA* | hsa-miR-361-5p | mirDIP |
| *VEGFA* | hsa-miR-452-5p | mirDIP |
| *VEGFA* | hsa-miR-655-3p | mirDIP |
| *VEGFA* | hsa-miR-29b-2-5p | mirDIP |
| *VEGFA* | hsa-miR-548p | mirDIP |
| *VEGFA* | hsa-miR-4306 | mirDIP |
| *VEGFA* | hsa-miR-7-1-3p | mirDIP |
| *VEGFA* | hsa-miR-4742-3p | mirDIP |
| *VEGFA* | hsa-miR-7-2-3p | mirDIP |
| *VEGFA* | hsa-miR-3668 | mirDIP |
| *VEGFA* | hsa-miR-4644 | mirDIP |
| *VEGFA* | hsa-miR-576-5p | mirDIP |
| *VEGFA* | hsa-miR-2115-3p | mirDIP |
| *VEGFA* | hsa-miR-877-3p | mirDIP |
| *VEGFA* | hsa-miR-520g-3p | mirDIP |
| *VEGFA* | hsa-miR-5682 | mirDIP |
| *VEGFA* | hsa-miR-2053 | mirDIP |
| *VEGFA* | hsa-miR-297 | mirDIP |
| *VEGFA* | hsa-miR-939-5p | mirDIP |
| *VEGFA* | hsa-miR-374a-3p | mirDIP |
| *VEGFA* | hsa-miR-578 | mirDIP |
| *VEGFA* | hsa-miR-190a-3p | mirDIP |
| *VEGFA* | hsa-miR-374b-3p | mirDIP |
| *VEGFA* | hsa-miR-889-3p | mirDIP |
| *VEGFA* | hsa-miR-892c-3p | mirDIP |
| *VEGFA* | hsa-miR-613 | mirDIP |
| *VEGFA* | hsa-miR-383-5p | mirDIP |
| *VEGFA* | hsa-miR-5196-5p | mirDIP |
| *VEGFA* | hsa-miR-3650 | mirDIP |
| *VEGFA* | hsa-miR-4524a-5p | mirDIP |
| *VEGFA* | hsa-miR-451b | mirDIP |
| *VEGFA* | hsa-miR-4524b-5p | mirDIP |
| *VEGFA* | hsa-miR-3908 | mirDIP |
| *VEGFA* | hsa-miR-5706 | mirDIP |
| *VEGFA* | hsa-miR-4455 | mirDIP |
| *VEGFA* | hsa-miR-141-5p | mirDIP |
| *VEGFA* | hsa-miR-3618 | mirDIP |
| *VEGFA* | hsa-miR-4676-3p | mirDIP |
| *VEGFA* | hsa-miR-4279 | mirDIP |
| *VEGFA* | hsa-miR-1343-3p | mirDIP |
| *VEGFA* | hsa-miR-4782-5p | mirDIP |
| *VEGFA* | hsa-miR-134-5p | mirDIP |
| *VEGFA* | hsa-miR-423-3p | mirDIP |
| *VEGFA* | hsa-miR-4428 | mirDIP |
| *VEGFA* | hsa-miR-8085 | mirDIP |
| *VEGFA* | hsa-miR-548av-3p | mirDIP |
| *VEGFA* | hsa-miR-1343-5p | mirDIP |
| *VEGFA* | hsa-miR-6731-5p | mirDIP |
| *VEGFA* | hsa-miR-6754-5p | mirDIP |
| *VEGFA* | hsa-miR-4766-3p | mirDIP |
| *VEGFA* | hsa-miR-4261 | mirDIP |
| *VEGFA* | hsa-miR-6748-5p | mirDIP |
| *VEGFA* | hsa-miR-3659 | mirDIP |
| *VEGFA* | hsa-miR-4274 | mirDIP |
| *VEGFA* | hsa-miR-943 | mirDIP |
| *VEGFA* | hsa-miR-3922-3p | mirDIP |
| *VEGFA* | hsa-miR-3152-3p | mirDIP |
| *VEGFA* | hsa-miR-6852-5p | mirDIP |
| *VEGFA* | hsa-miR-6809-3p | mirDIP |
| *VEGFA* | hsa-miR-5186 | mirDIP |
| *VEGFA* | hsa-miR-4743-3p | mirDIP |
| *VEGFA* | hsa-miR-5007-5p | mirDIP |
| *VEGFA* | hsa-miR-6777-5p | mirDIP |
| *VEGFA* | hsa-miR-6895-3p | mirDIP |
| *VEGFA* | hsa-miR-6889-5p | mirDIP |
| *VEGFA* | hsa-miR-4648 | mirDIP |
| *VEGFA* | hsa-miR-1247-3p | mirDIP |
| *VEGFA* | hsa-miR-1470 | mirDIP |
| *VEGFA* | hsa-miR-6090 | mirDIP |
| *VEGFA* | hsa-miR-6501-5p | mirDIP |
| *VEGFA* | hsa-miR-425-3p | mirDIP |
| *VEGFA* | hsa-miR-638 | mirDIP |
| *VEGFA* | hsa-miR-6870-3p | mirDIP |
| *VEGFA* | hsa-miR-598-5p | mirDIP |
| *VEGFA* | hsa-miR-126-3p | mirDIP |
| *VEGFA* | hsa-miR-6879-3p | mirDIP |
| *VEGFA* | hsa-miR-4467 | mirDIP |
| *VEGFA* | hsa-miR-4788 | mirDIP |
| *VEGFA* | hsa-miR-6863 | mirDIP |
| *VEGFA* | hsa-miR-3912-3p | mirDIP |
| *VEGFA* | hsa-miR-4497 | mirDIP |
| *VEGFA* | hsa-miR-12129 | mirDIP |
| *VEGFA* | hsa-miR-6529-3p | mirDIP |
| *VEGFA* | hsa-miR-200b-3p | ENCORI |
| *VEGFA* | hsa-miR-200a-3p | ENCORI |
| *VEGFA* | hsa-miR-429 | ENCORI |
| *VEGFA* | hsa-miR-34a-5p | ENCORI |
| *VEGFA* | hsa-miR-30e-5p | ENCORI |
| *VEGFA* | hsa-miR-30c-5p | ENCORI |
| *VEGFA* | hsa-miR-101-3p | ENCORI |
| *VEGFA* | hsa-miR-186-5p | ENCORI |
| *VEGFA* | hsa-miR-3118 | ENCORI |
| *VEGFA* | hsa-miR-190b | ENCORI |
| *VEGFA* | hsa-miR-199a-5p | ENCORI |
| *VEGFA* | hsa-miR-29c-3p | ENCORI |
| *VEGFA* | hsa-miR-29b-3p | ENCORI |
| *VEGFA* | hsa-miR-205-5p | ENCORI |
| *VEGFA* | hsa-miR-107 | ENCORI |
| *VEGFA* | hsa-miR-302e | ENCORI |
| *VEGFA* | hsa-miR-129-5p | ENCORI |
| *VEGFA* | hsa-miR-139-5p | ENCORI |
| *VEGFA* | hsa-miR-34b-5p | ENCORI |
| *VEGFA* | hsa-miR-34c-5p | ENCORI |
| *VEGFA* | hsa-miR-125b-5p | ENCORI |
| *VEGFA* | hsa-miR-200c-3p | ENCORI |
| *VEGFA* | hsa-miR-141-3p | ENCORI |
| *VEGFA* | hsa-miR-613 | ENCORI |
| *VEGFA* | hsa-miR-148b-3p | ENCORI |
| *VEGFA* | hsa-miR-26a-5p | ENCORI |
| *VEGFA* | hsa-miR-16-5p | ENCORI |
| *VEGFA* | hsa-miR-15a-5p | ENCORI |
| *VEGFA* | hsa-miR-17-5p | ENCORI |
| *VEGFA* | hsa-miR-20a-5p | ENCORI |
| *VEGFA* | hsa-miR-4306 | ENCORI |
| *VEGFA* | hsa-miR-136-5p | ENCORI |
| *VEGFA* | hsa-miR-299-3p | ENCORI |
| *VEGFA* | hsa-miR-329-3p | ENCORI |
| *VEGFA* | hsa-miR-494-3p | ENCORI |
| *VEGFA* | hsa-miR-543 | ENCORI |
| *VEGFA* | hsa-miR-495-3p | ENCORI |
| *VEGFA* | hsa-miR-376c-3p | ENCORI |
| *VEGFA* | hsa-miR-300 | ENCORI |
| *VEGFA* | hsa-miR-381-3p | ENCORI |
| *VEGFA* | hsa-miR-382-5p | ENCORI |
| *VEGFA* | hsa-miR-134-5p | ENCORI |
| *VEGFA* | hsa-miR-377-3p | ENCORI |
| *VEGFA* | hsa-miR-410-3p | ENCORI |
| *VEGFA* | hsa-miR-203a | ENCORI |
| *VEGFA* | hsa-miR-190a-5p | ENCORI |
| *VEGFA* | hsa-miR-184 | ENCORI |
| *VEGFA* | hsa-miR-138-5p | ENCORI |
| *VEGFA* | hsa-miR-140-5p | ENCORI |
| *VEGFA* | hsa-miR-195-5p | ENCORI |
| *VEGFA* | hsa-miR-497-5p | ENCORI |
| *VEGFA* | hsa-miR-451a | ENCORI |
| *VEGFA* | hsa-miR-144-3p | ENCORI |
| *VEGFA* | hsa-miR-1 | ENCORI |
| *VEGFA* | hsa-miR-4319 | ENCORI |
| *VEGFA* | hsa-miR-23a-3p | ENCORI |
| *VEGFA* | hsa-miR-150-5p | ENCORI |
| *VEGFA* | hsa-miR-125a-5p | ENCORI |
| *VEGFA* | hsa-miR-125a-3p | ENCORI |
| *VEGFA* | hsa-miR-520e | ENCORI |
| *VEGFA* | hsa-miR-520a-3p | ENCORI |
| *VEGFA* | hsa-miR-518f-3p | ENCORI |
| *VEGFA* | hsa-miR-520b | ENCORI |
| *VEGFA* | hsa-miR-520c-3p | ENCORI |
| *VEGFA* | hsa-miR-517a-3p | ENCORI |
| *VEGFA* | hsa-miR-519d-3p | ENCORI |
| *VEGFA* | hsa-miR-520d-3p | ENCORI |
| *VEGFA* | hsa-miR-372-3p | ENCORI |
| *VEGFA* | hsa-miR-373-3p | ENCORI |
| *VEGFA* | hsa-miR-216a-5p | ENCORI |
| *VEGFA* | hsa-miR-375 | ENCORI |
| *VEGFA* | hsa-miR-153-3p | ENCORI |
| *VEGFA* | hsa-miR-103a-3p | ENCORI |
| *VEGFA* | hsa-miR-499a-5p | ENCORI |
| *VEGFA* | hsa-miR-155-5p | ENCORI |
| *VEGFA* | hsa-miR-185-5p | ENCORI |
| *VEGFA* | hsa-miR-378b | ENCORI |
| *VEGFA* | hsa-miR-191-5p | ENCORI |
| *VEGFA* | hsa-miR-15b-5p | ENCORI |
| *VEGFA* | hsa-miR-378d | ENCORI |
| *VEGFA* | hsa-miR-302d-3p | ENCORI |
| *VEGFA* | hsa-miR-302a-3p | ENCORI |
| *VEGFA* | hsa-miR-302c-3p | ENCORI |
| *VEGFA* | hsa-miR-302b-3p | ENCORI |
| *VEGFA* | hsa-miR-449a | ENCORI |
| *VEGFA* | hsa-miR-449b-5p | ENCORI |
| *VEGFA* | hsa-miR-874-3p | ENCORI |
| *VEGFA* | hsa-miR-340-5p | ENCORI |
| *VEGFA* | hsa-miR-206 | ENCORI |
| *VEGFA* | hsa-miR-30a-5p | ENCORI |
| *VEGFA* | hsa-miR-4644 | ENCORI |
| *VEGFA* | hsa-miR-339-5p | ENCORI |
| *VEGFA* | hsa-miR-148a-3p | ENCORI |
| *VEGFA* | hsa-miR-590-3p | ENCORI |
| *VEGFA* | hsa-miR-653-5p | ENCORI |
| *VEGFA* | hsa-miR-93-5p | ENCORI |
| *VEGFA* | hsa-miR-106b-5p | ENCORI |
| *VEGFA* | hsa-miR-29a-3p | ENCORI |
| *VEGFA* | hsa-miR-383-5p | ENCORI |
| *VEGFA* | hsa-miR-486-5p | ENCORI |
| *VEGFA* | hsa-miR-30b-5p | ENCORI |
| *VEGFA* | hsa-miR-30d-5p | ENCORI |
| *VEGFA* | hsa-miR-491-5p | ENCORI |
| *VEGFA* | hsa-miR-31-5p | ENCORI |
| *VEGFA* | hsa-miR-876-5p | ENCORI |
| *VEGFA* | hsa-miR-23b-3p | ENCORI |
| *VEGFA* | hsa-miR-199b-5p | ENCORI |
| *VEGFA* | hsa-miR-126-3p | ENCORI |
| *VEGFA* | hsa-miR-362-3p | ENCORI |
| *VEGFA* | hsa-miR-374b-5p | ENCORI |
| *VEGFA* | hsa-miR-374a-5p | ENCORI |
| *VEGFA* | hsa-miR-361-5p | ENCORI |
| *VEGFA* | hsa-miR-448 | ENCORI |
| *VEGFA* | hsa-miR-20b-5p | ENCORI |
| *VEGFA* | hsa-miR-106a-5p | ENCORI |
| *VEGFA* | hsa-miR-503-5p | ENCORI |
| *VEGFA* | hsa-miR-424-5p | ENCORI |
| *VEGFA* | hsa-miR-205-5p | TargetScan |
| *VEGFA* | hsa-miR-302e | TargetScan |
| *VEGFA* | hsa-miR-520b | TargetScan |
| *VEGFA* | hsa-miR-302d-3p | TargetScan |
| *VEGFA* | hsa-miR-302a-3p | TargetScan |
| *VEGFA* | hsa-miR-302b-3p | TargetScan |
| *VEGFA* | hsa-miR-520c-3p | TargetScan |
| *VEGFA* | hsa-miR-302c-3p.1 | TargetScan |
| *VEGFA* | hsa-miR-372-3p | TargetScan |
| *VEGFA* | hsa-miR-520e | TargetScan |
| *VEGFA* | hsa-miR-520d-3p | TargetScan |
| *VEGFA* | hsa-miR-373-3p | TargetScan |
| *VEGFA* | hsa-miR-520a-3p | TargetScan |
| *VEGFA* | hsa-miR-106b-5p | TargetScan |
| *VEGFA* | hsa-miR-20a-5p | TargetScan |
| *VEGFA* | hsa-miR-519d-3p | TargetScan |
| *VEGFA* | hsa-miR-93-5p | TargetScan |
| *VEGFA* | hsa-miR-106a-5p | TargetScan |
| *VEGFA* | hsa-miR-20b-5p | TargetScan |
| *VEGFA* | hsa-miR-17-5p | TargetScan |
| *VEGFA* | hsa-miR-526b-3p | TargetScan |
| *VEGFA* | hsa-miR-195-5p | TargetScan |
| *VEGFA* | hsa-miR-15a-5p | TargetScan |
| *VEGFA* | hsa-miR-497-5p | TargetScan |
| *VEGFA* | hsa-miR-424-5p | TargetScan |
| *VEGFA* | hsa-miR-6838-5p | TargetScan |
| *VEGFA* | hsa-miR-16-5p | TargetScan |
| *VEGFA* | hsa-miR-15b-5p | TargetScan |
| *VEGFA* | hsa-miR-299-3p | TargetScan |
| *VEGFA* | hsa-miR-199a-5p | TargetScan |
| *VEGFA* | hsa-miR-199b-5p | TargetScan |
| *VEGFA* | hsa-miR-140-3p.1 | TargetScan |
| *VEGFA* | hsa-miR-140-5p | TargetScan |
| *VEGFA* | hsa-miR-377-3p | TargetScan |
| *VEGFA* | hsa-miR-892c-3p | TargetScan |
| *VEGFA* | hsa-miR-452-5p | TargetScan |
| *VEGFA* | hsa-miR-4676-3p | TargetScan |
| *VEGFA* | hsa-miR-429 | TargetScan |
| *VEGFA* | hsa-miR-200c-3p | TargetScan |
| *VEGFA* | hsa-miR-200b-3p | TargetScan |
| *VEGFA* | hsa-miR-613 | TargetScan |
| *VEGFA* | hsa-miR-206 | TargetScan |
| *VEGFA* | hsa-miR-1-3p | TargetScan |
| *VEGFA* | hsa-miR-361-5p | TargetScan |
| *VEGFA* | hsa-miR-29c-3p | TargetScan |
| *VEGFA* | hsa-miR-29b-3p | TargetScan |
| *VEGFA* | hsa-miR-29a-3p | TargetScan |
| *VEGFA* | hsa-miR-374c-5p | TargetScan |
| *VEGFA* | hsa-miR-655-3p | TargetScan |
| *VEGFA* | hsa-miR-383-5p.1 | TargetScan |
| *VEGFA* | hsa-miR-383-5p.2 | TargetScan |
| *VEGFA* | hsa-miR-5011-5p | DIANA-micro T |
| *VEGFA* | hsa-miR-1277-5p | DIANA-micro T |
| *VEGFA* | hsa-miR-190a-3p | DIANA-micro T |
| *VEGFA* | hsa-miR-16-5p | DIANA-micro T |
| *VEGFA* | hsa-miR-195-5p | DIANA-micro T |
| *VEGFA* | hsa-miR-361-5p | DIANA-micro T |
| *VEGFA* | hsa-miR-2115-3p | DIANA-micro T |
| *VEGFA* | hsa-miR-15a-5p | DIANA-micro T |
| *VEGFA* | hsa-miR-5706 | DIANA-micro T |
| *VEGFA* | hsa-miR-4782-5p | DIANA-micro T |
| *VEGFA* | hsa-miR-889-3p | DIANA-micro T |
| *VEGFA* | hsa-miR-15b-5p | DIANA-micro T |
| *VEGFA* | hsa-miR-6839-5p | DIANA-micro T |
| *VEGFA* | hsa-miR-3163 | DIANA-micro T |
| *VEGFA* | hsa-miR-4742-3p | DIANA-micro T |
| *VEGFA* | hsa-miR-29b-3p | DIANA-micro T |
| *VEGFA* | hsa-miR-4524a-5p | DIANA-micro T |
| *VEGFA* | hsa-miR-205-5p | DIANA-micro T |
| *VEGFA* | hsa-miR-29c-3p | DIANA-micro T |
| *VEGFA* | hsa-miR-29a-3p | DIANA-micro T |
| *VEGFA* | hsa-miR-4644 | DIANA-micro T |
| *VEGFA* | hsa-miR-4524b-5p | DIANA-micro T |
| *VEGFA* | hsa-miR-374c-5p | DIANA-micro T |
| *VEGFA* | hsa-miR-3908 | DIANA-micro T |
| *VEGFA* | hsa-miR-5692a | DIANA-micro T |
| *VEGFA* | hsa-miR-4441 | DIANA-micro T |
| *VEGFA* | hsa-miR-6748-5p | DIANA-micro T |
| *VEGFA* | hsa-miR-877-3p | DIANA-micro T |
| *VEGFA* | hsa-miR-548x-3p | DIANA-micro T |
| *VEGFA* | hsa-miR-548aj-3p | DIANA-micro T |
| *VEGFA* | hsa-miR-3121-3p | DIANA-micro T |
| *VEGFA* | hsa-miR-2053 | DIANA-micro T |
| *VEGFA* | hsa-miR-424-5p | DIANA-micro T |
| *VEGFA* | hsa-miR-374b-3p | DIANA-micro T |
| *VEGFA* | hsa-miR-3668 | DIANA-micro T |
| *VEGFA* | hsa-miR-6783-3p | DIANA-micro T |
| *VEGFA* | hsa-miR-548p | DIANA-micro T |
| *VEGFA* | hsa-miR-3924 | DIANA-micro T |
| *VEGFA* | hsa-miR-5682 | DIANA-micro T |
| *VEGFA* | hsa-miR-1236-3p | DIANA-micro T |
| *VEGFA* | hsa-miR-410-3p | DIANA-micro T |
| *VEGFA* | hsa-miR-497-5p | DIANA-micro T |
| *VEGFA* | hsa-miR-374a-3p | DIANA-micro T |
| *VEGFA* | hsa-miR-944 | DIANA-micro T |
| *VEGFA* | hsa-miR-6838-5p | DIANA-micro T |
| *VEGFA* | hsa-miR-548ar-3p | DIANA-micro T |
| *VEGFA* | hsa-miR-299-3p | DIANA-micro T |
| *VEGFA* | hsa-miR-1299 | DIANA-micro T |
| *VEGFA* | hsa-miR-6501-5p | DIANA-micro T |
| *VEGFA* | hsa-miR-95-5p | DIANA-micro T |
| *VEGFA* | hsa-miR-378j | DIANA-micro T |
| *VEGFA* | hsa-miR-5590-3p | DIANA-micro T |
| *VEGFA* | hsa-miR-613 | DIANA-micro T |
| *VEGFA* | hsa-miR-300 | DIANA-micro T |
| *VEGFA* | hsa-miR-381-3p | DIANA-micro T |
| *VEGFA* | hsa-miR-548ac | DIANA-micro T |
| *VEGFA* | hsa-miR-655-3p | DIANA-micro T |
| *VEGFA* | hsa-miR-548aq-3p | DIANA-micro T |
| *VEGFA* | hsa-miR-4282 | DIANA-micro T |
| *VEGFA* | hsa-miR-548ae-3p | DIANA-micro T |
| *VEGFA* | hsa-miR-548ah-3p | DIANA-micro T |
| *VEGFA* | hsa-miR-548z | DIANA-micro T |
| *VEGFA* | hsa-miR-548h-3p | DIANA-micro T |
| *VEGFA* | hsa-miR-548am-3p | DIANA-micro T |
| *VEGFA* | hsa-miR-3650 | DIANA-micro T |
| *VEGFA* | hsa-miR-548f-3p | DIANA-micro T |
| *VEGFA* | hsa-miR-548az-3p | DIANA-micro T |
| *VEGFA* | hsa-miR-548j-3p | DIANA-micro T |
| *VEGFA* | hsa-miR-567 | DIANA-micro T |
| *VEGFA* | hsa-miR-503-5p | DIANA-micro T |
| *VEGFA* | hsa-miR-29b-2-5p | DIANA-micro T |
| *VEGFA* | hsa-miR-3618 | DIANA-micro T |
| *VEGFA* | hsa-miR-510-3p | DIANA-micro T |
| *VEGFA* | hsa-miR-3143 | DIANA-micro T |
| *VEGFA* | hsa-miR-1343-3p | DIANA-micro T |
| *VEGFA* | hsa-miR-339-5p | DIANA-micro T |
| *VEGFA* | hsa-miR-4517 | DIANA-micro T |
| *VEGFA* | hsa-miR-4788 | DIANA-micro T |
| *VEGFA* | hsa-miR-3169 | DIANA-micro T |
| *VEGFA* | hsa-miR-6830-3p | DIANA-micro T |
| *VEGFA* | hsa-miR-4775 | DIANA-micro T |
| *VEGFA* | hsa-miR-6758-5p | DIANA-micro T |
| *VEGFA* | hsa-miR-7852-3p | DIANA-micro T |
| *VEGFA* | hsa-miR-548av-3p | DIANA-micro T |
| *VEGFA* | hsa-miR-5186 | DIANA-micro T |
| *VEGFA* | hsa-miR-4766-3p | DIANA-micro T |
| *VEGFA* | hsa-miR-6856-5p | DIANA-micro T |
| *VEGFA* | hsa-miR-4306 | DIANA-micro T |
| *VEGFA* | hsa-miR-3941 | DIANA-micro T |
| *VEGFA* | hsa-miR-548e-3p | DIANA-micro T |
| *VEGFA* | hsa-miR-3686 | DIANA-micro T |
| *VEGFA* | hsa-miR-185-5p | DIANA-micro T |
| *VEGFA* | hsa-miR-8485 | DIANA-micro T |
| *VEGFA* | hsa-miR-429 | DIANA-micro T |
| *VEGFA* | hsa-miR-495-5p | DIANA-micro T |
| *VEGFA* | hsa-miR-6875-5p | DIANA-micro T |
| *VEGFA* | hsa-miR-374b-5p | DIANA-micro T |
| *VEGFA* | hsa-miR-5583-3p | DIANA-micro T |
| *VEGFA* | hsa-miR-452-5p | DIANA-micro T |
| *VEGFA* | hsa-miR-675-3p | DIANA-micro T |
| *VEGFA* | hsa-miR-5582-3p | DIANA-micro T |
| *VEGFA* | hsa-miR-5571-5p | DIANA-micro T |
| *VEGFA* | hsa-miR-451b | DIANA-micro T |
| *VEGFA* | hsa-miR-892c-3p | DIANA-micro T |
| *VEGFA* | hsa-miR-526b-3p | DIANA-micro T |
| *VEGFA* | hsa-miR-6833-3p | DIANA-micro T |
| *VEGFA* | hsa-miR-4673 | DIANA-micro T |
| *VEGFA* | hsa-miR-627-3p | DIANA-micro T |
| *VEGFA* | hsa-miR-4753-3p | DIANA-micro T |
| *VEGFA* | hsa-miR-3659 | DIANA-micro T |
| *VEGFA* | hsa-miR-4274 | DIANA-micro T |
| *VEGFA* | hsa-miR-548d-3p | DIANA-micro T |
| *VEGFA* | hsa-miR-3145-3p | DIANA-micro T |
| *VEGFA* | hsa-miR-200b-3p | DIANA-micro T |
| *VEGFA* | hsa-miR-4703-5p | DIANA-micro T |
| *VEGFA* | hsa-miR-106a-5p | DIANA-micro T |
| *VEGFA* | hsa-miR-548bb-3p | DIANA-micro T |
| *VEGFA* | hsa-miR-4279 | DIANA-micro T |
| *VEGFA* | hsa-miR-4455 | DIANA-micro T |
| *VEGFA* | hsa-miR-200c-3p | DIANA-micro T |
| *VEGFA* | hsa-miR-8085 | DIANA-micro T |
| *VEGFA* | hsa-miR-548a-3p | DIANA-micro T |
| *VEGFA* | hsa-miR-6888-3p | DIANA-micro T |
| *VEGFA* | hsa-miR-543 | DIANA-micro T |
| *VEGFA* | hsa-miR-6769b-5p | DIANA-micro T |
| *VEGFA* | hsa-miR-6809-3p | DIANA-micro T |
| *VEGFA* | hsa-miR-1247-3p | DIANA-micro T |
| *VEGFA* | hsa-miR-569 | DIANA-micro T |
| *VEGFA* | hsa-miR-1468-3p | DIANA-micro T |
| *VEGFA* | hsa-miR-4662a-3p | DIANA-micro T |
| *VEGFA* | hsa-miR-590-3p | DIANA-micro T |
| *VEGFA* | hsa-miR-6852-5p | DIANA-micro T |
| *VEGFA* | hsa-miR-520a-3p | DIANA-micro T |
| *VEGFA* | hsa-miR-4676-3p | DIANA-micro T |
| *VEGFA* | hsa-miR-140-5p | DIANA-micro T |
| *VEGFA* | hsa-miR-3935 | DIANA-micro T |
| *VEGFA* | hsa-miR-106b-5p | DIANA-micro T |
| *VEGFA* | hsa-miR-1185-1-3p | DIANA-micro T |
| *VEGFA* | hsa-miR-23c | DIANA-micro T |
| *VEGFA* | hsa-miR-23b-3p | DIANA-micro T |
| *VEGFA* | hsa-miR-20a-5p | DIANA-micro T |
| *VEGFA* | hsa-miR-6829-5p | DIANA-micro T |
| *VEGFA* | hsa-miR-520b | DIANA-micro T |
| *VEGFA* | hsa-miR-520c-3p | DIANA-micro T |
| *VEGFA* | hsa-miR-1185-2-3p | DIANA-micro T |
| *VEGFA* | hsa-miR-23a-3p | DIANA-micro T |
| *VEGFA* | hsa-miR-126-5p | DIANA-micro T |
| *VEGFA* | hsa-miR-939-5p | DIANA-micro T |
| *VEGFA* | hsa-miR-6507-5p | DIANA-micro T |
| *VEGFA* | hsa-miR-939-3p | DIANA-micro T |
| *VEGFA* | hsa-miR-556-3p | DIANA-micro T |
| *VEGFA* | hsa-miR-107 | DIANA-micro T |
| *VEGFA* | hsa-miR-3613-3p | DIANA-micro T |
| *VEGFA* | hsa-miR-7-1-3p | DIANA-micro T |
| *VEGFA* | hsa-miR-7-2-3p | DIANA-micro T |
| *VEGFA* | hsa-miR-520e | DIANA-micro T |
| *VEGFA* | hsa-miR-548c-3p | DIANA-micro T |
| *VEGFA* | hsa-miR-6887-3p | DIANA-micro T |
| *VEGFA* | hsa-miR-2052 | DIANA-micro T |
| *VEGFA* | hsa-miR-4460 | DIANA-micro T |
| *VEGFA* | hsa-miR-5001-3p | DIANA-micro T |
| *VEGFA* | hsa-miR-576-5p | DIANA-micro T |
| *VEGFA* | hsa-miR-6759-5p | DIANA-micro T |
| *VEGFA* | hsa-miR-1825 | DIANA-micro T |
| *VEGFA* | hsa-miR-372-3p | DIANA-micro T |
| *VEGFA* | hsa-miR-206 | DIANA-micro T |
| *VEGFA* | hsa-miR-5694 | DIANA-micro T |
| *VEGFA* | hsa-miR-1-3p | DIANA-micro T |
| *VEGFA* | hsa-miR-578 | DIANA-micro T |
| *VEGFA* | hsa-miR-103a-3p | DIANA-micro T |
| *VEGFA* | hsa-miR-6873-3p | DIANA-micro T |
| *VEGFA* | hsa-miR-548aa | DIANA-micro T |
| *VEGFA* | hsa-miR-548t-3p | DIANA-micro T |
| *VEGFA* | hsa-miR-6879-3p | DIANA-micro T |
| *VEGFA* | hsa-miR-5192 | DIANA-micro T |
| *VEGFA* | hsa-miR-186-5p | DIANA-micro T |
| *VEGFA* | hsa-miR-4745-5p | DIANA-micro T |
| *VEGFA* | hsa-miR-519d-3p | DIANA-micro T |
| *VEGFA* | hsa-miR-656-3p | DIANA-micro T |
| *VEGFA* | hsa-miR-4684-5p | DIANA-micro T |
| *VEGFA* | hsa-miR-642b-5p | DIANA-micro T |
| *VEGFA* | hsa-miR-205-3p | DIANA-micro T |
| *VEGFA* | hsa-miR-548as-3p | DIANA-micro T |
| *VEGFA* | hsa-miR-548ap-3p | DIANA-micro T |
| *VEGFA* | hsa-miR-8066 | DIANA-micro T |
| *VEGFA* | hsa-miR-4796-5p | DIANA-micro T |
| *VEGFA* | hsa-miR-6881-3p | DIANA-micro T |
| *VEGFA* | hsa-miR-138-2-3p | DIANA-micro T |
| *VEGFA* | hsa-miR-4270 | DIANA-micro T |
| *VEGFA* | hsa-miR-6805-3p | DIANA-micro T |
| *VEGFA* | hsa-miR-130a-5p | DIANA-micro T |
| *VEGFA* | hsa-miR-5193 | DIANA-micro T |
| *VEGFA* | hsa-miR-5580-3p | DIANA-micro T |
| *VEGFA* | hsa-miR-6895-3p | DIANA-micro T |
| *VEGFA* | hsa-miR-141-5p | DIANA-micro T |
| *VEGFA* | hsa-miR-5787 | DIANA-micro T |
| *VEGFA* | hsa-miR-1287-3p | miRWalk |
| *VEGFA* | hsa-miR-6514-5p | miRWalk |
| *VEGFA* | hsa-miR-6801-3p | miRWalk |
| *VEGFA* | hsa-miR-6129 | miRWalk |
| *VEGFA* | hsa-miR-6503-5p | miRWalk |
| *VEGFA* | hsa-miR-6506-5p | miRWalk |
| *VEGFA* | hsa-miR-6747-5p | miRWalk |
| *VEGFA* | hsa-miR-6820-5p | miRWalk |
| *VEGFA* | hsa-miR-6855-5p | miRWalk |
| *VEGFA* | hsa-miR-11181-3p | miRWalk |
| *VEGFA* | hsa-let-7a-5p | miRWalk |
| *VEGFA* | hsa-miR-4307 | miRWalk |
| *VEGFA* | hsa-miR-6802-5p | miRWalk |
| *VEGFA* | hsa-miR-6826-3p | miRWalk |
| *VEGFA* | hsa-miR-3064-5p | miRWalk |
| *VEGFA* | hsa-miR-4514 | miRWalk |
| *VEGFA* | hsa-miR-328-5p | miRWalk |
| *VEGFA* | hsa-miR-4537 | miRWalk |
| *VEGFA* | hsa-miR-6811-5p | miRWalk |
| *VEGFA* | hsa-miR-3614-3p | miRWalk |
| *VEGFA* | hsa-miR-4519 | miRWalk |
| *VEGFA* | hsa-miR-6876-5p | miRWalk |
| *VEGFA* | hsa-miR-6734-5p | miRWalk |
| *VEGFA* | hsa-miR-1908-5p | miRWalk |
| *VEGFA* | hsa-miR-3917 | miRWalk |
| *VEGFA* | hsa-miR-6737-5p | miRWalk |
| *VEGFA* | hsa-miR-10226 | miRWalk |
| *VEGFA* | hsa-miR-3664-5p | miRWalk |
| *VEGFA* | hsa-miR-6772-3p | miRWalk |
| *VEGFA* | hsa-miR-6769b-5p | miRWalk |
| *VEGFA* | hsa-miR-8052 | miRWalk |
| *VEGFA* | hsa-miR-331-5p | miRWalk |
| *VEGFA* | hsa-miR-302a-3p | miRWalk |
| *VEGFA* | hsa-let-7a-2-3p | miRWalk |
| *VEGFA* | hsa-let-7e-3p | miRWalk |
| *VEGFA* | hsa-miR-18a-3p | miRWalk |
| *VEGFA* | hsa-miR-19b-1-5p | miRWalk |
| *VEGFA* | hsa-miR-23a-3p | miRWalk |
| *VEGFA* | hsa-miR-24-3p | miRWalk |
| *VEGFA* | hsa-miR-25-3p | miRWalk |
| *VEGFA* | hsa-miR-27a-5p | miRWalk |
| *VEGFA* | hsa-miR-33a-3p | miRWalk |
| *VEGFA* | hsa-miR-92a-3p | miRWalk |
| *VEGFA* | hsa-miR-29b-1-5p | miRWalk |
| *VEGFA* | hsa-miR-103a-3p | miRWalk |
| *VEGFA* | hsa-miR-197-5p | miRWalk |
| *VEGFA* | hsa-miR-34a-5p | miRWalk |
| *VEGFA* | hsa-miR-210-3p | miRWalk |
| *VEGFA* | hsa-miR-216a-3p | miRWalk |
| *VEGFA* | hsa-miR-217-5p | miRWalk |
| *VEGFA* | hsa-miR-219a-1-3p | miRWalk |
| *VEGFA* | hsa-miR-222-5p | miRWalk |
| *VEGFA* | hsa-miR-200b-5p | miRWalk |
| *VEGFA* | hsa-miR-23b-5p | miRWalk |
| *VEGFA* | hsa-miR-30b-3p | miRWalk |
| *VEGFA* | hsa-miR-124-5p | miRWalk |
| *VEGFA* | hsa-miR-128-1-5p | miRWalk |
| *VEGFA* | hsa-miR-132-3p | miRWalk |
| *VEGFA* | hsa-miR-135a-2-3p | miRWalk |
| *VEGFA* | hsa-miR-137-5p | miRWalk |
| *VEGFA* | hsa-miR-138-5p | miRWalk |
| *VEGFA* | hsa-miR-141-5p | miRWalk |
| *VEGFA* | hsa-miR-125a-5p | miRWalk |
| *VEGFA* | hsa-miR-125b-2-3p | miRWalk |
| *VEGFA* | hsa-miR-127-3p | miRWalk |
| *VEGFA* | hsa-miR-149-5p | miRWalk |
| *VEGFA* | hsa-miR-149-3p | miRWalk |
| *VEGFA* | hsa-miR-195-5p | miRWalk |
| *VEGFA* | hsa-miR-320a-5p | miRWalk |
| *VEGFA* | hsa-miR-320a-3p | miRWalk |
| *VEGFA* | hsa-miR-106b-5p | miRWalk |
| *VEGFA* | hsa-miR-219a-2-3p | miRWalk |
| *VEGFA* | hsa-miR-34b-5p | miRWalk |
| *VEGFA* | hsa-miR-34c-3p | miRWalk |
| *VEGFA* | hsa-miR-299-3p | miRWalk |
| *VEGFA* | hsa-miR-296-3p | miRWalk |
| *VEGFA* | hsa-miR-130b-5p | miRWalk |
| *VEGFA* | hsa-miR-302b-3p | miRWalk |
| *VEGFA* | hsa-miR-302c-3p | miRWalk |
| *VEGFA* | hsa-miR-302d-3p | miRWalk |
| *VEGFA* | hsa-miR-377-5p | miRWalk |
| *VEGFA* | hsa-miR-378a-5p | miRWalk |
| *VEGFA* | hsa-miR-380-5p | miRWalk |
| *VEGFA* | hsa-miR-381-5p | miRWalk |
| *VEGFA* | hsa-miR-383-5p | miRWalk |
| *VEGFA* | hsa-miR-328-3p | miRWalk |
| *VEGFA* | hsa-miR-345-5p | miRWalk |
| *VEGFA* | hsa-miR-422a | miRWalk |
| *VEGFA* | hsa-miR-423-5p | miRWalk |
| *VEGFA* | hsa-miR-18b-5p | miRWalk |
| *VEGFA* | hsa-miR-449a | miRWalk |
| *VEGFA* | hsa-miR-329-5p | miRWalk |
| *VEGFA* | hsa-miR-409-5p | miRWalk |
| *VEGFA* | hsa-miR-483-3p | miRWalk |
| *VEGFA* | hsa-miR-484 | miRWalk |
| *VEGFA* | hsa-miR-486-5p | miRWalk |
| *VEGFA* | hsa-miR-486-3p | miRWalk |
| *VEGFA* | hsa-miR-490-3p | miRWalk |
| *VEGFA* | hsa-miR-432-3p | miRWalk |
| *VEGFA* | hsa-miR-494-5p | miRWalk |
| *VEGFA* | hsa-miR-193b-5p | miRWalk |
| *VEGFA* | hsa-miR-181d-3p | miRWalk |
| *VEGFA* | hsa-miR-512-5p | miRWalk |
| *VEGFA* | hsa-miR-498-5p | miRWalk |
| *VEGFA* | hsa-miR-518f-5p | miRWalk |
| *VEGFA* | hsa-miR-516a-5p | miRWalk |
| *VEGFA* | hsa-miR-501-5p | miRWalk |
| *VEGFA* | hsa-miR-503-3p | miRWalk |
| *VEGFA* | hsa-miR-504-5p | miRWalk |
| *VEGFA* | hsa-miR-505-5p | miRWalk |
| *VEGFA* | hsa-miR-508-5p | miRWalk |
| *VEGFA* | hsa-miR-455-5p | miRWalk |
| *VEGFA* | hsa-miR-487b-5p | miRWalk |
| *VEGFA* | hsa-miR-552-5p | miRWalk |
| *VEGFA* | hsa-miR-555 | miRWalk |
| *VEGFA* | hsa-miR-564 | miRWalk |
| *VEGFA* | hsa-miR-573 | miRWalk |
| *VEGFA* | hsa-miR-574-5p | miRWalk |
| *VEGFA* | hsa-miR-574-3p | miRWalk |
| *VEGFA* | hsa-miR-585-5p | miRWalk |
| *VEGFA* | hsa-miR-598-5p | miRWalk |
| *VEGFA* | hsa-miR-605-3p | miRWalk |
| *VEGFA* | hsa-miR-609 | miRWalk |
| *VEGFA* | hsa-miR-614 | miRWalk |
| *VEGFA* | hsa-miR-615-5p | miRWalk |
| *VEGFA* | hsa-miR-619-3p | miRWalk |
| *VEGFA* | hsa-miR-629-3p | miRWalk |
| *VEGFA* | hsa-miR-637 | miRWalk |
| *VEGFA* | hsa-miR-642a-5p | miRWalk |
| *VEGFA* | hsa-miR-645 | miRWalk |
| *VEGFA* | hsa-miR-646 | miRWalk |
| *VEGFA* | hsa-miR-549a-5p | miRWalk |
| *VEGFA* | hsa-miR-659-5p | miRWalk |
| *VEGFA* | hsa-miR-758-5p | miRWalk |
| *VEGFA* | hsa-miR-671-5p | miRWalk |
| *VEGFA* | hsa-miR-668-5p | miRWalk |
| *VEGFA* | hsa-miR-1224-5p | miRWalk |
| *VEGFA* | hsa-miR-1296-5p | miRWalk |
| *VEGFA* | hsa-miR-766-5p | miRWalk |
| *VEGFA* | hsa-miR-378d | miRWalk |
| *VEGFA* | hsa-miR-1185-1-3p | miRWalk |
| *VEGFA* | hsa-miR-762 | miRWalk |
| *VEGFA* | hsa-miR-765 | miRWalk |
| *VEGFA* | hsa-miR-298 | miRWalk |
| *VEGFA* | hsa-miR-450b-3p | miRWalk |
| *VEGFA* | hsa-miR-874-5p | miRWalk |
| *VEGFA* | hsa-miR-875-3p | miRWalk |
| *VEGFA* | hsa-miR-920 | miRWalk |
| *VEGFA* | hsa-miR-921 | miRWalk |
| *VEGFA* | hsa-miR-937-5p | miRWalk |
| *VEGFA* | hsa-miR-1180-5p | miRWalk |
| *VEGFA* | hsa-miR-1228-5p | miRWalk |
| *VEGFA* | hsa-miR-1231 | miRWalk |
| *VEGFA* | hsa-miR-1236-5p | miRWalk |
| *VEGFA* | hsa-miR-1289 | miRWalk |
| *VEGFA* | hsa-miR-1293 | miRWalk |
| *VEGFA* | hsa-miR-1304-5p | miRWalk |
| *VEGFA* | hsa-miR-1249-5p | miRWalk |
| *VEGFA* | hsa-miR-1251-5p | miRWalk |
| *VEGFA* | hsa-miR-1263 | miRWalk |
| *VEGFA* | hsa-miR-1266-5p | miRWalk |
| *VEGFA* | hsa-miR-1266-3p | miRWalk |
| *VEGFA* | hsa-miR-1275 | miRWalk |
| *VEGFA* | hsa-miR-1276 | miRWalk |
| *VEGFA* | hsa-miR-1288-3p | miRWalk |
| *VEGFA* | hsa-miR-664a-5p | miRWalk |
| *VEGFA* | hsa-miR-1307-3p | miRWalk |
| *VEGFA* | hsa-miR-1539 | miRWalk |
| *VEGFA* | hsa-miR-1909-5p | miRWalk |
| *VEGFA* | hsa-miR-1909-3p | miRWalk |
| *VEGFA* | hsa-miR-1910-3p | miRWalk |
| *VEGFA* | hsa-miR-1914-5p | miRWalk |
| *VEGFA* | hsa-miR-1914-3p | miRWalk |
| *VEGFA* | hsa-miR-2110 | miRWalk |
| *VEGFA* | hsa-miR-2116-3p | miRWalk |
| *VEGFA* | hsa-miR-2117 | miRWalk |
| *VEGFA* | hsa-miR-2276-3p | miRWalk |
| *VEGFA* | hsa-miR-2682-5p | miRWalk |
| *VEGFA* | hsa-miR-711 | miRWalk |
| *VEGFA* | hsa-miR-3126-5p | miRWalk |
| *VEGFA* | hsa-miR-3127-5p | miRWalk |
| *VEGFA* | hsa-miR-3130-3p | miRWalk |
| *VEGFA* | hsa-miR-3132 | miRWalk |
| *VEGFA* | hsa-miR-378b | miRWalk |
| *VEGFA* | hsa-miR-466 | miRWalk |
| *VEGFA* | hsa-miR-3140-3p | miRWalk |
| *VEGFA* | hsa-miR-3141 | miRWalk |
| *VEGFA* | hsa-miR-3150a-3p | miRWalk |
| *VEGFA* | hsa-miR-3151-5p | miRWalk |
| *VEGFA* | hsa-miR-3153 | miRWalk |
| *VEGFA* | hsa-miR-3154 | miRWalk |
| *VEGFA* | hsa-miR-3158-5p | miRWalk |
| *VEGFA* | hsa-miR-3160-3p | miRWalk |
| *VEGFA* | hsa-miR-3162-5p | miRWalk |
| *VEGFA* | hsa-miR-3173-5p | miRWalk |
| *VEGFA* | hsa-miR-3181 | miRWalk |
| *VEGFA* | hsa-miR-3186-5p | miRWalk |
| *VEGFA* | hsa-miR-3189-5p | miRWalk |
| *VEGFA* | hsa-miR-3193 | miRWalk |
| *VEGFA* | hsa-miR-3194-5p | miRWalk |
| *VEGFA* | hsa-miR-3194-3p | miRWalk |
| *VEGFA* | hsa-miR-3202 | miRWalk |
| *VEGFA* | hsa-miR-4298 | miRWalk |
| *VEGFA* | hsa-miR-4322 | miRWalk |
| *VEGFA* | hsa-miR-4257 | miRWalk |
| *VEGFA* | hsa-miR-4253 | miRWalk |
| *VEGFA* | hsa-miR-4254 | miRWalk |
| *VEGFA* | hsa-miR-4265 | miRWalk |
| *VEGFA* | hsa-miR-4268 | miRWalk |
| *VEGFA* | hsa-miR-4263 | miRWalk |
| *VEGFA* | hsa-miR-4271 | miRWalk |
| *VEGFA* | hsa-miR-4278 | miRWalk |
| *VEGFA* | hsa-miR-4285 | miRWalk |
| *VEGFA* | hsa-miR-4286 | miRWalk |
| *VEGFA* | hsa-miR-4289 | miRWalk |
| *VEGFA* | hsa-miR-500b-3p | miRWalk |
| *VEGFA* | hsa-miR-3605-3p | miRWalk |
| *VEGFA* | hsa-miR-3606-5p | miRWalk |
| *VEGFA* | hsa-miR-3619-3p | miRWalk |
| *VEGFA* | hsa-miR-3622a-5p | miRWalk |
| *VEGFA* | hsa-miR-3650 | miRWalk |
| *VEGFA* | hsa-miR-3652 | miRWalk |
| *VEGFA* | hsa-miR-3661 | miRWalk |
| *VEGFA* | hsa-miR-3662 | miRWalk |
| *VEGFA* | hsa-miR-3664-3p | miRWalk |
| *VEGFA* | hsa-miR-3665 | miRWalk |
| *VEGFA* | hsa-miR-3679-5p | miRWalk |
| *VEGFA* | hsa-miR-3680-3p | miRWalk |
| *VEGFA* | hsa-miR-3683 | miRWalk |
| *VEGFA* | hsa-miR-3689a-5p | miRWalk |
| *VEGFA* | hsa-miR-3713 | miRWalk |
| *VEGFA* | hsa-miR-3714 | miRWalk |
| *VEGFA* | hsa-miR-3689b-5p | miRWalk |
| *VEGFA* | hsa-miR-3918 | miRWalk |
| *VEGFA* | hsa-miR-3150b-3p | miRWalk |
| *VEGFA* | hsa-miR-3922-5p | miRWalk |
| *VEGFA* | hsa-miR-3934-3p | miRWalk |
| *VEGFA* | hsa-miR-3941 | miRWalk |
| *VEGFA* | hsa-miR-550b-2-5p | miRWalk |
| *VEGFA* | hsa-miR-1268b | miRWalk |
| *VEGFA* | hsa-miR-4420 | miRWalk |
| *VEGFA* | hsa-miR-4430 | miRWalk |
| *VEGFA* | hsa-miR-4441 | miRWalk |
| *VEGFA* | hsa-miR-4444 | miRWalk |
| *VEGFA* | hsa-miR-4448 | miRWalk |
| *VEGFA* | hsa-miR-4467 | miRWalk |
| *VEGFA* | hsa-miR-4476 | miRWalk |
| *VEGFA* | hsa-miR-3689e | miRWalk |
| *VEGFA* | hsa-miR-3689f | miRWalk |
| *VEGFA* | hsa-miR-3155b | miRWalk |
| *VEGFA* | hsa-miR-4480 | miRWalk |
| *VEGFA* | hsa-miR-4481 | miRWalk |
| *VEGFA* | hsa-miR-4489 | miRWalk |
| *VEGFA* | hsa-miR-4502 | miRWalk |
| *VEGFA* | hsa-miR-4505 | miRWalk |
| *VEGFA* | hsa-miR-2392 | miRWalk |
| *VEGFA* | hsa-miR-4510 | miRWalk |
| *VEGFA* | hsa-miR-4516 | miRWalk |
| *VEGFA* | hsa-miR-4523 | miRWalk |
| *VEGFA* | hsa-miR-4529-5p | miRWalk |
| *VEGFA* | hsa-miR-4534 | miRWalk |
| *VEGFA* | hsa-miR-378i | miRWalk |
| *VEGFA* | hsa-miR-3973 | miRWalk |
| *VEGFA* | hsa-miR-3976 | miRWalk |
| *VEGFA* | hsa-miR-4632-5p | miRWalk |
| *VEGFA* | hsa-miR-4633-5p | miRWalk |
| *VEGFA* | hsa-miR-4638-5p | miRWalk |
| *VEGFA* | hsa-miR-4644 | miRWalk |
| *VEGFA* | hsa-miR-4646-5p | miRWalk |
| *VEGFA* | hsa-miR-4654 | miRWalk |
| *VEGFA* | hsa-miR-4663 | miRWalk |
| *VEGFA* | hsa-miR-4667-5p | miRWalk |
| *VEGFA* | hsa-miR-4667-3p | miRWalk |
| *VEGFA* | hsa-miR-4669 | miRWalk |
| *VEGFA* | hsa-miR-4672 | miRWalk |
| *VEGFA* | hsa-miR-4673 | miRWalk |
| *VEGFA* | hsa-miR-4675 | miRWalk |
| *VEGFA* | hsa-miR-4676-5p | miRWalk |
| *VEGFA* | hsa-miR-4682 | miRWalk |
| *VEGFA* | hsa-miR-4687-3p | miRWalk |
| *VEGFA* | hsa-miR-4692 | miRWalk |
| *VEGFA* | hsa-miR-4700-5p | miRWalk |
| *VEGFA* | hsa-miR-4710 | miRWalk |
| *VEGFA* | hsa-miR-4712-5p | miRWalk |
| *VEGFA* | hsa-miR-4712-3p | miRWalk |
| *VEGFA* | hsa-miR-4717-3p | miRWalk |
| *VEGFA* | hsa-miR-4725-3p | miRWalk |
| *VEGFA* | hsa-miR-4727-5p | miRWalk |
| *VEGFA* | hsa-miR-4731-5p | miRWalk |
| *VEGFA* | hsa-miR-4732-3p | miRWalk |
| *VEGFA* | hsa-miR-4739 | miRWalk |
| *VEGFA* | hsa-miR-4743-5p | miRWalk |
| *VEGFA* | hsa-miR-4743-3p | miRWalk |
| *VEGFA* | hsa-miR-4746-5p | miRWalk |
| *VEGFA* | hsa-miR-4746-3p | miRWalk |
| *VEGFA* | hsa-miR-4748 | miRWalk |
| *VEGFA* | hsa-miR-4750-5p | miRWalk |
| *VEGFA* | hsa-miR-4753-3p | miRWalk |
| *VEGFA* | hsa-miR-4754 | miRWalk |
| *VEGFA* | hsa-miR-4758-5p | miRWalk |
| *VEGFA* | hsa-miR-4764-5p | miRWalk |
| *VEGFA* | hsa-miR-4769-5p | miRWalk |
| *VEGFA* | hsa-miR-4776-5p | miRWalk |
| *VEGFA* | hsa-miR-4778-3p | miRWalk |
| *VEGFA* | hsa-miR-4436b-3p | miRWalk |
| *VEGFA* | hsa-miR-4783-3p | miRWalk |
| *VEGFA* | hsa-miR-2467-3p | miRWalk |
| *VEGFA* | hsa-miR-4787-5p | miRWalk |
| *VEGFA* | hsa-miR-4793-5p | miRWalk |
| *VEGFA* | hsa-miR-4799-3p | miRWalk |
| *VEGFA* | hsa-miR-4804-5p | miRWalk |
| *VEGFA* | hsa-miR-5001-3p | miRWalk |
| *VEGFA* | hsa-miR-5004-3p | miRWalk |
| *VEGFA* | hsa-miR-5010-5p | miRWalk |
| *VEGFA* | hsa-miR-5011-5p | miRWalk |
| *VEGFA* | hsa-miR-5088-5p | miRWalk |
| *VEGFA* | hsa-miR-5088-3p | miRWalk |
| *VEGFA* | hsa-miR-5090 | miRWalk |
| *VEGFA* | hsa-miR-5189-5p | miRWalk |
| *VEGFA* | hsa-miR-5193 | miRWalk |
| *VEGFA* | hsa-miR-5195-5p | miRWalk |
| *VEGFA* | hsa-miR-5196-5p | miRWalk |
| *VEGFA* | hsa-miR-5571-3p | miRWalk |
| *VEGFA* | hsa-miR-5572 | miRWalk |
| *VEGFA* | hsa-miR-5587-3p | miRWalk |
| *VEGFA* | hsa-miR-5588-5p | miRWalk |
| *VEGFA* | hsa-miR-5589-5p | miRWalk |
| *VEGFA* | hsa-miR-5694 | miRWalk |
| *VEGFA* | hsa-miR-5698 | miRWalk |
| *VEGFA* | hsa-miR-5708 | miRWalk |
| *VEGFA* | hsa-miR-5739 | miRWalk |
| *VEGFA* | hsa-miR-1199-5p | miRWalk |
| *VEGFA* | hsa-miR-1199-3p | miRWalk |
| *VEGFA* | hsa-miR-6072 | miRWalk |
| *VEGFA* | hsa-miR-6081 | miRWalk |
| *VEGFA* | hsa-miR-6124 | miRWalk |
| *VEGFA* | hsa-miR-6125 | miRWalk |
| *VEGFA* | hsa-miR-6127 | miRWalk |
| *VEGFA* | hsa-miR-6130 | miRWalk |
| *VEGFA* | hsa-miR-6132 | miRWalk |
| *VEGFA* | hsa-miR-6133 | miRWalk |
| *VEGFA* | hsa-miR-6134 | miRWalk |
| *VEGFA* | hsa-miR-6165 | miRWalk |
| *VEGFA* | hsa-miR-6500-3p | miRWalk |
| *VEGFA* | hsa-miR-6501-5p | miRWalk |
| *VEGFA* | hsa-miR-6505-3p | miRWalk |
| *VEGFA* | hsa-miR-6509-5p | miRWalk |
| *VEGFA* | hsa-miR-6510-5p | miRWalk |
| *VEGFA* | hsa-miR-6510-3p | miRWalk |
| *VEGFA* | hsa-miR-6515-3p | miRWalk |
| *VEGFA* | hsa-miR-6715b-3p | miRWalk |
| *VEGFA* | hsa-miR-6716-3p | miRWalk |
| *VEGFA* | hsa-miR-6717-5p | miRWalk |
| *VEGFA* | hsa-miR-6720-5p | miRWalk |
| *VEGFA* | hsa-miR-6720-3p | miRWalk |
| *VEGFA* | hsa-miR-892c-3p | miRWalk |
| *VEGFA* | hsa-miR-6726-5p | miRWalk |
| *VEGFA* | hsa-miR-6728-3p | miRWalk |
| *VEGFA* | hsa-miR-6729-5p | miRWalk |
| *VEGFA* | hsa-miR-6729-3p | miRWalk |
| *VEGFA* | hsa-miR-6730-5p | miRWalk |
| *VEGFA* | hsa-miR-6735-5p | miRWalk |
| *VEGFA* | hsa-miR-6736-5p | miRWalk |
| *VEGFA* | hsa-miR-6736-3p | miRWalk |
| *VEGFA* | hsa-miR-6738-5p | miRWalk |
| *VEGFA* | hsa-miR-6739-3p | miRWalk |
| *VEGFA* | hsa-miR-6740-5p | miRWalk |
| *VEGFA* | hsa-miR-6743-5p | miRWalk |
| *VEGFA* | hsa-miR-6746-5p | miRWalk |
| *VEGFA* | hsa-miR-6747-3p | miRWalk |
| *VEGFA* | hsa-miR-6748-5p | miRWalk |
| *VEGFA* | hsa-miR-6748-3p | miRWalk |
| *VEGFA* | hsa-miR-6751-5p | miRWalk |
| *VEGFA* | hsa-miR-6753-5p | miRWalk |
| *VEGFA* | hsa-miR-6753-3p | miRWalk |
| *VEGFA* | hsa-miR-6756-3p | miRWalk |
| *VEGFA* | hsa-miR-6757-5p | miRWalk |
| *VEGFA* | hsa-miR-6758-5p | miRWalk |
| *VEGFA* | hsa-miR-6758-3p | miRWalk |
| *VEGFA* | hsa-miR-6759-5p | miRWalk |
| *VEGFA* | hsa-miR-6760-5p | miRWalk |
| *VEGFA* | hsa-miR-6761-5p | miRWalk |
| *VEGFA* | hsa-miR-6764-5p | miRWalk |
| *VEGFA* | hsa-miR-6770-5p | miRWalk |
| *VEGFA* | hsa-miR-6772-5p | miRWalk |
| *VEGFA* | hsa-miR-6779-5p | miRWalk |
| *VEGFA* | hsa-miR-6779-3p | miRWalk |
| *VEGFA* | hsa-miR-6783-3p | miRWalk |
| *VEGFA* | hsa-miR-6785-5p | miRWalk |
| *VEGFA* | hsa-miR-6792-3p | miRWalk |
| *VEGFA* | hsa-miR-6793-5p | miRWalk |
| *VEGFA* | hsa-miR-6795-5p | miRWalk |
| *VEGFA* | hsa-miR-6795-3p | miRWalk |
| *VEGFA* | hsa-miR-6796-5p | miRWalk |
| *VEGFA* | hsa-miR-6797-5p | miRWalk |
| *VEGFA* | hsa-miR-6798-5p | miRWalk |
| *VEGFA* | hsa-miR-6799-5p | miRWalk |
| *VEGFA* | hsa-miR-6800-5p | miRWalk |
| *VEGFA* | hsa-miR-6809-3p | miRWalk |
| *VEGFA* | hsa-miR-6810-3p | miRWalk |
| *VEGFA* | hsa-miR-6812-5p | miRWalk |
| *VEGFA* | hsa-miR-6813-3p | miRWalk |
| *VEGFA* | hsa-miR-6818-5p | miRWalk |
| *VEGFA* | hsa-miR-6818-3p | miRWalk |
| *VEGFA* | hsa-miR-6819-5p | miRWalk |
| *VEGFA* | hsa-miR-6819-3p | miRWalk |
| *VEGFA* | hsa-miR-6821-5p | miRWalk |
| *VEGFA* | hsa-miR-6823-3p | miRWalk |
| *VEGFA* | hsa-miR-6824-5p | miRWalk |
| *VEGFA* | hsa-miR-6824-3p | miRWalk |
| *VEGFA* | hsa-miR-6825-5p | miRWalk |
| *VEGFA* | hsa-miR-6827-5p | miRWalk |
| *VEGFA* | hsa-miR-6829-5p | miRWalk |
| *VEGFA* | hsa-miR-6830-5p | miRWalk |
| *VEGFA* | hsa-miR-6831-5p | miRWalk |
| *VEGFA* | hsa-miR-6834-3p | miRWalk |
| *VEGFA* | hsa-miR-6835-5p | miRWalk |
| *VEGFA* | hsa-miR-6780b-5p | miRWalk |
| *VEGFA* | hsa-miR-6839-5p | miRWalk |
| *VEGFA* | hsa-miR-6840-3p | miRWalk |
| *VEGFA* | hsa-miR-6846-5p | miRWalk |
| *VEGFA* | hsa-miR-6848-5p | miRWalk |
| *VEGFA* | hsa-miR-6848-3p | miRWalk |
| *VEGFA* | hsa-miR-6849-5p | miRWalk |
| *VEGFA* | hsa-miR-6853-3p | miRWalk |
| *VEGFA* | hsa-miR-6856-5p | miRWalk |
| *VEGFA* | hsa-miR-6858-5p | miRWalk |
| *VEGFA* | hsa-miR-6769b-3p | miRWalk |
| *VEGFA* | hsa-miR-6860 | miRWalk |
| *VEGFA* | hsa-miR-6862-5p | miRWalk |
| *VEGFA* | hsa-miR-6864-3p | miRWalk |
| *VEGFA* | hsa-miR-6871-3p | miRWalk |
| *VEGFA* | hsa-miR-6873-3p | miRWalk |
| *VEGFA* | hsa-miR-6874-3p | miRWalk |
| *VEGFA* | hsa-miR-6875-5p | miRWalk |
| *VEGFA* | hsa-miR-6876-3p | miRWalk |
| *VEGFA* | hsa-miR-6883-5p | miRWalk |
| *VEGFA* | hsa-miR-6884-5p | miRWalk |
| *VEGFA* | hsa-miR-6886-5p | miRWalk |
| *VEGFA* | hsa-miR-6888-3p | miRWalk |
| *VEGFA* | hsa-miR-6890-5p | miRWalk |
| *VEGFA* | hsa-miR-6892-5p | miRWalk |
| *VEGFA* | hsa-miR-6893-5p | miRWalk |
| *VEGFA* | hsa-miR-6894-5p | miRWalk |
| *VEGFA* | hsa-miR-6895-3p | miRWalk |
| *VEGFA* | hsa-miR-7106-3p | miRWalk |
| *VEGFA* | hsa-miR-7107-5p | miRWalk |
| *VEGFA* | hsa-miR-7107-3p | miRWalk |
| *VEGFA* | hsa-miR-7110-5p | miRWalk |
| *VEGFA* | hsa-miR-7111-5p | miRWalk |
| *VEGFA* | hsa-miR-7112-3p | miRWalk |
| *VEGFA* | hsa-miR-7150 | miRWalk |
| *VEGFA* | hsa-miR-7161-5p | miRWalk |
| *VEGFA* | hsa-miR-7160-3p | miRWalk |
| *VEGFA* | hsa-miR-7162-5p | miRWalk |
| *VEGFA* | hsa-miR-7702 | miRWalk |
| *VEGFA* | hsa-miR-7706 | miRWalk |
| *VEGFA* | hsa-miR-4433b-3p | miRWalk |
| *VEGFA* | hsa-miR-8058 | miRWalk |
| *VEGFA* | hsa-miR-8071 | miRWalk |
| *VEGFA* | hsa-miR-8078 | miRWalk |
| *VEGFA* | hsa-miR-8085 | miRWalk |
| *VEGFA* | hsa-miR-8485 | miRWalk |
| *VEGFA* | hsa-miR-9718 | miRWalk |
| *VEGFA* | hsa-miR-9899 | miRWalk |
| *VEGFA* | hsa-miR-10392-5p | miRWalk |
| *VEGFA* | hsa-miR-10396a-5p | miRWalk |
| *VEGFA* | hsa-miR-10398-5p | miRWalk |
| *VEGFA* | hsa-miR-10398-3p | miRWalk |
| *VEGFA* | hsa-miR-10401-5p | miRWalk |
| *VEGFA* | hsa-miR-10396b-5p | miRWalk |
| *VEGFA* | hsa-miR-10396b-3p | miRWalk |
| *VEGFA* | hsa-miR-11400 | miRWalk |
| *VEGFA* | hsa-miR-3059-5p | miRWalk |
| *VEGFA* | hsa-miR-9851-5p | miRWalk |
| *VEGFA* | hsa-miR-12118 | miRWalk |
| *VEGFA* | hsa-miR-12120 | miRWalk |
| *VEGFA* | hsa-miR-12124 | miRWalk |
| *VEGFA* | hsa-miR-12127 | miRWalk |
| *VEGFA* | hsa-miR-12130 | miRWalk |
| *VEGFA* | hsa-miR-12133 | miRWalk |
| *VEGFA* | hsa-miR-23b-3p | miRWalk |
| *VEGFA* | hsa-miR-346 | miRWalk |
| *VEGFA* | hsa-miR-584-5p | miRWalk |
| *VEGFA* | hsa-miR-320b | miRWalk |
| *VEGFA* | hsa-miR-1304-3p | miRWalk |
| *VEGFA* | hsa-miR-2115-5p | miRWalk |
| *VEGFA* | hsa-miR-3177-3p | miRWalk |
| *VEGFA* | hsa-miR-4329 | miRWalk |
| *VEGFA* | hsa-miR-4330 | miRWalk |
| *VEGFA* | hsa-miR-4653-3p | miRWalk |
| *VEGFA* | hsa-miR-4728-3p | miRWalk |
| *VEGFA* | hsa-miR-2467-5p | miRWalk |
| *VEGFA* | hsa-miR-6859-3p | miRWalk |
| *VEGFA* | hsa-miR-6861-5p | miRWalk |
| *VEGFA* | hsa-miR-6888-5p | miRWalk |
| *VEGFA* | hsa-miR-7151-3p | miRWalk |
| *VEGFA* | hsa-miR-3074-3p | miRWalk |
| *VEGFA* | hsa-miR-4433a-3p | miRWalk |
| *VEGFA* | hsa-miR-7854-3p | miRWalk |
| *VEGFA* | hsa-miR-596 | miRWalk |
| *VEGFA* | hsa-miR-4788 | miRWalk |
| *VEGFA* | hsa-miR-5002-5p | miRWalk |
| *VEGFA* | hsa-miR-6852-5p | miRWalk |
| *VEGFA* | hsa-miR-127-5p | miRWalk |
| *VEGFA* | hsa-miR-4653-5p | miRWalk |
| *VEGFA* | hsa-miR-659-3p | miRWalk |
| *VEGFA* | hsa-miR-4442 | miRWalk |
| *VEGFA* | hsa-let-7c-5p | miRWalk |
| *VEGFA* | hsa-miR-16-5p | miRWalk |
| *VEGFA* | hsa-miR-18a-5p | miRWalk |
| *VEGFA* | hsa-miR-20a-5p | miRWalk |
| *VEGFA* | hsa-miR-29b-2-5p | miRWalk |
| *VEGFA* | hsa-miR-125a-3p | miRWalk |
| *VEGFA* | hsa-miR-150-5p | miRWalk |
| *VEGFA* | hsa-miR-194-3p | miRWalk |
| *VEGFA* | hsa-miR-376a-5p | miRWalk |
| *VEGFA* | hsa-miR-382-3p | miRWalk |
| *VEGFA* | hsa-miR-151a-3p | miRWalk |
| *VEGFA* | hsa-miR-488-5p | miRWalk |
| *VEGFA* | hsa-miR-491-5p | miRWalk |
| *VEGFA* | hsa-miR-511-5p | miRWalk |
| *VEGFA* | hsa-miR-520a-3p | miRWalk |
| *VEGFA* | hsa-miR-526b-5p | miRWalk |
| *VEGFA* | hsa-miR-518c-5p | miRWalk |
| *VEGFA* | hsa-miR-450a-2-3p | miRWalk |
| *VEGFA* | hsa-miR-583 | miRWalk |
| *VEGFA* | hsa-miR-595 | miRWalk |
| *VEGFA* | hsa-miR-610 | miRWalk |
| *VEGFA* | hsa-miR-1271-5p | miRWalk |
| *VEGFA* | hsa-miR-761 | miRWalk |
| *VEGFA* | hsa-miR-942-5p | miRWalk |
| *VEGFA* | hsa-miR-1178-3p | miRWalk |
| *VEGFA* | hsa-miR-1233-5p | miRWalk |
| *VEGFA* | hsa-miR-1207-5p | miRWalk |
| *VEGFA* | hsa-miR-1277-5p | miRWalk |
| *VEGFA* | hsa-miR-1281 | miRWalk |
| *VEGFA* | hsa-miR-1912-3p | miRWalk |
| *VEGFA* | hsa-miR-3126-3p | miRWalk |
| *VEGFA* | hsa-miR-3147 | miRWalk |
| *VEGFA* | hsa-miR-3155a | miRWalk |
| *VEGFA* | hsa-miR-3157-5p | miRWalk |
| *VEGFA* | hsa-miR-3175 | miRWalk |
| *VEGFA* | hsa-miR-3188 | miRWalk |
| *VEGFA* | hsa-miR-4324 | miRWalk |
| *VEGFA* | hsa-miR-2355-5p | miRWalk |
| *VEGFA* | hsa-miR-4270 | miRWalk |
| *VEGFA* | hsa-miR-3616-3p | miRWalk |
| *VEGFA* | hsa-miR-3690 | miRWalk |
| *VEGFA* | hsa-miR-3691-3p | miRWalk |
| *VEGFA* | hsa-miR-3937 | miRWalk |
| *VEGFA* | hsa-miR-4423-5p | miRWalk |
| *VEGFA* | hsa-miR-4439 | miRWalk |
| *VEGFA* | hsa-miR-4490 | miRWalk |
| *VEGFA* | hsa-miR-4530 | miRWalk |
| *VEGFA* | hsa-miR-4661-3p | miRWalk |
| *VEGFA* | hsa-miR-4684-3p | miRWalk |
| *VEGFA* | hsa-miR-4691-3p | miRWalk |
| *VEGFA* | hsa-miR-4722-5p | miRWalk |
| *VEGFA* | hsa-miR-4774-3p | miRWalk |
| *VEGFA* | hsa-miR-4784 | miRWalk |
| *VEGFA* | hsa-miR-4796-3p | miRWalk |
| *VEGFA* | hsa-miR-5187-5p | miRWalk |
| *VEGFA* | hsa-miR-5195-3p | miRWalk |
| *VEGFA* | hsa-miR-664b-5p | miRWalk |
| *VEGFA* | hsa-miR-5589-3p | miRWalk |
| *VEGFA* | hsa-miR-6074 | miRWalk |
| *VEGFA* | hsa-miR-6088 | miRWalk |
| *VEGFA* | hsa-miR-6499-5p | miRWalk |
| *VEGFA* | hsa-miR-6745 | miRWalk |
| *VEGFA* | hsa-miR-6754-5p | miRWalk |
| *VEGFA* | hsa-miR-6763-5p | miRWalk |
| *VEGFA* | hsa-miR-6764-3p | miRWalk |
| *VEGFA* | hsa-miR-6777-5p | miRWalk |
| *VEGFA* | hsa-miR-6792-5p | miRWalk |
| *VEGFA* | hsa-miR-6807-5p | miRWalk |
| *VEGFA* | hsa-miR-6814-5p | miRWalk |
| *VEGFA* | hsa-miR-6780b-3p | miRWalk |
| *VEGFA* | hsa-miR-6837-3p | miRWalk |
| *VEGFA* | hsa-miR-6891-5p | miRWalk |
| *VEGFA* | hsa-miR-7114-5p | miRWalk |
| *VEGFA* | hsa-miR-7156-5p | miRWalk |
| *VEGFA* | hsa-miR-1273h-5p | miRWalk |
| *VEGFA* | hsa-miR-10522-5p | miRWalk |
| *VEGFA* | hsa-miR-11399 | miRWalk |
| *VEGFA* | hsa-miR-12119 | miRWalk |
