# Supplementary material for: Integrated bioinformatics analysis for the identification of idiopathic pulmonary fibrosis–related genes and potential therapeutic drugs
Source: BMC Pulm Med. 2023 Oct 4;23:373. doi: 10.1186/s12890-023-02678-z (PMC10552267; doi:10.1186/s12890-023-02678-z)
Supplement: Supplementary file 1 — Additional file 1: Table S1. The analyze network results of 1640 DEGs. Table S2. GO terms of the 18 hub genes. Table S3. KEGG pathways of the 18 hub genes. Table S4. Target microRNAs of SPP1 based on five online miRNA databases. Table S5. Target microRNAs of VEGFA based on five online miRNA databases. Table S6. Target microRNAs of COL1A1 based on five online miRNA databases. Table S7. Target microRNAs of CAV1 based on five online miRNA databases. Table S8. Target microRNAs of PECAM1 based on five online miRNA databases. Table S9. Target microRNAs of BMP4 based on five online miRNA databases. Table S10. Target microRNAs of FYN based on five online miRNA databases. Table S11. Traditional Chinese medicine prediction results of COL1A1. Table S12. Traditional Chinese medicine prediction results of VEGFA. Table S13. Traditional Chinese medicine prediction results of SPP1. [file 12890_2023_2678_MOESM1_ESM.zip › Supplementary Tables/Supplementary Table10.docx]

**Table S10 Target microRNAs of *FYN* based on five online miRNA databases**

| Gene Symbol | microRNA | Database |
| --- | --- | --- |
| *FYN* | hsa-miR-369-3p | mirDIP |
| *FYN* | hsa-miR-125a-3p | mirDIP |
| *FYN* | hsa-miR-583 | mirDIP |
| *FYN* | hsa-miR-4717-5p | mirDIP |
| *FYN* | hsa-miR-1226-5p | mirDIP |
| *FYN* | hsa-miR-4257 | mirDIP |
| *FYN* | hsa-miR-4797-5p | mirDIP |
| *FYN* | hsa-miR-616-3p | mirDIP |
| *FYN* | hsa-miR-4748 | mirDIP |
| *FYN* | hsa-miR-4260 | mirDIP |
| *FYN* | hsa-miR-200b-3p | ENCORI |
| *FYN* | hsa-miR-200a-3p | ENCORI |
| *FYN* | hsa-miR-429 | ENCORI |
| *FYN* | hsa-miR-186-5p | ENCORI |
| *FYN* | hsa-miR-137 | ENCORI |
| *FYN* | hsa-miR-29c-3p | ENCORI |
| *FYN* | hsa-miR-29b-3p | ENCORI |
| *FYN* | hsa-miR-4295 | ENCORI |
| *FYN* | hsa-miR-302e | ENCORI |
| *FYN* | hsa-miR-129-5p | ENCORI |
| *FYN* | hsa-miR-200c-3p | ENCORI |
| *FYN* | hsa-miR-141-3p | ENCORI |
| *FYN* | hsa-miR-17-5p | ENCORI |
| *FYN* | hsa-miR-20a-5p | ENCORI |
| *FYN* | hsa-miR-432-5p | ENCORI |
| *FYN* | hsa-miR-494-3p | ENCORI |
| *FYN* | hsa-miR-539-5p | ENCORI |
| *FYN* | hsa-miR-410-3p | ENCORI |
| *FYN* | hsa-miR-203a | ENCORI |
| *FYN* | hsa-miR-7-5p | ENCORI |
| *FYN* | hsa-miR-1 | ENCORI |
| *FYN* | hsa-miR-27a-3p | ENCORI |
| *FYN* | hsa-miR-23a-3p | ENCORI |
| *FYN* | hsa-miR-125a-3p | ENCORI |
| *FYN* | hsa-miR-520f-3p | ENCORI |
| *FYN* | hsa-miR-526b-3p | ENCORI |
| *FYN* | hsa-miR-520b | ENCORI |
| *FYN* | hsa-miR-520c-3p | ENCORI |
| *FYN* | hsa-miR-520d-3p | ENCORI |
| *FYN* | hsa-miR-153-3p | ENCORI |
| *FYN* | hsa-miR-155-5p | ENCORI |
| *FYN* | hsa-miR-1271-5p | ENCORI |
| *FYN* | hsa-miR-206 | ENCORI |
| *FYN* | hsa-miR-590-3p | ENCORI |
| *FYN* | hsa-miR-653-5p | ENCORI |
| *FYN* | hsa-miR-93-5p | ENCORI |
| *FYN* | hsa-miR-106b-5p | ENCORI |
| *FYN* | hsa-miR-182-5p | ENCORI |
| *FYN* | hsa-miR-96-5p | ENCORI |
| *FYN* | hsa-miR-183-5p | ENCORI |
| *FYN* | hsa-miR-29a-3p | ENCORI |
| *FYN* | hsa-miR-23b-3p | ENCORI |
| *FYN* | hsa-miR-27b-3p | ENCORI |
| *FYN* | hsa-miR-374b-5p | ENCORI |
| *FYN* | hsa-miR-374a-5p | ENCORI |
| *FYN* | hsa-miR-20b-5p | ENCORI |
| *FYN* | hsa-miR-106a-5p | ENCORI |
| *FYN* | hsa-miR-513a-5p | ENCORI |
| *FYN* | hsa-miR-183-5p.2 | TargetScan |
| *FYN* | hsa-miR-96-5p | TargetScan |
| *FYN* | hsa-miR-1271-5p | TargetScan |
| *FYN* | hsa-miR-6835-3p | TargetScan |
| *FYN* | hsa-miR-33b-5p | TargetScan |
| *FYN* | hsa-miR-33a-5p | TargetScan |
| *FYN* | hsa-miR-200b-3p | TargetScan |
| *FYN* | hsa-miR-429 | TargetScan |
| *FYN* | hsa-miR-200c-3p | TargetScan |
| *FYN* | hsa-miR-369-3p | TargetScan |
| *FYN* | hsa-miR-130b-3p | TargetScan |
| *FYN* | hsa-miR-130a-3p | TargetScan |
| *FYN* | hsa-miR-3666 | TargetScan |
| *FYN* | hsa-miR-454-3p | TargetScan |
| *FYN* | hsa-miR-301a-3p | TargetScan |
| *FYN* | hsa-miR-4295 | TargetScan |
| *FYN* | hsa-miR-301b-3p | TargetScan |
| *FYN* | hsa-miR-20a-5p | TargetScan |
| *FYN* | hsa-miR-106b-5p | TargetScan |
| *FYN* | hsa-miR-20b-5p | TargetScan |
| *FYN* | hsa-miR-106a-5p | TargetScan |
| *FYN* | hsa-miR-93-5p | TargetScan |
| *FYN* | hsa-miR-17-5p | TargetScan |
| *FYN* | hsa-miR-519d-3p | TargetScan |
| *FYN* | hsa-miR-526b-3p | TargetScan |
| *FYN* | hsa-miR-5195-3p | TargetScan |
| *FYN* | hsa-miR-145-5p | TargetScan |
| *FYN* | hsa-miR-381-3p | TargetScan |
| *FYN* | hsa-miR-300 | TargetScan |
| *FYN* | hsa-miR-153-3p | TargetScan |
| *FYN* | hsa-miR-203a-3p.1 | TargetScan |
| *FYN* | hsa-miR-324-5p | TargetScan |
| *FYN* | hsa-miR-499a-5p | TargetScan |
| *FYN* | hsa-miR-30c-5p | TargetScan |
| *FYN* | hsa-miR-30b-5p | TargetScan |
| *FYN* | hsa-miR-30a-5p | TargetScan |
| *FYN* | hsa-miR-30d-5p | TargetScan |
| *FYN* | hsa-miR-30e-5p | TargetScan |
| *FYN* | hsa-miR-4495 | DIANA-micro T |
| *FYN* | hsa-miR-380-3p | DIANA-micro T |
| *FYN* | hsa-miR-548aj-3p | DIANA-micro T |
| *FYN* | hsa-miR-548x-3p | DIANA-micro T |
| *FYN* | hsa-miR-6810-5p | DIANA-micro T |
| *FYN* | hsa-miR-499a-5p | DIANA-micro T |
| *FYN* | hsa-miR-5688 | DIANA-micro T |
| *FYN* | hsa-miR-4671-3p | DIANA-micro T |
| *FYN* | hsa-miR-4719 | DIANA-micro T |
| *FYN* | hsa-miR-300 | DIANA-micro T |
| *FYN* | hsa-miR-381-3p | DIANA-micro T |
| *FYN* | hsa-miR-513b-3p | DIANA-micro T |
| *FYN* | hsa-miR-495-3p | DIANA-micro T |
| *FYN* | hsa-miR-6825-5p | DIANA-micro T |
| *FYN* | hsa-miR-411-3p | DIANA-micro T |
| *FYN* | hsa-miR-379-3p | DIANA-micro T |
| *FYN* | hsa-miR-548c-3p | DIANA-micro T |
| *FYN* | hsa-miR-5011-5p | DIANA-micro T |
| *FYN* | hsa-miR-3658 | DIANA-micro T |
| *FYN* | hsa-miR-548ah-3p | DIANA-micro T |
| *FYN* | hsa-miR-548am-3p | DIANA-micro T |
| *FYN* | hsa-miR-548aq-3p | DIANA-micro T |
| *FYN* | hsa-miR-548ae-3p | DIANA-micro T |
| *FYN* | hsa-miR-4797-5p | DIANA-micro T |
| *FYN* | hsa-miR-548j-3p | DIANA-micro T |
| *FYN* | hsa-miR-153-3p | DIANA-micro T |
| *FYN* | hsa-miR-5692a | DIANA-micro T |
| *FYN* | hsa-miR-3662 | DIANA-micro T |
| *FYN* | hsa-miR-7856-5p | DIANA-micro T |
| *FYN* | hsa-miR-5010-3p | DIANA-micro T |
| *FYN* | hsa-miR-16-1-3p | DIANA-micro T |
| *FYN* | hsa-miR-4728-5p | DIANA-micro T |
| *FYN* | hsa-miR-6847-5p | DIANA-micro T |
| *FYN* | hsa-miR-6835-3p | DIANA-micro T |
| *FYN* | hsa-miR-96-5p | DIANA-micro T |
| *FYN* | hsa-miR-1290 | DIANA-micro T |
| *FYN* | hsa-miR-324-5p | DIANA-micro T |
| *FYN* | hsa-miR-3159 | DIANA-micro T |
| *FYN* | hsa-miR-3613-3p | DIANA-micro T |
| *FYN* | hsa-miR-548d-3p | DIANA-micro T |
| *FYN* | hsa-miR-30c-5p | DIANA-micro T |
| *FYN* | hsa-miR-4424 | DIANA-micro T |
| *FYN* | hsa-miR-590-3p | DIANA-micro T |
| *FYN* | hsa-miR-30b-5p | DIANA-micro T |
| *FYN* | hsa-miR-6857-5p | DIANA-micro T |
| *FYN* | hsa-miR-651-3p | DIANA-micro T |
| *FYN* | hsa-miR-105-5p | DIANA-micro T |
| *FYN* | hsa-miR-6750-3p | DIANA-micro T |
| *FYN* | hsa-miR-129-5p | DIANA-micro T |
| *FYN* | hsa-miR-6871-3p | DIANA-micro T |
| *FYN* | hsa-miR-7853-5p | DIANA-micro T |
| *FYN* | hsa-miR-7-2-3p | DIANA-micro T |
| *FYN* | hsa-miR-7-1-3p | DIANA-micro T |
| *FYN* | hsa-miR-190a-3p | DIANA-micro T |
| *FYN* | hsa-miR-454-3p | DIANA-micro T |
| *FYN* | hsa-miR-548z | DIANA-micro T |
| *FYN* | hsa-miR-548h-3p | DIANA-micro T |
| *FYN* | hsa-miR-150-3p | DIANA-micro T |
| *FYN* | hsa-miR-548ac | DIANA-micro T |
| *FYN* | hsa-miR-4282 | DIANA-micro T |
| *FYN* | hsa-miR-4717-5p | DIANA-micro T |
| *FYN* | hsa-miR-4668-3p | DIANA-micro T |
| *FYN* | hsa-miR-4798-3p | DIANA-micro T |
| *FYN* | hsa-miR-5003-3p | DIANA-micro T |
| *FYN* | hsa-miR-4251 | DIANA-micro T |
| *FYN* | hsa-miR-5680 | DIANA-micro T |
| *FYN* | hsa-miR-3191-3p | DIANA-micro T |
| *FYN* | hsa-miR-548bb-3p | DIANA-micro T |
| *FYN* | hsa-miR-30a-5p | DIANA-micro T |
| *FYN* | hsa-miR-8086 | DIANA-micro T |
| *FYN* | hsa-miR-2110 | DIANA-micro T |
| *FYN* | hsa-miR-30d-5p | DIANA-micro T |
| *FYN* | hsa-miR-7153-5p | DIANA-micro T |
| *FYN* | hsa-miR-146a-5p | DIANA-micro T |
| *FYN* | hsa-miR-8087 | DIANA-micro T |
| *FYN* | hsa-miR-146b-5p | DIANA-micro T |
| *FYN* | hsa-miR-526b-3p | DIANA-micro T |
| *FYN* | hsa-miR-1343-5p | DIANA-micro T |
| *FYN* | hsa-miR-30e-5p | DIANA-micro T |
| *FYN* | hsa-miR-4721 | DIANA-micro T |
| *FYN* | hsa-miR-939-5p | DIANA-micro T |
| *FYN* | hsa-miR-369-3p | DIANA-micro T |
| *FYN* | hsa-miR-4659b-5p | DIANA-micro T |
| *FYN* | hsa-miR-3167 | DIANA-micro T |
| *FYN* | hsa-miR-450a-2-3p | DIANA-micro T |
| *FYN* | hsa-miR-211-3p | DIANA-micro T |
| *FYN* | hsa-miR-3151-3p | DIANA-micro T |
| *FYN* | hsa-miR-33b-5p | DIANA-micro T |
| *FYN* | hsa-miR-583 | DIANA-micro T |
| *FYN* | hsa-miR-5700 | DIANA-micro T |
| *FYN* | hsa-miR-4666a-3p | DIANA-micro T |
| *FYN* | hsa-miR-3143 | DIANA-micro T |
| *FYN* | hsa-miR-548at-5p | DIANA-micro T |
| *FYN* | hsa-miR-589-5p | DIANA-micro T |
| *FYN* | hsa-miR-539-5p | DIANA-micro T |
| *FYN* | hsa-miR-3977 | DIANA-micro T |
| *FYN* | hsa-miR-5682 | DIANA-micro T |
| *FYN* | hsa-miR-513c-3p | DIANA-micro T |
| *FYN* | hsa-let-7g-3p | DIANA-micro T |
| *FYN* | hsa-miR-429 | DIANA-micro T |
| *FYN* | hsa-miR-4252 | DIANA-micro T |
| *FYN* | hsa-miR-200c-3p | DIANA-micro T |
| *FYN* | hsa-miR-200b-3p | DIANA-micro T |
| *FYN* | hsa-miR-4699-3p | DIANA-micro T |
| *FYN* | hsa-miR-148a-5p | DIANA-micro T |
| *FYN* | hsa-miR-513a-3p | DIANA-micro T |
| *FYN* | hsa-miR-1271-5p | DIANA-micro T |
| *FYN* | hsa-miR-6826-5p | DIANA-micro T |
| *FYN* | hsa-miR-3150a-3p | DIANA-micro T |
| *FYN* | hsa-miR-2113 | DIANA-micro T |
| *FYN* | hsa-miR-6730-5p | DIANA-micro T |
| *FYN* | hsa-miR-1207-3p | DIANA-micro T |
| *FYN* | hsa-miR-3148 | DIANA-micro T |
| *FYN* | hsa-miR-29b-3p | DIANA-micro T |
| *FYN* | hsa-miR-147a | DIANA-micro T |
| *FYN* | hsa-miR-29c-3p | DIANA-micro T |
| *FYN* | hsa-miR-548av-5p | DIANA-micro T |
| *FYN* | hsa-miR-8060 | DIANA-micro T |
| *FYN* | hsa-miR-6839-3p | DIANA-micro T |
| *FYN* | hsa-miR-3115 | DIANA-micro T |
| *FYN* | hsa-miR-4638-3p | DIANA-micro T |
| *FYN* | hsa-miR-9-5p | DIANA-micro T |
| *FYN* | hsa-miR-2053 | DIANA-micro T |
| *FYN* | hsa-miR-29a-3p | DIANA-micro T |
| *FYN* | hsa-miR-6763-5p | DIANA-micro T |
| *FYN* | hsa-miR-624-3p | DIANA-micro T |
| *FYN* | hsa-miR-569 | DIANA-micro T |
| *FYN* | hsa-miR-4666b | DIANA-micro T |
| *FYN* | hsa-miR-661 | DIANA-micro T |
| *FYN* | hsa-miR-4753-3p | DIANA-micro T |
| *FYN* | hsa-miR-543 | DIANA-micro T |
| *FYN* | hsa-miR-4477a | DIANA-micro T |
| *FYN* | hsa-miR-6729-3p | DIANA-micro T |
| *FYN* | hsa-miR-374b-3p | DIANA-micro T |
| *FYN* | hsa-miR-4724-5p | DIANA-micro T |
| *FYN* | hsa-miR-149-3p | DIANA-micro T |
| *FYN* | hsa-miR-5681a | DIANA-micro T |
| *FYN* | hsa-miR-5582-3p | DIANA-micro T |
| *FYN* | hsa-let-7c-3p | DIANA-micro T |
| *FYN* | hsa-miR-6785-5p | DIANA-micro T |
| *FYN* | hsa-miR-6883-5p | DIANA-micro T |
| *FYN* | hsa-miR-448 | DIANA-micro T |
| *FYN* | hsa-miR-559 | DIANA-micro T |
| *FYN* | hsa-miR-6776-3p | DIANA-micro T |
| *FYN* | hsa-miR-6516-5p | DIANA-micro T |
| *FYN* | hsa-miR-33a-5p | DIANA-micro T |
| *FYN* | hsa-miR-32-3p | DIANA-micro T |
| *FYN* | hsa-miR-3591-3p | DIANA-micro T |
| *FYN* | hsa-miR-5093 | DIANA-micro T |
| *FYN* | hsa-miR-4257 | DIANA-micro T |
| *FYN* | hsa-miR-3116 | DIANA-micro T |
| *FYN* | hsa-miR-92a-1-5p | DIANA-micro T |
| *FYN* | hsa-miR-545-5p | DIANA-micro T |
| *FYN* | hsa-miR-3934-5p | DIANA-micro T |
| *FYN* | hsa-miR-6797-3p | DIANA-micro T |
| *FYN* | hsa-miR-3666 | DIANA-micro T |
| *FYN* | hsa-miR-6895-5p | DIANA-micro T |
| *FYN* | hsa-miR-6761-5p | DIANA-micro T |
| *FYN* | hsa-miR-664a-3p | DIANA-micro T |
| *FYN* | hsa-miR-3613-5p | DIANA-micro T |
| *FYN* | hsa-miR-1226-5p | DIANA-micro T |
| *FYN* | hsa-miR-656-5p | DIANA-micro T |
| *FYN* | hsa-miR-876-5p | DIANA-micro T |
| *FYN* | hsa-let-7d-3p | miRWalk |
| *FYN* | hsa-miR-488-5p | miRWalk |
| *FYN* | hsa-miR-4433b-5p | miRWalk |
| *FYN* | hsa-miR-450a-1-3p | miRWalk |
| *FYN* | hsa-miR-138-5p | miRWalk |
| *FYN* | hsa-miR-326 | miRWalk |
| *FYN* | hsa-miR-1237-3p | miRWalk |
| *FYN* | hsa-miR-4313 | miRWalk |
| *FYN* | hsa-miR-6738-3p | miRWalk |
| *FYN* | hsa-miR-6752-3p | miRWalk |
| *FYN* | hsa-miR-6759-3p | miRWalk |
| *FYN* | hsa-miR-6771-5p | miRWalk |
| *FYN* | hsa-miR-6771-3p | miRWalk |
| *FYN* | hsa-miR-6803-3p | miRWalk |
| *FYN* | hsa-miR-6819-3p | miRWalk |
| *FYN* | hsa-miR-6826-3p | miRWalk |
| *FYN* | hsa-miR-6887-3p | miRWalk |
| *FYN* | hsa-miR-6888-3p | miRWalk |
| *FYN* | hsa-miR-6894-3p | miRWalk |
| *FYN* | hsa-miR-6895-3p | miRWalk |
| *FYN* | hsa-miR-206 | miRWalk |
| *FYN* | hsa-miR-6765-5p | miRWalk |
| *FYN* | hsa-miR-6782-3p | miRWalk |
| *FYN* | hsa-miR-92a-2-5p | miRWalk |
| *FYN* | hsa-miR-512-3p | miRWalk |
| *FYN* | hsa-miR-630 | miRWalk |
| *FYN* | hsa-miR-6754-5p | miRWalk |
| *FYN* | hsa-miR-30a-3p | miRWalk |
| *FYN* | hsa-miR-381-5p | miRWalk |
| *FYN* | hsa-miR-9983-3p | miRWalk |
| *FYN* | hsa-miR-4667-5p | miRWalk |
| *FYN* | hsa-miR-4800-5p | miRWalk |
| *FYN* | hsa-miR-6816-5p | miRWalk |
| *FYN* | hsa-miR-3156-3p | miRWalk |
| *FYN* | hsa-miR-3619-5p | miRWalk |
| *FYN* | hsa-miR-4651 | miRWalk |
| *FYN* | hsa-miR-4323 | miRWalk |
| *FYN* | hsa-miR-3065-3p | miRWalk |
| *FYN* | hsa-miR-3944-3p | miRWalk |
| *FYN* | hsa-let-7a-2-3p | miRWalk |
| *FYN* | hsa-miR-197-5p | miRWalk |
| *FYN* | hsa-miR-129-1-3p | miRWalk |
| *FYN* | hsa-miR-141-5p | miRWalk |
| *FYN* | hsa-miR-152-3p | miRWalk |
| *FYN* | hsa-miR-106b-5p | miRWalk |
| *FYN* | hsa-miR-382-5p | miRWalk |
| *FYN* | hsa-miR-324-5p | miRWalk |
| *FYN* | hsa-miR-425-3p | miRWalk |
| *FYN* | hsa-miR-490-3p | miRWalk |
| *FYN* | hsa-miR-520f-5p | miRWalk |
| *FYN* | hsa-miR-517a-3p | miRWalk |
| *FYN* | hsa-miR-517b-3p | miRWalk |
| *FYN* | hsa-miR-501-3p | miRWalk |
| *FYN* | hsa-miR-504-3p | miRWalk |
| *FYN* | hsa-miR-551a | miRWalk |
| *FYN* | hsa-miR-578 | miRWalk |
| *FYN* | hsa-miR-671-5p | miRWalk |
| *FYN* | hsa-miR-1224-3p | miRWalk |
| *FYN* | hsa-miR-1301-5p | miRWalk |
| *FYN* | hsa-miR-449c-3p | miRWalk |
| *FYN* | hsa-miR-1283 | miRWalk |
| *FYN* | hsa-miR-298 | miRWalk |
| *FYN* | hsa-miR-1270 | miRWalk |
| *FYN* | hsa-miR-664a-5p | miRWalk |
| *FYN* | hsa-miR-2276-5p | miRWalk |
| *FYN* | hsa-miR-3116 | miRWalk |
| *FYN* | hsa-miR-3159 | miRWalk |
| *FYN* | hsa-miR-4316 | miRWalk |
| *FYN* | hsa-miR-4326 | miRWalk |
| *FYN* | hsa-miR-4290 | miRWalk |
| *FYN* | hsa-miR-3667-3p | miRWalk |
| *FYN* | hsa-miR-3926 | miRWalk |
| *FYN* | hsa-miR-3945 | miRWalk |
| *FYN* | hsa-miR-4469 | miRWalk |
| *FYN* | hsa-miR-4485-3p | miRWalk |
| *FYN* | hsa-miR-4524a-5p | miRWalk |
| *FYN* | hsa-miR-4649-5p | miRWalk |
| *FYN* | hsa-miR-4671-3p | miRWalk |
| *FYN* | hsa-miR-4690-3p | miRWalk |
| *FYN* | hsa-miR-4727-3p | miRWalk |
| *FYN* | hsa-miR-4729 | miRWalk |
| *FYN* | hsa-miR-371b-3p | miRWalk |
| *FYN* | hsa-miR-4757-5p | miRWalk |
| *FYN* | hsa-miR-4769-3p | miRWalk |
| *FYN* | hsa-miR-4782-5p | miRWalk |
| *FYN* | hsa-miR-4786-5p | miRWalk |
| *FYN* | hsa-miR-4804-3p | miRWalk |
| *FYN* | hsa-miR-5088-3p | miRWalk |
| *FYN* | hsa-miR-5089-5p | miRWalk |
| *FYN* | hsa-miR-5192 | miRWalk |
| *FYN* | hsa-miR-5586-3p | miRWalk |
| *FYN* | hsa-miR-548aw | miRWalk |
| *FYN* | hsa-miR-5681b | miRWalk |
| *FYN* | hsa-miR-5787 | miRWalk |
| *FYN* | hsa-miR-6729-3p | miRWalk |
| *FYN* | hsa-miR-6730-3p | miRWalk |
| *FYN* | hsa-miR-6731-3p | miRWalk |
| *FYN* | hsa-miR-6735-5p | miRWalk |
| *FYN* | hsa-miR-6785-3p | miRWalk |
| *FYN* | hsa-miR-6786-3p | miRWalk |
| *FYN* | hsa-miR-6795-3p | miRWalk |
| *FYN* | hsa-miR-6797-3p | miRWalk |
| *FYN* | hsa-miR-6798-3p | miRWalk |
| *FYN* | hsa-miR-6805-5p | miRWalk |
| *FYN* | hsa-miR-6810-3p | miRWalk |
| *FYN* | hsa-miR-6813-3p | miRWalk |
| *FYN* | hsa-miR-6823-3p | miRWalk |
| *FYN* | hsa-miR-6836-3p | miRWalk |
| *FYN* | hsa-miR-6846-3p | miRWalk |
| *FYN* | hsa-miR-6858-3p | miRWalk |
| *FYN* | hsa-miR-6769b-5p | miRWalk |
| *FYN* | hsa-miR-6867-5p | miRWalk |
| *FYN* | hsa-miR-7158-5p | miRWalk |
| *FYN* | hsa-miR-7703 | miRWalk |
| *FYN* | hsa-miR-6516-5p | miRWalk |
| *FYN* | hsa-miR-9718 | miRWalk |
| *FYN* | hsa-miR-10400-3p | miRWalk |
| *FYN* | hsa-miR-3085-5p | miRWalk |
| *FYN* | hsa-miR-9851-5p | miRWalk |
| *FYN* | hsa-miR-485-5p | miRWalk |
| *FYN* | hsa-miR-1225-3p | miRWalk |
| *FYN* | hsa-miR-1234-3p | miRWalk |
| *FYN* | hsa-miR-1236-3p | miRWalk |
| *FYN* | hsa-miR-1908-3p | miRWalk |
| *FYN* | hsa-miR-3619-3p | miRWalk |
| *FYN* | hsa-miR-4492 | miRWalk |
| *FYN* | hsa-miR-4684-3p | miRWalk |
| *FYN* | hsa-miR-2467-3p | miRWalk |
| *FYN* | hsa-miR-6073 | miRWalk |
| *FYN* | hsa-miR-6074 | miRWalk |
| *FYN* | hsa-miR-6075 | miRWalk |
| *FYN* | hsa-miR-6799-3p | miRWalk |
| *FYN* | hsa-miR-6814-3p | miRWalk |
| *FYN* | hsa-miR-6842-5p | miRWalk |
| *FYN* | hsa-miR-7106-3p | miRWalk |
| *FYN* | hsa-miR-7111-3p | miRWalk |
| *FYN* | hsa-miR-103a-3p | miRWalk |
| *FYN* | hsa-miR-107 | miRWalk |
| *FYN* | hsa-miR-30d-3p | miRWalk |
| *FYN* | hsa-miR-183-5p | miRWalk |
| *FYN* | hsa-miR-125b-1-3p | miRWalk |
| *FYN* | hsa-miR-186-5p | miRWalk |
| *FYN* | hsa-miR-193a-5p | miRWalk |
| *FYN* | hsa-miR-106b-3p | miRWalk |
| *FYN* | hsa-miR-370-5p | miRWalk |
| *FYN* | hsa-miR-370-3p | miRWalk |
| *FYN* | hsa-miR-330-5p | miRWalk |
| *FYN* | hsa-miR-331-5p | miRWalk |
| *FYN* | hsa-miR-339-3p | miRWalk |
| *FYN* | hsa-miR-193b-3p | miRWalk |
| *FYN* | hsa-miR-520a-3p | miRWalk |
| *FYN* | hsa-miR-499a-5p | miRWalk |
| *FYN* | hsa-miR-506-5p | miRWalk |
| *FYN* | hsa-miR-510-5p | miRWalk |
| *FYN* | hsa-miR-92b-5p | miRWalk |
| *FYN* | hsa-miR-601 | miRWalk |
| *FYN* | hsa-miR-604 | miRWalk |
| *FYN* | hsa-miR-614 | miRWalk |
| *FYN* | hsa-miR-650 | miRWalk |
| *FYN* | hsa-miR-662 | miRWalk |
| *FYN* | hsa-miR-769-5p | miRWalk |
| *FYN* | hsa-miR-769-3p | miRWalk |
| *FYN* | hsa-miR-541-3p | miRWalk |
| *FYN* | hsa-miR-216b-5p | miRWalk |
| *FYN* | hsa-miR-920 | miRWalk |
| *FYN* | hsa-miR-1182 | miRWalk |
| *FYN* | hsa-miR-1184 | miRWalk |
| *FYN* | hsa-miR-663b | miRWalk |
| *FYN* | hsa-miR-1204 | miRWalk |
| *FYN* | hsa-miR-1286 | miRWalk |
| *FYN* | hsa-miR-1265 | miRWalk |
| *FYN* | hsa-miR-513b-5p | miRWalk |
| *FYN* | hsa-miR-1914-3p | miRWalk |
| *FYN* | hsa-miR-2276-3p | miRWalk |
| *FYN* | hsa-miR-3130-5p | miRWalk |
| *FYN* | hsa-miR-3131 | miRWalk |
| *FYN* | hsa-miR-3136-5p | miRWalk |
| *FYN* | hsa-miR-3141 | miRWalk |
| *FYN* | hsa-miR-548v | miRWalk |
| *FYN* | hsa-miR-3157-5p | miRWalk |
| *FYN* | hsa-miR-3162-5p | miRWalk |
| *FYN* | hsa-miR-3167 | miRWalk |
| *FYN* | hsa-miR-3190-5p | miRWalk |
| *FYN* | hsa-miR-3191-3p | miRWalk |
| *FYN* | hsa-miR-3198 | miRWalk |
| *FYN* | hsa-miR-3200-5p | miRWalk |
| *FYN* | hsa-miR-4320 | miRWalk |
| *FYN* | hsa-miR-4270 | miRWalk |
| *FYN* | hsa-miR-4281 | miRWalk |
| *FYN* | hsa-miR-3680-3p | miRWalk |
| *FYN* | hsa-miR-3692-5p | miRWalk |
| *FYN* | hsa-miR-3713 | miRWalk |
| *FYN* | hsa-miR-3909 | miRWalk |
| *FYN* | hsa-miR-3917 | miRWalk |
| *FYN* | hsa-miR-3918 | miRWalk |
| *FYN* | hsa-miR-3925-3p | miRWalk |
| *FYN* | hsa-miR-3934-3p | miRWalk |
| *FYN* | hsa-miR-4472 | miRWalk |
| *FYN* | hsa-miR-4478 | miRWalk |
| *FYN* | hsa-miR-4482-5p | miRWalk |
| *FYN* | hsa-miR-4483 | miRWalk |
| *FYN* | hsa-miR-4486 | miRWalk |
| *FYN* | hsa-miR-4487 | miRWalk |
| *FYN* | hsa-miR-4510 | miRWalk |
| *FYN* | hsa-miR-4516 | miRWalk |
| *FYN* | hsa-miR-4522 | miRWalk |
| *FYN* | hsa-miR-4538 | miRWalk |
| *FYN* | hsa-miR-4632-5p | miRWalk |
| *FYN* | hsa-miR-4650-3p | miRWalk |
| *FYN* | hsa-miR-4653-5p | miRWalk |
| *FYN* | hsa-miR-4656 | miRWalk |
| *FYN* | hsa-miR-4660 | miRWalk |
| *FYN* | hsa-miR-4665-3p | miRWalk |
| *FYN* | hsa-miR-4667-3p | miRWalk |
| *FYN* | hsa-miR-4672 | miRWalk |
| *FYN* | hsa-miR-4684-5p | miRWalk |
| *FYN* | hsa-miR-4687-3p | miRWalk |
| *FYN* | hsa-miR-4688 | miRWalk |
| *FYN* | hsa-miR-4697-5p | miRWalk |
| *FYN* | hsa-miR-4710 | miRWalk |
| *FYN* | hsa-miR-4711-5p | miRWalk |
| *FYN* | hsa-miR-4717-3p | miRWalk |
| *FYN* | hsa-miR-4724-5p | miRWalk |
| *FYN* | hsa-miR-4725-3p | miRWalk |
| *FYN* | hsa-miR-4747-5p | miRWalk |
| *FYN* | hsa-miR-4748 | miRWalk |
| *FYN* | hsa-miR-4756-5p | miRWalk |
| *FYN* | hsa-miR-4758-5p | miRWalk |
| *FYN* | hsa-miR-4768-5p | miRWalk |
| *FYN* | hsa-miR-4787-5p | miRWalk |
| *FYN* | hsa-miR-5006-3p | miRWalk |
| *FYN* | hsa-miR-5009-5p | miRWalk |
| *FYN* | hsa-miR-5087 | miRWalk |
| *FYN* | hsa-miR-5100 | miRWalk |
| *FYN* | hsa-miR-5572 | miRWalk |
| *FYN* | hsa-miR-5584-3p | miRWalk |
| *FYN* | hsa-miR-5705 | miRWalk |
| *FYN* | hsa-miR-6085 | miRWalk |
| *FYN* | hsa-miR-6088 | miRWalk |
| *FYN* | hsa-miR-6089 | miRWalk |
| *FYN* | hsa-miR-6127 | miRWalk |
| *FYN* | hsa-miR-6133 | miRWalk |
| *FYN* | hsa-miR-6507-3p | miRWalk |
| *FYN* | hsa-miR-6515-5p | miRWalk |
| *FYN* | hsa-miR-6720-5p | miRWalk |
| *FYN* | hsa-miR-6722-3p | miRWalk |
| *FYN* | hsa-miR-6727-5p | miRWalk |
| *FYN* | hsa-miR-6736-5p | miRWalk |
| *FYN* | hsa-miR-6746-5p | miRWalk |
| *FYN* | hsa-miR-6748-5p | miRWalk |
| *FYN* | hsa-miR-6752-5p | miRWalk |
| *FYN* | hsa-miR-6763-5p | miRWalk |
| *FYN* | hsa-miR-6766-5p | miRWalk |
| *FYN* | hsa-miR-6782-5p | miRWalk |
| *FYN* | hsa-miR-6785-5p | miRWalk |
| *FYN* | hsa-miR-6810-5p | miRWalk |
| *FYN* | hsa-miR-6824-5p | miRWalk |
| *FYN* | hsa-miR-6830-3p | miRWalk |
| *FYN* | hsa-miR-6835-5p | miRWalk |
| *FYN* | hsa-miR-6842-3p | miRWalk |
| *FYN* | hsa-miR-6843-3p | miRWalk |
| *FYN* | hsa-miR-6851-5p | miRWalk |
| *FYN* | hsa-miR-6860 | miRWalk |
| *FYN* | hsa-miR-6883-5p | miRWalk |
| *FYN* | hsa-miR-6885-5p | miRWalk |
| *FYN* | hsa-miR-6891-5p | miRWalk |
| *FYN* | hsa-miR-6893-5p | miRWalk |
| *FYN* | hsa-miR-7106-5p | miRWalk |
| *FYN* | hsa-miR-7110-5p | miRWalk |
| *FYN* | hsa-miR-7154-3p | miRWalk |
| *FYN* | hsa-miR-7158-3p | miRWalk |
| *FYN* | hsa-miR-1273h-5p | miRWalk |
| *FYN* | hsa-miR-7849-3p | miRWalk |
| *FYN* | hsa-miR-8069 | miRWalk |
| *FYN* | hsa-miR-8071 | miRWalk |
| *FYN* | hsa-miR-8078 | miRWalk |
| *FYN* | hsa-miR-8080 | miRWalk |
| *FYN* | hsa-miR-10396b-5p | miRWalk |
| *FYN* | hsa-miR-11181-3p | miRWalk |
| *FYN* | hsa-let-7c-3p | miRWalk |
| *FYN* | hsa-let-7e-3p | miRWalk |
| *FYN* | hsa-miR-19a-5p | miRWalk |
| *FYN* | hsa-miR-28-3p | miRWalk |
| *FYN* | hsa-miR-197-3p | miRWalk |
| *FYN* | hsa-miR-214-5p | miRWalk |
| *FYN* | hsa-miR-150-5p | miRWalk |
| *FYN* | hsa-miR-372-3p | miRWalk |
| *FYN* | hsa-miR-519b-3p | miRWalk |
| *FYN* | hsa-miR-517c-3p | miRWalk |
| *FYN* | hsa-miR-519a-3p | miRWalk |
| *FYN* | hsa-miR-450a-2-3p | miRWalk |
| *FYN* | hsa-miR-652-5p | miRWalk |
| *FYN* | hsa-miR-670-5p | miRWalk |
| *FYN* | hsa-miR-675-3p | miRWalk |
| *FYN* | hsa-miR-1236-5p | miRWalk |
| *FYN* | hsa-miR-1207-3p | miRWalk |
| *FYN* | hsa-miR-2115-5p | miRWalk |
| *FYN* | hsa-miR-3124-3p | miRWalk |
| *FYN* | hsa-miR-3162-3p | miRWalk |
| *FYN* | hsa-miR-3184-3p | miRWalk |
| *FYN* | hsa-miR-3150b-3p | miRWalk |
| *FYN* | hsa-miR-3689d | miRWalk |
| *FYN* | hsa-miR-4652-5p | miRWalk |
| *FYN* | hsa-miR-4652-3p | miRWalk |
| *FYN* | hsa-miR-4677-5p | miRWalk |
| *FYN* | hsa-miR-4713-5p | miRWalk |
| *FYN* | hsa-miR-1245b-5p | miRWalk |
| *FYN* | hsa-miR-548ao-3p | miRWalk |
| *FYN* | hsa-miR-5187-3p | miRWalk |
| *FYN* | hsa-miR-5196-3p | miRWalk |
| *FYN* | hsa-miR-548as-5p | miRWalk |
| *FYN* | hsa-miR-6515-3p | miRWalk |
| *FYN* | hsa-miR-6732-3p | miRWalk |
| *FYN* | hsa-miR-6734-3p | miRWalk |
| *FYN* | hsa-miR-6737-3p | miRWalk |
| *FYN* | hsa-miR-6741-3p | miRWalk |
| *FYN* | hsa-miR-6746-3p | miRWalk |
| *FYN* | hsa-miR-6761-5p | miRWalk |
| *FYN* | hsa-miR-6793-3p | miRWalk |
| *FYN* | hsa-miR-6794-3p | miRWalk |
| *FYN* | hsa-miR-6816-3p | miRWalk |
| *FYN* | hsa-miR-6827-3p | miRWalk |
| *FYN* | hsa-miR-6828-3p | miRWalk |
| *FYN* | hsa-miR-6831-3p | miRWalk |
| *FYN* | hsa-miR-6832-3p | miRWalk |
| *FYN* | hsa-miR-6780b-3p | miRWalk |
| *FYN* | hsa-miR-6841-3p | miRWalk |
| *FYN* | hsa-miR-6852-5p | miRWalk |
| *FYN* | hsa-miR-6853-3p | miRWalk |
| *FYN* | hsa-miR-6877-3p | miRWalk |
| *FYN* | hsa-miR-6880-3p | miRWalk |
| *FYN* | hsa-miR-6883-3p | miRWalk |
| *FYN* | hsa-miR-6884-3p | miRWalk |
| *FYN* | hsa-miR-6889-3p | miRWalk |
| *FYN* | hsa-miR-6891-3p | miRWalk |
| *FYN* | hsa-miR-7108-3p | miRWalk |
| *FYN* | hsa-miR-7162-3p | miRWalk |
| *FYN* | hsa-miR-9986 | miRWalk |
| *FYN* | hsa-miR-12131 | miRWalk |
