# Supplementary material for: Integrated bioinformatics analysis for the identification of idiopathic pulmonary fibrosis–related genes and potential therapeutic drugs
Source: BMC Pulm Med. 2023 Oct 4;23:373. doi: 10.1186/s12890-023-02678-z (PMC10552267; doi:10.1186/s12890-023-02678-z)
Supplement: Supplementary file 1 — Additional file 1: Table S1. The analyze network results of 1640 DEGs. Table S2. GO terms of the 18 hub genes. Table S3. KEGG pathways of the 18 hub genes. Table S4. Target microRNAs of SPP1 based on five online miRNA databases. Table S5. Target microRNAs of VEGFA based on five online miRNA databases. Table S6. Target microRNAs of COL1A1 based on five online miRNA databases. Table S7. Target microRNAs of CAV1 based on five online miRNA databases. Table S8. Target microRNAs of PECAM1 based on five online miRNA databases. Table S9. Target microRNAs of BMP4 based on five online miRNA databases. Table S10. Target microRNAs of FYN based on five online miRNA databases. Table S11. Traditional Chinese medicine prediction results of COL1A1. Table S12. Traditional Chinese medicine prediction results of VEGFA. Table S13. Traditional Chinese medicine prediction results of SPP1. [file 12890_2023_2678_MOESM1_ESM.zip › Supplementary Tables/Supplementary Table11.docx]

**Table S11 Traditional Chinese medicine prediction results of *COL1A1***

| Gene Symbol | Herb | FDR |
| --- | --- | --- |
| *COL1A1* | Buckeye Seed | 1.06E-04 |
| *COL1A1* | Salviae Chinensis Herba | 1.18E-04 |
| *COL1A1* | rhizome of Airpotato Yam | 3.55E-04 |
| *COL1A1* | Flatstem Milkvetch Seed | 3.86E-04 |
| *COL1A1* | Asiatic Cornelian Cherry Fruit | 4.29E-04 |
| *COL1A1* | root of Ligulilobe sage | 6.18E-04 |
| *COL1A1* | fruit of Axillary choerospondias | 9.37E-04 |
| *COL1A1* | Glabrous Greenbrier Rhizome | 9.42E-04 |
| *COL1A1* | Gnaphalium Affine | 9.53E-04 |
| *COL1A1* | Lotus leaf | 9.72E-04 |
| *COL1A1* | all - grass of Japanese St. Johnswort | 1.10E-03 |
| *COL1A1* | Ginkgo seed | 1.51E-03 |
| *COL1A1* | Armand Clematis Stem | 1.77E-03 |
| *COL1A1* | Jujube Chinese date | 1.83E-03 |
| *COL1A1* | root of Grand Hogfennel | 1.86E-03 |
| *COL1A1* | flower bud of common coltsfoot | 1.88E-03 |
| *COL1A1* | Tuber - root of Common Turmeric | 1.92E-03 |
| *COL1A1* | Vacarria seed, Cow soapwort seed | 2.04E-03 |
| *COL1A1* | spine of Chinese Honeylocust | 2.04E-03 |
| *COL1A1* | Chicory Herb | 2.05E-03 |
| *COL1A1* | Dried Tangerine Peel | 2.45E-03 |
| *COL1A1* | all-grass of Tubercultae speranskia | 2.47E-03 |
| *COL1A1* | Sapindi Mukorossiperic Arpium | 2.58E-03 |
| *COL1A1* | all - grass of Stringy stonecrop | 2.60E-03 |
| *COL1A1* | all-grass of Glabrous sarcandra | 2.68E-03 |
| *COL1A1* | Cochinchinese Asparagus Root | 2.78E-03 |
| *COL1A1* | European Verbe Herb | 2.95E-03 |
| *COL1A1* | Mulberry Fruit | 3.20E-03 |
| *COL1A1* | root of Twotooth Achyranthes | 3.23E-03 |
| *COL1A1* | Pumpkin seed and husks | 3.23E-03 |
| *COL1A1* | Smoked Plum | 3.23E-03 |
| *COL1A1* | Pipewort Flower | 3.34E-03 |
| *COL1A1* | Gynostemmae Pentaphylli Herba | 3.40E-03 |
| *COL1A1* | flower bud of lobed kudzuvine | 3.44E-03 |
| *COL1A1* | Microctis Folium | 3.44E-03 |
| *COL1A1* | fennel fruit | 3.79E-03 |
| *COL1A1* | Paniculate Bolbostemma | 3.90E-03 |
| *COL1A1* | root of Medicil cyathula | 4.01E-03 |
| *COL1A1* | Root of Dyers Woad. | 4.06E-03 |
| *COL1A1* | all - grass of Humifuse Euphorbia | 4.13E-03 |
| *COL1A1* | stem or leaf of Fung Waxplant | 4.13E-03 |
| *COL1A1* | Myrrh | 4.13E-03 |
| *COL1A1* | Honey | 4.37E-03 |
| *COL1A1* | motherwort | 4.49E-03 |
| *COL1A1* | Globethistle Root | 4.49E-03 |
| *COL1A1* | Litseae Fructus | 4.60E-03 |
| *COL1A1* | root of Gansui | 4.72E-03 |
| *COL1A1* | flower bud of Lilac Daphne | 5.23E-03 |
| *COL1A1* | Actinidia Chinensis Planch | 5.36E-03 |
| *COL1A1* | root of Common Monkshood | 5.40E-03 |
| *COL1A1* | Silybum Marianum | 5.63E-03 |
| *COL1A1* | fruit -spike of Common Selfheal | 5.63E-03 |
| *COL1A1* | stem of Chinese starjasmine | 5.78E-03 |
| *COL1A1* | All - grass of Dahurian Patrinia | 6.06E-03 |
| *COL1A1* | rhizome of Chinese Goldthread | 6.06E-03 |
| *COL1A1* | Lumbricus terrestris | 6.71E-03 |
| *COL1A1* | flower of Chinese Globeflower | 6.78E-03 |
| *COL1A1* | all - grass of Chinese Brake | 6.89E-03 |
| *COL1A1* | Saffron | 6.89E-03 |
| *COL1A1* | all-grass of Spanishneedles | 7.02E-03 |
| *COL1A1* | Prince's-feather Fruit | 7.02E-03 |
| *COL1A1* | Emblic Leafflower Fruit | 7.09E-03 |
| *COL1A1* | root of Membranous Milkvetch | 7.09E-03 |
| *COL1A1* | Carii Fructus | 7.15E-03 |
| *COL1A1* | Chinese Lizardtail Rhizome or Herb | 7.15E-03 |
| *COL1A1* | Purpleflower Holly Leaf | 7.15E-03 |
| *COL1A1* | Sea Buckthorn | 7.17E-03 |
| *COL1A1* | White Hyacinth Bean | 7.23E-03 |
| *COL1A1* | Asiatic Pennywort Herb | 7.30E-03 |
| *COL1A1* | Boat-fruited Sterculia Seed | 7.30E-03 |
| *COL1A1* | Honeysuckle Flower | 7.74E-03 |
| *COL1A1* | fruit of Cherokee Rose | 7.76E-03 |
| *COL1A1* | all-grass of Shepherdspurse | 7.76E-03 |
| *COL1A1* | root of Tali Madder | 7.91E-03 |
| *COL1A1* | all-grass of Purslane | 8.07E-03 |
| *COL1A1* | Wild Buckwheat Rhizome | 8.21E-03 |
| *COL1A1* | all-grass of Rippleseed plantain | 8.23E-03 |
| *COL1A1* | Lepidium seed, Descurainia seed | 8.37E-03 |
| *COL1A1* | Euphorbiae Helioscopiae Herba | 8.37E-03 |
| *COL1A1* | Chinese Clematis Root | 8.50E-03 |
| *COL1A1* | Bombyx batryticatus | 8.80E-03 |
| *COL1A1* | fruit of Glossy privet | 9.33E-03 |
| *COL1A1* | bulb of longstamen onion | 9.53E-03 |

Notes. FDR denotes false discovery rate.
