# Supplementary material for: Integrated bioinformatics analysis for the identification of idiopathic pulmonary fibrosis–related genes and potential therapeutic drugs
Source: BMC Pulm Med. 2023 Oct 4;23:373. doi: 10.1186/s12890-023-02678-z (PMC10552267; doi:10.1186/s12890-023-02678-z)
Supplement: Supplementary file 1 — Additional file 1: Table S1. The analyze network results of 1640 DEGs. Table S2. GO terms of the 18 hub genes. Table S3. KEGG pathways of the 18 hub genes. Table S4. Target microRNAs of SPP1 based on five online miRNA databases. Table S5. Target microRNAs of VEGFA based on five online miRNA databases. Table S6. Target microRNAs of COL1A1 based on five online miRNA databases. Table S7. Target microRNAs of CAV1 based on five online miRNA databases. Table S8. Target microRNAs of PECAM1 based on five online miRNA databases. Table S9. Target microRNAs of BMP4 based on five online miRNA databases. Table S10. Target microRNAs of FYN based on five online miRNA databases. Table S11. Traditional Chinese medicine prediction results of COL1A1. Table S12. Traditional Chinese medicine prediction results of VEGFA. Table S13. Traditional Chinese medicine prediction results of SPP1. [file 12890_2023_2678_MOESM1_ESM.zip › Supplementary Tables/Supplementary Table12.docx]

**Table S12 Traditional Chinese medicine prediction results of *VEGFA***

| Gene Symbol | | Herb | FDR |
| --- | --- | --- | --- |
| *VEGFA* | Ginkgo seed | | 0.00E+00 |
| *VEGFA* | Chinese Cinquefoil | | 0.00E+00 |
| *VEGFA* | Indian Trumpetflower Seed | | 2.00E-06 |
| *VEGFA* | Safflower | | 8.00E-06 |
| *VEGFA* | all - grss of Hairyvein Agrimonia | | 8.00E-06 |
| *VEGFA* | Fermented Soybean | | 2.00E-05 |
| *VEGFA* | all - grass of Barbed Skullcap | | 2.10E-05 |
| *VEGFA* | Semen Pini Koraiensis | | 2.50E-05 |
| *VEGFA* | Herba Lagotidis | | 3.00E-05 |
| *VEGFA* | All - grass of Dahurian Patrinia | | 4.50E-05 |
| *VEGFA* | all-grass of Purslane | | 4.50E-05 |
| *VEGFA* | Radix Stephaniae Cepharanthae. | | 7.60E-05 |
| *VEGFA* | Aloe | | 9.30E-05 |
| *VEGFA* | Solanum Nigrum Linn. | | 1.39E-04 |
| *VEGFA* | Clinopodium Herb | | 2.01E-04 |
| *VEGFA* | Common Scouring Rush Herb | | 2.31E-04 |
| *VEGFA* | Wild Chrysanthemum Flower | | 2.44E-04 |
| *VEGFA* | root of Medicil cyathula | | 2.62E-04 |
| *VEGFA* | All - grass of Common Heron's bill | | 3.13E-04 |
| *VEGFA* | Broussonetiae Fructus | | 3.96E-04 |
| *VEGFA* | root of lobed kudzuvine | | 4.06E-04 |
| *VEGFA* | Folium Ilicis Cornutae | | 4.24E-04 |
| *VEGFA* | All - grass of Japanese Inula | | 4.35E-04 |
| *VEGFA* | flower bud of Lilac Daphne | | 4.57E-04 |
| *VEGFA* | all-grass of Yerbadetajo | | 4.81E-04 |
| *VEGFA* | Buckeye Seed | | 4.81E-04 |
| *VEGFA* | Sea Buckthorn | | 5.32E-04 |
| *VEGFA* | all-grass of Common Ducksmeat | | 6.14E-04 |
| *VEGFA* | Chicory Herb | | 7.07E-04 |
| *VEGFA* | Hemp Seed | | 7.29E-04 |
| *VEGFA* | Semino-dermis Arachidis | | 7.81E-04 |
| *VEGFA* | seed of Asiatic pantain | | 8.19E-04 |
| *VEGFA* | Alpiniae Officirum Rhizome | | 8.19E-04 |
| *VEGFA* | rhizome of Gaint Knotweed | | 8.63E-04 |
| *VEGFA* | Purpleflower Holly Leaf | | 8.63E-04 |
| *VEGFA* | Asiatic Pennywort Herb | | 8.99E-04 |
| *VEGFA* | fruit of Cherokee Rose | | 1.01E-03 |
| *VEGFA* | Rhizome of Fragrant Solomonseal | | 1.03E-03 |
| *VEGFA* | Vietmese Sophora Root | | 1.07E-03 |
| *VEGFA* | Sweet Wormwood Herb | | 1.11E-03 |
| *VEGFA* | fruit of Chinese Wolfberry | | 1.21E-03 |
| *VEGFA* | Ailanthi Altissimae Fructus | | 1.34E-03 |
| *VEGFA* | all-grass of Rippleseed plantain | | 1.36E-03 |
| *VEGFA* | root of Membranous Milkvetch | | 1.40E-03 |
| *VEGFA* | Germited Barley | | 1.42E-03 |
| *VEGFA* | Pomegrate Rind | | 1.45E-03 |
| *VEGFA* | Weeping Forsythia Capsule | | 1.47E-03 |
| *VEGFA* | Jujube Chinese date | | 1.50E-03 |
| *VEGFA* | Dioscoreae Septemlo Bae Rhizoma | | 1.63E-03 |
| *VEGFA* | Emblic Leafflower Fruit | | 1.70E-03 |
| *VEGFA* | Suberect Spatholobus Stem | | 1.70E-03 |
| *VEGFA* | root of common htreewingnut | | 1.92E-03 |
| *VEGFA* | Hoveniae Dulcis Semen | | 2.06E-03 |
| *VEGFA* | Cortex Mori | | 2.29E-03 |
| *VEGFA* | Microctis Folium | | 2.54E-03 |
| *VEGFA* | Puerariae Thomsonii Radix | | 2.54E-03 |
| *VEGFA* | Lysimachiae Foenigraeci Herba | | 2.62E-03 |
| *VEGFA* | European Verbe Herb | | 2.66E-03 |
| *VEGFA* | Crataegi Folium | | 2.67E-03 |
| *VEGFA* | Hydnocarpi Anthelminticae Semen | | 2.80E-03 |
| *VEGFA* | Honeysuckle Flower | | 2.90E-03 |
| *VEGFA* | root Pilose Asiabell | | 3.02E-03 |
| *VEGFA* | Trigonellae Semen | | 3.03E-03 |
| *VEGFA* | Blackberrylily Rhizome | | 3.03E-03 |
| *VEGFA* | Perilla Leaf | | 3.04E-03 |
| *VEGFA* | mung bean | | 3.14E-03 |
| *VEGFA* | Gnaphalium Affine | | 3.18E-03 |
| *VEGFA* | pod of Japanese pagodatree | | 3.22E-03 |
| *VEGFA* | flower bud of lobed kudzuvine | | 3.28E-03 |
| *VEGFA* | Silybum Marianum | | 3.37E-03 |
| *VEGFA* | Lamiophlomis rotata | | 3.59E-03 |
| *VEGFA* | root of Ligulilobe sage | | 3.59E-03 |
| *VEGFA* | all-grass of japanese hop | | 3.75E-03 |
| *VEGFA* | Field Thistle Herb | | 4.00E-03 |
| *VEGFA* | White Hyacinth Bean | | 4.17E-03 |
| *VEGFA* | Flower of Magnolia | | 4.21E-03 |
| *VEGFA* | Chinese Dwarf Cherry Seed | | 4.45E-03 |
| *VEGFA* | seed of Gordon Euryale | | 4.64E-03 |
| *VEGFA* | Chelidonii Herba | | 4.87E-03 |
| *VEGFA* | flower of Hyacinth Dolichos | | 5.11E-03 |
| *VEGFA* | pollen of longbract cattail | | 5.25E-03 |
| *VEGFA* | fruit -spike of Common Selfheal | | 5.25E-03 |
| *VEGFA* | Ephedra | | 5.30E-03 |
| *VEGFA* | Rose Flower | | 5.37E-03 |
| *VEGFA* | stem of Chinese starjasmine | | 5.43E-03 |
| *VEGFA* | Celastri Orbiculati Fructus | | 5.51E-03 |
| *VEGFA* | Glechomae Herba | | 6.83E-03 |
| *VEGFA* | flower of Chinese Globeflower | | 6.84E-03 |
| *VEGFA* | Actinidia Chinensis Planch | | 6.91E-03 |
| *VEGFA* | all - grass of Chinese Brake | | 7.04E-03 |
| *VEGFA* | root of Japanese Ampelopsis | | 7.23E-03 |
| *VEGFA* | Lotus stamen | | 7.25E-03 |
| *VEGFA* | root of Grand Hogfennel | | 7.41E-03 |
| *VEGFA* | all-grass of Haichow Elsholtzia | | 7.41E-03 |
| *VEGFA* | Glabrous Greenbrier Rhizome | | 7.43E-03 |
| *VEGFA* | Schizonepetae Spica | | 7.48E-03 |
| *VEGFA* | root of Chinese Thorowax | | 7.56E-03 |
| *VEGFA* | twig of Winged Euonymus | | 7.65E-03 |
| *VEGFA* | Eucommia bark | | 7.67E-03 |
| *VEGFA* | Cayratiae Japonicae Herba | | 7.68E-03 |
| *VEGFA* | root of Baikal skullcap | | 7.80E-03 |
| *VEGFA* | Longan Pulp | | 7.81E-03 |
| *VEGFA* | Flatstem Milkvetch Seed | | 8.63E-03 |
| *VEGFA* | Trichosanthes root, Skegourd root | | 9.32E-03 |
| *VEGFA* | Coriandri Sativi Herba | | 9.33E-03 |
| *VEGFA* | root of Coastal Glehnia | | 9.37E-03 |
| *VEGFA* | Cockscomb flower | | 9.95E-03 |

Notes. FDR denotes false discovery rate.
