# Supplementary material for: Integrated bioinformatics analysis for the identification of idiopathic pulmonary fibrosis–related genes and potential therapeutic drugs
Source: BMC Pulm Med. 2023 Oct 4;23:373. doi: 10.1186/s12890-023-02678-z (PMC10552267; doi:10.1186/s12890-023-02678-z)
Supplement: Supplementary file 1 — Additional file 1: Table S1. The analyze network results of 1640 DEGs. Table S2. GO terms of the 18 hub genes. Table S3. KEGG pathways of the 18 hub genes. Table S4. Target microRNAs of SPP1 based on five online miRNA databases. Table S5. Target microRNAs of VEGFA based on five online miRNA databases. Table S6. Target microRNAs of COL1A1 based on five online miRNA databases. Table S7. Target microRNAs of CAV1 based on five online miRNA databases. Table S8. Target microRNAs of PECAM1 based on five online miRNA databases. Table S9. Target microRNAs of BMP4 based on five online miRNA databases. Table S10. Target microRNAs of FYN based on five online miRNA databases. Table S11. Traditional Chinese medicine prediction results of COL1A1. Table S12. Traditional Chinese medicine prediction results of VEGFA. Table S13. Traditional Chinese medicine prediction results of SPP1. [file 12890_2023_2678_MOESM1_ESM.zip › Supplementary Tables/Supplementary Table6.docx]

**Table S6 Target microRNAs of *COL1A1* based on five online miRNA databases**

| Gene Symbol | microRNA | Database |  |
| --- | --- | --- | --- |
| *COL1A1* | | hsa-miR-5682 | mirDIP |
| *COL1A1* | | hsa-miR-548x-3p | mirDIP |
| *COL1A1* | | hsa-miR-548aj-3p | mirDIP |
| *COL1A1* | | hsa-miR-143-3p | mirDIP |
| *COL1A1* | | hsa-miR-625-5p | mirDIP |
| *COL1A1* | | hsa-miR-548am-3p | mirDIP |
| *COL1A1* | | hsa-miR-548ah-3p | mirDIP |
| *COL1A1* | | hsa-miR-548ae-3p | mirDIP |
| *COL1A1* | | hsa-miR-548aq-3p | mirDIP |
| *COL1A1* | | hsa-miR-193a-5p | mirDIP |
| *COL1A1* | | hsa-miR-4789-5p | mirDIP |
| *COL1A1* | | hsa-miR-4458 | mirDIP |
| *COL1A1* | | hsa-miR-4500 | mirDIP |
| *COL1A1* | | hsa-miR-3175 | mirDIP |
| *COL1A1* | | hsa-miR-516b-3p | mirDIP |
| *COL1A1* | | hsa-miR-3940-5p | mirDIP |
| *COL1A1* | | hsa-miR-92a-1-5p | mirDIP |
| *COL1A1* | | hsa-miR-4507 | mirDIP |
| *COL1A1* | | hsa-miR-7847-3p | mirDIP |
| *COL1A1* | | hsa-miR-4457 | mirDIP |
| *COL1A1* | | hsa-miR-4713-5p | mirDIP |
| *COL1A1* | | hsa-miR-6795-5p | mirDIP |
| *COL1A1* | | hsa-miR-4468 | mirDIP |
| *COL1A1* | | hsa-miR-6749-3p | mirDIP |
| *COL1A1* | | hsa-miR-2116-3p | mirDIP |
| *COL1A1* | | hsa-miR-7109-5p | mirDIP |
| *COL1A1* | | hsa-miR-6830-5p | mirDIP |
| *COL1A1* | | hsa-miR-363-5p | mirDIP |
| *COL1A1* | | hsa-miR-561-5p | mirDIP |
| *COL1A1* | | hsa-miR-365a-5p | mirDIP |
| *COL1A1* | | hsa-miR-4317 | mirDIP |
| *COL1A1* | | hsa-miR-6827-5p | mirDIP |
| *COL1A1* | | hsa-miR-6740-5p | mirDIP |
| *COL1A1* | | hsa-miR-4783-3p | mirDIP |
| *COL1A1* | | hsa-miR-6871-3p | mirDIP |
| *COL1A1* | | hsa-miR-7112-5p | mirDIP |
| *COL1A1* | | hsa-let-7a-5p | ENCORI |
| *COL1A1* | | hsa-let-7b-5p | ENCORI |
| *COL1A1* | | hsa-let-7c-5p | ENCORI |
| *COL1A1* | | hsa-let-7d-5p | ENCORI |
| *COL1A1* | | hsa-let-7e-5p | ENCORI |
| *COL1A1* | | hsa-let-7f-5p | ENCORI |
| *COL1A1* | | hsa-miR-15a-5p | ENCORI |
| *COL1A1* | | hsa-miR-16-5p | ENCORI |
| *COL1A1* | | hsa-miR-17-5p | ENCORI |
| *COL1A1* | | hsa-miR-20a-5p | ENCORI |
| *COL1A1* | | hsa-miR-23a-3p | ENCORI |
| *COL1A1* | | hsa-miR-28-5p | ENCORI |
| *COL1A1* | | hsa-miR-29a-3p | ENCORI |
| *COL1A1* | | hsa-miR-31-5p | ENCORI |
| *COL1A1* | | hsa-miR-93-5p | ENCORI |
| *COL1A1* | | hsa-miR-96-5p | ENCORI |
| *COL1A1* | | hsa-miR-98-5p | ENCORI |
| *COL1A1* | | hsa-miR-29b-3p | ENCORI |
| *COL1A1* | | hsa-miR-103a-3p | ENCORI |
| *COL1A1* | | hsa-miR-106a-5p | ENCORI |
| *COL1A1* | | hsa-miR-107 | ENCORI |
| *COL1A1* | | hsa-miR-196a-5p | ENCORI |
| *COL1A1* | | hsa-miR-197-3p | ENCORI |
| *COL1A1* | | hsa-miR-199a-5p | ENCORI |
| *COL1A1* | | hsa-miR-129-5p | ENCORI |
| *COL1A1* | | hsa-miR-10a-5p | ENCORI |
| *COL1A1* | | hsa-miR-10b-5p | ENCORI |
| *COL1A1* | | hsa-miR-34a-5p | ENCORI |
| *COL1A1* | | hsa-miR-182-5p | ENCORI |
| *COL1A1* | | hsa-miR-183-5p | ENCORI |
| *COL1A1* | | hsa-miR-199b-5p | ENCORI |
| *COL1A1* | | hsa-miR-212-3p | ENCORI |
| *COL1A1* | | hsa-miR-214-3p | ENCORI |
| *COL1A1* | | hsa-miR-218-5p | ENCORI |
| *COL1A1* | | hsa-let-7g-5p | ENCORI |
| *COL1A1* | | hsa-let-7i-5p | ENCORI |
| *COL1A1* | | hsa-miR-15b-5p | ENCORI |
| *COL1A1* | | hsa-miR-23b-3p | ENCORI |
| *COL1A1* | | hsa-miR-122-5p | ENCORI |
| *COL1A1* | | hsa-miR-128-3p | ENCORI |
| *COL1A1* | | hsa-miR-132-3p | ENCORI |
| *COL1A1* | | hsa-miR-133a-3p | ENCORI |
| *COL1A1* | | hsa-miR-138-5p | ENCORI |
| *COL1A1* | | hsa-miR-143-3p | ENCORI |
| *COL1A1* | | hsa-miR-9-5p | ENCORI |
| *COL1A1* | | hsa-miR-126-5p | ENCORI |
| *COL1A1* | | hsa-miR-146a-5p | ENCORI |
| *COL1A1* | | hsa-miR-150-5p | ENCORI |
| *COL1A1* | | hsa-miR-185-5p | ENCORI |
| *COL1A1* | | hsa-miR-186-5p | ENCORI |
| *COL1A1* | | hsa-miR-195-5p | ENCORI |
| *COL1A1* | | hsa-miR-106b-5p | ENCORI |
| *COL1A1* | | hsa-miR-29c-3p | ENCORI |
| *COL1A1* | | hsa-miR-34c-5p | ENCORI |
| *COL1A1* | | hsa-miR-376c-3p | ENCORI |
| *COL1A1* | | hsa-miR-381-3p | ENCORI |
| *COL1A1* | | hsa-miR-382-5p | ENCORI |
| *COL1A1* | | hsa-miR-328-3p | ENCORI |
| *COL1A1* | | hsa-miR-151a-3p | ENCORI |
| *COL1A1* | | hsa-miR-324-3p | ENCORI |
| *COL1A1* | | hsa-miR-338-3p | ENCORI |
| *COL1A1* | | hsa-miR-335-5p | ENCORI |
| *COL1A1* | | hsa-miR-133b | ENCORI |
| *COL1A1* | | hsa-miR-345-5p | ENCORI |
| *COL1A1* | | hsa-miR-196b-5p | ENCORI |
| *COL1A1* | | hsa-miR-424-5p | ENCORI |
| *COL1A1* | | hsa-miR-20b-5p | ENCORI |
| *COL1A1* | | hsa-miR-449a | ENCORI |
| *COL1A1* | | hsa-miR-491-5p | ENCORI |
| *COL1A1* | | hsa-miR-146b-5p | ENCORI |
| *COL1A1* | | hsa-miR-495-3p | ENCORI |
| *COL1A1* | | hsa-miR-497-5p | ENCORI |
| *COL1A1* | | hsa-miR-498 | ENCORI |
| *COL1A1* | | hsa-miR-524-5p | ENCORI |
| *COL1A1* | | hsa-miR-519d-3p | ENCORI |
| *COL1A1* | | hsa-miR-520d-5p | ENCORI |
| *COL1A1* | | hsa-miR-520g-3p | ENCORI |
| *COL1A1* | | hsa-miR-520h | ENCORI |
| *COL1A1* | | hsa-miR-522-3p | ENCORI |
| *COL1A1* | | hsa-miR-503-5p | ENCORI |
| *COL1A1* | | hsa-miR-514a-3p | ENCORI |
| *COL1A1* | | hsa-miR-545-3p | ENCORI |
| *COL1A1* | | hsa-miR-574-3p | ENCORI |
| *COL1A1* | | hsa-miR-577 | ENCORI |
| *COL1A1* | | hsa-miR-579-3p | ENCORI |
| *COL1A1* | | hsa-miR-580-3p | ENCORI |
| *COL1A1* | | hsa-miR-620 | ENCORI |
| *COL1A1* | | hsa-miR-625-5p | ENCORI |
| *COL1A1* | | hsa-miR-641 | ENCORI |
| *COL1A1* | | hsa-miR-642a-5p | ENCORI |
| *COL1A1* | | hsa-miR-650 | ENCORI |
| *COL1A1* | | hsa-miR-663a | ENCORI |
| *COL1A1* | | hsa-miR-449b-5p | ENCORI |
| *COL1A1* | | hsa-miR-654-5p | ENCORI |
| *COL1A1* | | hsa-miR-660-5p | ENCORI |
| *COL1A1* | | hsa-miR-542-3p | ENCORI |
| *COL1A1* | | hsa-miR-671-5p | ENCORI |
| *COL1A1* | | hsa-miR-129-2-3p | ENCORI |
| *COL1A1* | | hsa-miR-193a-5p | ENCORI |
| *COL1A1* | | hsa-miR-296-3p | ENCORI |
| *COL1A1* | | hsa-miR-361-3p | ENCORI |
| *COL1A1* | | hsa-miR-371a-5p | ENCORI |
| *COL1A1* | | hsa-miR-340-5p | ENCORI |
| *COL1A1* | | hsa-miR-339-3p | ENCORI |
| *COL1A1* | | hsa-miR-423-5p | ENCORI |
| *COL1A1* | | hsa-miR-501-3p | ENCORI |
| *COL1A1* | | hsa-miR-502-3p | ENCORI |
| *COL1A1* | | hsa-miR-532-3p | ENCORI |
| *COL1A1* | | hsa-miR-589-5p | ENCORI |
| *COL1A1* | | hsa-miR-616-3p | ENCORI |
| *COL1A1* | | hsa-miR-624-3p | ENCORI |
| *COL1A1* | | hsa-miR-629-5p | ENCORI |
| *COL1A1* | | hsa-miR-300 | ENCORI |
| *COL1A1* | | hsa-miR-541-3p | ENCORI |
| *COL1A1* | | hsa-miR-708-5p | ENCORI |
| *COL1A1* | | hsa-miR-665 | ENCORI |
| *COL1A1* | | hsa-miR-873-5p | ENCORI |
| *COL1A1* | | hsa-miR-543 | ENCORI |
| *COL1A1* | | hsa-miR-941 | ENCORI |
| *COL1A1* | | hsa-miR-944 | ENCORI |
| *COL1A1* | | hsa-miR-513b-5p | ENCORI |
| *COL1A1* | | hsa-miR-513c-5p | ENCORI |
| *COL1A1* | | hsa-miR-1271-5p | ENCORI |
| *COL1A1* | | hsa-miR-1301-3p | ENCORI |
| *COL1A1* | | hsa-miR-1185-5p | ENCORI |
| *COL1A1* | | hsa-miR-1179 | ENCORI |
| *COL1A1* | | hsa-miR-1286 | ENCORI |
| *COL1A1* | | hsa-miR-1287-5p | ENCORI |
| *COL1A1* | | hsa-miR-1294 | ENCORI |
| *COL1A1* | | hsa-miR-1270 | ENCORI |
| *COL1A1* | | hsa-miR-1252-5p | ENCORI |
| *COL1A1* | | hsa-miR-1197 | ENCORI |
| *COL1A1* | | hsa-miR-1913 | ENCORI |
| *COL1A1* | | hsa-miR-761 | ENCORI |
| *COL1A1* | | hsa-miR-2116-3p | ENCORI |
| *COL1A1* | | hsa-miR-3121-3p | ENCORI |
| *COL1A1* | | hsa-miR-3126-5p | ENCORI |
| *COL1A1* | | hsa-miR-3150a-3p | ENCORI |
| *COL1A1* | | hsa-miR-3179 | ENCORI |
| *COL1A1* | | hsa-miR-3184-5p | ENCORI |
| *COL1A1* | | hsa-miR-4306 | ENCORI |
| *COL1A1* | | hsa-miR-3619-5p | ENCORI |
| *COL1A1* | | hsa-miR-3918 | ENCORI |
| *COL1A1* | | hsa-miR-4428 | ENCORI |
| *COL1A1* | | hsa-miR-4458 | ENCORI |
| *COL1A1* | | hsa-miR-4500 | ENCORI |
| *COL1A1* | | hsa-miR-3173-5p | ENCORI |
| *COL1A1* | | hsa-miR-4640-5p | ENCORI |
| *COL1A1* | | hsa-miR-4644 | ENCORI |
| *COL1A1* | | hsa-miR-4726-5p | ENCORI |
| *COL1A1* | | hsa-miR-4731-5p | ENCORI |
| *COL1A1* | | hsa-miR-4739 | ENCORI |
| *COL1A1* | | hsa-miR-4766-5p | ENCORI |
| *COL1A1* | | hsa-miR-4770 | ENCORI |
| *COL1A1* | | hsa-miR-2467-3p | ENCORI |
| *COL1A1* | | hsa-miR-5194 | ENCORI |
| *COL1A1* | | hsa-miR-561-5p | ENCORI |
| *COL1A1* | | hsa-miR-6088 | ENCORI |
| *COL1A1* | | hsa-miR-129-5p | TargetScan |
| *COL1A1* | | hsa-miR-6088 | TargetScan |
| *COL1A1* | | hsa-miR-4770 | TargetScan |
| *COL1A1* | | hsa-miR-143-3p | TargetScan |
| *COL1A1* | | hsa-miR-133a-3p.2 | TargetScan |
| *COL1A1* | | hsa-miR-133b | TargetScan |
| *COL1A1* | | hsa-miR-193a-5p | TargetScan |
| *COL1A1* | | hsa-miR-196a-5p | TargetScan |
| *COL1A1* | | hsa-miR-196b-5p | TargetScan |
| *COL1A1* | | hsa-miR-4500 | TargetScan |
| *COL1A1* | | hsa-let-7g-5p | TargetScan |
| *COL1A1* | | hsa-let-7i-5p | TargetScan |
| *COL1A1* | | hsa-let-7a-5p | TargetScan |
| *COL1A1* | | hsa-let-7b-5p | TargetScan |
| *COL1A1* | | hsa-let-7c-5p | TargetScan |
| *COL1A1* | | hsa-let-7f-5p | TargetScan |
| *COL1A1* | | hsa-miR-98-5p | TargetScan |
| *COL1A1* | | hsa-let-7e-5p | TargetScan |
| *COL1A1* | | hsa-let-7d-5p | TargetScan |
| *COL1A1* | | hsa-miR-4458 | TargetScan |
| *COL1A1* | | hsa-miR-29a-3p | TargetScan |
| *COL1A1* | | hsa-miR-29b-3p | TargetScan |
| *COL1A1* | | hsa-miR-29c-3p | TargetScan |
| *COL1A1* | | hsa-miR-382-5p | TargetScan |
| *COL1A1* | | hsa-miR-218-5p | TargetScan |
| *COL1A1* | | hsa-miR-532-3p | TargetScan |
| *COL1A1* | | hsa-miR-371a-5p | TargetScan |
| *COL1A1* | | hsa-miR-338-3p | TargetScan |
| *COL1A1* | | hsa-miR-548x-3p | DIANA-micro T |
| *COL1A1* | | hsa-miR-548aj-3p | DIANA-micro T |
| *COL1A1* | | hsa-miR-651-3p | DIANA-micro T |
| *COL1A1* | | hsa-miR-4728-5p | DIANA-micro T |
| *COL1A1* | | hsa-miR-149-3p | DIANA-micro T |
| *COL1A1* | | hsa-miR-516b-3p | DIANA-micro T |
| *COL1A1* | | hsa-miR-516a-3p | DIANA-micro T |
| *COL1A1* | | hsa-miR-6785-5p | DIANA-micro T |
| *COL1A1* | | hsa-miR-4700-5p | DIANA-micro T |
| *COL1A1* | | hsa-miR-5682 | DIANA-micro T |
| *COL1A1* | | hsa-miR-7162-5p | DIANA-micro T |
| *COL1A1* | | hsa-miR-548am-3p | DIANA-micro T |
| *COL1A1* | | hsa-miR-548ah-3p | DIANA-micro T |
| *COL1A1* | | hsa-miR-6766-5p | DIANA-micro T |
| *COL1A1* | | hsa-miR-6722-3p | DIANA-micro T |
| *COL1A1* | | hsa-miR-548ae-3p | DIANA-micro T |
| *COL1A1* | | hsa-miR-548j-3p | DIANA-micro T |
| *COL1A1* | | hsa-miR-29a-3p | DIANA-micro T |
| *COL1A1* | | hsa-miR-29b-3p | DIANA-micro T |
| *COL1A1* | | hsa-miR-548aq-3p | DIANA-micro T |
| *COL1A1* | | hsa-miR-29c-3p | DIANA-micro T |
| *COL1A1* | | hsa-miR-1249-5p | DIANA-micro T |
| *COL1A1* | | hsa-miR-625-5p | DIANA-micro T |
| *COL1A1* | | hsa-miR-6763-5p | DIANA-micro T |
| *COL1A1* | | hsa-miR-2116-3p | DIANA-micro T |
| *COL1A1* | | hsa-miR-6825-5p | DIANA-micro T |
| *COL1A1* | | hsa-miR-6081 | DIANA-micro T |
| *COL1A1* | | hsa-miR-593-3p | DIANA-micro T |
| *COL1A1* | | hsa-miR-3119 | DIANA-micro T |
| *COL1A1* | | hsa-miR-1226-3p | DIANA-micro T |
| *COL1A1* | | hsa-miR-6797-5p | DIANA-micro T |
| *COL1A1* | | hsa-miR-6887-5p | DIANA-micro T |
| *COL1A1* | | hsa-miR-4733-3p | DIANA-micro T |
| *COL1A1* | | hsa-miR-3150a-3p | DIANA-micro T |
| *COL1A1* | | hsa-miR-6745 | DIANA-micro T |
| *COL1A1* | | hsa-miR-3144-5p | DIANA-micro T |
| *COL1A1* | | hsa-miR-6088 | DIANA-micro T |
| *COL1A1* | | hsa-miR-6795-5p | DIANA-micro T |
| *COL1A1* | | hsa-miR-4468 | DIANA-micro T |
| *COL1A1* | | hsa-miR-3148 | DIANA-micro T |
| *COL1A1* | | hsa-miR-6756-5p | DIANA-micro T |
| *COL1A1* | | hsa-miR-6883-5p | DIANA-micro T |
| *COL1A1* | | hsa-let-7d-5p | DIANA-micro T |
| *COL1A1* | | hsa-miR-1343-5p | DIANA-micro T |
| *COL1A1* | | hsa-miR-6798-5p | DIANA-micro T |
| *COL1A1* | | hsa-miR-6818-3p | DIANA-micro T |
| *COL1A1* | | hsa-miR-133b | DIANA-micro T |
| *COL1A1* | | hsa-miR-133a-3p | DIANA-micro T |
| *COL1A1* | | hsa-miR-3663-3p | DIANA-micro T |
| *COL1A1* | | hsa-miR-939-5p | DIANA-micro T |
| *COL1A1* | | hsa-miR-4713-5p | DIANA-micro T |
| *COL1A1* | | hsa-let-7g-5p | DIANA-micro T |
| *COL1A1* | | hsa-miR-4800-5p | DIANA-micro T |
| *COL1A1* | | hsa-miR-532-3p | DIANA-micro T |
| *COL1A1* | | hsa-miR-7110-5p | DIANA-micro T |
| *COL1A1* | | hsa-miR-6857-5p | DIANA-micro T |
| *COL1A1* | | hsa-let-7a-5p | DIANA-micro T |
| *COL1A1* | | hsa-miR-150-5p | DIANA-micro T |
| *COL1A1* | | hsa-miR-3187-5p | DIANA-micro T |
| *COL1A1* | | hsa-miR-4667-5p | DIANA-micro T |
| *COL1A1* | | hsa-miR-3175 | DIANA-micro T |
| *COL1A1* | | hsa-miR-3918 | DIANA-micro T |
| *COL1A1* | | hsa-miR-548e-3p | DIANA-micro T |
| *COL1A1* | | hsa-let-7b-5p | DIANA-micro T |
| *COL1A1* | | hsa-let-7f-5p | DIANA-micro T |
| *COL1A1* | | hsa-miR-3166 | DIANA-micro T |
| *COL1A1* | | hsa-let-7i-5p | DIANA-micro T |
| *COL1A1* | | hsa-miR-6752-5p | DIANA-micro T |
| *COL1A1* | | hsa-miR-548ac | DIANA-micro T |
| *COL1A1* | | hsa-miR-1293 | DIANA-micro T |
| *COL1A1* | | hsa-miR-6855-5p | DIANA-micro T |
| *COL1A1* | | hsa-miR-548at-5p | DIANA-micro T |
| *COL1A1* | | hsa-miR-143-3p | DIANA-micro T |
| *COL1A1* | | hsa-miR-129-5p | DIANA-micro T |
| *COL1A1* | | hsa-miR-8089 | DIANA-micro T |
| *COL1A1* | | hsa-miR-7106-5p | DIANA-micro T |
| *COL1A1* | | hsa-miR-548z | DIANA-micro T |
| *COL1A1* | | hsa-miR-548h-3p | DIANA-micro T |
| *COL1A1* | | hsa-miR-6885-5p | DIANA-micro T |
| *COL1A1* | | hsa-miR-583 | DIANA-micro T |
| *COL1A1* | | hsa-miR-3173-3p | DIANA-micro T |
| *COL1A1* | | hsa-miR-6860 | DIANA-micro T |
| *COL1A1* | | hsa-miR-1233-5p | DIANA-micro T |
| *COL1A1* | | hsa-miR-612 | DIANA-micro T |
| *COL1A1* | | hsa-miR-6749-3p | DIANA-micro T |
| *COL1A1* | | hsa-miR-6778-5p | DIANA-micro T |
| *COL1A1* | | hsa-miR-6740-5p | DIANA-micro T |
| *COL1A1* | | hsa-miR-548f-3p | DIANA-micro T |
| *COL1A1* | | hsa-miR-7112-5p | DIANA-micro T |
| *COL1A1* | | hsa-miR-6799-5p | DIANA-micro T |
| *COL1A1* | | hsa-miR-5681a | DIANA-micro T |
| *COL1A1* | | hsa-miR-548az-3p | DIANA-micro T |
| *COL1A1* | | hsa-miR-98-5p | DIANA-micro T |
| *COL1A1* | | hsa-let-7c-5p | DIANA-micro T |
| *COL1A1* | | hsa-miR-527 | DIANA-micro T |
| *COL1A1* | | hsa-miR-518a-5p | DIANA-micro T |
| *COL1A1* | | hsa-miR-4716-3p | DIANA-micro T |
| *COL1A1* | | hsa-miR-6721-5p | DIANA-micro T |
| *COL1A1* | | hsa-miR-4776-5p | DIANA-micro T |
| *COL1A1* | | hsa-miR-92a-1-5p | DIANA-micro T |
| *COL1A1* | | hsa-miR-6732-5p | DIANA-micro T |
| *COL1A1* | | hsa-miR-3184-5p | DIANA-micro T |
| *COL1A1* | | hsa-miR-6895-5p | DIANA-micro T |
| *COL1A1* | | hsa-miR-561-5p | DIANA-micro T |
| *COL1A1* | | hsa-miR-4747-5p | DIANA-micro T |
| *COL1A1* | | hsa-miR-423-5p | DIANA-micro T |
| *COL1A1* | | hsa-miR-6753-5p | DIANA-micro T |
| *COL1A1* | | hsa-miR-6830-5p | DIANA-micro T |
| *COL1A1* | | hsa-miR-6867-3p | DIANA-micro T |
| *COL1A1* | | hsa-miR-3914 | DIANA-micro T |
| *COL1A1* | | hsa-miR-761 | DIANA-micro T |
| *COL1A1* | | hsa-miR-4500 | DIANA-micro T |
| *COL1A1* | | hsa-miR-6839-3p | DIANA-micro T |
| *COL1A1* | | hsa-miR-944 | DIANA-micro T |
| *COL1A1* | | hsa-miR-466 | DIANA-micro T |
| *COL1A1* | | hsa-miR-6780a-5p | DIANA-micro T |
| *COL1A1* | | hsa-miR-6779-5p | DIANA-micro T |
| *COL1A1* | | hsa-miR-338-3p | DIANA-micro T |
| *COL1A1* | | hsa-miR-4731-5p | DIANA-micro T |
| *COL1A1* | | hsa-miR-4640-5p | DIANA-micro T |
| *COL1A1* | | hsa-miR-372-5p | DIANA-micro T |
| *COL1A1* | | hsa-miR-6845-5p | DIANA-micro T |
| *COL1A1* | | hsa-miR-6730-5p | DIANA-micro T |
| *COL1A1* | | hsa-miR-6836-5p | DIANA-micro T |
| *COL1A1* | | hsa-miR-3179 | DIANA-micro T |
| *COL1A1* | | hsa-miR-548av-3p | DIANA-micro T |
| *COL1A1* | | hsa-miR-328-5p | DIANA-micro T |
| *COL1A1* | | hsa-miR-548bb-3p | DIANA-micro T |
| *COL1A1* | | hsa-miR-6734-5p | DIANA-micro T |
| *COL1A1* | | hsa-miR-6130 | DIANA-micro T |
| *COL1A1* | | hsa-miR-548d-3p | DIANA-micro T |
| *COL1A1* | | hsa-miR-6794-5p | DIANA-micro T |
| *COL1A1* | | hsa-miR-6782-3p | DIANA-micro T |
| *COL1A1* | | hsa-miR-7109-5p | DIANA-micro T |
| *COL1A1* | | hsa-miR-4801 | DIANA-micro T |
| *COL1A1* | | hsa-miR-6090 | DIANA-micro T |
| *COL1A1* | | hsa-miR-6842-5p | DIANA-micro T |
| *COL1A1* | | hsa-miR-6132 | DIANA-micro T |
| *COL1A1* | | hsa-miR-4530 | DIANA-micro T |
| *COL1A1* | | hsa-miR-541-5p | DIANA-micro T |
| *COL1A1* | | hsa-miR-6512-3p | DIANA-micro T |
| *COL1A1* | | hsa-miR-7113-5p | DIANA-micro T |
| *COL1A1* | | hsa-miR-7155-5p | DIANA-micro T |
| *COL1A1* | | hsa-miR-3170 | DIANA-micro T |
| *COL1A1* | | hsa-miR-574-5p | DIANA-micro T |
| *COL1A1* | | hsa-miR-5194 | DIANA-micro T |
| *COL1A1* | | hsa-miR-6805-5p | DIANA-micro T |
| *COL1A1* | | hsa-miR-609 | DIANA-micro T |
| *COL1A1* | | hsa-miR-6124 | DIANA-micro T |
| *COL1A1* | | hsa-miR-4789-5p | DIANA-micro T |
| *COL1A1* | | hsa-miR-1915-3p | DIANA-micro T |
| *COL1A1* | | hsa-miR-92a-2-5p | DIANA-micro T |
| *COL1A1* | | hsa-miR-1296-3p | DIANA-micro T |
| *COL1A1* | | hsa-miR-1285-3p | DIANA-micro T |
| *COL1A1* | | hsa-miR-30b-3p | DIANA-micro T |
| *COL1A1* | | hsa-miR-4459 | DIANA-micro T |
| *COL1A1* | | hsa-miR-615-5p | DIANA-micro T |
| *COL1A1* | | hsa-miR-4481 | DIANA-micro T |
| *COL1A1* | | hsa-miR-4755-3p | DIANA-micro T |
| *COL1A1* | | hsa-miR-4428 | DIANA-micro T |
| *COL1A1* | | hsa-miR-6510-5p | DIANA-micro T |
| *COL1A1* | | hsa-miR-4779 | DIANA-micro T |
| *COL1A1* | | hsa-miR-920 | DIANA-micro T |
| *COL1A1* | | hsa-miR-548ar-3p | DIANA-micro T |
| *COL1A1* | | hsa-miR-3613-3p | DIANA-micro T |
| *COL1A1* | | hsa-miR-6720-5p | DIANA-micro T |
| *COL1A1* | | hsa-miR-3913-5p | DIANA-micro T |
| *COL1A1* | | hsa-miR-4483 | DIANA-micro T |
| *COL1A1* | | hsa-miR-634 | DIANA-micro T |
| *COL1A1* | | hsa-miR-4695-5p | DIANA-micro T |
| *COL1A1* | | hsa-miR-4450 | DIANA-micro T |
| *COL1A1* | | hsa-miR-1911-3p | DIANA-micro T |
| *COL1A1* | | hsa-miR-4726-5p | DIANA-micro T |
| *COL1A1* | | hsa-miR-2113 | DIANA-micro T |
| *COL1A1* | | hsa-miR-1273h-5p | DIANA-micro T |
| *COL1A1* | | hsa-miR-214-3p | DIANA-micro T |
| *COL1A1* | | hsa-miR-665 | DIANA-micro T |
| *COL1A1* | | hsa-miR-4789-3p | DIANA-micro T |
| *COL1A1* | | hsa-miR-4419a | DIANA-micro T |
| *COL1A1* | | hsa-miR-1252-5p | DIANA-micro T |
| *COL1A1* | | hsa-miR-3619-5p | DIANA-micro T |
| *COL1A1* | | hsa-miR-5006-5p | DIANA-micro T |
| *COL1A1* | | hsa-miR-3689b-3p | DIANA-micro T |
| *COL1A1* | | hsa-miR-3689c | DIANA-micro T |
| *COL1A1* | | hsa-miR-577 | DIANA-micro T |
| *COL1A1* | | hsa-miR-4708-3p | DIANA-micro T |
| *COL1A1* | | hsa-miR-4770 | DIANA-micro T |
| *COL1A1* | | hsa-miR-6127 | DIANA-micro T |
| *COL1A1* | | hsa-miR-5189-5p | DIANA-micro T |
| *COL1A1* | | hsa-miR-4533 | DIANA-micro T |
| *COL1A1* | | hsa-miR-8055 | DIANA-micro T |
| *COL1A1* | | hsa-miR-3689d | DIANA-micro T |
| *COL1A1* | | hsa-miR-3689a-3p | DIANA-micro T |
| *COL1A1* | | hsa-miR-28-5p | DIANA-micro T |
| *COL1A1* | | hsa-miR-3121-3p | DIANA-micro T |
| *COL1A1* | | hsa-miR-1286 | DIANA-micro T |
| *COL1A1* | | hsa-miR-3126-5p | DIANA-micro T |
| *COL1A1* | | hsa-miR-3620-3p | DIANA-micro T |
| *COL1A1* | | hsa-miR-4632-5p | DIANA-micro T |
| *COL1A1* | | hsa-let-7a-5p | miRWalk |
| *COL1A1* | | hsa-let-7a-2-3p | miRWalk |
| *COL1A1* | | hsa-let-7b-5p | miRWalk |
| *COL1A1* | | hsa-let-7c-5p | miRWalk |
| *COL1A1* | | hsa-let-7d-5p | miRWalk |
| *COL1A1* | | hsa-let-7e-5p | miRWalk |
| *COL1A1* | | hsa-let-7f-2-3p | miRWalk |
| *COL1A1* | | hsa-miR-15a-3p | miRWalk |
| *COL1A1* | | hsa-miR-18a-5p | miRWalk |
| *COL1A1* | | hsa-miR-18a-3p | miRWalk |
| *COL1A1* | | hsa-miR-19b-1-5p | miRWalk |
| *COL1A1* | | hsa-miR-19b-2-5p | miRWalk |
| *COL1A1* | | hsa-miR-20a-3p | miRWalk |
| *COL1A1* | | hsa-miR-21-3p | miRWalk |
| *COL1A1* | | hsa-miR-22-5p | miRWalk |
| *COL1A1* | | hsa-miR-22-3p | miRWalk |
| *COL1A1* | | hsa-miR-23a-5p | miRWalk |
| *COL1A1* | | hsa-miR-23a-3p | miRWalk |
| *COL1A1* | | hsa-miR-24-3p | miRWalk |
| *COL1A1* | | hsa-miR-24-2-5p | miRWalk |
| *COL1A1* | | hsa-miR-25-5p | miRWalk |
| *COL1A1* | | hsa-miR-25-3p | miRWalk |
| *COL1A1* | | hsa-miR-26b-3p | miRWalk |
| *COL1A1* | | hsa-miR-29a-3p | miRWalk |
| *COL1A1* | | hsa-miR-30a-3p | miRWalk |
| *COL1A1* | | hsa-miR-32-5p | miRWalk |
| *COL1A1* | | hsa-miR-33a-3p | miRWalk |
| *COL1A1* | | hsa-miR-92a-3p | miRWalk |
| *COL1A1* | | hsa-miR-92a-2-5p | miRWalk |
| *COL1A1* | | hsa-miR-93-5p | miRWalk |
| *COL1A1* | | hsa-miR-96-5p | miRWalk |
| *COL1A1* | | hsa-miR-100-5p | miRWalk |
| *COL1A1* | | hsa-miR-101-5p | miRWalk |
| *COL1A1* | | hsa-miR-29b-1-5p | miRWalk |
| *COL1A1* | | hsa-miR-29b-3p | miRWalk |
| *COL1A1* | | hsa-miR-103a-2-5p | miRWalk |
| *COL1A1* | | hsa-miR-103a-3p | miRWalk |
| *COL1A1* | | hsa-miR-105-5p | miRWalk |
| *COL1A1* | | hsa-miR-105-3p | miRWalk |
| *COL1A1* | | hsa-miR-107 | miRWalk |
| *COL1A1* | | hsa-miR-16-2-3p | miRWalk |
| *COL1A1* | | hsa-miR-192-3p | miRWalk |
| *COL1A1* | | hsa-miR-196a-5p | miRWalk |
| *COL1A1* | | hsa-miR-197-5p | miRWalk |
| *COL1A1* | | hsa-miR-197-3p | miRWalk |
| *COL1A1* | | hsa-miR-198 | miRWalk |
| *COL1A1* | | hsa-miR-208a-5p | miRWalk |
| *COL1A1* | | hsa-miR-129-1-3p | miRWalk |
| *COL1A1* | | hsa-miR-148a-5p | miRWalk |
| *COL1A1* | | hsa-miR-30c-2-3p | miRWalk |
| *COL1A1* | | hsa-miR-30d-3p | miRWalk |
| *COL1A1* | | hsa-miR-139-5p | miRWalk |
| *COL1A1* | | hsa-miR-139-3p | miRWalk |
| *COL1A1* | | hsa-miR-7-5p | miRWalk |
| *COL1A1* | | hsa-miR-7-2-3p | miRWalk |
| *COL1A1* | | hsa-miR-10a-5p | miRWalk |
| *COL1A1* | | hsa-miR-10b-5p | miRWalk |
| *COL1A1* | | hsa-miR-10b-3p | miRWalk |
| *COL1A1* | | hsa-miR-34a-5p | miRWalk |
| *COL1A1* | | hsa-miR-34a-3p | miRWalk |
| *COL1A1* | | hsa-miR-181a-2-3p | miRWalk |
| *COL1A1* | | hsa-miR-181b-5p | miRWalk |
| *COL1A1* | | hsa-miR-181c-3p | miRWalk |
| *COL1A1* | | hsa-miR-182-3p | miRWalk |
| *COL1A1* | | hsa-miR-183-5p | miRWalk |
| *COL1A1* | | hsa-miR-183-3p | miRWalk |
| *COL1A1* | | hsa-miR-187-5p | miRWalk |
| *COL1A1* | | hsa-miR-187-3p | miRWalk |
| *COL1A1* | | hsa-miR-204-5p | miRWalk |
| *COL1A1* | | hsa-miR-204-3p | miRWalk |
| *COL1A1* | | hsa-miR-205-5p | miRWalk |
| *COL1A1* | | hsa-miR-210-5p | miRWalk |
| *COL1A1* | | hsa-miR-210-3p | miRWalk |
| *COL1A1* | | hsa-miR-211-5p | miRWalk |
| *COL1A1* | | hsa-miR-211-3p | miRWalk |
| *COL1A1* | | hsa-miR-212-5p | miRWalk |
| *COL1A1* | | hsa-miR-212-3p | miRWalk |
| *COL1A1* | | hsa-miR-214-5p | miRWalk |
| *COL1A1* | | hsa-miR-214-3p | miRWalk |
| *COL1A1* | | hsa-miR-215-3p | miRWalk |
| *COL1A1* | | hsa-miR-216a-3p | miRWalk |
| *COL1A1* | | hsa-miR-217-3p | miRWalk |
| *COL1A1* | | hsa-miR-218-5p | miRWalk |
| *COL1A1* | | hsa-miR-218-1-3p | miRWalk |
| *COL1A1* | | hsa-miR-219a-1-3p | miRWalk |
| *COL1A1* | | hsa-miR-221-3p | miRWalk |
| *COL1A1* | | hsa-miR-222-3p | miRWalk |
| *COL1A1* | | hsa-miR-223-3p | miRWalk |
| *COL1A1* | | hsa-miR-224-5p | miRWalk |
| *COL1A1* | | hsa-miR-200b-5p | miRWalk |
| *COL1A1* | | hsa-let-7g-5p | miRWalk |
| *COL1A1* | | hsa-let-7g-3p | miRWalk |
| *COL1A1* | | hsa-let-7i-5p | miRWalk |
| *COL1A1* | | hsa-let-7i-3p | miRWalk |
| *COL1A1* | | hsa-miR-15b-5p | miRWalk |
| *COL1A1* | | hsa-miR-23b-5p | miRWalk |
| *COL1A1* | | hsa-miR-23b-3p | miRWalk |
| *COL1A1* | | hsa-miR-27b-3p | miRWalk |
| *COL1A1* | | hsa-miR-30b-5p | miRWalk |
| *COL1A1* | | hsa-miR-30b-3p | miRWalk |
| *COL1A1* | | hsa-miR-124-3p | miRWalk |
| *COL1A1* | | hsa-miR-125b-5p | miRWalk |
| *COL1A1* | | hsa-miR-125b-1-3p | miRWalk |
| *COL1A1* | | hsa-miR-128-3p | miRWalk |
| *COL1A1* | | hsa-miR-132-5p | miRWalk |
| *COL1A1* | | hsa-miR-132-3p | miRWalk |
| *COL1A1* | | hsa-miR-133a-5p | miRWalk |
| *COL1A1* | | hsa-miR-133a-3p | miRWalk |
| *COL1A1* | | hsa-miR-135a-5p | miRWalk |
| *COL1A1* | | hsa-miR-135a-3p | miRWalk |
| *COL1A1* | | hsa-miR-135a-2-3p | miRWalk |
| *COL1A1* | | hsa-miR-137-5p | miRWalk |
| *COL1A1* | | hsa-miR-138-5p | miRWalk |
| *COL1A1* | | hsa-miR-138-2-3p | miRWalk |
| *COL1A1* | | hsa-miR-140-5p | miRWalk |
| *COL1A1* | | hsa-miR-140-3p | miRWalk |
| *COL1A1* | | hsa-miR-141-5p | miRWalk |
| *COL1A1* | | hsa-miR-141-3p | miRWalk |
| *COL1A1* | | hsa-miR-143-5p | miRWalk |
| *COL1A1* | | hsa-miR-145-5p | miRWalk |
| *COL1A1* | | hsa-miR-191-3p | miRWalk |
| *COL1A1* | | hsa-miR-9-5p | miRWalk |
| *COL1A1* | | hsa-miR-125a-5p | miRWalk |
| *COL1A1* | | hsa-miR-125a-3p | miRWalk |
| *COL1A1* | | hsa-miR-125b-2-3p | miRWalk |
| *COL1A1* | | hsa-miR-127-5p | miRWalk |
| *COL1A1* | | hsa-miR-127-3p | miRWalk |
| *COL1A1* | | hsa-miR-129-2-3p | miRWalk |
| *COL1A1* | | hsa-miR-134-5p | miRWalk |
| *COL1A1* | | hsa-miR-134-3p | miRWalk |
| *COL1A1* | | hsa-miR-136-5p | miRWalk |
| *COL1A1* | | hsa-miR-136-3p | miRWalk |
| *COL1A1* | | hsa-miR-138-1-3p | miRWalk |
| *COL1A1* | | hsa-miR-146a-5p | miRWalk |
| *COL1A1* | | hsa-miR-146a-3p | miRWalk |
| *COL1A1* | | hsa-miR-149-3p | miRWalk |
| *COL1A1* | | hsa-miR-150-3p | miRWalk |
| *COL1A1* | | hsa-miR-154-5p | miRWalk |
| *COL1A1* | | hsa-miR-185-5p | miRWalk |
| *COL1A1* | | hsa-miR-185-3p | miRWalk |
| *COL1A1* | | hsa-miR-188-3p | miRWalk |
| *COL1A1* | | hsa-miR-193a-5p | miRWalk |
| *COL1A1* | | hsa-miR-193a-3p | miRWalk |
| *COL1A1* | | hsa-miR-195-5p | miRWalk |
| *COL1A1* | | hsa-miR-320a-5p | miRWalk |
| *COL1A1* | | hsa-miR-320a-3p | miRWalk |
| *COL1A1* | | hsa-miR-155-3p | miRWalk |
| *COL1A1* | | hsa-miR-128-2-5p | miRWalk |
| *COL1A1* | | hsa-miR-194-3p | miRWalk |
| *COL1A1* | | hsa-miR-106b-5p | miRWalk |
| *COL1A1* | | hsa-miR-106b-3p | miRWalk |
| *COL1A1* | | hsa-miR-29c-5p | miRWalk |
| *COL1A1* | | hsa-miR-29c-3p | miRWalk |
| *COL1A1* | | hsa-miR-30c-1-3p | miRWalk |
| *COL1A1* | | hsa-miR-200a-5p | miRWalk |
| *COL1A1* | | hsa-miR-200a-3p | miRWalk |
| *COL1A1* | | hsa-miR-302a-3p | miRWalk |
| *COL1A1* | | hsa-miR-101-2-5p | miRWalk |
| *COL1A1* | | hsa-miR-219a-2-3p | miRWalk |
| *COL1A1* | | hsa-miR-34b-5p | miRWalk |
| *COL1A1* | | hsa-miR-34b-3p | miRWalk |
| *COL1A1* | | hsa-miR-34c-5p | miRWalk |
| *COL1A1* | | hsa-miR-34c-3p | miRWalk |
| *COL1A1* | | hsa-miR-299-5p | miRWalk |
| *COL1A1* | | hsa-miR-301a-5p | miRWalk |
| *COL1A1* | | hsa-miR-99b-5p | miRWalk |
| *COL1A1* | | hsa-miR-99b-3p | miRWalk |
| *COL1A1* | | hsa-miR-296-5p | miRWalk |
| *COL1A1* | | hsa-miR-130b-5p | miRWalk |
| *COL1A1* | | hsa-miR-30e-5p | miRWalk |
| *COL1A1* | | hsa-miR-26a-2-3p | miRWalk |
| *COL1A1* | | hsa-miR-361-5p | miRWalk |
| *COL1A1* | | hsa-miR-361-3p | miRWalk |
| *COL1A1* | | hsa-miR-362-5p | miRWalk |
| *COL1A1* | | hsa-miR-363-5p | miRWalk |
| *COL1A1* | | hsa-miR-363-3p | miRWalk |
| *COL1A1* | | hsa-miR-365a-5p | miRWalk |
| *COL1A1* | | hsa-miR-365a-3p | miRWalk |
| *COL1A1* | | hsa-miR-365b-5p | miRWalk |
| *COL1A1* | | hsa-miR-365b-3p | miRWalk |
| *COL1A1* | | hsa-miR-302b-3p | miRWalk |
| *COL1A1* | | hsa-miR-302c-3p | miRWalk |
| *COL1A1* | | hsa-miR-367-3p | miRWalk |
| *COL1A1* | | hsa-miR-376c-5p | miRWalk |
| *COL1A1* | | hsa-miR-370-5p | miRWalk |
| *COL1A1* | | hsa-miR-370-3p | miRWalk |
| *COL1A1* | | hsa-miR-371a-3p | miRWalk |
| *COL1A1* | | hsa-miR-372-3p | miRWalk |
| *COL1A1* | | hsa-miR-373-5p | miRWalk |
| *COL1A1* | | hsa-miR-373-3p | miRWalk |
| *COL1A1* | | hsa-miR-375-5p | miRWalk |
| *COL1A1* | | hsa-miR-375-3p | miRWalk |
| *COL1A1* | | hsa-miR-376a-5p | miRWalk |
| *COL1A1* | | hsa-miR-377-5p | miRWalk |
| *COL1A1* | | hsa-miR-378a-5p | miRWalk |
| *COL1A1* | | hsa-miR-378a-3p | miRWalk |
| *COL1A1* | | hsa-miR-379-5p | miRWalk |
| *COL1A1* | | hsa-miR-380-5p | miRWalk |
| *COL1A1* | | hsa-miR-381-5p | miRWalk |
| *COL1A1* | | hsa-miR-381-3p | miRWalk |
| *COL1A1* | | hsa-miR-382-5p | miRWalk |
| *COL1A1* | | hsa-miR-340-3p | miRWalk |
| *COL1A1* | | hsa-miR-328-3p | miRWalk |
| *COL1A1* | | hsa-miR-342-5p | miRWalk |
| *COL1A1* | | hsa-miR-337-5p | miRWalk |
| *COL1A1* | | hsa-miR-323a-5p | miRWalk |
| *COL1A1* | | hsa-miR-323a-3p | miRWalk |
| *COL1A1* | | hsa-miR-326 | miRWalk |
| *COL1A1* | | hsa-miR-151a-5p | miRWalk |
| *COL1A1* | | hsa-miR-151a-3p | miRWalk |
| *COL1A1* | | hsa-miR-135b-5p | miRWalk |
| *COL1A1* | | hsa-miR-135b-3p | miRWalk |
| *COL1A1* | | hsa-miR-331-5p | miRWalk |
| *COL1A1* | | hsa-miR-331-3p | miRWalk |
| *COL1A1* | | hsa-miR-324-3p | miRWalk |
| *COL1A1* | | hsa-miR-338-5p | miRWalk |
| *COL1A1* | | hsa-miR-338-3p | miRWalk |
| *COL1A1* | | hsa-miR-339-5p | miRWalk |
| *COL1A1* | | hsa-miR-339-3p | miRWalk |
| *COL1A1* | | hsa-miR-335-3p | miRWalk |
| *COL1A1* | | hsa-miR-133b | miRWalk |
| *COL1A1* | | hsa-miR-325 | miRWalk |
| *COL1A1* | | hsa-miR-345-5p | miRWalk |
| *COL1A1* | | hsa-miR-345-3p | miRWalk |
| *COL1A1* | | hsa-miR-423-5p | miRWalk |
| *COL1A1* | | hsa-miR-423-3p | miRWalk |
| *COL1A1* | | hsa-miR-424-3p | miRWalk |
| *COL1A1* | | hsa-miR-425-5p | miRWalk |
| *COL1A1* | | hsa-miR-425-3p | miRWalk |
| *COL1A1* | | hsa-miR-18b-5p | miRWalk |
| *COL1A1* | | hsa-miR-18b-3p | miRWalk |
| *COL1A1* | | hsa-miR-20b-3p | miRWalk |
| *COL1A1* | | hsa-miR-448 | miRWalk |
| *COL1A1* | | hsa-miR-429 | miRWalk |
| *COL1A1* | | hsa-miR-449a | miRWalk |
| *COL1A1* | | hsa-miR-450a-1-3p | miRWalk |
| *COL1A1* | | hsa-miR-431-5p | miRWalk |
| *COL1A1* | | hsa-miR-431-3p | miRWalk |
| *COL1A1* | | hsa-miR-433-5p | miRWalk |
| *COL1A1* | | hsa-miR-433-3p | miRWalk |
| *COL1A1* | | hsa-miR-329-5p | miRWalk |
| *COL1A1* | | hsa-miR-452-5p | miRWalk |
| *COL1A1* | | hsa-miR-452-3p | miRWalk |
| *COL1A1* | | hsa-miR-409-3p | miRWalk |
| *COL1A1* | | hsa-miR-412-3p | miRWalk |
| *COL1A1* | | hsa-miR-410-5p | miRWalk |
| *COL1A1* | | hsa-miR-376b-5p | miRWalk |
| *COL1A1* | | hsa-miR-483-5p | miRWalk |
| *COL1A1* | | hsa-miR-483-3p | miRWalk |
| *COL1A1* | | hsa-miR-484 | miRWalk |
| *COL1A1* | | hsa-miR-485-5p | miRWalk |
| *COL1A1* | | hsa-miR-485-3p | miRWalk |
| *COL1A1* | | hsa-miR-486-5p | miRWalk |
| *COL1A1* | | hsa-miR-487a-5p | miRWalk |
| *COL1A1* | | hsa-miR-487a-3p | miRWalk |
| *COL1A1* | | hsa-miR-490-3p | miRWalk |
| *COL1A1* | | hsa-miR-491-5p | miRWalk |
| *COL1A1* | | hsa-miR-491-3p | miRWalk |
| *COL1A1* | | hsa-miR-511-5p | miRWalk |
| *COL1A1* | | hsa-miR-146b-5p | miRWalk |
| *COL1A1* | | hsa-miR-146b-3p | miRWalk |
| *COL1A1* | | hsa-miR-492 | miRWalk |
| *COL1A1* | | hsa-miR-493-5p | miRWalk |
| *COL1A1* | | hsa-miR-493-3p | miRWalk |
| *COL1A1* | | hsa-miR-432-3p | miRWalk |
| *COL1A1* | | hsa-miR-494-5p | miRWalk |
| *COL1A1* | | hsa-miR-495-5p | miRWalk |
| *COL1A1* | | hsa-miR-193b-5p | miRWalk |
| *COL1A1* | | hsa-miR-193b-3p | miRWalk |
| *COL1A1* | | hsa-miR-497-5p | miRWalk |
| *COL1A1* | | hsa-miR-497-3p | miRWalk |
| *COL1A1* | | hsa-miR-181d-5p | miRWalk |
| *COL1A1* | | hsa-miR-512-5p | miRWalk |
| *COL1A1* | | hsa-miR-512-3p | miRWalk |
| *COL1A1* | | hsa-miR-498-5p | miRWalk |
| *COL1A1* | | hsa-miR-498-3p | miRWalk |
| *COL1A1* | | hsa-miR-520e-3p | miRWalk |
| *COL1A1* | | hsa-miR-519e-5p | miRWalk |
| *COL1A1* | | hsa-miR-519e-3p | miRWalk |
| *COL1A1* | | hsa-miR-520f-5p | miRWalk |
| *COL1A1* | | hsa-miR-519c-5p | miRWalk |
| *COL1A1* | | hsa-miR-520a-5p | miRWalk |
| *COL1A1* | | hsa-miR-526b-5p | miRWalk |
| *COL1A1* | | hsa-miR-526b-3p | miRWalk |
| *COL1A1* | | hsa-miR-519b-5p | miRWalk |
| *COL1A1* | | hsa-miR-525-5p | miRWalk |
| *COL1A1* | | hsa-miR-525-3p | miRWalk |
| *COL1A1* | | hsa-miR-523-5p | miRWalk |
| *COL1A1* | | hsa-miR-523-3p | miRWalk |
| *COL1A1* | | hsa-miR-518f-5p | miRWalk |
| *COL1A1* | | hsa-miR-520b-5p | miRWalk |
| *COL1A1* | | hsa-miR-520b-3p | miRWalk |
| *COL1A1* | | hsa-miR-526a-5p | miRWalk |
| *COL1A1* | | hsa-miR-520c-5p | miRWalk |
| *COL1A1* | | hsa-miR-520c-3p | miRWalk |
| *COL1A1* | | hsa-miR-518c-5p | miRWalk |
| *COL1A1* | | hsa-miR-518c-3p | miRWalk |
| *COL1A1* | | hsa-miR-524-5p | miRWalk |
| *COL1A1* | | hsa-miR-524-3p | miRWalk |
| *COL1A1* | | hsa-miR-517a-3p | miRWalk |
| *COL1A1* | | hsa-miR-519d-5p | miRWalk |
| *COL1A1* | | hsa-miR-519d-3p | miRWalk |
| *COL1A1* | | hsa-miR-520d-5p | miRWalk |
| *COL1A1* | | hsa-miR-520d-3p | miRWalk |
| *COL1A1* | | hsa-miR-517b-3p | miRWalk |
| *COL1A1* | | hsa-miR-520g-5p | miRWalk |
| *COL1A1* | | hsa-miR-516b-5p | miRWalk |
| *COL1A1* | | hsa-miR-516b-3p | miRWalk |
| *COL1A1* | | hsa-miR-518e-5p | miRWalk |
| *COL1A1* | | hsa-miR-518a-5p | miRWalk |
| *COL1A1* | | hsa-miR-518a-3p | miRWalk |
| *COL1A1* | | hsa-miR-518d-5p | miRWalk |
| *COL1A1* | | hsa-miR-522-5p | miRWalk |
| *COL1A1* | | hsa-miR-519a-5p | miRWalk |
| *COL1A1* | | hsa-miR-527 | miRWalk |
| *COL1A1* | | hsa-miR-516a-5p | miRWalk |
| *COL1A1* | | hsa-miR-516a-3p | miRWalk |
| *COL1A1* | | hsa-miR-519a-2-5p | miRWalk |
| *COL1A1* | | hsa-miR-500a-5p | miRWalk |
| *COL1A1* | | hsa-miR-500a-3p | miRWalk |
| *COL1A1* | | hsa-miR-501-5p | miRWalk |
| *COL1A1* | | hsa-miR-501-3p | miRWalk |
| *COL1A1* | | hsa-miR-502-3p | miRWalk |
| *COL1A1* | | hsa-miR-503-5p | miRWalk |
| *COL1A1* | | hsa-miR-503-3p | miRWalk |
| *COL1A1* | | hsa-miR-504-5p | miRWalk |
| *COL1A1* | | hsa-miR-504-3p | miRWalk |
| *COL1A1* | | hsa-miR-505-5p | miRWalk |
| *COL1A1* | | hsa-miR-505-3p | miRWalk |
| *COL1A1* | | hsa-miR-513a-5p | miRWalk |
| *COL1A1* | | hsa-miR-513a-3p | miRWalk |
| *COL1A1* | | hsa-miR-506-5p | miRWalk |
| *COL1A1* | | hsa-miR-508-5p | miRWalk |
| *COL1A1* | | hsa-miR-509-5p | miRWalk |
| *COL1A1* | | hsa-miR-509-3p | miRWalk |
| *COL1A1* | | hsa-miR-510-3p | miRWalk |
| *COL1A1* | | hsa-miR-514a-3p | miRWalk |
| *COL1A1* | | hsa-miR-532-5p | miRWalk |
| *COL1A1* | | hsa-miR-532-3p | miRWalk |
| *COL1A1* | | hsa-miR-455-5p | miRWalk |
| *COL1A1* | | hsa-miR-455-3p | miRWalk |
| *COL1A1* | | hsa-miR-539-5p | miRWalk |
| *COL1A1* | | hsa-miR-487b-5p | miRWalk |
| *COL1A1* | | hsa-miR-487b-3p | miRWalk |
| *COL1A1* | | hsa-miR-552-5p | miRWalk |
| *COL1A1* | | hsa-miR-552-3p | miRWalk |
| *COL1A1* | | hsa-miR-92b-5p | miRWalk |
| *COL1A1* | | hsa-miR-555 | miRWalk |
| *COL1A1* | | hsa-miR-557 | miRWalk |
| *COL1A1* | | hsa-miR-558 | miRWalk |
| *COL1A1* | | hsa-miR-562 | miRWalk |
| *COL1A1* | | hsa-miR-563 | miRWalk |
| *COL1A1* | | hsa-miR-567 | miRWalk |
| *COL1A1* | | hsa-miR-551b-5p | miRWalk |
| *COL1A1* | | hsa-miR-551b-3p | miRWalk |
| *COL1A1* | | hsa-miR-570-5p | miRWalk |
| *COL1A1* | | hsa-miR-570-3p | miRWalk |
| *COL1A1* | | hsa-miR-571 | miRWalk |
| *COL1A1* | | hsa-miR-574-3p | miRWalk |
| *COL1A1* | | hsa-miR-576-5p | miRWalk |
| *COL1A1* | | hsa-miR-578 | miRWalk |
| *COL1A1* | | hsa-miR-579-5p | miRWalk |
| *COL1A1* | | hsa-miR-580-3p | miRWalk |
| *COL1A1* | | hsa-miR-581 | miRWalk |
| *COL1A1* | | hsa-miR-583 | miRWalk |
| *COL1A1* | | hsa-miR-584-5p | miRWalk |
| *COL1A1* | | hsa-miR-584-3p | miRWalk |
| *COL1A1* | | hsa-miR-585-5p | miRWalk |
| *COL1A1* | | hsa-miR-585-3p | miRWalk |
| *COL1A1* | | hsa-miR-586 | miRWalk |
| *COL1A1* | | hsa-miR-587 | miRWalk |
| *COL1A1* | | hsa-miR-548b-3p | miRWalk |
| *COL1A1* | | hsa-miR-588 | miRWalk |
| *COL1A1* | | hsa-miR-589-5p | miRWalk |
| *COL1A1* | | hsa-miR-550a-3p | miRWalk |
| *COL1A1* | | hsa-miR-591 | miRWalk |
| *COL1A1* | | hsa-miR-593-5p | miRWalk |
| *COL1A1* | | hsa-miR-593-3p | miRWalk |
| *COL1A1* | | hsa-miR-595 | miRWalk |
| *COL1A1* | | hsa-miR-596 | miRWalk |
| *COL1A1* | | hsa-miR-597-5p | miRWalk |
| *COL1A1* | | hsa-miR-597-3p | miRWalk |
| *COL1A1* | | hsa-miR-598-5p | miRWalk |
| *COL1A1* | | hsa-miR-598-3p | miRWalk |
| *COL1A1* | | hsa-miR-601 | miRWalk |
| *COL1A1* | | hsa-miR-602 | miRWalk |
| *COL1A1* | | hsa-miR-603 | miRWalk |
| *COL1A1* | | hsa-miR-605-5p | miRWalk |
| *COL1A1* | | hsa-miR-608 | miRWalk |
| *COL1A1* | | hsa-miR-609 | miRWalk |
| *COL1A1* | | hsa-miR-611 | miRWalk |
| *COL1A1* | | hsa-miR-612 | miRWalk |
| *COL1A1* | | hsa-miR-613 | miRWalk |
| *COL1A1* | | hsa-miR-614 | miRWalk |
| *COL1A1* | | hsa-miR-615-5p | miRWalk |
| *COL1A1* | | hsa-miR-615-3p | miRWalk |
| *COL1A1* | | hsa-miR-616-3p | miRWalk |
| *COL1A1* | | hsa-miR-548c-5p | miRWalk |
| *COL1A1* | | hsa-miR-617 | miRWalk |
| *COL1A1* | | hsa-miR-618 | miRWalk |
| *COL1A1* | | hsa-miR-619-5p | miRWalk |
| *COL1A1* | | hsa-miR-619-3p | miRWalk |
| *COL1A1* | | hsa-miR-620 | miRWalk |
| *COL1A1* | | hsa-miR-621 | miRWalk |
| *COL1A1* | | hsa-miR-622 | miRWalk |
| *COL1A1* | | hsa-miR-623 | miRWalk |
| *COL1A1* | | hsa-miR-624-5p | miRWalk |
| *COL1A1* | | hsa-miR-624-3p | miRWalk |
| *COL1A1* | | hsa-miR-625-5p | miRWalk |
| *COL1A1* | | hsa-miR-625-3p | miRWalk |
| *COL1A1* | | hsa-miR-627-3p | miRWalk |
| *COL1A1* | | hsa-miR-628-3p | miRWalk |
| *COL1A1* | | hsa-miR-629-5p | miRWalk |
| *COL1A1* | | hsa-miR-629-3p | miRWalk |
| *COL1A1* | | hsa-miR-630 | miRWalk |
| *COL1A1* | | hsa-miR-33b-5p | miRWalk |
| *COL1A1* | | hsa-miR-33b-3p | miRWalk |
| *COL1A1* | | hsa-miR-632 | miRWalk |
| *COL1A1* | | hsa-miR-635 | miRWalk |
| *COL1A1* | | hsa-miR-636 | miRWalk |
| *COL1A1* | | hsa-miR-637 | miRWalk |
| *COL1A1* | | hsa-miR-638 | miRWalk |
| *COL1A1* | | hsa-miR-639 | miRWalk |
| *COL1A1* | | hsa-miR-642a-5p | miRWalk |
| *COL1A1* | | hsa-miR-642a-3p | miRWalk |
| *COL1A1* | | hsa-miR-643 | miRWalk |
| *COL1A1* | | hsa-miR-644a | miRWalk |
| *COL1A1* | | hsa-miR-646 | miRWalk |
| *COL1A1* | | hsa-miR-650 | miRWalk |
| *COL1A1* | | hsa-miR-651-5p | miRWalk |
| *COL1A1* | | hsa-miR-652-5p | miRWalk |
| *COL1A1* | | hsa-miR-652-3p | miRWalk |
| *COL1A1* | | hsa-miR-548d-5p | miRWalk |
| *COL1A1* | | hsa-miR-661 | miRWalk |
| *COL1A1* | | hsa-miR-663a | miRWalk |
| *COL1A1* | | hsa-miR-449b-5p | miRWalk |
| *COL1A1* | | hsa-miR-449b-3p | miRWalk |
| *COL1A1* | | hsa-miR-656-5p | miRWalk |
| *COL1A1* | | hsa-miR-549a-5p | miRWalk |
| *COL1A1* | | hsa-miR-657 | miRWalk |
| *COL1A1* | | hsa-miR-658 | miRWalk |
| *COL1A1* | | hsa-miR-659-5p | miRWalk |
| *COL1A1* | | hsa-miR-659-3p | miRWalk |
| *COL1A1* | | hsa-miR-660-5p | miRWalk |
| *COL1A1* | | hsa-miR-660-3p | miRWalk |
| *COL1A1* | | hsa-miR-542-5p | miRWalk |
| *COL1A1* | | hsa-miR-758-5p | miRWalk |
| *COL1A1* | | hsa-miR-758-3p | miRWalk |
| *COL1A1* | | hsa-miR-1264 | miRWalk |
| *COL1A1* | | hsa-miR-671-5p | miRWalk |
| *COL1A1* | | hsa-miR-671-3p | miRWalk |
| *COL1A1* | | hsa-miR-668-5p | miRWalk |
| *COL1A1* | | hsa-miR-668-3p | miRWalk |
| *COL1A1* | | hsa-miR-550a-3-5p | miRWalk |
| *COL1A1* | | hsa-miR-767-5p | miRWalk |
| *COL1A1* | | hsa-miR-767-3p | miRWalk |
| *COL1A1* | | hsa-miR-1224-5p | miRWalk |
| *COL1A1* | | hsa-miR-1224-3p | miRWalk |
| *COL1A1* | | hsa-miR-320b | miRWalk |
| *COL1A1* | | hsa-miR-320c | miRWalk |
| *COL1A1* | | hsa-miR-1296-5p | miRWalk |
| *COL1A1* | | hsa-miR-1296-3p | miRWalk |
| *COL1A1* | | hsa-miR-1468-5p | miRWalk |
| *COL1A1* | | hsa-miR-1323 | miRWalk |
| *COL1A1* | | hsa-miR-1271-5p | miRWalk |
| *COL1A1* | | hsa-miR-1271-3p | miRWalk |
| *COL1A1* | | hsa-miR-1301-5p | miRWalk |
| *COL1A1* | | hsa-miR-1301-3p | miRWalk |
| *COL1A1* | | hsa-miR-454-5p | miRWalk |
| *COL1A1* | | hsa-miR-449c-5p | miRWalk |
| *COL1A1* | | hsa-miR-449c-3p | miRWalk |
| *COL1A1* | | hsa-miR-769-3p | miRWalk |
| *COL1A1* | | hsa-miR-766-5p | miRWalk |
| *COL1A1* | | hsa-miR-766-3p | miRWalk |
| *COL1A1* | | hsa-miR-378d | miRWalk |
| *COL1A1* | | hsa-miR-802 | miRWalk |
| *COL1A1* | | hsa-miR-1298-5p | miRWalk |
| *COL1A1* | | hsa-miR-1298-3p | miRWalk |
| *COL1A1* | | hsa-miR-2113 | miRWalk |
| *COL1A1* | | hsa-miR-764 | miRWalk |
| *COL1A1* | | hsa-miR-759 | miRWalk |
| *COL1A1* | | hsa-miR-765 | miRWalk |
| *COL1A1* | | hsa-miR-770-5p | miRWalk |
| *COL1A1* | | hsa-miR-675-5p | miRWalk |
| *COL1A1* | | hsa-miR-675-3p | miRWalk |
| *COL1A1* | | hsa-miR-298 | miRWalk |
| *COL1A1* | | hsa-miR-891a-5p | miRWalk |
| *COL1A1* | | hsa-miR-891a-3p | miRWalk |
| *COL1A1* | | hsa-miR-300 | miRWalk |
| *COL1A1* | | hsa-miR-892a | miRWalk |
| *COL1A1* | | hsa-miR-450b-3p | miRWalk |
| *COL1A1* | | hsa-miR-874-5p | miRWalk |
| *COL1A1* | | hsa-miR-874-3p | miRWalk |
| *COL1A1* | | hsa-miR-891b | miRWalk |
| *COL1A1* | | hsa-miR-888-3p | miRWalk |
| *COL1A1* | | hsa-miR-892b | miRWalk |
| *COL1A1* | | hsa-miR-541-5p | miRWalk |
| *COL1A1* | | hsa-miR-889-5p | miRWalk |
| *COL1A1* | | hsa-miR-875-5p | miRWalk |
| *COL1A1* | | hsa-miR-875-3p | miRWalk |
| *COL1A1* | | hsa-miR-876-3p | miRWalk |
| *COL1A1* | | hsa-miR-147b-3p | miRWalk |
| *COL1A1* | | hsa-miR-744-5p | miRWalk |
| *COL1A1* | | hsa-miR-885-5p | miRWalk |
| *COL1A1* | | hsa-miR-885-3p | miRWalk |
| *COL1A1* | | hsa-miR-877-5p | miRWalk |
| *COL1A1* | | hsa-miR-877-3p | miRWalk |
| *COL1A1* | | hsa-miR-887-5p | miRWalk |
| *COL1A1* | | hsa-miR-887-3p | miRWalk |
| *COL1A1* | | hsa-miR-665 | miRWalk |
| *COL1A1* | | hsa-miR-873-5p | miRWalk |
| *COL1A1* | | hsa-miR-873-3p | miRWalk |
| *COL1A1* | | hsa-miR-301b-5p | miRWalk |
| *COL1A1* | | hsa-miR-216b-3p | miRWalk |
| *COL1A1* | | hsa-miR-208b-5p | miRWalk |
| *COL1A1* | | hsa-miR-920 | miRWalk |
| *COL1A1* | | hsa-miR-921 | miRWalk |
| *COL1A1* | | hsa-miR-922 | miRWalk |
| *COL1A1* | | hsa-miR-509-3-5p | miRWalk |
| *COL1A1* | | hsa-miR-933 | miRWalk |
| *COL1A1* | | hsa-miR-935 | miRWalk |
| *COL1A1* | | hsa-miR-937-5p | miRWalk |
| *COL1A1* | | hsa-miR-937-3p | miRWalk |
| *COL1A1* | | hsa-miR-938 | miRWalk |
| *COL1A1* | | hsa-miR-939-5p | miRWalk |
| *COL1A1* | | hsa-miR-939-3p | miRWalk |
| *COL1A1* | | hsa-miR-940 | miRWalk |
| *COL1A1* | | hsa-miR-941 | miRWalk |
| *COL1A1* | | hsa-miR-942-5p | miRWalk |
| *COL1A1* | | hsa-miR-943 | miRWalk |
| *COL1A1* | | hsa-miR-1178-5p | miRWalk |
| *COL1A1* | | hsa-miR-1178-3p | miRWalk |
| *COL1A1* | | hsa-miR-1180-5p | miRWalk |
| *COL1A1* | | hsa-miR-1181 | miRWalk |
| *COL1A1* | | hsa-miR-1182 | miRWalk |
| *COL1A1* | | hsa-miR-1183 | miRWalk |
| *COL1A1* | | hsa-miR-1184 | miRWalk |
| *COL1A1* | | hsa-miR-1225-5p | miRWalk |
| *COL1A1* | | hsa-miR-1226-3p | miRWalk |
| *COL1A1* | | hsa-miR-1227-5p | miRWalk |
| *COL1A1* | | hsa-miR-1227-3p | miRWalk |
| *COL1A1* | | hsa-miR-1228-5p | miRWalk |
| *COL1A1* | | hsa-miR-1228-3p | miRWalk |
| *COL1A1* | | hsa-miR-1229-5p | miRWalk |
| *COL1A1* | | hsa-miR-1229-3p | miRWalk |
| *COL1A1* | | hsa-miR-1233-5p | miRWalk |
| *COL1A1* | | hsa-miR-1234-3p | miRWalk |
| *COL1A1* | | hsa-miR-1236-5p | miRWalk |
| *COL1A1* | | hsa-miR-1236-3p | miRWalk |
| *COL1A1* | | hsa-miR-1237-5p | miRWalk |
| *COL1A1* | | hsa-miR-1237-3p | miRWalk |
| *COL1A1* | | hsa-miR-1238-5p | miRWalk |
| *COL1A1* | | hsa-miR-1238-3p | miRWalk |
| *COL1A1* | | hsa-miR-1200 | miRWalk |
| *COL1A1* | | hsa-miR-1202 | miRWalk |
| *COL1A1* | | hsa-miR-1203 | miRWalk |
| *COL1A1* | | hsa-miR-663b | miRWalk |
| *COL1A1* | | hsa-miR-1204 | miRWalk |
| *COL1A1* | | hsa-miR-1205 | miRWalk |
| *COL1A1* | | hsa-miR-1207-5p | miRWalk |
| *COL1A1* | | hsa-miR-1207-3p | miRWalk |
| *COL1A1* | | hsa-miR-1208 | miRWalk |
| *COL1A1* | | hsa-miR-1285-5p | miRWalk |
| *COL1A1* | | hsa-miR-1285-3p | miRWalk |
| *COL1A1* | | hsa-miR-1286 | miRWalk |
| *COL1A1* | | hsa-miR-1290 | miRWalk |
| *COL1A1* | | hsa-miR-1291 | miRWalk |
| *COL1A1* | | hsa-miR-548k | miRWalk |
| *COL1A1* | | hsa-miR-1293 | miRWalk |
| *COL1A1* | | hsa-miR-1294 | miRWalk |
| *COL1A1* | | hsa-miR-1295a | miRWalk |
| *COL1A1* | | hsa-miR-1299 | miRWalk |
| *COL1A1* | | hsa-miR-1302 | miRWalk |
| *COL1A1* | | hsa-miR-1303 | miRWalk |
| *COL1A1* | | hsa-miR-1244 | miRWalk |
| *COL1A1* | | hsa-miR-1247-5p | miRWalk |
| *COL1A1* | | hsa-miR-1247-3p | miRWalk |
| *COL1A1* | | hsa-miR-1248 | miRWalk |
| *COL1A1* | | hsa-miR-1249-5p | miRWalk |
| *COL1A1* | | hsa-miR-1249-3p | miRWalk |
| *COL1A1* | | hsa-miR-1250-3p | miRWalk |
| *COL1A1* | | hsa-miR-1251-5p | miRWalk |
| *COL1A1* | | hsa-miR-1251-3p | miRWalk |
| *COL1A1* | | hsa-miR-1258 | miRWalk |
| *COL1A1* | | hsa-miR-1260a | miRWalk |
| *COL1A1* | | hsa-miR-1261 | miRWalk |
| *COL1A1* | | hsa-miR-1263 | miRWalk |
| *COL1A1* | | hsa-miR-1266-5p | miRWalk |
| *COL1A1* | | hsa-miR-1266-3p | miRWalk |
| *COL1A1* | | hsa-miR-1268a | miRWalk |
| *COL1A1* | | hsa-miR-1270 | miRWalk |
| *COL1A1* | | hsa-miR-1272 | miRWalk |
| *COL1A1* | | hsa-miR-548h-5p | miRWalk |
| *COL1A1* | | hsa-miR-1275 | miRWalk |
| *COL1A1* | | hsa-miR-1276 | miRWalk |
| *COL1A1* | | hsa-miR-1281 | miRWalk |
| *COL1A1* | | hsa-miR-1282 | miRWalk |
| *COL1A1* | | hsa-miR-1284 | miRWalk |
| *COL1A1* | | hsa-miR-1288-5p | miRWalk |
| *COL1A1* | | hsa-miR-1292-5p | miRWalk |
| *COL1A1* | | hsa-miR-1292-3p | miRWalk |
| *COL1A1* | | hsa-miR-1255b-2-3p | miRWalk |
| *COL1A1* | | hsa-miR-664a-3p | miRWalk |
| *COL1A1* | | hsa-miR-1306-5p | miRWalk |
| *COL1A1* | | hsa-miR-1306-3p | miRWalk |
| *COL1A1* | | hsa-miR-1307-5p | miRWalk |
| *COL1A1* | | hsa-miR-1307-3p | miRWalk |
| *COL1A1* | | hsa-miR-513b-5p | miRWalk |
| *COL1A1* | | hsa-miR-513c-3p | miRWalk |
| *COL1A1* | | hsa-miR-1321 | miRWalk |
| *COL1A1* | | hsa-miR-1324 | miRWalk |
| *COL1A1* | | hsa-miR-1470 | miRWalk |
| *COL1A1* | | hsa-miR-1471 | miRWalk |
| *COL1A1* | | hsa-miR-1538 | miRWalk |
| *COL1A1* | | hsa-miR-1539 | miRWalk |
| *COL1A1* | | hsa-miR-103b | miRWalk |
| *COL1A1* | | hsa-miR-320d | miRWalk |
| *COL1A1* | | hsa-miR-1825 | miRWalk |
| *COL1A1* | | hsa-miR-1908-5p | miRWalk |
| *COL1A1* | | hsa-miR-1908-3p | miRWalk |
| *COL1A1* | | hsa-miR-1909-5p | miRWalk |
| *COL1A1* | | hsa-miR-1909-3p | miRWalk |
| *COL1A1* | | hsa-miR-1910-5p | miRWalk |
| *COL1A1* | | hsa-miR-1911-5p | miRWalk |
| *COL1A1* | | hsa-miR-1911-3p | miRWalk |
| *COL1A1* | | hsa-miR-1912-5p | miRWalk |
| *COL1A1* | | hsa-miR-1912-3p | miRWalk |
| *COL1A1* | | hsa-miR-1914-3p | miRWalk |
| *COL1A1* | | hsa-miR-1915-5p | miRWalk |
| *COL1A1* | | hsa-miR-1973 | miRWalk |
| *COL1A1* | | hsa-miR-1976 | miRWalk |
| *COL1A1* | | hsa-miR-2114-5p | miRWalk |
| *COL1A1* | | hsa-miR-2114-3p | miRWalk |
| *COL1A1* | | hsa-miR-2115-3p | miRWalk |
| *COL1A1* | | hsa-miR-2116-5p | miRWalk |
| *COL1A1* | | hsa-miR-2116-3p | miRWalk |
| *COL1A1* | | hsa-miR-2276-5p | miRWalk |
| *COL1A1* | | hsa-miR-2276-3p | miRWalk |
| *COL1A1* | | hsa-miR-2277-5p | miRWalk |
| *COL1A1* | | hsa-miR-2277-3p | miRWalk |
| *COL1A1* | | hsa-miR-2278 | miRWalk |
| *COL1A1* | | hsa-miR-2681-5p | miRWalk |
| *COL1A1* | | hsa-miR-2682-3p | miRWalk |
| *COL1A1* | | hsa-miR-711 | miRWalk |
| *COL1A1* | | hsa-miR-718 | miRWalk |
| *COL1A1* | | hsa-miR-2909 | miRWalk |
| *COL1A1* | | hsa-miR-3115 | miRWalk |
| *COL1A1* | | hsa-miR-3116 | miRWalk |
| *COL1A1* | | hsa-miR-3117-3p | miRWalk |
| *COL1A1* | | hsa-miR-3119 | miRWalk |
| *COL1A1* | | hsa-miR-3120-5p | miRWalk |
| *COL1A1* | | hsa-miR-3120-3p | miRWalk |
| *COL1A1* | | hsa-miR-3121-5p | miRWalk |
| *COL1A1* | | hsa-miR-3121-3p | miRWalk |
| *COL1A1* | | hsa-miR-3122 | miRWalk |
| *COL1A1* | | hsa-miR-3124-5p | miRWalk |
| *COL1A1* | | hsa-miR-3124-3p | miRWalk |
| *COL1A1* | | hsa-miR-3125 | miRWalk |
| *COL1A1* | | hsa-miR-3126-5p | miRWalk |
| *COL1A1* | | hsa-miR-3126-3p | miRWalk |
| *COL1A1* | | hsa-miR-3127-5p | miRWalk |
| *COL1A1* | | hsa-miR-3130-5p | miRWalk |
| *COL1A1* | | hsa-miR-3130-3p | miRWalk |
| *COL1A1* | | hsa-miR-3131 | miRWalk |
| *COL1A1* | | hsa-miR-3132 | miRWalk |
| *COL1A1* | | hsa-miR-3136-3p | miRWalk |
| *COL1A1* | | hsa-miR-544b | miRWalk |
| *COL1A1* | | hsa-miR-3137 | miRWalk |
| *COL1A1* | | hsa-miR-3138 | miRWalk |
| *COL1A1* | | hsa-miR-548t-3p | miRWalk |
| *COL1A1* | | hsa-miR-3141 | miRWalk |
| *COL1A1* | | hsa-miR-3142 | miRWalk |
| *COL1A1* | | hsa-miR-3144-5p | miRWalk |
| *COL1A1* | | hsa-miR-3145-3p | miRWalk |
| *COL1A1* | | hsa-miR-1273c | miRWalk |
| *COL1A1* | | hsa-miR-3146 | miRWalk |
| *COL1A1* | | hsa-miR-3147 | miRWalk |
| *COL1A1* | | hsa-miR-3148 | miRWalk |
| *COL1A1* | | hsa-miR-3150a-5p | miRWalk |
| *COL1A1* | | hsa-miR-3150a-3p | miRWalk |
| *COL1A1* | | hsa-miR-3151-5p | miRWalk |
| *COL1A1* | | hsa-miR-3151-3p | miRWalk |
| *COL1A1* | | hsa-miR-3152-5p | miRWalk |
| *COL1A1* | | hsa-miR-3152-3p | miRWalk |
| *COL1A1* | | hsa-miR-3153 | miRWalk |
| *COL1A1* | | hsa-miR-3074-5p | miRWalk |
| *COL1A1* | | hsa-miR-3154 | miRWalk |
| *COL1A1* | | hsa-miR-3155a | miRWalk |
| *COL1A1* | | hsa-miR-3156-5p | miRWalk |
| *COL1A1* | | hsa-miR-3156-3p | miRWalk |
| *COL1A1* | | hsa-miR-3157-5p | miRWalk |
| *COL1A1* | | hsa-miR-3158-5p | miRWalk |
| *COL1A1* | | hsa-miR-3158-3p | miRWalk |
| *COL1A1* | | hsa-miR-3160-5p | miRWalk |
| *COL1A1* | | hsa-miR-3162-5p | miRWalk |
| *COL1A1* | | hsa-miR-3162-3p | miRWalk |
| *COL1A1* | | hsa-miR-3163 | miRWalk |
| *COL1A1* | | hsa-miR-3164 | miRWalk |
| *COL1A1* | | hsa-miR-3165 | miRWalk |
| *COL1A1* | | hsa-miR-3166 | miRWalk |
| *COL1A1* | | hsa-miR-1260b | miRWalk |
| *COL1A1* | | hsa-miR-3169 | miRWalk |
| *COL1A1* | | hsa-miR-3173-5p | miRWalk |
| *COL1A1* | | hsa-miR-3173-3p | miRWalk |
| *COL1A1* | | hsa-miR-1193 | miRWalk |
| *COL1A1* | | hsa-miR-323b-3p | miRWalk |
| *COL1A1* | | hsa-miR-3174 | miRWalk |
| *COL1A1* | | hsa-miR-3175 | miRWalk |
| *COL1A1* | | hsa-miR-3176 | miRWalk |
| *COL1A1* | | hsa-miR-3177-3p | miRWalk |
| *COL1A1* | | hsa-miR-3179 | miRWalk |
| *COL1A1* | | hsa-miR-3180-5p | miRWalk |
| *COL1A1* | | hsa-miR-3180-3p | miRWalk |
| *COL1A1* | | hsa-miR-548w | miRWalk |
| *COL1A1* | | hsa-miR-3181 | miRWalk |
| *COL1A1* | | hsa-miR-3183 | miRWalk |
| *COL1A1* | | hsa-miR-3184-5p | miRWalk |
| *COL1A1* | | hsa-miR-3184-3p | miRWalk |
| *COL1A1* | | hsa-miR-3185 | miRWalk |
| *COL1A1* | | hsa-miR-3065-3p | miRWalk |
| *COL1A1* | | hsa-miR-3186-5p | miRWalk |
| *COL1A1* | | hsa-miR-3187-5p | miRWalk |
| *COL1A1* | | hsa-miR-3187-3p | miRWalk |
| *COL1A1* | | hsa-miR-3188 | miRWalk |
| *COL1A1* | | hsa-miR-3189-5p | miRWalk |
| *COL1A1* | | hsa-miR-3189-3p | miRWalk |
| *COL1A1* | | hsa-miR-320e | miRWalk |
| *COL1A1* | | hsa-miR-3190-5p | miRWalk |
| *COL1A1* | | hsa-miR-3190-3p | miRWalk |
| *COL1A1* | | hsa-miR-3191-3p | miRWalk |
| *COL1A1* | | hsa-miR-3192-5p | miRWalk |
| *COL1A1* | | hsa-miR-3192-3p | miRWalk |
| *COL1A1* | | hsa-miR-3193 | miRWalk |
| *COL1A1* | | hsa-miR-3194-5p | miRWalk |
| *COL1A1* | | hsa-miR-3194-3p | miRWalk |
| *COL1A1* | | hsa-miR-3195 | miRWalk |
| *COL1A1* | | hsa-miR-3196 | miRWalk |
| *COL1A1* | | hsa-miR-3197 | miRWalk |
| *COL1A1* | | hsa-miR-3198 | miRWalk |
| *COL1A1* | | hsa-miR-3200-3p | miRWalk |
| *COL1A1* | | hsa-miR-514b-3p | miRWalk |
| *COL1A1* | | hsa-miR-4296 | miRWalk |
| *COL1A1* | | hsa-miR-4297 | miRWalk |
| *COL1A1* | | hsa-miR-4294 | miRWalk |
| *COL1A1* | | hsa-miR-4301 | miRWalk |
| *COL1A1* | | hsa-miR-4299 | miRWalk |
| *COL1A1* | | hsa-miR-4298 | miRWalk |
| *COL1A1* | | hsa-miR-4300 | miRWalk |
| *COL1A1* | | hsa-miR-4302 | miRWalk |
| *COL1A1* | | hsa-miR-4303 | miRWalk |
| *COL1A1* | | hsa-miR-4305 | miRWalk |
| *COL1A1* | | hsa-miR-4306 | miRWalk |
| *COL1A1* | | hsa-miR-4308 | miRWalk |
| *COL1A1* | | hsa-miR-4312 | miRWalk |
| *COL1A1* | | hsa-miR-4313 | miRWalk |
| *COL1A1* | | hsa-miR-4316 | miRWalk |
| *COL1A1* | | hsa-miR-4314 | miRWalk |
| *COL1A1* | | hsa-miR-4318 | miRWalk |
| *COL1A1* | | hsa-miR-4319 | miRWalk |
| *COL1A1* | | hsa-miR-4320 | miRWalk |
| *COL1A1* | | hsa-miR-4317 | miRWalk |
| *COL1A1* | | hsa-miR-4322 | miRWalk |
| *COL1A1* | | hsa-miR-4256 | miRWalk |
| *COL1A1* | | hsa-miR-4257 | miRWalk |
| *COL1A1* | | hsa-miR-4258 | miRWalk |
| *COL1A1* | | hsa-miR-4259 | miRWalk |
| *COL1A1* | | hsa-miR-4260 | miRWalk |
| *COL1A1* | | hsa-miR-4253 | miRWalk |
| *COL1A1* | | hsa-miR-4254 | miRWalk |
| *COL1A1* | | hsa-miR-4252 | miRWalk |
| *COL1A1* | | hsa-miR-4326 | miRWalk |
| *COL1A1* | | hsa-miR-4327 | miRWalk |
| *COL1A1* | | hsa-miR-4265 | miRWalk |
| *COL1A1* | | hsa-miR-4266 | miRWalk |
| *COL1A1* | | hsa-miR-4267 | miRWalk |
| *COL1A1* | | hsa-miR-2355-5p | miRWalk |
| *COL1A1* | | hsa-miR-4268 | miRWalk |
| *COL1A1* | | hsa-miR-4270 | miRWalk |
| *COL1A1* | | hsa-miR-4271 | miRWalk |
| *COL1A1* | | hsa-miR-4274 | miRWalk |
| *COL1A1* | | hsa-miR-4281 | miRWalk |
| *COL1A1* | | hsa-miR-4277 | miRWalk |
| *COL1A1* | | hsa-miR-4279 | miRWalk |
| *COL1A1* | | hsa-miR-4278 | miRWalk |
| *COL1A1* | | hsa-miR-4282 | miRWalk |
| *COL1A1* | | hsa-miR-4283 | miRWalk |
| *COL1A1* | | hsa-miR-4284 | miRWalk |
| *COL1A1* | | hsa-miR-4286 | miRWalk |
| *COL1A1* | | hsa-miR-4287 | miRWalk |
| *COL1A1* | | hsa-miR-4288 | miRWalk |
| *COL1A1* | | hsa-miR-4289 | miRWalk |
| *COL1A1* | | hsa-miR-4290 | miRWalk |
| *COL1A1* | | hsa-miR-4291 | miRWalk |
| *COL1A1* | | hsa-miR-4329 | miRWalk |
| *COL1A1* | | hsa-miR-4330 | miRWalk |
| *COL1A1* | | hsa-miR-500b-5p | miRWalk |
| *COL1A1* | | hsa-miR-500b-3p | miRWalk |
| *COL1A1* | | hsa-miR-4328 | miRWalk |
| *COL1A1* | | hsa-miR-3605-5p | miRWalk |
| *COL1A1* | | hsa-miR-3605-3p | miRWalk |
| *COL1A1* | | hsa-miR-3606-3p | miRWalk |
| *COL1A1* | | hsa-miR-3609 | miRWalk |
| *COL1A1* | | hsa-miR-3610 | miRWalk |
| *COL1A1* | | hsa-miR-3612 | miRWalk |
| *COL1A1* | | hsa-miR-3614-5p | miRWalk |
| *COL1A1* | | hsa-miR-3614-3p | miRWalk |
| *COL1A1* | | hsa-miR-3615 | miRWalk |
| *COL1A1* | | hsa-miR-3617-5p | miRWalk |
| *COL1A1* | | hsa-miR-3617-3p | miRWalk |
| *COL1A1* | | hsa-miR-3619-5p | miRWalk |
| *COL1A1* | | hsa-miR-3619-3p | miRWalk |
| *COL1A1* | | hsa-miR-3621 | miRWalk |
| *COL1A1* | | hsa-miR-3622a-5p | miRWalk |
| *COL1A1* | | hsa-miR-3622a-3p | miRWalk |
| *COL1A1* | | hsa-miR-3622b-5p | miRWalk |
| *COL1A1* | | hsa-miR-3622b-3p | miRWalk |
| *COL1A1* | | hsa-miR-3648 | miRWalk |
| *COL1A1* | | hsa-miR-3649 | miRWalk |
| *COL1A1* | | hsa-miR-3651 | miRWalk |
| *COL1A1* | | hsa-miR-3655 | miRWalk |
| *COL1A1* | | hsa-miR-3657 | miRWalk |
| *COL1A1* | | hsa-miR-3659 | miRWalk |
| *COL1A1* | | hsa-miR-3660 | miRWalk |
| *COL1A1* | | hsa-miR-3661 | miRWalk |
| *COL1A1* | | hsa-miR-3662 | miRWalk |
| *COL1A1* | | hsa-miR-3663-5p | miRWalk |
| *COL1A1* | | hsa-miR-3663-3p | miRWalk |
| *COL1A1* | | hsa-miR-3664-5p | miRWalk |
| *COL1A1* | | hsa-miR-3667-3p | miRWalk |
| *COL1A1* | | hsa-miR-3670 | miRWalk |
| *COL1A1* | | hsa-miR-3675-3p | miRWalk |
| *COL1A1* | | hsa-miR-3677-3p | miRWalk |
| *COL1A1* | | hsa-miR-3678-3p | miRWalk |
| *COL1A1* | | hsa-miR-3679-5p | miRWalk |
| *COL1A1* | | hsa-miR-3679-3p | miRWalk |
| *COL1A1* | | hsa-miR-3680-5p | miRWalk |
| *COL1A1* | | hsa-miR-3681-3p | miRWalk |
| *COL1A1* | | hsa-miR-3682-3p | miRWalk |
| *COL1A1* | | hsa-miR-3685 | miRWalk |
| *COL1A1* | | hsa-miR-3688-5p | miRWalk |
| *COL1A1* | | hsa-miR-3689a-3p | miRWalk |
| *COL1A1* | | hsa-miR-3690 | miRWalk |
| *COL1A1* | | hsa-miR-3691-3p | miRWalk |
| *COL1A1* | | hsa-miR-3692-5p | miRWalk |
| *COL1A1* | | hsa-miR-3692-3p | miRWalk |
| *COL1A1* | | hsa-miR-3714 | miRWalk |
| *COL1A1* | | hsa-miR-3180 | miRWalk |
| *COL1A1* | | hsa-miR-3907 | miRWalk |
| *COL1A1* | | hsa-miR-3689b-3p | miRWalk |
| *COL1A1* | | hsa-miR-3909 | miRWalk |
| *COL1A1* | | hsa-miR-3911 | miRWalk |
| *COL1A1* | | hsa-miR-3913-5p | miRWalk |
| *COL1A1* | | hsa-miR-3916 | miRWalk |
| *COL1A1* | | hsa-miR-3918 | miRWalk |
| *COL1A1* | | hsa-miR-3150b-5p | miRWalk |
| *COL1A1* | | hsa-miR-3150b-3p | miRWalk |
| *COL1A1* | | hsa-miR-3921 | miRWalk |
| *COL1A1* | | hsa-miR-3922-5p | miRWalk |
| *COL1A1* | | hsa-miR-3922-3p | miRWalk |
| *COL1A1* | | hsa-miR-3927-5p | miRWalk |
| *COL1A1* | | hsa-miR-3927-3p | miRWalk |
| *COL1A1* | | hsa-miR-676-3p | miRWalk |
| *COL1A1* | | hsa-miR-3928-5p | miRWalk |
| *COL1A1* | | hsa-miR-3928-3p | miRWalk |
| *COL1A1* | | hsa-miR-3929 | miRWalk |
| *COL1A1* | | hsa-miR-3934-5p | miRWalk |
| *COL1A1* | | hsa-miR-3934-3p | miRWalk |
| *COL1A1* | | hsa-miR-3935 | miRWalk |
| *COL1A1* | | hsa-miR-3936 | miRWalk |
| *COL1A1* | | hsa-miR-3937 | miRWalk |
| *COL1A1* | | hsa-miR-3938 | miRWalk |
| *COL1A1* | | hsa-miR-3939 | miRWalk |
| *COL1A1* | | hsa-miR-3940-5p | miRWalk |
| *COL1A1* | | hsa-miR-3940-3p | miRWalk |
| *COL1A1* | | hsa-miR-3943 | miRWalk |
| *COL1A1* | | hsa-miR-3944-5p | miRWalk |
| *COL1A1* | | hsa-miR-3944-3p | miRWalk |
| *COL1A1* | | hsa-miR-3945 | miRWalk |
| *COL1A1* | | hsa-miR-374c-3p | miRWalk |
| *COL1A1* | | hsa-miR-642b-5p | miRWalk |
| *COL1A1* | | hsa-miR-642b-3p | miRWalk |
| *COL1A1* | | hsa-miR-550b-2-5p | miRWalk |
| *COL1A1* | | hsa-miR-548aa | miRWalk |
| *COL1A1* | | hsa-miR-548o-5p | miRWalk |
| *COL1A1* | | hsa-miR-4418 | miRWalk |
| *COL1A1* | | hsa-miR-378f | miRWalk |
| *COL1A1* | | hsa-miR-4421 | miRWalk |
| *COL1A1* | | hsa-miR-4423-5p | miRWalk |
| *COL1A1* | | hsa-miR-4423-3p | miRWalk |
| *COL1A1* | | hsa-miR-378g | miRWalk |
| *COL1A1* | | hsa-miR-548ac | miRWalk |
| *COL1A1* | | hsa-miR-4425 | miRWalk |
| *COL1A1* | | hsa-miR-4428 | miRWalk |
| *COL1A1* | | hsa-miR-4429 | miRWalk |
| *COL1A1* | | hsa-miR-4430 | miRWalk |
| *COL1A1* | | hsa-miR-548ad-3p | miRWalk |
| *COL1A1* | | hsa-miR-4432 | miRWalk |
| *COL1A1* | | hsa-miR-4433a-5p | miRWalk |
| *COL1A1* | | hsa-miR-4433a-3p | miRWalk |
| *COL1A1* | | hsa-miR-4435 | miRWalk |
| *COL1A1* | | hsa-miR-4436a | miRWalk |
| *COL1A1* | | hsa-miR-4437 | miRWalk |
| *COL1A1* | | hsa-miR-4439 | miRWalk |
| *COL1A1* | | hsa-miR-4440 | miRWalk |
| *COL1A1* | | hsa-miR-4441 | miRWalk |
| *COL1A1* | | hsa-miR-4442 | miRWalk |
| *COL1A1* | | hsa-miR-4443 | miRWalk |
| *COL1A1* | | hsa-miR-4444 | miRWalk |
| *COL1A1* | | hsa-miR-4446-5p | miRWalk |
| *COL1A1* | | hsa-miR-4447 | miRWalk |
| *COL1A1* | | hsa-miR-4448 | miRWalk |
| *COL1A1* | | hsa-miR-4449 | miRWalk |
| *COL1A1* | | hsa-miR-4450 | miRWalk |
| *COL1A1* | | hsa-miR-548ah-3p | miRWalk |
| *COL1A1* | | hsa-miR-4452 | miRWalk |
| *COL1A1* | | hsa-miR-4453 | miRWalk |
| *COL1A1* | | hsa-miR-4454 | miRWalk |
| *COL1A1* | | hsa-miR-4456 | miRWalk |
| *COL1A1* | | hsa-miR-4457 | miRWalk |
| *COL1A1* | | hsa-miR-4458 | miRWalk |
| *COL1A1* | | hsa-miR-378h | miRWalk |
| *COL1A1* | | hsa-miR-3135b | miRWalk |
| *COL1A1* | | hsa-miR-4462 | miRWalk |
| *COL1A1* | | hsa-miR-548ai | miRWalk |
| *COL1A1* | | hsa-miR-4465 | miRWalk |
| *COL1A1* | | hsa-miR-4467 | miRWalk |
| *COL1A1* | | hsa-miR-4468 | miRWalk |
| *COL1A1* | | hsa-miR-4470 | miRWalk |
| *COL1A1* | | hsa-miR-4471 | miRWalk |
| *COL1A1* | | hsa-miR-4472 | miRWalk |
| *COL1A1* | | hsa-miR-4473 | miRWalk |
| *COL1A1* | | hsa-miR-4474-3p | miRWalk |
| *COL1A1* | | hsa-miR-4475 | miRWalk |
| *COL1A1* | | hsa-miR-4476 | miRWalk |
| *COL1A1* | | hsa-miR-4478 | miRWalk |
| *COL1A1* | | hsa-miR-3689c | miRWalk |
| *COL1A1* | | hsa-miR-3689d | miRWalk |
| *COL1A1* | | hsa-miR-3689f | miRWalk |
| *COL1A1* | | hsa-miR-4479 | miRWalk |
| *COL1A1* | | hsa-miR-3155b | miRWalk |
| *COL1A1* | | hsa-miR-4481 | miRWalk |
| *COL1A1* | | hsa-miR-4482-5p | miRWalk |
| *COL1A1* | | hsa-miR-4482-3p | miRWalk |
| *COL1A1* | | hsa-miR-4483 | miRWalk |
| *COL1A1* | | hsa-miR-4485-5p | miRWalk |
| *COL1A1* | | hsa-miR-4485-3p | miRWalk |
| *COL1A1* | | hsa-miR-4486 | miRWalk |
| *COL1A1* | | hsa-miR-4487 | miRWalk |
| *COL1A1* | | hsa-miR-4488 | miRWalk |
| *COL1A1* | | hsa-miR-4489 | miRWalk |
| *COL1A1* | | hsa-miR-4491 | miRWalk |
| *COL1A1* | | hsa-miR-4492 | miRWalk |
| *COL1A1* | | hsa-miR-4493 | miRWalk |
| *COL1A1* | | hsa-miR-4494 | miRWalk |
| *COL1A1* | | hsa-miR-4497 | miRWalk |
| *COL1A1* | | hsa-miR-4498 | miRWalk |
| *COL1A1* | | hsa-miR-4499 | miRWalk |
| *COL1A1* | | hsa-miR-4500 | miRWalk |
| *COL1A1* | | hsa-miR-4502 | miRWalk |
| *COL1A1* | | hsa-miR-4505 | miRWalk |
| *COL1A1* | | hsa-miR-2392 | miRWalk |
| *COL1A1* | | hsa-miR-4507 | miRWalk |
| *COL1A1* | | hsa-miR-4508 | miRWalk |
| *COL1A1* | | hsa-miR-4510 | miRWalk |
| *COL1A1* | | hsa-miR-4512 | miRWalk |
| *COL1A1* | | hsa-miR-4513 | miRWalk |
| *COL1A1* | | hsa-miR-4514 | miRWalk |
| *COL1A1* | | hsa-miR-4515 | miRWalk |
| *COL1A1* | | hsa-miR-4516 | miRWalk |
| *COL1A1* | | hsa-miR-4518 | miRWalk |
| *COL1A1* | | hsa-miR-4519 | miRWalk |
| *COL1A1* | | hsa-miR-4520-5p | miRWalk |
| *COL1A1* | | hsa-miR-4520-3p | miRWalk |
| *COL1A1* | | hsa-miR-4521 | miRWalk |
| *COL1A1* | | hsa-miR-4523 | miRWalk |
| *COL1A1* | | hsa-miR-4524a-3p | miRWalk |
| *COL1A1* | | hsa-miR-4525 | miRWalk |
| *COL1A1* | | hsa-miR-4526 | miRWalk |
| *COL1A1* | | hsa-miR-4529-5p | miRWalk |
| *COL1A1* | | hsa-miR-4530 | miRWalk |
| *COL1A1* | | hsa-miR-4531 | miRWalk |
| *COL1A1* | | hsa-miR-4533 | miRWalk |
| *COL1A1* | | hsa-miR-4534 | miRWalk |
| *COL1A1* | | hsa-miR-378i | miRWalk |
| *COL1A1* | | hsa-miR-4535 | miRWalk |
| *COL1A1* | | hsa-miR-548am-5p | miRWalk |
| *COL1A1* | | hsa-miR-548am-3p | miRWalk |
| *COL1A1* | | hsa-miR-1587 | miRWalk |
| *COL1A1* | | hsa-miR-4537 | miRWalk |
| *COL1A1* | | hsa-miR-4538 | miRWalk |
| *COL1A1* | | hsa-miR-4539 | miRWalk |
| *COL1A1* | | hsa-miR-4540 | miRWalk |
| *COL1A1* | | hsa-miR-3960 | miRWalk |
| *COL1A1* | | hsa-miR-3972 | miRWalk |
| *COL1A1* | | hsa-miR-3973 | miRWalk |
| *COL1A1* | | hsa-miR-3975 | miRWalk |
| *COL1A1* | | hsa-miR-3976 | miRWalk |
| *COL1A1* | | hsa-miR-3978 | miRWalk |
| *COL1A1* | | hsa-miR-4632-5p | miRWalk |
| *COL1A1* | | hsa-miR-4632-3p | miRWalk |
| *COL1A1* | | hsa-miR-4633-5p | miRWalk |
| *COL1A1* | | hsa-miR-4633-3p | miRWalk |
| *COL1A1* | | hsa-miR-4634 | miRWalk |
| *COL1A1* | | hsa-miR-4638-5p | miRWalk |
| *COL1A1* | | hsa-miR-4638-3p | miRWalk |
| *COL1A1* | | hsa-miR-4639-5p | miRWalk |
| *COL1A1* | | hsa-miR-4640-5p | miRWalk |
| *COL1A1* | | hsa-miR-4641 | miRWalk |
| *COL1A1* | | hsa-miR-4642 | miRWalk |
| *COL1A1* | | hsa-miR-4644 | miRWalk |
| *COL1A1* | | hsa-miR-4645-3p | miRWalk |
| *COL1A1* | | hsa-miR-4646-5p | miRWalk |
| *COL1A1* | | hsa-miR-4646-3p | miRWalk |
| *COL1A1* | | hsa-miR-4647 | miRWalk |
| *COL1A1* | | hsa-miR-4648 | miRWalk |
| *COL1A1* | | hsa-miR-4649-3p | miRWalk |
| *COL1A1* | | hsa-miR-4650-5p | miRWalk |
| *COL1A1* | | hsa-miR-4650-3p | miRWalk |
| *COL1A1* | | hsa-miR-4652-5p | miRWalk |
| *COL1A1* | | hsa-miR-4652-3p | miRWalk |
| *COL1A1* | | hsa-miR-4653-3p | miRWalk |
| *COL1A1* | | hsa-miR-4654 | miRWalk |
| *COL1A1* | | hsa-miR-4655-5p | miRWalk |
| *COL1A1* | | hsa-miR-4656 | miRWalk |
| *COL1A1* | | hsa-miR-4658 | miRWalk |
| *COL1A1* | | hsa-miR-4659a-5p | miRWalk |
| *COL1A1* | | hsa-miR-4659a-3p | miRWalk |
| *COL1A1* | | hsa-miR-4660 | miRWalk |
| *COL1A1* | | hsa-miR-4661-5p | miRWalk |
| *COL1A1* | | hsa-miR-4661-3p | miRWalk |
| *COL1A1* | | hsa-miR-4662a-5p | miRWalk |
| *COL1A1* | | hsa-miR-4659b-3p | miRWalk |
| *COL1A1* | | hsa-miR-4663 | miRWalk |
| *COL1A1* | | hsa-miR-4664-5p | miRWalk |
| *COL1A1* | | hsa-miR-4664-3p | miRWalk |
| *COL1A1* | | hsa-miR-4665-5p | miRWalk |
| *COL1A1* | | hsa-miR-4667-5p | miRWalk |
| *COL1A1* | | hsa-miR-4667-3p | miRWalk |
| *COL1A1* | | hsa-miR-219b-5p | miRWalk |
| *COL1A1* | | hsa-miR-219b-3p | miRWalk |
| *COL1A1* | | hsa-miR-4669 | miRWalk |
| *COL1A1* | | hsa-miR-4670-3p | miRWalk |
| *COL1A1* | | hsa-miR-4672 | miRWalk |
| *COL1A1* | | hsa-miR-4673 | miRWalk |
| *COL1A1* | | hsa-miR-4674 | miRWalk |
| *COL1A1* | | hsa-miR-4675 | miRWalk |
| *COL1A1* | | hsa-miR-4676-3p | miRWalk |
| *COL1A1* | | hsa-miR-4677-5p | miRWalk |
| *COL1A1* | | hsa-miR-4677-3p | miRWalk |
| *COL1A1* | | hsa-miR-4678 | miRWalk |
| *COL1A1* | | hsa-miR-4681 | miRWalk |
| *COL1A1* | | hsa-miR-4682 | miRWalk |
| *COL1A1* | | hsa-miR-4683 | miRWalk |
| *COL1A1* | | hsa-miR-4684-5p | miRWalk |
| *COL1A1* | | hsa-miR-4684-3p | miRWalk |
| *COL1A1* | | hsa-miR-4685-3p | miRWalk |
| *COL1A1* | | hsa-miR-4686 | miRWalk |
| *COL1A1* | | hsa-miR-4687-5p | miRWalk |
| *COL1A1* | | hsa-miR-4687-3p | miRWalk |
| *COL1A1* | | hsa-miR-1343-5p | miRWalk |
| *COL1A1* | | hsa-miR-1343-3p | miRWalk |
| *COL1A1* | | hsa-miR-4688 | miRWalk |
| *COL1A1* | | hsa-miR-4689 | miRWalk |
| *COL1A1* | | hsa-miR-4690-5p | miRWalk |
| *COL1A1* | | hsa-miR-4690-3p | miRWalk |
| *COL1A1* | | hsa-miR-4691-5p | miRWalk |
| *COL1A1* | | hsa-miR-4692 | miRWalk |
| *COL1A1* | | hsa-miR-4695-5p | miRWalk |
| *COL1A1* | | hsa-miR-4695-3p | miRWalk |
| *COL1A1* | | hsa-miR-4697-5p | miRWalk |
| *COL1A1* | | hsa-miR-4697-3p | miRWalk |
| *COL1A1* | | hsa-miR-4698 | miRWalk |
| *COL1A1* | | hsa-miR-4700-5p | miRWalk |
| *COL1A1* | | hsa-miR-4700-3p | miRWalk |
| *COL1A1* | | hsa-miR-4701-5p | miRWalk |
| *COL1A1* | | hsa-miR-4701-3p | miRWalk |
| *COL1A1* | | hsa-miR-4703-5p | miRWalk |
| *COL1A1* | | hsa-miR-4706 | miRWalk |
| *COL1A1* | | hsa-miR-4707-5p | miRWalk |
| *COL1A1* | | hsa-miR-4707-3p | miRWalk |
| *COL1A1* | | hsa-miR-4708-5p | miRWalk |
| *COL1A1* | | hsa-miR-4708-3p | miRWalk |
| *COL1A1* | | hsa-miR-4709-5p | miRWalk |
| *COL1A1* | | hsa-miR-203b-3p | miRWalk |
| *COL1A1* | | hsa-miR-4710 | miRWalk |
| *COL1A1* | | hsa-miR-4711-5p | miRWalk |
| *COL1A1* | | hsa-miR-4711-3p | miRWalk |
| *COL1A1* | | hsa-miR-4712-5p | miRWalk |
| *COL1A1* | | hsa-miR-4713-5p | miRWalk |
| *COL1A1* | | hsa-miR-4713-3p | miRWalk |
| *COL1A1* | | hsa-miR-4714-5p | miRWalk |
| *COL1A1* | | hsa-miR-4715-5p | miRWalk |
| *COL1A1* | | hsa-miR-4715-3p | miRWalk |
| *COL1A1* | | hsa-miR-4716-5p | miRWalk |
| *COL1A1* | | hsa-miR-3529-5p | miRWalk |
| *COL1A1* | | hsa-miR-4717-5p | miRWalk |
| *COL1A1* | | hsa-miR-4717-3p | miRWalk |
| *COL1A1* | | hsa-miR-4721 | miRWalk |
| *COL1A1* | | hsa-miR-4722-5p | miRWalk |
| *COL1A1* | | hsa-miR-4722-3p | miRWalk |
| *COL1A1* | | hsa-miR-4520-2-3p | miRWalk |
| *COL1A1* | | hsa-miR-4723-5p | miRWalk |
| *COL1A1* | | hsa-miR-4723-3p | miRWalk |
| *COL1A1* | | hsa-miR-4724-5p | miRWalk |
| *COL1A1* | | hsa-miR-4724-3p | miRWalk |
| *COL1A1* | | hsa-miR-4725-5p | miRWalk |
| *COL1A1* | | hsa-miR-4725-3p | miRWalk |
| *COL1A1* | | hsa-miR-4726-5p | miRWalk |
| *COL1A1* | | hsa-miR-4727-3p | miRWalk |
| *COL1A1* | | hsa-miR-4728-5p | miRWalk |
| *COL1A1* | | hsa-miR-4730 | miRWalk |
| *COL1A1* | | hsa-miR-4731-5p | miRWalk |
| *COL1A1* | | hsa-miR-4731-3p | miRWalk |
| *COL1A1* | | hsa-miR-4732-5p | miRWalk |
| *COL1A1* | | hsa-miR-4732-3p | miRWalk |
| *COL1A1* | | hsa-miR-4733-3p | miRWalk |
| *COL1A1* | | hsa-miR-4734 | miRWalk |
| *COL1A1* | | hsa-miR-4736 | miRWalk |
| *COL1A1* | | hsa-miR-4737 | miRWalk |
| *COL1A1* | | hsa-miR-3064-5p | miRWalk |
| *COL1A1* | | hsa-miR-4738-3p | miRWalk |
| *COL1A1* | | hsa-miR-4740-5p | miRWalk |
| *COL1A1* | | hsa-miR-4740-3p | miRWalk |
| *COL1A1* | | hsa-miR-4741 | miRWalk |
| *COL1A1* | | hsa-miR-4742-5p | miRWalk |
| *COL1A1* | | hsa-miR-4742-3p | miRWalk |
| *COL1A1* | | hsa-miR-4743-5p | miRWalk |
| *COL1A1* | | hsa-miR-4743-3p | miRWalk |
| *COL1A1* | | hsa-miR-4744 | miRWalk |
| *COL1A1* | | hsa-miR-122b-3p | miRWalk |
| *COL1A1* | | hsa-miR-4745-5p | miRWalk |
| *COL1A1* | | hsa-miR-4745-3p | miRWalk |
| *COL1A1* | | hsa-miR-4746-3p | miRWalk |
| *COL1A1* | | hsa-miR-4747-5p | miRWalk |
| *COL1A1* | | hsa-miR-4747-3p | miRWalk |
| *COL1A1* | | hsa-miR-4748 | miRWalk |
| *COL1A1* | | hsa-miR-4749-5p | miRWalk |
| *COL1A1* | | hsa-miR-4749-3p | miRWalk |
| *COL1A1* | | hsa-miR-4750-5p | miRWalk |
| *COL1A1* | | hsa-miR-4750-3p | miRWalk |
| *COL1A1* | | hsa-miR-4753-3p | miRWalk |
| *COL1A1* | | hsa-miR-371b-5p | miRWalk |
| *COL1A1* | | hsa-miR-4754 | miRWalk |
| *COL1A1* | | hsa-miR-4755-5p | miRWalk |
| *COL1A1* | | hsa-miR-4755-3p | miRWalk |
| *COL1A1* | | hsa-miR-4756-5p | miRWalk |
| *COL1A1* | | hsa-miR-4756-3p | miRWalk |
| *COL1A1* | | hsa-miR-4757-5p | miRWalk |
| *COL1A1* | | hsa-miR-4757-3p | miRWalk |
| *COL1A1* | | hsa-miR-4758-5p | miRWalk |
| *COL1A1* | | hsa-miR-4758-3p | miRWalk |
| *COL1A1* | | hsa-miR-4761-3p | miRWalk |
| *COL1A1* | | hsa-miR-4763-5p | miRWalk |
| *COL1A1* | | hsa-miR-4764-5p | miRWalk |
| *COL1A1* | | hsa-miR-4764-3p | miRWalk |
| *COL1A1* | | hsa-miR-4767 | miRWalk |
| *COL1A1* | | hsa-miR-4768-5p | miRWalk |
| *COL1A1* | | hsa-miR-4768-3p | miRWalk |
| *COL1A1* | | hsa-miR-4769-5p | miRWalk |
| *COL1A1* | | hsa-miR-4769-3p | miRWalk |
| *COL1A1* | | hsa-miR-4772-5p | miRWalk |
| *COL1A1* | | hsa-miR-4772-3p | miRWalk |
| *COL1A1* | | hsa-miR-4773 | miRWalk |
| *COL1A1* | | hsa-miR-4774-5p | miRWalk |
| *COL1A1* | | hsa-miR-4774-3p | miRWalk |
| *COL1A1* | | hsa-miR-4776-5p | miRWalk |
| *COL1A1* | | hsa-miR-4776-3p | miRWalk |
| *COL1A1* | | hsa-miR-4778-5p | miRWalk |
| *COL1A1* | | hsa-miR-4779 | miRWalk |
| *COL1A1* | | hsa-miR-4780 | miRWalk |
| *COL1A1* | | hsa-miR-4436b-5p | miRWalk |
| *COL1A1* | | hsa-miR-4436b-3p | miRWalk |
| *COL1A1* | | hsa-miR-4781-5p | miRWalk |
| *COL1A1* | | hsa-miR-4781-3p | miRWalk |
| *COL1A1* | | hsa-miR-4782-5p | miRWalk |
| *COL1A1* | | hsa-miR-4783-5p | miRWalk |
| *COL1A1* | | hsa-miR-4783-3p | miRWalk |
| *COL1A1* | | hsa-miR-4784 | miRWalk |
| *COL1A1* | | hsa-miR-4785 | miRWalk |
| *COL1A1* | | hsa-miR-2467-5p | miRWalk |
| *COL1A1* | | hsa-miR-2467-3p | miRWalk |
| *COL1A1* | | hsa-miR-4786-5p | miRWalk |
| *COL1A1* | | hsa-miR-4786-3p | miRWalk |
| *COL1A1* | | hsa-miR-4787-5p | miRWalk |
| *COL1A1* | | hsa-miR-4787-3p | miRWalk |
| *COL1A1* | | hsa-miR-4788 | miRWalk |
| *COL1A1* | | hsa-miR-4793-5p | miRWalk |
| *COL1A1* | | hsa-miR-4793-3p | miRWalk |
| *COL1A1* | | hsa-miR-4794 | miRWalk |
| *COL1A1* | | hsa-miR-4796-5p | miRWalk |
| *COL1A1* | | hsa-miR-4797-3p | miRWalk |
| *COL1A1* | | hsa-miR-4799-3p | miRWalk |
| *COL1A1* | | hsa-miR-4800-5p | miRWalk |
| *COL1A1* | | hsa-miR-4800-3p | miRWalk |
| *COL1A1* | | hsa-miR-4802-5p | miRWalk |
| *COL1A1* | | hsa-miR-4802-3p | miRWalk |
| *COL1A1* | | hsa-miR-4804-3p | miRWalk |
| *COL1A1* | | hsa-miR-5000-5p | miRWalk |
| *COL1A1* | | hsa-miR-5000-3p | miRWalk |
| *COL1A1* | | hsa-miR-5001-5p | miRWalk |
| *COL1A1* | | hsa-miR-5002-5p | miRWalk |
| *COL1A1* | | hsa-miR-5002-3p | miRWalk |
| *COL1A1* | | hsa-miR-5003-5p | miRWalk |
| *COL1A1* | | hsa-miR-5003-3p | miRWalk |
| *COL1A1* | | hsa-miR-5004-5p | miRWalk |
| *COL1A1* | | hsa-miR-5004-3p | miRWalk |
| *COL1A1* | | hsa-miR-548ao-5p | miRWalk |
| *COL1A1* | | hsa-miR-548ao-3p | miRWalk |
| *COL1A1* | | hsa-miR-5006-5p | miRWalk |
| *COL1A1* | | hsa-miR-5006-3p | miRWalk |
| *COL1A1* | | hsa-miR-5007-5p | miRWalk |
| *COL1A1* | | hsa-miR-5008-5p | miRWalk |
| *COL1A1* | | hsa-miR-5008-3p | miRWalk |
| *COL1A1* | | hsa-miR-5009-5p | miRWalk |
| *COL1A1* | | hsa-miR-5009-3p | miRWalk |
| *COL1A1* | | hsa-miR-5010-5p | miRWalk |
| *COL1A1* | | hsa-miR-5010-3p | miRWalk |
| *COL1A1* | | hsa-miR-5047 | miRWalk |
| *COL1A1* | | hsa-miR-5087 | miRWalk |
| *COL1A1* | | hsa-miR-5088-5p | miRWalk |
| *COL1A1* | | hsa-miR-5088-3p | miRWalk |
| *COL1A1* | | hsa-miR-5089-5p | miRWalk |
| *COL1A1* | | hsa-miR-5090 | miRWalk |
| *COL1A1* | | hsa-miR-5092 | miRWalk |
| *COL1A1* | | hsa-miR-5093 | miRWalk |
| *COL1A1* | | hsa-miR-5094 | miRWalk |
| *COL1A1* | | hsa-miR-5187-5p | miRWalk |
| *COL1A1* | | hsa-miR-5187-3p | miRWalk |
| *COL1A1* | | hsa-miR-5189-5p | miRWalk |
| *COL1A1* | | hsa-miR-5189-3p | miRWalk |
| *COL1A1* | | hsa-miR-5190 | miRWalk |
| *COL1A1* | | hsa-miR-5192 | miRWalk |
| *COL1A1* | | hsa-miR-5193 | miRWalk |
| *COL1A1* | | hsa-miR-5194 | miRWalk |
| *COL1A1* | | hsa-miR-5195-5p | miRWalk |
| *COL1A1* | | hsa-miR-5196-5p | miRWalk |
| *COL1A1* | | hsa-miR-5196-3p | miRWalk |
| *COL1A1* | | hsa-miR-4524b-5p | miRWalk |
| *COL1A1* | | hsa-miR-4524b-3p | miRWalk |
| *COL1A1* | | hsa-miR-5571-5p | miRWalk |
| *COL1A1* | | hsa-miR-5571-3p | miRWalk |
| *COL1A1* | | hsa-miR-5100 | miRWalk |
| *COL1A1* | | hsa-miR-5572 | miRWalk |
| *COL1A1* | | hsa-miR-548aq-5p | miRWalk |
| *COL1A1* | | hsa-miR-548as-5p | miRWalk |
| *COL1A1* | | hsa-miR-5579-3p | miRWalk |
| *COL1A1* | | hsa-miR-664b-5p | miRWalk |
| *COL1A1* | | hsa-miR-664b-3p | miRWalk |
| *COL1A1* | | hsa-miR-5580-5p | miRWalk |
| *COL1A1* | | hsa-miR-5584-3p | miRWalk |
| *COL1A1* | | hsa-miR-5585-5p | miRWalk |
| *COL1A1* | | hsa-miR-5585-3p | miRWalk |
| *COL1A1* | | hsa-miR-5587-5p | miRWalk |
| *COL1A1* | | hsa-miR-5587-3p | miRWalk |
| *COL1A1* | | hsa-miR-548au-3p | miRWalk |
| *COL1A1* | | hsa-miR-1295b-5p | miRWalk |
| *COL1A1* | | hsa-miR-1295b-3p | miRWalk |
| *COL1A1* | | hsa-miR-5588-5p | miRWalk |
| *COL1A1* | | hsa-miR-5588-3p | miRWalk |
| *COL1A1* | | hsa-miR-5589-5p | miRWalk |
| *COL1A1* | | hsa-miR-5589-3p | miRWalk |
| *COL1A1* | | hsa-miR-5591-5p | miRWalk |
| *COL1A1* | | hsa-miR-5591-3p | miRWalk |
| *COL1A1* | | hsa-miR-548av-3p | miRWalk |
| *COL1A1* | | hsa-miR-5681a | miRWalk |
| *COL1A1* | | hsa-miR-5682 | miRWalk |
| *COL1A1* | | hsa-miR-548aw | miRWalk |
| *COL1A1* | | hsa-miR-5683 | miRWalk |
| *COL1A1* | | hsa-miR-5684 | miRWalk |
| *COL1A1* | | hsa-miR-5685 | miRWalk |
| *COL1A1* | | hsa-miR-5692c | miRWalk |
| *COL1A1* | | hsa-miR-5689 | miRWalk |
| *COL1A1* | | hsa-miR-5691 | miRWalk |
| *COL1A1* | | hsa-miR-4666b | miRWalk |
| *COL1A1* | | hsa-miR-5694 | miRWalk |
| *COL1A1* | | hsa-miR-5698 | miRWalk |
| *COL1A1* | | hsa-miR-5699-3p | miRWalk |
| *COL1A1* | | hsa-miR-5703 | miRWalk |
| *COL1A1* | | hsa-miR-5704 | miRWalk |
| *COL1A1* | | hsa-miR-5705 | miRWalk |
| *COL1A1* | | hsa-miR-5708 | miRWalk |
| *COL1A1* | | hsa-miR-5739 | miRWalk |
| *COL1A1* | | hsa-miR-5787 | miRWalk |
| *COL1A1* | | hsa-miR-1199-5p | miRWalk |
| *COL1A1* | | hsa-miR-1199-3p | miRWalk |
| *COL1A1* | | hsa-miR-6068 | miRWalk |
| *COL1A1* | | hsa-miR-6069 | miRWalk |
| *COL1A1* | | hsa-miR-6070 | miRWalk |
| *COL1A1* | | hsa-miR-6071 | miRWalk |
| *COL1A1* | | hsa-miR-6072 | miRWalk |
| *COL1A1* | | hsa-miR-6073 | miRWalk |
| *COL1A1* | | hsa-miR-6074 | miRWalk |
| *COL1A1* | | hsa-miR-6075 | miRWalk |
| *COL1A1* | | hsa-miR-6076 | miRWalk |
| *COL1A1* | | hsa-miR-6077 | miRWalk |
| *COL1A1* | | hsa-miR-6078 | miRWalk |
| *COL1A1* | | hsa-miR-6079 | miRWalk |
| *COL1A1* | | hsa-miR-6081 | miRWalk |
| *COL1A1* | | hsa-miR-6084 | miRWalk |
| *COL1A1* | | hsa-miR-6085 | miRWalk |
| *COL1A1* | | hsa-miR-6086 | miRWalk |
| *COL1A1* | | hsa-miR-6089 | miRWalk |
| *COL1A1* | | hsa-miR-6090 | miRWalk |
| *COL1A1* | | hsa-miR-6124 | miRWalk |
| *COL1A1* | | hsa-miR-6125 | miRWalk |
| *COL1A1* | | hsa-miR-6126 | miRWalk |
| *COL1A1* | | hsa-miR-6127 | miRWalk |
| *COL1A1* | | hsa-miR-6128 | miRWalk |
| *COL1A1* | | hsa-miR-378j | miRWalk |
| *COL1A1* | | hsa-miR-6129 | miRWalk |
| *COL1A1* | | hsa-miR-6130 | miRWalk |
| *COL1A1* | | hsa-miR-6131 | miRWalk |
| *COL1A1* | | hsa-miR-6132 | miRWalk |
| *COL1A1* | | hsa-miR-6134 | miRWalk |
| *COL1A1* | | hsa-miR-6165 | miRWalk |
| *COL1A1* | | hsa-miR-6499-5p | miRWalk |
| *COL1A1* | | hsa-miR-6499-3p | miRWalk |
| *COL1A1* | | hsa-miR-548ay-3p | miRWalk |
| *COL1A1* | | hsa-miR-6500-5p | miRWalk |
| *COL1A1* | | hsa-miR-6500-3p | miRWalk |
| *COL1A1* | | hsa-miR-6501-5p | miRWalk |
| *COL1A1* | | hsa-miR-6501-3p | miRWalk |
| *COL1A1* | | hsa-miR-6503-3p | miRWalk |
| *COL1A1* | | hsa-miR-6504-5p | miRWalk |
| *COL1A1* | | hsa-miR-6504-3p | miRWalk |
| *COL1A1* | | hsa-miR-6505-5p | miRWalk |
| *COL1A1* | | hsa-miR-6505-3p | miRWalk |
| *COL1A1* | | hsa-miR-6506-5p | miRWalk |
| *COL1A1* | | hsa-miR-6507-5p | miRWalk |
| *COL1A1* | | hsa-miR-6507-3p | miRWalk |
| *COL1A1* | | hsa-miR-6508-3p | miRWalk |
| *COL1A1* | | hsa-miR-6509-5p | miRWalk |
| *COL1A1* | | hsa-miR-6509-3p | miRWalk |
| *COL1A1* | | hsa-miR-6510-5p | miRWalk |
| *COL1A1* | | hsa-miR-6511a-5p | miRWalk |
| *COL1A1* | | hsa-miR-6511a-3p | miRWalk |
| *COL1A1* | | hsa-miR-6514-3p | miRWalk |
| *COL1A1* | | hsa-miR-6515-5p | miRWalk |
| *COL1A1* | | hsa-miR-6515-3p | miRWalk |
| *COL1A1* | | hsa-miR-6715a-3p | miRWalk |
| *COL1A1* | | hsa-miR-6715b-5p | miRWalk |
| *COL1A1* | | hsa-miR-6715b-3p | miRWalk |
| *COL1A1* | | hsa-miR-6716-3p | miRWalk |
| *COL1A1* | | hsa-miR-6511b-5p | miRWalk |
| *COL1A1* | | hsa-miR-6511b-3p | miRWalk |
| *COL1A1* | | hsa-miR-6718-5p | miRWalk |
| *COL1A1* | | hsa-miR-6719-3p | miRWalk |
| *COL1A1* | | hsa-miR-6720-5p | miRWalk |
| *COL1A1* | | hsa-miR-6720-3p | miRWalk |
| *COL1A1* | | hsa-miR-6721-5p | miRWalk |
| *COL1A1* | | hsa-miR-6722-5p | miRWalk |
| *COL1A1* | | hsa-miR-6722-3p | miRWalk |
| *COL1A1* | | hsa-miR-892c-3p | miRWalk |
| *COL1A1* | | hsa-miR-6726-5p | miRWalk |
| *COL1A1* | | hsa-miR-6726-3p | miRWalk |
| *COL1A1* | | hsa-miR-6727-5p | miRWalk |
| *COL1A1* | | hsa-miR-6727-3p | miRWalk |
| *COL1A1* | | hsa-miR-6728-5p | miRWalk |
| *COL1A1* | | hsa-miR-6728-3p | miRWalk |
| *COL1A1* | | hsa-miR-6729-5p | miRWalk |
| *COL1A1* | | hsa-miR-6729-3p | miRWalk |
| *COL1A1* | | hsa-miR-6730-5p | miRWalk |
| *COL1A1* | | hsa-miR-6730-3p | miRWalk |
| *COL1A1* | | hsa-miR-6731-5p | miRWalk |
| *COL1A1* | | hsa-miR-6731-3p | miRWalk |
| *COL1A1* | | hsa-miR-6732-3p | miRWalk |
| *COL1A1* | | hsa-miR-6733-3p | miRWalk |
| *COL1A1* | | hsa-miR-6734-5p | miRWalk |
| *COL1A1* | | hsa-miR-6734-3p | miRWalk |
| *COL1A1* | | hsa-miR-6735-5p | miRWalk |
| *COL1A1* | | hsa-miR-6735-3p | miRWalk |
| *COL1A1* | | hsa-miR-6736-5p | miRWalk |
| *COL1A1* | | hsa-miR-6736-3p | miRWalk |
| *COL1A1* | | hsa-miR-6737-5p | miRWalk |
| *COL1A1* | | hsa-miR-6737-3p | miRWalk |
| *COL1A1* | | hsa-miR-6738-5p | miRWalk |
| *COL1A1* | | hsa-miR-6740-5p | miRWalk |
| *COL1A1* | | hsa-miR-6740-3p | miRWalk |
| *COL1A1* | | hsa-miR-6741-3p | miRWalk |
| *COL1A1* | | hsa-miR-6742-5p | miRWalk |
| *COL1A1* | | hsa-miR-6742-3p | miRWalk |
| *COL1A1* | | hsa-miR-6743-5p | miRWalk |
| *COL1A1* | | hsa-miR-6743-3p | miRWalk |
| *COL1A1* | | hsa-miR-6744-5p | miRWalk |
| *COL1A1* | | hsa-miR-6744-3p | miRWalk |
| *COL1A1* | | hsa-miR-6745 | miRWalk |
| *COL1A1* | | hsa-miR-6746-3p | miRWalk |
| *COL1A1* | | hsa-miR-6747-5p | miRWalk |
| *COL1A1* | | hsa-miR-6747-3p | miRWalk |
| *COL1A1* | | hsa-miR-6748-5p | miRWalk |
| *COL1A1* | | hsa-miR-6748-3p | miRWalk |
| *COL1A1* | | hsa-miR-6749-5p | miRWalk |
| *COL1A1* | | hsa-miR-6749-3p | miRWalk |
| *COL1A1* | | hsa-miR-6750-5p | miRWalk |
| *COL1A1* | | hsa-miR-6750-3p | miRWalk |
| *COL1A1* | | hsa-miR-6751-5p | miRWalk |
| *COL1A1* | | hsa-miR-6751-3p | miRWalk |
| *COL1A1* | | hsa-miR-6752-5p | miRWalk |
| *COL1A1* | | hsa-miR-6752-3p | miRWalk |
| *COL1A1* | | hsa-miR-6753-5p | miRWalk |
| *COL1A1* | | hsa-miR-6753-3p | miRWalk |
| *COL1A1* | | hsa-miR-6754-5p | miRWalk |
| *COL1A1* | | hsa-miR-6755-3p | miRWalk |
| *COL1A1* | | hsa-miR-6756-5p | miRWalk |
| *COL1A1* | | hsa-miR-6756-3p | miRWalk |
| *COL1A1* | | hsa-miR-6757-5p | miRWalk |
| *COL1A1* | | hsa-miR-6757-3p | miRWalk |
| *COL1A1* | | hsa-miR-6758-5p | miRWalk |
| *COL1A1* | | hsa-miR-6758-3p | miRWalk |
| *COL1A1* | | hsa-miR-6759-5p | miRWalk |
| *COL1A1* | | hsa-miR-6759-3p | miRWalk |
| *COL1A1* | | hsa-miR-6760-5p | miRWalk |
| *COL1A1* | | hsa-miR-6760-3p | miRWalk |
| *COL1A1* | | hsa-miR-6761-5p | miRWalk |
| *COL1A1* | | hsa-miR-6762-5p | miRWalk |
| *COL1A1* | | hsa-miR-6762-3p | miRWalk |
| *COL1A1* | | hsa-miR-6763-5p | miRWalk |
| *COL1A1* | | hsa-miR-6763-3p | miRWalk |
| *COL1A1* | | hsa-miR-6764-5p | miRWalk |
| *COL1A1* | | hsa-miR-6764-3p | miRWalk |
| *COL1A1* | | hsa-miR-6765-5p | miRWalk |
| *COL1A1* | | hsa-miR-6766-3p | miRWalk |
| *COL1A1* | | hsa-miR-6767-5p | miRWalk |
| *COL1A1* | | hsa-miR-6767-3p | miRWalk |
| *COL1A1* | | hsa-miR-6768-5p | miRWalk |
| *COL1A1* | | hsa-miR-6768-3p | miRWalk |
| *COL1A1* | | hsa-miR-6769a-5p | miRWalk |
| *COL1A1* | | hsa-miR-6769a-3p | miRWalk |
| *COL1A1* | | hsa-miR-6770-5p | miRWalk |
| *COL1A1* | | hsa-miR-6770-3p | miRWalk |
| *COL1A1* | | hsa-miR-6771-5p | miRWalk |
| *COL1A1* | | hsa-miR-6771-3p | miRWalk |
| *COL1A1* | | hsa-miR-6772-5p | miRWalk |
| *COL1A1* | | hsa-miR-6772-3p | miRWalk |
| *COL1A1* | | hsa-miR-6773-3p | miRWalk |
| *COL1A1* | | hsa-miR-6774-5p | miRWalk |
| *COL1A1* | | hsa-miR-6774-3p | miRWalk |
| *COL1A1* | | hsa-miR-6775-5p | miRWalk |
| *COL1A1* | | hsa-miR-6775-3p | miRWalk |
| *COL1A1* | | hsa-miR-6776-3p | miRWalk |
| *COL1A1* | | hsa-miR-6777-5p | miRWalk |
| *COL1A1* | | hsa-miR-6777-3p | miRWalk |
| *COL1A1* | | hsa-miR-6778-5p | miRWalk |
| *COL1A1* | | hsa-miR-6778-3p | miRWalk |
| *COL1A1* | | hsa-miR-6779-5p | miRWalk |
| *COL1A1* | | hsa-miR-6779-3p | miRWalk |
| *COL1A1* | | hsa-miR-6780a-5p | miRWalk |
| *COL1A1* | | hsa-miR-6780a-3p | miRWalk |
| *COL1A1* | | hsa-miR-6781-5p | miRWalk |
| *COL1A1* | | hsa-miR-6781-3p | miRWalk |
| *COL1A1* | | hsa-miR-6782-5p | miRWalk |
| *COL1A1* | | hsa-miR-6782-3p | miRWalk |
| *COL1A1* | | hsa-miR-6783-3p | miRWalk |
| *COL1A1* | | hsa-miR-6784-3p | miRWalk |
| *COL1A1* | | hsa-miR-6785-5p | miRWalk |
| *COL1A1* | | hsa-miR-6785-3p | miRWalk |
| *COL1A1* | | hsa-miR-6786-5p | miRWalk |
| *COL1A1* | | hsa-miR-6786-3p | miRWalk |
| *COL1A1* | | hsa-miR-6787-3p | miRWalk |
| *COL1A1* | | hsa-miR-6788-5p | miRWalk |
| *COL1A1* | | hsa-miR-6788-3p | miRWalk |
| *COL1A1* | | hsa-miR-6789-5p | miRWalk |
| *COL1A1* | | hsa-miR-6789-3p | miRWalk |
| *COL1A1* | | hsa-miR-6790-5p | miRWalk |
| *COL1A1* | | hsa-miR-6790-3p | miRWalk |
| *COL1A1* | | hsa-miR-6791-5p | miRWalk |
| *COL1A1* | | hsa-miR-6791-3p | miRWalk |
| *COL1A1* | | hsa-miR-6792-5p | miRWalk |
| *COL1A1* | | hsa-miR-6792-3p | miRWalk |
| *COL1A1* | | hsa-miR-6793-5p | miRWalk |
| *COL1A1* | | hsa-miR-6793-3p | miRWalk |
| *COL1A1* | | hsa-miR-6794-5p | miRWalk |
| *COL1A1* | | hsa-miR-6794-3p | miRWalk |
| *COL1A1* | | hsa-miR-6795-5p | miRWalk |
| *COL1A1* | | hsa-miR-6795-3p | miRWalk |
| *COL1A1* | | hsa-miR-6796-5p | miRWalk |
| *COL1A1* | | hsa-miR-6796-3p | miRWalk |
| *COL1A1* | | hsa-miR-6797-3p | miRWalk |
| *COL1A1* | | hsa-miR-6798-3p | miRWalk |
| *COL1A1* | | hsa-miR-6799-3p | miRWalk |
| *COL1A1* | | hsa-miR-6800-5p | miRWalk |
| *COL1A1* | | hsa-miR-6800-3p | miRWalk |
| *COL1A1* | | hsa-miR-6801-5p | miRWalk |
| *COL1A1* | | hsa-miR-6801-3p | miRWalk |
| *COL1A1* | | hsa-miR-6802-5p | miRWalk |
| *COL1A1* | | hsa-miR-6802-3p | miRWalk |
| *COL1A1* | | hsa-miR-6803-5p | miRWalk |
| *COL1A1* | | hsa-miR-6803-3p | miRWalk |
| *COL1A1* | | hsa-miR-6804-3p | miRWalk |
| *COL1A1* | | hsa-miR-6805-5p | miRWalk |
| *COL1A1* | | hsa-miR-6805-3p | miRWalk |
| *COL1A1* | | hsa-miR-6806-5p | miRWalk |
| *COL1A1* | | hsa-miR-6806-3p | miRWalk |
| *COL1A1* | | hsa-miR-6807-5p | miRWalk |
| *COL1A1* | | hsa-miR-6807-3p | miRWalk |
| *COL1A1* | | hsa-miR-6808-3p | miRWalk |
| *COL1A1* | | hsa-miR-6809-3p | miRWalk |
| *COL1A1* | | hsa-miR-6810-5p | miRWalk |
| *COL1A1* | | hsa-miR-6810-3p | miRWalk |
| *COL1A1* | | hsa-miR-6811-5p | miRWalk |
| *COL1A1* | | hsa-miR-6811-3p | miRWalk |
| *COL1A1* | | hsa-miR-6812-3p | miRWalk |
| *COL1A1* | | hsa-miR-6813-5p | miRWalk |
| *COL1A1* | | hsa-miR-6813-3p | miRWalk |
| *COL1A1* | | hsa-miR-6814-3p | miRWalk |
| *COL1A1* | | hsa-miR-6815-5p | miRWalk |
| *COL1A1* | | hsa-miR-6815-3p | miRWalk |
| *COL1A1* | | hsa-miR-6816-3p | miRWalk |
| *COL1A1* | | hsa-miR-6817-5p | miRWalk |
| *COL1A1* | | hsa-miR-6817-3p | miRWalk |
| *COL1A1* | | hsa-miR-6818-3p | miRWalk |
| *COL1A1* | | hsa-miR-6819-5p | miRWalk |
| *COL1A1* | | hsa-miR-6819-3p | miRWalk |
| *COL1A1* | | hsa-miR-6820-5p | miRWalk |
| *COL1A1* | | hsa-miR-6820-3p | miRWalk |
| *COL1A1* | | hsa-miR-6821-5p | miRWalk |
| *COL1A1* | | hsa-miR-6821-3p | miRWalk |
| *COL1A1* | | hsa-miR-6822-5p | miRWalk |
| *COL1A1* | | hsa-miR-6822-3p | miRWalk |
| *COL1A1* | | hsa-miR-6823-5p | miRWalk |
| *COL1A1* | | hsa-miR-6823-3p | miRWalk |
| *COL1A1* | | hsa-miR-6824-5p | miRWalk |
| *COL1A1* | | hsa-miR-6824-3p | miRWalk |
| *COL1A1* | | hsa-miR-6825-5p | miRWalk |
| *COL1A1* | | hsa-miR-6825-3p | miRWalk |
| *COL1A1* | | hsa-miR-6826-5p | miRWalk |
| *COL1A1* | | hsa-miR-6826-3p | miRWalk |
| *COL1A1* | | hsa-miR-6827-5p | miRWalk |
| *COL1A1* | | hsa-miR-6827-3p | miRWalk |
| *COL1A1* | | hsa-miR-6828-3p | miRWalk |
| *COL1A1* | | hsa-miR-6829-5p | miRWalk |
| *COL1A1* | | hsa-miR-6829-3p | miRWalk |
| *COL1A1* | | hsa-miR-6830-5p | miRWalk |
| *COL1A1* | | hsa-miR-6830-3p | miRWalk |
| *COL1A1* | | hsa-miR-6831-5p | miRWalk |
| *COL1A1* | | hsa-miR-6831-3p | miRWalk |
| *COL1A1* | | hsa-miR-6833-5p | miRWalk |
| *COL1A1* | | hsa-miR-6833-3p | miRWalk |
| *COL1A1* | | hsa-miR-6834-5p | miRWalk |
| *COL1A1* | | hsa-miR-6834-3p | miRWalk |
| *COL1A1* | | hsa-miR-6835-5p | miRWalk |
| *COL1A1* | | hsa-miR-6835-3p | miRWalk |
| *COL1A1* | | hsa-miR-6780b-5p | miRWalk |
| *COL1A1* | | hsa-miR-6780b-3p | miRWalk |
| *COL1A1* | | hsa-miR-6836-3p | miRWalk |
| *COL1A1* | | hsa-miR-6837-5p | miRWalk |
| *COL1A1* | | hsa-miR-6838-5p | miRWalk |
| *COL1A1* | | hsa-miR-6839-3p | miRWalk |
| *COL1A1* | | hsa-miR-6840-5p | miRWalk |
| *COL1A1* | | hsa-miR-6840-3p | miRWalk |
| *COL1A1* | | hsa-miR-6841-5p | miRWalk |
| *COL1A1* | | hsa-miR-6841-3p | miRWalk |
| *COL1A1* | | hsa-miR-6842-5p | miRWalk |
| *COL1A1* | | hsa-miR-6842-3p | miRWalk |
| *COL1A1* | | hsa-miR-6843-3p | miRWalk |
| *COL1A1* | | hsa-miR-6845-5p | miRWalk |
| *COL1A1* | | hsa-miR-6845-3p | miRWalk |
| *COL1A1* | | hsa-miR-6847-5p | miRWalk |
| *COL1A1* | | hsa-miR-6847-3p | miRWalk |
| *COL1A1* | | hsa-miR-6848-5p | miRWalk |
| *COL1A1* | | hsa-miR-6848-3p | miRWalk |
| *COL1A1* | | hsa-miR-6849-5p | miRWalk |
| *COL1A1* | | hsa-miR-6849-3p | miRWalk |
| *COL1A1* | | hsa-miR-6850-5p | miRWalk |
| *COL1A1* | | hsa-miR-6850-3p | miRWalk |
| *COL1A1* | | hsa-miR-6851-5p | miRWalk |
| *COL1A1* | | hsa-miR-6851-3p | miRWalk |
| *COL1A1* | | hsa-miR-6852-5p | miRWalk |
| *COL1A1* | | hsa-miR-6852-3p | miRWalk |
| *COL1A1* | | hsa-miR-6853-5p | miRWalk |
| *COL1A1* | | hsa-miR-6853-3p | miRWalk |
| *COL1A1* | | hsa-miR-6854-5p | miRWalk |
| *COL1A1* | | hsa-miR-6856-5p | miRWalk |
| *COL1A1* | | hsa-miR-6856-3p | miRWalk |
| *COL1A1* | | hsa-miR-6857-5p | miRWalk |
| *COL1A1* | | hsa-miR-6857-3p | miRWalk |
| *COL1A1* | | hsa-miR-6858-5p | miRWalk |
| *COL1A1* | | hsa-miR-6858-3p | miRWalk |
| *COL1A1* | | hsa-miR-6859-5p | miRWalk |
| *COL1A1* | | hsa-miR-6859-3p | miRWalk |
| *COL1A1* | | hsa-miR-6769b-5p | miRWalk |
| *COL1A1* | | hsa-miR-6769b-3p | miRWalk |
| *COL1A1* | | hsa-miR-6860 | miRWalk |
| *COL1A1* | | hsa-miR-6861-5p | miRWalk |
| *COL1A1* | | hsa-miR-6861-3p | miRWalk |
| *COL1A1* | | hsa-miR-6862-5p | miRWalk |
| *COL1A1* | | hsa-miR-6862-3p | miRWalk |
| *COL1A1* | | hsa-miR-6864-3p | miRWalk |
| *COL1A1* | | hsa-miR-6865-5p | miRWalk |
| *COL1A1* | | hsa-miR-6865-3p | miRWalk |
| *COL1A1* | | hsa-miR-6866-5p | miRWalk |
| *COL1A1* | | hsa-miR-6866-3p | miRWalk |
| *COL1A1* | | hsa-miR-6867-5p | miRWalk |
| *COL1A1* | | hsa-miR-6867-3p | miRWalk |
| *COL1A1* | | hsa-miR-6868-5p | miRWalk |
| *COL1A1* | | hsa-miR-6869-5p | miRWalk |
| *COL1A1* | | hsa-miR-6869-3p | miRWalk |
| *COL1A1* | | hsa-miR-6870-3p | miRWalk |
| *COL1A1* | | hsa-miR-6871-5p | miRWalk |
| *COL1A1* | | hsa-miR-6871-3p | miRWalk |
| *COL1A1* | | hsa-miR-6872-3p | miRWalk |
| *COL1A1* | | hsa-miR-6873-5p | miRWalk |
| *COL1A1* | | hsa-miR-6873-3p | miRWalk |
| *COL1A1* | | hsa-miR-6874-5p | miRWalk |
| *COL1A1* | | hsa-miR-6875-5p | miRWalk |
| *COL1A1* | | hsa-miR-6875-3p | miRWalk |
| *COL1A1* | | hsa-miR-6876-5p | miRWalk |
| *COL1A1* | | hsa-miR-6877-5p | miRWalk |
| *COL1A1* | | hsa-miR-6877-3p | miRWalk |
| *COL1A1* | | hsa-miR-6878-5p | miRWalk |
| *COL1A1* | | hsa-miR-6878-3p | miRWalk |
| *COL1A1* | | hsa-miR-6879-5p | miRWalk |
| *COL1A1* | | hsa-miR-6879-3p | miRWalk |
| *COL1A1* | | hsa-miR-6880-5p | miRWalk |
| *COL1A1* | | hsa-miR-6880-3p | miRWalk |
| *COL1A1* | | hsa-miR-6881-5p | miRWalk |
| *COL1A1* | | hsa-miR-6881-3p | miRWalk |
| *COL1A1* | | hsa-miR-6882-5p | miRWalk |
| *COL1A1* | | hsa-miR-6882-3p | miRWalk |
| *COL1A1* | | hsa-miR-6883-5p | miRWalk |
| *COL1A1* | | hsa-miR-6883-3p | miRWalk |
| *COL1A1* | | hsa-miR-6884-5p | miRWalk |
| *COL1A1* | | hsa-miR-6884-3p | miRWalk |
| *COL1A1* | | hsa-miR-6885-5p | miRWalk |
| *COL1A1* | | hsa-miR-6885-3p | miRWalk |
| *COL1A1* | | hsa-miR-6886-5p | miRWalk |
| *COL1A1* | | hsa-miR-6886-3p | miRWalk |
| *COL1A1* | | hsa-miR-6887-5p | miRWalk |
| *COL1A1* | | hsa-miR-6887-3p | miRWalk |
| *COL1A1* | | hsa-miR-6888-3p | miRWalk |
| *COL1A1* | | hsa-miR-6889-3p | miRWalk |
| *COL1A1* | | hsa-miR-6890-5p | miRWalk |
| *COL1A1* | | hsa-miR-6890-3p | miRWalk |
| *COL1A1* | | hsa-miR-6891-5p | miRWalk |
| *COL1A1* | | hsa-miR-6891-3p | miRWalk |
| *COL1A1* | | hsa-miR-6892-5p | miRWalk |
| *COL1A1* | | hsa-miR-6892-3p | miRWalk |
| *COL1A1* | | hsa-miR-6893-5p | miRWalk |
| *COL1A1* | | hsa-miR-6894-5p | miRWalk |
| *COL1A1* | | hsa-miR-6894-3p | miRWalk |
| *COL1A1* | | hsa-miR-6895-5p | miRWalk |
| *COL1A1* | | hsa-miR-6895-3p | miRWalk |
| *COL1A1* | | hsa-miR-7106-5p | miRWalk |
| *COL1A1* | | hsa-miR-7106-3p | miRWalk |
| *COL1A1* | | hsa-miR-7107-5p | miRWalk |
| *COL1A1* | | hsa-miR-7107-3p | miRWalk |
| *COL1A1* | | hsa-miR-7108-5p | miRWalk |
| *COL1A1* | | hsa-miR-7109-5p | miRWalk |
| *COL1A1* | | hsa-miR-7109-3p | miRWalk |
| *COL1A1* | | hsa-miR-7110-5p | miRWalk |
| *COL1A1* | | hsa-miR-7110-3p | miRWalk |
| *COL1A1* | | hsa-miR-7111-5p | miRWalk |
| *COL1A1* | | hsa-miR-7111-3p | miRWalk |
| *COL1A1* | | hsa-miR-7112-5p | miRWalk |
| *COL1A1* | | hsa-miR-7112-3p | miRWalk |
| *COL1A1* | | hsa-miR-7113-5p | miRWalk |
| *COL1A1* | | hsa-miR-7113-3p | miRWalk |
| *COL1A1* | | hsa-miR-7114-5p | miRWalk |
| *COL1A1* | | hsa-miR-7114-3p | miRWalk |
| *COL1A1* | | hsa-miR-7150 | miRWalk |
| *COL1A1* | | hsa-miR-7151-5p | miRWalk |
| *COL1A1* | | hsa-miR-7151-3p | miRWalk |
| *COL1A1* | | hsa-miR-7152-5p | miRWalk |
| *COL1A1* | | hsa-miR-7152-3p | miRWalk |
| *COL1A1* | | hsa-miR-7154-5p | miRWalk |
| *COL1A1* | | hsa-miR-7154-3p | miRWalk |
| *COL1A1* | | hsa-miR-7155-5p | miRWalk |
| *COL1A1* | | hsa-miR-7155-3p | miRWalk |
| *COL1A1* | | hsa-miR-7156-5p | miRWalk |
| *COL1A1* | | hsa-miR-7156-3p | miRWalk |
| *COL1A1* | | hsa-miR-7157-5p | miRWalk |
| *COL1A1* | | hsa-miR-7157-3p | miRWalk |
| *COL1A1* | | hsa-miR-7158-5p | miRWalk |
| *COL1A1* | | hsa-miR-7158-3p | miRWalk |
| *COL1A1* | | hsa-miR-7161-3p | miRWalk |
| *COL1A1* | | hsa-miR-7160-5p | miRWalk |
| *COL1A1* | | hsa-miR-7162-5p | miRWalk |
| *COL1A1* | | hsa-miR-7162-3p | miRWalk |
| *COL1A1* | | hsa-miR-7702 | miRWalk |
| *COL1A1* | | hsa-miR-7703 | miRWalk |
| *COL1A1* | | hsa-miR-7704 | miRWalk |
| *COL1A1* | | hsa-miR-7843-5p | miRWalk |
| *COL1A1* | | hsa-miR-7843-3p | miRWalk |
| *COL1A1* | | hsa-miR-4433b-5p | miRWalk |
| *COL1A1* | | hsa-miR-1273h-5p | miRWalk |
| *COL1A1* | | hsa-miR-1273h-3p | miRWalk |
| *COL1A1* | | hsa-miR-7844-5p | miRWalk |
| *COL1A1* | | hsa-miR-7845-5p | miRWalk |
| *COL1A1* | | hsa-miR-7846-3p | miRWalk |
| *COL1A1* | | hsa-miR-7847-3p | miRWalk |
| *COL1A1* | | hsa-miR-7848-3p | miRWalk |
| *COL1A1* | | hsa-miR-7850-5p | miRWalk |
| *COL1A1* | | hsa-miR-7851-3p | miRWalk |
| *COL1A1* | | hsa-miR-7854-3p | miRWalk |
| *COL1A1* | | hsa-miR-7855-5p | miRWalk |
| *COL1A1* | | hsa-miR-7973 | miRWalk |
| *COL1A1* | | hsa-miR-7974 | miRWalk |
| *COL1A1* | | hsa-miR-7976 | miRWalk |
| *COL1A1* | | hsa-miR-7977 | miRWalk |
| *COL1A1* | | hsa-miR-7978 | miRWalk |
| *COL1A1* | | hsa-miR-8053 | miRWalk |
| *COL1A1* | | hsa-miR-8055 | miRWalk |
| *COL1A1* | | hsa-miR-8057 | miRWalk |
| *COL1A1* | | hsa-miR-8058 | miRWalk |
| *COL1A1* | | hsa-miR-8060 | miRWalk |
| *COL1A1* | | hsa-miR-8063 | miRWalk |
| *COL1A1* | | hsa-miR-8064 | miRWalk |
| *COL1A1* | | hsa-miR-8065 | miRWalk |
| *COL1A1* | | hsa-miR-8066 | miRWalk |
| *COL1A1* | | hsa-miR-8069 | miRWalk |
| *COL1A1* | | hsa-miR-8070 | miRWalk |
| *COL1A1* | | hsa-miR-8071 | miRWalk |
| *COL1A1* | | hsa-miR-8072 | miRWalk |
| *COL1A1* | | hsa-miR-8073 | miRWalk |
| *COL1A1* | | hsa-miR-8075 | miRWalk |
| *COL1A1* | | hsa-miR-8077 | miRWalk |
| *COL1A1* | | hsa-miR-8078 | miRWalk |
| *COL1A1* | | hsa-miR-8079 | miRWalk |
| *COL1A1* | | hsa-miR-8081 | miRWalk |
| *COL1A1* | | hsa-miR-8082 | miRWalk |
| *COL1A1* | | hsa-miR-8083 | miRWalk |
| *COL1A1* | | hsa-miR-8085 | miRWalk |
| *COL1A1* | | hsa-miR-8086 | miRWalk |
| *COL1A1* | | hsa-miR-8087 | miRWalk |
| *COL1A1* | | hsa-miR-8088 | miRWalk |
| *COL1A1* | | hsa-miR-8089 | miRWalk |
| *COL1A1* | | hsa-miR-8485 | miRWalk |
| *COL1A1* | | hsa-miR-9718 | miRWalk |
| *COL1A1* | | hsa-miR-9899 | miRWalk |
| *COL1A1* | | hsa-miR-9901 | miRWalk |
| *COL1A1* | | hsa-miR-9903 | miRWalk |
| *COL1A1* | | hsa-miR-9985 | miRWalk |
| *COL1A1* | | hsa-miR-1843 | miRWalk |
| *COL1A1* | | hsa-miR-9986 | miRWalk |
| *COL1A1* | | hsa-miR-10226 | miRWalk |
| *COL1A1* | | hsa-miR-10392-5p | miRWalk |
| *COL1A1* | | hsa-miR-10392-3p | miRWalk |
| *COL1A1* | | hsa-miR-10393-3p | miRWalk |
| *COL1A1* | | hsa-miR-10394-5p | miRWalk |
| *COL1A1* | | hsa-miR-10394-3p | miRWalk |
| *COL1A1* | | hsa-miR-10395-3p | miRWalk |
| *COL1A1* | | hsa-miR-10396a-5p | miRWalk |
| *COL1A1* | | hsa-miR-10396a-3p | miRWalk |
| *COL1A1* | | hsa-miR-10397-5p | miRWalk |
| *COL1A1* | | hsa-miR-10397-3p | miRWalk |
| *COL1A1* | | hsa-miR-10398-5p | miRWalk |
| *COL1A1* | | hsa-miR-10398-3p | miRWalk |
| *COL1A1* | | hsa-miR-10400-5p | miRWalk |
| *COL1A1* | | hsa-miR-10400-3p | miRWalk |
| *COL1A1* | | hsa-miR-10401-5p | miRWalk |
| *COL1A1* | | hsa-miR-10401-3p | miRWalk |
| *COL1A1* | | hsa-miR-10396b-5p | miRWalk |
| *COL1A1* | | hsa-miR-9983-3p | miRWalk |
| *COL1A1* | | hsa-miR-10524-5p | miRWalk |
| *COL1A1* | | hsa-miR-10526-3p | miRWalk |
| *COL1A1* | | hsa-miR-11181-5p | miRWalk |
| *COL1A1* | | hsa-miR-11181-3p | miRWalk |
| *COL1A1* | | hsa-miR-11399 | miRWalk |
| *COL1A1* | | hsa-miR-11400 | miRWalk |
| *COL1A1* | | hsa-miR-3059-5p | miRWalk |
| *COL1A1* | | hsa-miR-3059-3p | miRWalk |
| *COL1A1* | | hsa-miR-3085-5p | miRWalk |
| *COL1A1* | | hsa-miR-3085-3p | miRWalk |
| *COL1A1* | | hsa-miR-6529-5p | miRWalk |
| *COL1A1* | | hsa-miR-9851-5p | miRWalk |
| *COL1A1* | | hsa-miR-9851-3p | miRWalk |
| *COL1A1* | | hsa-miR-12113 | miRWalk |
| *COL1A1* | | hsa-miR-12114 | miRWalk |
| *COL1A1* | | hsa-miR-12115 | miRWalk |
| *COL1A1* | | hsa-miR-12116 | miRWalk |
| *COL1A1* | | hsa-miR-12117 | miRWalk |
| *COL1A1* | | hsa-miR-12118 | miRWalk |
| *COL1A1* | | hsa-miR-12119 | miRWalk |
| *COL1A1* | | hsa-miR-12120 | miRWalk |
| *COL1A1* | | hsa-miR-12121 | miRWalk |
| *COL1A1* | | hsa-miR-12122 | miRWalk |
| *COL1A1* | | hsa-miR-12124 | miRWalk |
| *COL1A1* | | hsa-miR-12125 | miRWalk |
| *COL1A1* | | hsa-miR-12126 | miRWalk |
| *COL1A1* | | hsa-miR-12128 | miRWalk |
| *COL1A1* | | hsa-miR-12129 | miRWalk |
| *COL1A1* | | hsa-miR-12131 | miRWalk |
| *COL1A1* | | hsa-miR-12133 | miRWalk |
| *COL1A1* | | hsa-miR-12136 | miRWalk |
