# Supplementary material for: Integrated bioinformatics analysis for the identification of idiopathic pulmonary fibrosis–related genes and potential therapeutic drugs
Source: BMC Pulm Med. 2023 Oct 4;23:373. doi: 10.1186/s12890-023-02678-z (PMC10552267; doi:10.1186/s12890-023-02678-z)
Supplement: Supplementary file 1 — Additional file 1: Table S1. The analyze network results of 1640 DEGs. Table S2. GO terms of the 18 hub genes. Table S3. KEGG pathways of the 18 hub genes. Table S4. Target microRNAs of SPP1 based on five online miRNA databases. Table S5. Target microRNAs of VEGFA based on five online miRNA databases. Table S6. Target microRNAs of COL1A1 based on five online miRNA databases. Table S7. Target microRNAs of CAV1 based on five online miRNA databases. Table S8. Target microRNAs of PECAM1 based on five online miRNA databases. Table S9. Target microRNAs of BMP4 based on five online miRNA databases. Table S10. Target microRNAs of FYN based on five online miRNA databases. Table S11. Traditional Chinese medicine prediction results of COL1A1. Table S12. Traditional Chinese medicine prediction results of VEGFA. Table S13. Traditional Chinese medicine prediction results of SPP1. [file 12890_2023_2678_MOESM1_ESM.zip › Supplementary Tables/Supplementary Table7.docx]

**Table S7 Target microRNAs of *CAV1* based on five online miRNA databases**

| Gene Symbol | microRNA | Database |
| --- | --- | --- |
| *CAV1* | hsa-miR-199b-5p | mirDIP |
| *CAV1* | hsa-miR-4282 | mirDIP |
| *CAV1* | hsa-miR-125a-3p | mirDIP |
| *CAV1* | hsa-miR-449c-5p | mirDIP |
| *CAV1* | hsa-miR-3140-3p | mirDIP |
| *CAV1* | hsa-miR-651-3p | mirDIP |
| *CAV1* | hsa-miR-30e-3p | mirDIP |
| *CAV1* | hsa-miR-30a-3p | mirDIP |
| *CAV1* | hsa-miR-30d-3p | mirDIP |
| *CAV1* | hsa-miR-940 | mirDIP |
| *CAV1* | hsa-miR-3609 | mirDIP |
| *CAV1* | hsa-miR-552-3p | mirDIP |
| *CAV1* | hsa-miR-3074-3p | mirDIP |
| *CAV1* | hsa-miR-513c-3p | mirDIP |
| *CAV1* | hsa-miR-5197-3p | mirDIP |
| *CAV1* | hsa-miR-1207-5p | mirDIP |
| *CAV1* | hsa-miR-1305 | mirDIP |
| *CAV1* | hsa-miR-4763-3p | mirDIP |
| *CAV1* | hsa-miR-612 | mirDIP |
| *CAV1* | hsa-miR-4672 | mirDIP |
| *CAV1* | hsa-miR-4506 | mirDIP |
| *CAV1* | hsa-miR-506-5p | mirDIP |
| *CAV1* | hsa-miR-4692 | mirDIP |
| *CAV1* | hsa-miR-5193 | mirDIP |
| *CAV1* | hsa-miR-548ah-5p | mirDIP |
| *CAV1* | hsa-miR-628-5p | mirDIP |
| *CAV1* | hsa-miR-6511a-5p | mirDIP |
| *CAV1* | hsa-miR-377-5p | mirDIP |
| *CAV1* | hsa-miR-2682-5p | mirDIP |
| *CAV1* | hsa-miR-4773 | mirDIP |
| *CAV1* | hsa-miR-6854-5p | mirDIP |
| *CAV1* | hsa-miR-1910-3p | mirDIP |
| *CAV1* | hsa-miR-593-5p | mirDIP |
| *CAV1* | hsa-miR-605-3p | mirDIP |
| *CAV1* | hsa-miR-1245a | mirDIP |
| *CAV1* | hsa-miR-4494 | mirDIP |
| *CAV1* | hsa-miR-4673 | mirDIP |
| *CAV1* | hsa-miR-526b-5p | mirDIP |
| *CAV1* | hsa-miR-4733-3p | mirDIP |
| *CAV1* | hsa-miR-548au-3p | mirDIP |
| *CAV1* | hsa-miR-7705 | mirDIP |
| *CAV1* | hsa-miR-4645-5p | mirDIP |
| *CAV1* | hsa-miR-6860 | mirDIP |
| *CAV1* | hsa-miR-3126-5p | mirDIP |
| *CAV1* | hsa-miR-4776-5p | mirDIP |
| *CAV1* | hsa-miR-4463 | mirDIP |
| *CAV1* | hsa-miR-5002-3p | mirDIP |
| *CAV1* | hsa-miR-7850-5p | mirDIP |
| *CAV1* | hsa-miR-1243 | mirDIP |
| *CAV1* | hsa-miR-1911-3p | mirDIP |
| *CAV1* | hsa-miR-4999-5p | mirDIP |
| *CAV1* | hsa-miR-892c-5p | mirDIP |
| *CAV1* | hsa-miR-6839-3p | mirDIP |
| *CAV1* | hsa-miR-5702 | mirDIP |
| *CAV1* | hsa-miR-8062 | mirDIP |
| *CAV1* | hsa-miR-12136 | mirDIP |
| *CAV1* | hsa-miR-524-3p | mirDIP |
| *CAV1* | hsa-miR-525-3p | mirDIP |
| *CAV1* | hsa-miR-8077 | mirDIP |
| *CAV1* | hsa-miR-4734 | mirDIP |
| *CAV1* | hsa-miR-758-5p | mirDIP |
| *CAV1* | hsa-miR-8069 | mirDIP |
| *CAV1* | hsa-miR-6872-3p | mirDIP |
| *CAV1* | hsa-miR-4783-3p | mirDIP |
| *CAV1* | hsa-miR-9718 | mirDIP |
| *CAV1* | hsa-miR-34a-5p | ENCORI |
| *CAV1* | hsa-miR-30e-5p | ENCORI |
| *CAV1* | hsa-miR-190b | ENCORI |
| *CAV1* | hsa-miR-199a-3p | ENCORI |
| *CAV1* | hsa-miR-205-5p | ENCORI |
| *CAV1* | hsa-miR-194-5p | ENCORI |
| *CAV1* | hsa-miR-202-3p | ENCORI |
| *CAV1* | hsa-miR-302e | ENCORI |
| *CAV1* | hsa-miR-196a-5p | ENCORI |
| *CAV1* | hsa-miR-17-5p | ENCORI |
| *CAV1* | hsa-miR-19a-3p | ENCORI |
| *CAV1* | hsa-miR-20a-5p | ENCORI |
| *CAV1* | hsa-miR-19b-3p | ENCORI |
| *CAV1* | hsa-miR-431-5p | ENCORI |
| *CAV1* | hsa-miR-432-5p | ENCORI |
| *CAV1* | hsa-miR-376a-3p | ENCORI |
| *CAV1* | hsa-miR-376b-3p | ENCORI |
| *CAV1* | hsa-miR-544a | ENCORI |
| *CAV1* | hsa-miR-323b-5p | ENCORI |
| *CAV1* | hsa-miR-154-5p | ENCORI |
| *CAV1* | hsa-miR-211-5p | ENCORI |
| *CAV1* | hsa-miR-190a-5p | ENCORI |
| *CAV1* | hsa-miR-7-5p | ENCORI |
| *CAV1* | hsa-miR-365a-3p | ENCORI |
| *CAV1* | hsa-miR-451a | ENCORI |
| *CAV1* | hsa-miR-144-3p | ENCORI |
| *CAV1* | hsa-miR-27a-3p | ENCORI |
| *CAV1* | hsa-miR-330-3p | ENCORI |
| *CAV1* | hsa-miR-125a-3p | ENCORI |
| *CAV1* | hsa-miR-498 | ENCORI |
| *CAV1* | hsa-miR-520e | ENCORI |
| *CAV1* | hsa-miR-520a-3p | ENCORI |
| *CAV1* | hsa-miR-520b | ENCORI |
| *CAV1* | hsa-miR-520c-3p | ENCORI |
| *CAV1* | hsa-miR-520d-3p | ENCORI |
| *CAV1* | hsa-miR-520g-3p | ENCORI |
| *CAV1* | hsa-miR-372-3p | ENCORI |
| *CAV1* | hsa-miR-373-3p | ENCORI |
| *CAV1* | hsa-miR-217 | ENCORI |
| *CAV1* | hsa-miR-216a-5p | ENCORI |
| *CAV1* | hsa-miR-128-3p | ENCORI |
| *CAV1* | hsa-miR-124-3p | ENCORI |
| *CAV1* | hsa-miR-302d-3p | ENCORI |
| *CAV1* | hsa-miR-302a-3p | ENCORI |
| *CAV1* | hsa-miR-302c-5p | ENCORI |
| *CAV1* | hsa-miR-302c-3p | ENCORI |
| *CAV1* | hsa-miR-302b-3p | ENCORI |
| *CAV1* | hsa-miR-449a | ENCORI |
| *CAV1* | hsa-miR-1271-5p | ENCORI |
| *CAV1* | hsa-miR-340-5p | ENCORI |
| *CAV1* | hsa-miR-196b-5p | ENCORI |
| *CAV1* | hsa-miR-93-5p | ENCORI |
| *CAV1* | hsa-miR-106b-5p | ENCORI |
| *CAV1* | hsa-miR-96-5p | ENCORI |
| *CAV1* | hsa-miR-31-5p | ENCORI |
| *CAV1* | hsa-miR-204-5p | ENCORI |
| *CAV1* | hsa-let-7d-5p | ENCORI |
| *CAV1* | hsa-miR-27b-3p | ENCORI |
| *CAV1* | hsa-miR-223-3p | ENCORI |
| *CAV1* | hsa-miR-421 | ENCORI |
| *CAV1* | hsa-miR-361-5p | ENCORI |
| *CAV1* | hsa-miR-448 | ENCORI |
| *CAV1* | hsa-miR-20b-5p | ENCORI |
| *CAV1* | hsa-miR-106a-5p | ENCORI |
| *CAV1* | hsa-miR-506-3p | ENCORI |
| *CAV1* | hsa-miR-124-3p.1 | TargetScan |
| *CAV1* | hsa-miR-670-3p | TargetScan |
| *CAV1* | hsa-miR-199a-5p | TargetScan |
| *CAV1* | hsa-miR-199b-5p | TargetScan |
| *CAV1* | hsa-miR-3163 | DIANA-micro T |
| *CAV1* | hsa-miR-4282 | DIANA-micro T |
| *CAV1* | hsa-miR-3148 | DIANA-micro T |
| *CAV1* | hsa-miR-548c-3p | DIANA-micro T |
| *CAV1* | hsa-miR-3606-3p | DIANA-micro T |
| *CAV1* | hsa-miR-6854-5p | DIANA-micro T |
| *CAV1* | hsa-miR-651-3p | DIANA-micro T |
| *CAV1* | hsa-miR-513a-3p | DIANA-micro T |
| *CAV1* | hsa-miR-5193 | DIANA-micro T |
| *CAV1* | hsa-miR-513c-3p | DIANA-micro T |
| *CAV1* | hsa-miR-4269 | DIANA-micro T |
| *CAV1* | hsa-miR-1911-3p | DIANA-micro T |
| *CAV1* | hsa-miR-6511a-5p | DIANA-micro T |
| *CAV1* | hsa-miR-548e-3p | DIANA-micro T |
| *CAV1* | hsa-miR-1245a | DIANA-micro T |
| *CAV1* | hsa-miR-6833-3p | DIANA-micro T |
| *CAV1* | hsa-miR-548y | DIANA-micro T |
| *CAV1* | hsa-miR-548bb-5p | DIANA-micro T |
| *CAV1* | hsa-miR-5197-3p | DIANA-micro T |
| *CAV1* | hsa-miR-451a | DIANA-micro T |
| *CAV1* | hsa-miR-6875-5p | DIANA-micro T |
| *CAV1* | hsa-miR-548d-5p | DIANA-micro T |
| *CAV1* | hsa-miR-1305 | DIANA-micro T |
| *CAV1* | hsa-miR-627-3p | DIANA-micro T |
| *CAV1* | hsa-miR-940 | DIANA-micro T |
| *CAV1* | hsa-miR-548c-5p | DIANA-micro T |
| *CAV1* | hsa-miR-548o-5p | DIANA-micro T |
| *CAV1* | hsa-miR-548am-5p | DIANA-micro T |
| *CAV1* | hsa-miR-548w | DIANA-micro T |
| *CAV1* | hsa-miR-548b-5p | DIANA-micro T |
| *CAV1* | hsa-miR-4692 | DIANA-micro T |
| *CAV1* | hsa-miR-548i | DIANA-micro T |
| *CAV1* | hsa-miR-548ab | DIANA-micro T |
| *CAV1* | hsa-miR-1910-3p | DIANA-micro T |
| *CAV1* | hsa-miR-4776-5p | DIANA-micro T |
| *CAV1* | hsa-miR-548as-5p | DIANA-micro T |
| *CAV1* | hsa-miR-548ay-5p | DIANA-micro T |
| *CAV1* | hsa-miR-6839-3p | DIANA-micro T |
| *CAV1* | hsa-miR-6124 | DIANA-micro T |
| *CAV1* | hsa-miR-548a-5p | DIANA-micro T |
| *CAV1* | hsa-miR-548n | DIANA-micro T |
| *CAV1* | hsa-miR-548ar-3p | DIANA-micro T |
| *CAV1* | hsa-miR-548au-5p | DIANA-micro T |
| *CAV1* | hsa-miR-548j-5p | DIANA-micro T |
| *CAV1* | hsa-miR-4760-3p | DIANA-micro T |
| *CAV1* | hsa-miR-34b-5p | DIANA-micro T |
| *CAV1* | hsa-miR-4506 | DIANA-micro T |
| *CAV1* | hsa-miR-449c-5p | DIANA-micro T |
| *CAV1* | hsa-miR-548h-5p | DIANA-micro T |
| *CAV1* | hsa-miR-6733-3p | DIANA-micro T |
| *CAV1* | hsa-miR-4999-5p | DIANA-micro T |
| *CAV1* | hsa-miR-2682-5p | DIANA-micro T |
| *CAV1* | hsa-miR-559 | DIANA-micro T |
| *CAV1* | hsa-miR-548ah-5p | DIANA-micro T |
| *CAV1* | hsa-miR-548ad-5p | DIANA-micro T |
| *CAV1* | hsa-miR-548ae-5p | DIANA-micro T |
| *CAV1* | hsa-miR-548ap-5p | DIANA-micro T |
| *CAV1* | hsa-miR-548ar-5p | DIANA-micro T |
| *CAV1* | hsa-miR-3126-5p | DIANA-micro T |
| *CAV1* | hsa-miR-548ak | DIANA-micro T |
| *CAV1* | hsa-miR-1277-5p | DIANA-micro T |
| *CAV1* | hsa-miR-4668-3p | DIANA-micro T |
| *CAV1* | hsa-miR-153-5p | DIANA-micro T |
| *CAV1* | hsa-miR-5000-5p | DIANA-micro T |
| *CAV1* | hsa-miR-6808-5p | DIANA-micro T |
| *CAV1* | hsa-miR-7705 | DIANA-micro T |
| *CAV1* | hsa-miR-5189-5p | DIANA-micro T |
| *CAV1* | hsa-miR-548l | DIANA-micro T |
| *CAV1* | hsa-miR-548x-3p | DIANA-micro T |
| *CAV1* | hsa-miR-548aj-3p | DIANA-micro T |
| *CAV1* | hsa-miR-577 | DIANA-micro T |
| *CAV1* | hsa-miR-5582-3p | DIANA-micro T |
| *CAV1* | hsa-miR-593-5p | DIANA-micro T |
| *CAV1* | hsa-miR-3714 | DIANA-micro T |
| *CAV1* | hsa-miR-4673 | DIANA-micro T |
| *CAV1* | hsa-miR-5004-3p | DIANA-micro T |
| *CAV1* | hsa-miR-3944-5p | DIANA-micro T |
| *CAV1* | hsa-miR-3074-3p | DIANA-micro T |
| *CAV1* | hsa-miR-4763-3p | DIANA-micro T |
| *CAV1* | hsa-miR-5590-3p | DIANA-micro T |
| *CAV1* | hsa-miR-8062 | DIANA-micro T |
| *CAV1* | hsa-miR-4672 | DIANA-micro T |
| *CAV1* | hsa-miR-6811-5p | DIANA-micro T |
| *CAV1* | hsa-miR-338-3p | DIANA-micro T |
| *CAV1* | hsa-miR-548az-3p | DIANA-micro T |
| *CAV1* | hsa-miR-890 | DIANA-micro T |
| *CAV1* | hsa-miR-6875-3p | DIANA-micro T |
| *CAV1* | hsa-miR-605-3p | DIANA-micro T |
| *CAV1* | hsa-miR-194-3p | DIANA-micro T |
| *CAV1* | hsa-miR-548k | DIANA-micro T |
| *CAV1* | hsa-miR-7850-5p | DIANA-micro T |
| *CAV1* | hsa-miR-6893-5p | DIANA-micro T |
| *CAV1* | hsa-miR-548a-3p | DIANA-micro T |
| *CAV1* | hsa-miR-5701 | DIANA-micro T |
| *CAV1* | hsa-miR-106a-5p | DIANA-micro T |
| *CAV1* | hsa-miR-3681-3p | DIANA-micro T |
| *CAV1* | hsa-miR-597-5p | DIANA-micro T |
| *CAV1* | hsa-miR-548av-5p | DIANA-micro T |
| *CAV1* | hsa-miR-520d-3p | DIANA-micro T |
| *CAV1* | hsa-miR-1285-3p | DIANA-micro T |
| *CAV1* | hsa-miR-22-5p | DIANA-micro T |
| *CAV1* | hsa-miR-1271-5p | DIANA-micro T |
| *CAV1* | hsa-miR-4494 | DIANA-micro T |
| *CAV1* | hsa-miR-3662 | DIANA-micro T |
| *CAV1* | hsa-miR-4251 | DIANA-micro T |
| *CAV1* | hsa-miR-520e | DIANA-micro T |
| *CAV1* | hsa-miR-6512-3p | DIANA-micro T |
| *CAV1* | hsa-miR-4517 | DIANA-micro T |
| *CAV1* | hsa-miR-6787-3p | DIANA-micro T |
| *CAV1* | hsa-miR-520c-3p | DIANA-micro T |
| *CAV1* | hsa-miR-4496 | DIANA-micro T |
| *CAV1* | hsa-miR-4520-2-3p | DIANA-micro T |
| *CAV1* | hsa-miR-3157-5p | DIANA-micro T |
| *CAV1* | hsa-miR-4476 | DIANA-micro T |
| *CAV1* | hsa-miR-520b | DIANA-micro T |
| *CAV1* | hsa-miR-6876-5p | DIANA-micro T |
| *CAV1* | hsa-miR-34c-5p | DIANA-micro T |
| *CAV1* | hsa-miR-4459 | DIANA-micro T |
| *CAV1* | hsa-miR-6165 | DIANA-micro T |
| *CAV1* | hsa-miR-6715b-5p | DIANA-micro T |
| *CAV1* | hsa-miR-7159-5p | DIANA-micro T |
| *CAV1* | hsa-miR-1207-5p | DIANA-micro T |
| *CAV1* | hsa-miR-573 | DIANA-micro T |
| *CAV1* | hsa-miR-449b-5p | DIANA-micro T |
| *CAV1* | hsa-miR-205-3p | DIANA-micro T |
| *CAV1* | hsa-miR-6505-5p | DIANA-micro T |
| *CAV1* | hsa-miR-8079 | DIANA-micro T |
| *CAV1* | hsa-miR-520a-3p | DIANA-micro T |
| *CAV1* | hsa-miR-664a-3p | DIANA-micro T |
| *CAV1* | hsa-miR-506-3p | DIANA-micro T |
| *CAV1* | hsa-miR-124-3p | DIANA-micro T |
| *CAV1* | hsa-miR-6792-5p | DIANA-micro T |
| *CAV1* | hsa-miR-4514 | DIANA-micro T |
| *CAV1* | hsa-miR-4742-5p | DIANA-micro T |
| *CAV1* | hsa-miR-548au-3p | DIANA-micro T |
| *CAV1* | hsa-miR-510-3p | DIANA-micro T |
| *CAV1* | hsa-miR-340-5p | DIANA-micro T |
| *CAV1* | hsa-miR-6797-3p | DIANA-micro T |
| *CAV1* | hsa-miR-1279 | DIANA-micro T |
| *CAV1* | hsa-miR-4773 | DIANA-micro T |
| *CAV1* | hsa-miR-548f-3p | DIANA-micro T |
| *CAV1* | hsa-miR-128-3p | DIANA-micro T |
| *CAV1* | hsa-miR-5007-3p | DIANA-micro T |
| *CAV1* | hsa-miR-6720-5p | DIANA-micro T |
| *CAV1* | hsa-miR-6829-3p | DIANA-micro T |
| *CAV1* | hsa-miR-6722-3p | DIANA-micro T |
| *CAV1* | hsa-miR-545-5p | DIANA-micro T |
| *CAV1* | hsa-miR-4803 | DIANA-micro T |
| *CAV1* | hsa-miR-520e-3p | miRWalk |
| *CAV1* | hsa-miR-520b-3p | miRWalk |
| *CAV1* | hsa-miR-7158-3p | miRWalk |
| *CAV1* | hsa-miR-8077 | miRWalk |
| *CAV1* | hsa-let-7b-5p | miRWalk |
| *CAV1* | hsa-let-7c-5p | miRWalk |
| *CAV1* | hsa-miR-27a-3p | miRWalk |
| *CAV1* | hsa-miR-320a-5p | miRWalk |
| *CAV1* | hsa-miR-619-5p | miRWalk |
| *CAV1* | hsa-miR-1237-3p | miRWalk |
| *CAV1* | hsa-miR-1908-5p | miRWalk |
| *CAV1* | hsa-miR-3120-5p | miRWalk |
| *CAV1* | hsa-miR-4437 | miRWalk |
| *CAV1* | hsa-miR-6090 | miRWalk |
| *CAV1* | hsa-miR-6751-3p | miRWalk |
| *CAV1* | hsa-miR-6780a-5p | miRWalk |
| *CAV1* | hsa-miR-6854-5p | miRWalk |
| *CAV1* | hsa-miR-6878-3p | miRWalk |
| *CAV1* | hsa-miR-6884-5p | miRWalk |
| *CAV1* | hsa-miR-6890-5p | miRWalk |
| *CAV1* | hsa-miR-1273h-5p | miRWalk |
| *CAV1* | hsa-miR-1236-5p | miRWalk |
| *CAV1* | hsa-miR-1914-3p | miRWalk |
| *CAV1* | hsa-miR-6736-5p | miRWalk |
| *CAV1* | hsa-miR-6813-3p | miRWalk |
| *CAV1* | hsa-miR-127-5p | miRWalk |
| *CAV1* | hsa-miR-8087 | miRWalk |
| *CAV1* | hsa-miR-4787-5p | miRWalk |
| *CAV1* | hsa-miR-24-2-5p | miRWalk |
| *CAV1* | hsa-miR-519d-5p | miRWalk |
| *CAV1* | hsa-miR-1229-5p | miRWalk |
| *CAV1* | hsa-miR-7106-5p | miRWalk |
| *CAV1* | hsa-miR-3085-5p | miRWalk |
| *CAV1* | hsa-miR-4747-5p | miRWalk |
| *CAV1* | hsa-miR-3679-5p | miRWalk |
| *CAV1* | hsa-miR-4449 | miRWalk |
| *CAV1* | hsa-miR-6797-5p | miRWalk |
| *CAV1* | hsa-let-7a-5p | miRWalk |
| *CAV1* | hsa-let-7e-5p | miRWalk |
| *CAV1* | hsa-miR-15a-3p | miRWalk |
| *CAV1* | hsa-miR-17-3p | miRWalk |
| *CAV1* | hsa-miR-19b-1-5p | miRWalk |
| *CAV1* | hsa-miR-23a-5p | miRWalk |
| *CAV1* | hsa-miR-30a-3p | miRWalk |
| *CAV1* | hsa-miR-31-5p | miRWalk |
| *CAV1* | hsa-miR-92a-1-5p | miRWalk |
| *CAV1* | hsa-miR-93-5p | miRWalk |
| *CAV1* | hsa-miR-99a-3p | miRWalk |
| *CAV1* | hsa-miR-29b-2-5p | miRWalk |
| *CAV1* | hsa-miR-196a-5p | miRWalk |
| *CAV1* | hsa-miR-197-3p | miRWalk |
| *CAV1* | hsa-miR-198 | miRWalk |
| *CAV1* | hsa-miR-139-3p | miRWalk |
| *CAV1* | hsa-miR-183-3p | miRWalk |
| *CAV1* | hsa-miR-204-3p | miRWalk |
| *CAV1* | hsa-miR-212-3p | miRWalk |
| *CAV1* | hsa-let-7g-3p | miRWalk |
| *CAV1* | hsa-miR-23b-5p | miRWalk |
| *CAV1* | hsa-miR-27b-5p | miRWalk |
| *CAV1* | hsa-miR-140-3p | miRWalk |
| *CAV1* | hsa-miR-143-3p | miRWalk |
| *CAV1* | hsa-miR-191-3p | miRWalk |
| *CAV1* | hsa-miR-125a-3p | miRWalk |
| *CAV1* | hsa-miR-146a-3p | miRWalk |
| *CAV1* | hsa-miR-149-3p | miRWalk |
| *CAV1* | hsa-miR-185-3p | miRWalk |
| *CAV1* | hsa-miR-320a-3p | miRWalk |
| *CAV1* | hsa-miR-128-2-5p | miRWalk |
| *CAV1* | hsa-miR-194-3p | miRWalk |
| *CAV1* | hsa-miR-106b-3p | miRWalk |
| *CAV1* | hsa-miR-130b-5p | miRWalk |
| *CAV1* | hsa-miR-361-3p | miRWalk |
| *CAV1* | hsa-miR-365b-5p | miRWalk |
| *CAV1* | hsa-miR-372-5p | miRWalk |
| *CAV1* | hsa-miR-377-5p | miRWalk |
| *CAV1* | hsa-miR-380-5p | miRWalk |
| *CAV1* | hsa-miR-330-3p | miRWalk |
| *CAV1* | hsa-miR-338-3p | miRWalk |
| *CAV1* | hsa-miR-346 | miRWalk |
| *CAV1* | hsa-miR-196b-5p | miRWalk |
| *CAV1* | hsa-miR-423-5p | miRWalk |
| *CAV1* | hsa-miR-424-5p | miRWalk |
| *CAV1* | hsa-miR-425-3p | miRWalk |
| *CAV1* | hsa-miR-18b-3p | miRWalk |
| *CAV1* | hsa-miR-433-3p | miRWalk |
| *CAV1* | hsa-miR-451a | miRWalk |
| *CAV1* | hsa-miR-409-3p | miRWalk |
| *CAV1* | hsa-miR-376b-5p | miRWalk |
| *CAV1* | hsa-miR-483-5p | miRWalk |
| *CAV1* | hsa-miR-483-3p | miRWalk |
| *CAV1* | hsa-miR-486-3p | miRWalk |
| *CAV1* | hsa-miR-488-5p | miRWalk |
| *CAV1* | hsa-miR-492 | miRWalk |
| *CAV1* | hsa-miR-432-3p | miRWalk |
| *CAV1* | hsa-miR-512-5p | miRWalk |
| *CAV1* | hsa-miR-498-5p | miRWalk |
| *CAV1* | hsa-miR-519e-3p | miRWalk |
| *CAV1* | hsa-miR-525-5p | miRWalk |
| *CAV1* | hsa-miR-516b-3p | miRWalk |
| *CAV1* | hsa-miR-518a-5p | miRWalk |
| *CAV1* | hsa-miR-518a-3p | miRWalk |
| *CAV1* | hsa-miR-527 | miRWalk |
| *CAV1* | hsa-miR-516a-3p | miRWalk |
| *CAV1* | hsa-miR-502-3p | miRWalk |
| *CAV1* | hsa-miR-503-3p | miRWalk |
| *CAV1* | hsa-miR-504-3p | miRWalk |
| *CAV1* | hsa-miR-505-5p | miRWalk |
| *CAV1* | hsa-miR-506-5p | miRWalk |
| *CAV1* | hsa-miR-508-5p | miRWalk |
| *CAV1* | hsa-miR-508-3p | miRWalk |
| *CAV1* | hsa-miR-510-5p | miRWalk |
| *CAV1* | hsa-miR-487b-5p | miRWalk |
| *CAV1* | hsa-miR-552-3p | miRWalk |
| *CAV1* | hsa-miR-558 | miRWalk |
| *CAV1* | hsa-miR-584-5p | miRWalk |
| *CAV1* | hsa-miR-588 | miRWalk |
| *CAV1* | hsa-miR-590-5p | miRWalk |
| *CAV1* | hsa-miR-592 | miRWalk |
| *CAV1* | hsa-miR-593-5p | miRWalk |
| *CAV1* | hsa-miR-596 | miRWalk |
| *CAV1* | hsa-miR-597-5p | miRWalk |
| *CAV1* | hsa-miR-601 | miRWalk |
| *CAV1* | hsa-miR-610 | miRWalk |
| *CAV1* | hsa-miR-612 | miRWalk |
| *CAV1* | hsa-miR-615-5p | miRWalk |
| *CAV1* | hsa-miR-622 | miRWalk |
| *CAV1* | hsa-miR-623 | miRWalk |
| *CAV1* | hsa-miR-630 | miRWalk |
| *CAV1* | hsa-miR-639 | miRWalk |
| *CAV1* | hsa-miR-643 | miRWalk |
| *CAV1* | hsa-miR-646 | miRWalk |
| *CAV1* | hsa-miR-647 | miRWalk |
| *CAV1* | hsa-miR-648 | miRWalk |
| *CAV1* | hsa-miR-650 | miRWalk |
| *CAV1* | hsa-miR-449b-3p | miRWalk |
| *CAV1* | hsa-miR-653-3p | miRWalk |
| *CAV1* | hsa-miR-659-3p | miRWalk |
| *CAV1* | hsa-miR-660-3p | miRWalk |
| *CAV1* | hsa-miR-668-5p | miRWalk |
| *CAV1* | hsa-miR-668-3p | miRWalk |
| *CAV1* | hsa-miR-320b | miRWalk |
| *CAV1* | hsa-miR-320c | miRWalk |
| *CAV1* | hsa-miR-1301-3p | miRWalk |
| *CAV1* | hsa-miR-766-5p | miRWalk |
| *CAV1* | hsa-miR-766-3p | miRWalk |
| *CAV1* | hsa-miR-1298-3p | miRWalk |
| *CAV1* | hsa-miR-765 | miRWalk |
| *CAV1* | hsa-miR-675-5p | miRWalk |
| *CAV1* | hsa-miR-298 | miRWalk |
| *CAV1* | hsa-miR-300 | miRWalk |
| *CAV1* | hsa-miR-892a | miRWalk |
| *CAV1* | hsa-miR-892b | miRWalk |
| *CAV1* | hsa-miR-875-3p | miRWalk |
| *CAV1* | hsa-miR-744-5p | miRWalk |
| *CAV1* | hsa-miR-885-5p | miRWalk |
| *CAV1* | hsa-miR-885-3p | miRWalk |
| *CAV1* | hsa-miR-877-3p | miRWalk |
| *CAV1* | hsa-miR-887-5p | miRWalk |
| *CAV1* | hsa-miR-920 | miRWalk |
| *CAV1* | hsa-miR-933 | miRWalk |
| *CAV1* | hsa-miR-937-5p | miRWalk |
| *CAV1* | hsa-miR-939-5p | miRWalk |
| *CAV1* | hsa-miR-943 | miRWalk |
| *CAV1* | hsa-miR-1178-5p | miRWalk |
| *CAV1* | hsa-miR-1182 | miRWalk |
| *CAV1* | hsa-miR-1225-3p | miRWalk |
| *CAV1* | hsa-miR-1227-5p | miRWalk |
| *CAV1* | hsa-miR-1228-5p | miRWalk |
| *CAV1* | hsa-miR-1233-5p | miRWalk |
| *CAV1* | hsa-miR-1236-3p | miRWalk |
| *CAV1* | hsa-miR-1237-5p | miRWalk |
| *CAV1* | hsa-miR-1202 | miRWalk |
| *CAV1* | hsa-miR-663b | miRWalk |
| *CAV1* | hsa-miR-1207-5p | miRWalk |
| *CAV1* | hsa-miR-1291 | miRWalk |
| *CAV1* | hsa-miR-1304-3p | miRWalk |
| *CAV1* | hsa-miR-1243 | miRWalk |
| *CAV1* | hsa-miR-1244 | miRWalk |
| *CAV1* | hsa-miR-1249-5p | miRWalk |
| *CAV1* | hsa-miR-1268a | miRWalk |
| *CAV1* | hsa-miR-1270 | miRWalk |
| *CAV1* | hsa-miR-1272 | miRWalk |
| *CAV1* | hsa-miR-1281 | miRWalk |
| *CAV1* | hsa-miR-1288-3p | miRWalk |
| *CAV1* | hsa-miR-1292-3p | miRWalk |
| *CAV1* | hsa-miR-664a-5p | miRWalk |
| *CAV1* | hsa-miR-1306-5p | miRWalk |
| *CAV1* | hsa-miR-1469 | miRWalk |
| *CAV1* | hsa-miR-1908-3p | miRWalk |
| *CAV1* | hsa-miR-1910-5p | miRWalk |
| *CAV1* | hsa-miR-1910-3p | miRWalk |
| *CAV1* | hsa-miR-2110 | miRWalk |
| *CAV1* | hsa-miR-2114-5p | miRWalk |
| *CAV1* | hsa-miR-548q | miRWalk |
| *CAV1* | hsa-miR-2276-3p | miRWalk |
| *CAV1* | hsa-miR-2277-5p | miRWalk |
| *CAV1* | hsa-miR-2682-5p | miRWalk |
| *CAV1* | hsa-miR-711 | miRWalk |
| *CAV1* | hsa-miR-2861 | miRWalk |
| *CAV1* | hsa-miR-3117-3p | miRWalk |
| *CAV1* | hsa-miR-3120-3p | miRWalk |
| *CAV1* | hsa-miR-3124-5p | miRWalk |
| *CAV1* | hsa-miR-3125 | miRWalk |
| *CAV1* | hsa-miR-3127-3p | miRWalk |
| *CAV1* | hsa-miR-3131 | miRWalk |
| *CAV1* | hsa-miR-3132 | miRWalk |
| *CAV1* | hsa-miR-378b | miRWalk |
| *CAV1* | hsa-miR-3140-3p | miRWalk |
| *CAV1* | hsa-miR-3141 | miRWalk |
| *CAV1* | hsa-miR-3152-5p | miRWalk |
| *CAV1* | hsa-miR-3153 | miRWalk |
| *CAV1* | hsa-miR-3154 | miRWalk |
| *CAV1* | hsa-miR-3155a | miRWalk |
| *CAV1* | hsa-miR-3158-5p | miRWalk |
| *CAV1* | hsa-miR-3159 | miRWalk |
| *CAV1* | hsa-miR-3161 | miRWalk |
| *CAV1* | hsa-miR-3162-5p | miRWalk |
| *CAV1* | hsa-miR-3163 | miRWalk |
| *CAV1* | hsa-miR-3173-5p | miRWalk |
| *CAV1* | hsa-miR-3173-3p | miRWalk |
| *CAV1* | hsa-miR-323b-5p | miRWalk |
| *CAV1* | hsa-miR-3180-5p | miRWalk |
| *CAV1* | hsa-miR-3184-5p | miRWalk |
| *CAV1* | hsa-miR-3185 | miRWalk |
| *CAV1* | hsa-miR-3065-3p | miRWalk |
| *CAV1* | hsa-miR-3187-5p | miRWalk |
| *CAV1* | hsa-miR-3188 | miRWalk |
| *CAV1* | hsa-miR-320e | miRWalk |
| *CAV1* | hsa-miR-3194-3p | miRWalk |
| *CAV1* | hsa-miR-3195 | miRWalk |
| *CAV1* | hsa-miR-3197 | miRWalk |
| *CAV1* | hsa-miR-3200-5p | miRWalk |
| *CAV1* | hsa-miR-514b-5p | miRWalk |
| *CAV1* | hsa-miR-3202 | miRWalk |
| *CAV1* | hsa-miR-4296 | miRWalk |
| *CAV1* | hsa-miR-4298 | miRWalk |
| *CAV1* | hsa-miR-4308 | miRWalk |
| *CAV1* | hsa-miR-4315 | miRWalk |
| *CAV1* | hsa-miR-4320 | miRWalk |
| *CAV1* | hsa-miR-4317 | miRWalk |
| *CAV1* | hsa-miR-4257 | miRWalk |
| *CAV1* | hsa-miR-4254 | miRWalk |
| *CAV1* | hsa-miR-4327 | miRWalk |
| *CAV1* | hsa-miR-4268 | miRWalk |
| *CAV1* | hsa-miR-4269 | miRWalk |
| *CAV1* | hsa-miR-4270 | miRWalk |
| *CAV1* | hsa-miR-4279 | miRWalk |
| *CAV1* | hsa-miR-4278 | miRWalk |
| *CAV1* | hsa-miR-4280 | miRWalk |
| *CAV1* | hsa-miR-4292 | miRWalk |
| *CAV1* | hsa-miR-4289 | miRWalk |
| *CAV1* | hsa-miR-3605-3p | miRWalk |
| *CAV1* | hsa-miR-3616-3p | miRWalk |
| *CAV1* | hsa-miR-3619-3p | miRWalk |
| *CAV1* | hsa-miR-3621 | miRWalk |
| *CAV1* | hsa-miR-3622a-5p | miRWalk |
| *CAV1* | hsa-miR-3622b-5p | miRWalk |
| *CAV1* | hsa-miR-3649 | miRWalk |
| *CAV1* | hsa-miR-3663-5p | miRWalk |
| *CAV1* | hsa-miR-3665 | miRWalk |
| *CAV1* | hsa-miR-3677-5p | miRWalk |
| *CAV1* | hsa-miR-3680-3p | miRWalk |
| *CAV1* | hsa-miR-3689a-5p | miRWalk |
| *CAV1* | hsa-miR-3690 | miRWalk |
| *CAV1* | hsa-miR-3692-5p | miRWalk |
| *CAV1* | hsa-miR-3689b-5p | miRWalk |
| *CAV1* | hsa-miR-3689b-3p | miRWalk |
| *CAV1* | hsa-miR-3911 | miRWalk |
| *CAV1* | hsa-miR-3917 | miRWalk |
| *CAV1* | hsa-miR-3926 | miRWalk |
| *CAV1* | hsa-miR-676-3p | miRWalk |
| *CAV1* | hsa-miR-3929 | miRWalk |
| *CAV1* | hsa-miR-3934-5p | miRWalk |
| *CAV1* | hsa-miR-3934-3p | miRWalk |
| *CAV1* | hsa-miR-3937 | miRWalk |
| *CAV1* | hsa-miR-3940-5p | miRWalk |
| *CAV1* | hsa-miR-3940-3p | miRWalk |
| *CAV1* | hsa-miR-3943 | miRWalk |
| *CAV1* | hsa-miR-3944-3p | miRWalk |
| *CAV1* | hsa-miR-3945 | miRWalk |
| *CAV1* | hsa-miR-550b-2-5p | miRWalk |
| *CAV1* | hsa-miR-4420 | miRWalk |
| *CAV1* | hsa-miR-378g | miRWalk |
| *CAV1* | hsa-miR-4430 | miRWalk |
| *CAV1* | hsa-miR-4433a-5p | miRWalk |
| *CAV1* | hsa-miR-4435 | miRWalk |
| *CAV1* | hsa-miR-4438 | miRWalk |
| *CAV1* | hsa-miR-4439 | miRWalk |
| *CAV1* | hsa-miR-4440 | miRWalk |
| *CAV1* | hsa-miR-4444 | miRWalk |
| *CAV1* | hsa-miR-4448 | miRWalk |
| *CAV1* | hsa-miR-4453 | miRWalk |
| *CAV1* | hsa-miR-4463 | miRWalk |
| *CAV1* | hsa-miR-4467 | miRWalk |
| *CAV1* | hsa-miR-4469 | miRWalk |
| *CAV1* | hsa-miR-4472 | miRWalk |
| *CAV1* | hsa-miR-4475 | miRWalk |
| *CAV1* | hsa-miR-4478 | miRWalk |
| *CAV1* | hsa-miR-3689c | miRWalk |
| *CAV1* | hsa-miR-3689d | miRWalk |
| *CAV1* | hsa-miR-3689e | miRWalk |
| *CAV1* | hsa-miR-4484 | miRWalk |
| *CAV1* | hsa-miR-4486 | miRWalk |
| *CAV1* | hsa-miR-4487 | miRWalk |
| *CAV1* | hsa-miR-4494 | miRWalk |
| *CAV1* | hsa-miR-4499 | miRWalk |
| *CAV1* | hsa-miR-4502 | miRWalk |
| *CAV1* | hsa-miR-4506 | miRWalk |
| *CAV1* | hsa-miR-4508 | miRWalk |
| *CAV1* | hsa-miR-4512 | miRWalk |
| *CAV1* | hsa-miR-4516 | miRWalk |
| *CAV1* | hsa-miR-4523 | miRWalk |
| *CAV1* | hsa-miR-4526 | miRWalk |
| *CAV1* | hsa-miR-4529-5p | miRWalk |
| *CAV1* | hsa-miR-4529-3p | miRWalk |
| *CAV1* | hsa-miR-1587 | miRWalk |
| *CAV1* | hsa-miR-3960 | miRWalk |
| *CAV1* | hsa-miR-3972 | miRWalk |
| *CAV1* | hsa-miR-3976 | miRWalk |
| *CAV1* | hsa-miR-4638-5p | miRWalk |
| *CAV1* | hsa-miR-4646-5p | miRWalk |
| *CAV1* | hsa-miR-4646-3p | miRWalk |
| *CAV1* | hsa-miR-4649-5p | miRWalk |
| *CAV1* | hsa-miR-4649-3p | miRWalk |
| *CAV1* | hsa-miR-4650-5p | miRWalk |
| *CAV1* | hsa-miR-4650-3p | miRWalk |
| *CAV1* | hsa-miR-4660 | miRWalk |
| *CAV1* | hsa-miR-4662a-5p | miRWalk |
| *CAV1* | hsa-miR-4663 | miRWalk |
| *CAV1* | hsa-miR-4664-5p | miRWalk |
| *CAV1* | hsa-miR-4669 | miRWalk |
| *CAV1* | hsa-miR-4685-5p | miRWalk |
| *CAV1* | hsa-miR-1343-3p | miRWalk |
| *CAV1* | hsa-miR-4688 | miRWalk |
| *CAV1* | hsa-miR-4689 | miRWalk |
| *CAV1* | hsa-miR-4690-5p | miRWalk |
| *CAV1* | hsa-miR-4692 | miRWalk |
| *CAV1* | hsa-miR-4695-5p | miRWalk |
| *CAV1* | hsa-miR-4697-5p | miRWalk |
| *CAV1* | hsa-miR-4700-3p | miRWalk |
| *CAV1* | hsa-miR-4701-3p | miRWalk |
| *CAV1* | hsa-miR-4709-3p | miRWalk |
| *CAV1* | hsa-miR-4711-5p | miRWalk |
| *CAV1* | hsa-miR-4713-3p | miRWalk |
| *CAV1* | hsa-miR-3529-5p | miRWalk |
| *CAV1* | hsa-miR-4722-5p | miRWalk |
| *CAV1* | hsa-miR-4722-3p | miRWalk |
| *CAV1* | hsa-miR-4723-5p | miRWalk |
| *CAV1* | hsa-miR-4725-5p | miRWalk |
| *CAV1* | hsa-miR-4725-3p | miRWalk |
| *CAV1* | hsa-miR-4727-3p | miRWalk |
| *CAV1* | hsa-miR-4728-3p | miRWalk |
| *CAV1* | hsa-miR-4731-5p | miRWalk |
| *CAV1* | hsa-miR-4731-3p | miRWalk |
| *CAV1* | hsa-miR-4741 | miRWalk |
| *CAV1* | hsa-miR-4742-5p | miRWalk |
| *CAV1* | hsa-miR-4742-3p | miRWalk |
| *CAV1* | hsa-miR-4748 | miRWalk |
| *CAV1* | hsa-miR-4749-5p | miRWalk |
| *CAV1* | hsa-miR-4750-5p | miRWalk |
| *CAV1* | hsa-miR-4755-3p | miRWalk |
| *CAV1* | hsa-miR-4769-5p | miRWalk |
| *CAV1* | hsa-miR-4769-3p | miRWalk |
| *CAV1* | hsa-miR-4776-5p | miRWalk |
| *CAV1* | hsa-miR-4779 | miRWalk |
| *CAV1* | hsa-miR-4436b-5p | miRWalk |
| *CAV1* | hsa-miR-4781-5p | miRWalk |
| *CAV1* | hsa-miR-4784 | miRWalk |
| *CAV1* | hsa-miR-4785 | miRWalk |
| *CAV1* | hsa-miR-2467-5p | miRWalk |
| *CAV1* | hsa-miR-2467-3p | miRWalk |
| *CAV1* | hsa-miR-4787-3p | miRWalk |
| *CAV1* | hsa-miR-4802-5p | miRWalk |
| *CAV1* | hsa-miR-4804-3p | miRWalk |
| *CAV1* | hsa-miR-5000-3p | miRWalk |
| *CAV1* | hsa-miR-5010-3p | miRWalk |
| *CAV1* | hsa-miR-5087 | miRWalk |
| *CAV1* | hsa-miR-5088-3p | miRWalk |
| *CAV1* | hsa-miR-5091 | miRWalk |
| *CAV1* | hsa-miR-5092 | miRWalk |
| *CAV1* | hsa-miR-5186 | miRWalk |
| *CAV1* | hsa-miR-5187-5p | miRWalk |
| *CAV1* | hsa-miR-5187-3p | miRWalk |
| *CAV1* | hsa-miR-5193 | miRWalk |
| *CAV1* | hsa-miR-664b-5p | miRWalk |
| *CAV1* | hsa-miR-5584-5p | miRWalk |
| *CAV1* | hsa-miR-1295b-3p | miRWalk |
| *CAV1* | hsa-miR-5591-3p | miRWalk |
| *CAV1* | hsa-miR-5681b | miRWalk |
| *CAV1* | hsa-miR-5693 | miRWalk |
| *CAV1* | hsa-miR-5703 | miRWalk |
| *CAV1* | hsa-miR-5706 | miRWalk |
| *CAV1* | hsa-miR-1199-5p | miRWalk |
| *CAV1* | hsa-miR-6069 | miRWalk |
| *CAV1* | hsa-miR-6070 | miRWalk |
| *CAV1* | hsa-miR-6076 | miRWalk |
| *CAV1* | hsa-miR-6082 | miRWalk |
| *CAV1* | hsa-miR-6085 | miRWalk |
| *CAV1* | hsa-miR-6089 | miRWalk |
| *CAV1* | hsa-miR-6124 | miRWalk |
| *CAV1* | hsa-miR-6125 | miRWalk |
| *CAV1* | hsa-miR-6127 | miRWalk |
| *CAV1* | hsa-miR-6132 | miRWalk |
| *CAV1* | hsa-miR-6133 | miRWalk |
| *CAV1* | hsa-miR-6165 | miRWalk |
| *CAV1* | hsa-miR-6499-3p | miRWalk |
| *CAV1* | hsa-miR-6500-5p | miRWalk |
| *CAV1* | hsa-miR-6501-5p | miRWalk |
| *CAV1* | hsa-miR-6503-3p | miRWalk |
| *CAV1* | hsa-miR-6507-5p | miRWalk |
| *CAV1* | hsa-miR-6511a-5p | miRWalk |
| *CAV1* | hsa-miR-6513-5p | miRWalk |
| *CAV1* | hsa-miR-6515-5p | miRWalk |
| *CAV1* | hsa-miR-6715b-5p | miRWalk |
| *CAV1* | hsa-miR-6511b-3p | miRWalk |
| *CAV1* | hsa-miR-6720-5p | miRWalk |
| *CAV1* | hsa-miR-6721-5p | miRWalk |
| *CAV1* | hsa-miR-6722-3p | miRWalk |
| *CAV1* | hsa-miR-6726-5p | miRWalk |
| *CAV1* | hsa-miR-6727-5p | miRWalk |
| *CAV1* | hsa-miR-6728-3p | miRWalk |
| *CAV1* | hsa-miR-6730-5p | miRWalk |
| *CAV1* | hsa-miR-6731-5p | miRWalk |
| *CAV1* | hsa-miR-6731-3p | miRWalk |
| *CAV1* | hsa-miR-6732-5p | miRWalk |
| *CAV1* | hsa-miR-6732-3p | miRWalk |
| *CAV1* | hsa-miR-6734-5p | miRWalk |
| *CAV1* | hsa-miR-6734-3p | miRWalk |
| *CAV1* | hsa-miR-6735-5p | miRWalk |
| *CAV1* | hsa-miR-6735-3p | miRWalk |
| *CAV1* | hsa-miR-6736-3p | miRWalk |
| *CAV1* | hsa-miR-6737-5p | miRWalk |
| *CAV1* | hsa-miR-6738-3p | miRWalk |
| *CAV1* | hsa-miR-6739-3p | miRWalk |
| *CAV1* | hsa-miR-6742-5p | miRWalk |
| *CAV1* | hsa-miR-6744-5p | miRWalk |
| *CAV1* | hsa-miR-6745 | miRWalk |
| *CAV1* | hsa-miR-6747-5p | miRWalk |
| *CAV1* | hsa-miR-6748-5p | miRWalk |
| *CAV1* | hsa-miR-6749-5p | miRWalk |
| *CAV1* | hsa-miR-6750-5p | miRWalk |
| *CAV1* | hsa-miR-6752-3p | miRWalk |
| *CAV1* | hsa-miR-6753-5p | miRWalk |
| *CAV1* | hsa-miR-6754-5p | miRWalk |
| *CAV1* | hsa-miR-6756-5p | miRWalk |
| *CAV1* | hsa-miR-6757-5p | miRWalk |
| *CAV1* | hsa-miR-6757-3p | miRWalk |
| *CAV1* | hsa-miR-6758-3p | miRWalk |
| *CAV1* | hsa-miR-6759-5p | miRWalk |
| *CAV1* | hsa-miR-6759-3p | miRWalk |
| *CAV1* | hsa-miR-6760-5p | miRWalk |
| *CAV1* | hsa-miR-6760-3p | miRWalk |
| *CAV1* | hsa-miR-6763-5p | miRWalk |
| *CAV1* | hsa-miR-6764-3p | miRWalk |
| *CAV1* | hsa-miR-6768-3p | miRWalk |
| *CAV1* | hsa-miR-6769a-3p | miRWalk |
| *CAV1* | hsa-miR-6771-5p | miRWalk |
| *CAV1* | hsa-miR-6772-5p | miRWalk |
| *CAV1* | hsa-miR-6772-3p | miRWalk |
| *CAV1* | hsa-miR-6773-3p | miRWalk |
| *CAV1* | hsa-miR-6774-5p | miRWalk |
| *CAV1* | hsa-miR-6775-5p | miRWalk |
| *CAV1* | hsa-miR-6777-5p | miRWalk |
| *CAV1* | hsa-miR-6778-5p | miRWalk |
| *CAV1* | hsa-miR-6779-5p | miRWalk |
| *CAV1* | hsa-miR-6779-3p | miRWalk |
| *CAV1* | hsa-miR-6780a-3p | miRWalk |
| *CAV1* | hsa-miR-6782-5p | miRWalk |
| *CAV1* | hsa-miR-6783-3p | miRWalk |
| *CAV1* | hsa-miR-6784-3p | miRWalk |
| *CAV1* | hsa-miR-6786-5p | miRWalk |
| *CAV1* | hsa-miR-6787-5p | miRWalk |
| *CAV1* | hsa-miR-6788-5p | miRWalk |
| *CAV1* | hsa-miR-6791-3p | miRWalk |
| *CAV1* | hsa-miR-6792-5p | miRWalk |
| *CAV1* | hsa-miR-6793-5p | miRWalk |
| *CAV1* | hsa-miR-6794-5p | miRWalk |
| *CAV1* | hsa-miR-6795-5p | miRWalk |
| *CAV1* | hsa-miR-6795-3p | miRWalk |
| *CAV1* | hsa-miR-6798-5p | miRWalk |
| *CAV1* | hsa-miR-6799-5p | miRWalk |
| *CAV1* | hsa-miR-6800-5p | miRWalk |
| *CAV1* | hsa-miR-6800-3p | miRWalk |
| *CAV1* | hsa-miR-6801-5p | miRWalk |
| *CAV1* | hsa-miR-6803-3p | miRWalk |
| *CAV1* | hsa-miR-6804-3p | miRWalk |
| *CAV1* | hsa-miR-6805-5p | miRWalk |
| *CAV1* | hsa-miR-6807-5p | miRWalk |
| *CAV1* | hsa-miR-6809-5p | miRWalk |
| *CAV1* | hsa-miR-6809-3p | miRWalk |
| *CAV1* | hsa-miR-6810-3p | miRWalk |
| *CAV1* | hsa-miR-6812-3p | miRWalk |
| *CAV1* | hsa-miR-6813-5p | miRWalk |
| *CAV1* | hsa-miR-6814-5p | miRWalk |
| *CAV1* | hsa-miR-6815-5p | miRWalk |
| *CAV1* | hsa-miR-6816-5p | miRWalk |
| *CAV1* | hsa-miR-6818-3p | miRWalk |
| *CAV1* | hsa-miR-6821-5p | miRWalk |
| *CAV1* | hsa-miR-6821-3p | miRWalk |
| *CAV1* | hsa-miR-6822-5p | miRWalk |
| *CAV1* | hsa-miR-6823-5p | miRWalk |
| *CAV1* | hsa-miR-6824-5p | miRWalk |
| *CAV1* | hsa-miR-6826-3p | miRWalk |
| *CAV1* | hsa-miR-6827-3p | miRWalk |
| *CAV1* | hsa-miR-6828-3p | miRWalk |
| *CAV1* | hsa-miR-6829-5p | miRWalk |
| *CAV1* | hsa-miR-6830-3p | miRWalk |
| *CAV1* | hsa-miR-6833-5p | miRWalk |
| *CAV1* | hsa-miR-6833-3p | miRWalk |
| *CAV1* | hsa-miR-6834-5p | miRWalk |
| *CAV1* | hsa-miR-6780b-5p | miRWalk |
| *CAV1* | hsa-miR-6780b-3p | miRWalk |
| *CAV1* | hsa-miR-6837-3p | miRWalk |
| *CAV1* | hsa-miR-6842-3p | miRWalk |
| *CAV1* | hsa-miR-6845-5p | miRWalk |
| *CAV1* | hsa-miR-6847-5p | miRWalk |
| *CAV1* | hsa-miR-6848-3p | miRWalk |
| *CAV1* | hsa-miR-6850-5p | miRWalk |
| *CAV1* | hsa-miR-6852-5p | miRWalk |
| *CAV1* | hsa-miR-6853-3p | miRWalk |
| *CAV1* | hsa-miR-6856-5p | miRWalk |
| *CAV1* | hsa-miR-6856-3p | miRWalk |
| *CAV1* | hsa-miR-6857-5p | miRWalk |
| *CAV1* | hsa-miR-6857-3p | miRWalk |
| *CAV1* | hsa-miR-6858-5p | miRWalk |
| *CAV1* | hsa-miR-6859-3p | miRWalk |
| *CAV1* | hsa-miR-6769b-5p | miRWalk |
| *CAV1* | hsa-miR-6860 | miRWalk |
| *CAV1* | hsa-miR-6861-5p | miRWalk |
| *CAV1* | hsa-miR-6866-5p | miRWalk |
| *CAV1* | hsa-miR-6871-3p | miRWalk |
| *CAV1* | hsa-miR-6875-5p | miRWalk |
| *CAV1* | hsa-miR-6878-5p | miRWalk |
| *CAV1* | hsa-miR-6879-5p | miRWalk |
| *CAV1* | hsa-miR-6882-3p | miRWalk |
| *CAV1* | hsa-miR-6885-5p | miRWalk |
| *CAV1* | hsa-miR-6885-3p | miRWalk |
| *CAV1* | hsa-miR-6887-3p | miRWalk |
| *CAV1* | hsa-miR-6888-5p | miRWalk |
| *CAV1* | hsa-miR-6889-5p | miRWalk |
| *CAV1* | hsa-miR-6891-5p | miRWalk |
| *CAV1* | hsa-miR-6891-3p | miRWalk |
| *CAV1* | hsa-miR-6892-3p | miRWalk |
| *CAV1* | hsa-miR-6893-5p | miRWalk |
| *CAV1* | hsa-miR-6893-3p | miRWalk |
| *CAV1* | hsa-miR-6894-5p | miRWalk |
| *CAV1* | hsa-miR-6894-3p | miRWalk |
| *CAV1* | hsa-miR-7107-5p | miRWalk |
| *CAV1* | hsa-miR-7108-5p | miRWalk |
| *CAV1* | hsa-miR-7108-3p | miRWalk |
| *CAV1* | hsa-miR-7109-5p | miRWalk |
| *CAV1* | hsa-miR-7109-3p | miRWalk |
| *CAV1* | hsa-miR-7110-5p | miRWalk |
| *CAV1* | hsa-miR-7110-3p | miRWalk |
| *CAV1* | hsa-miR-7111-5p | miRWalk |
| *CAV1* | hsa-miR-7150 | miRWalk |
| *CAV1* | hsa-miR-7155-5p | miRWalk |
| *CAV1* | hsa-miR-7160-3p | miRWalk |
| *CAV1* | hsa-miR-7162-5p | miRWalk |
| *CAV1* | hsa-miR-7702 | miRWalk |
| *CAV1* | hsa-miR-7703 | miRWalk |
| *CAV1* | hsa-miR-4433b-5p | miRWalk |
| *CAV1* | hsa-miR-7847-3p | miRWalk |
| *CAV1* | hsa-miR-7851-3p | miRWalk |
| *CAV1* | hsa-miR-8053 | miRWalk |
| *CAV1* | hsa-miR-8064 | miRWalk |
| *CAV1* | hsa-miR-8065 | miRWalk |
| *CAV1* | hsa-miR-8071 | miRWalk |
| *CAV1* | hsa-miR-8072 | miRWalk |
| *CAV1* | hsa-miR-8074 | miRWalk |
| *CAV1* | hsa-miR-8082 | miRWalk |
| *CAV1* | hsa-miR-8088 | miRWalk |
| *CAV1* | hsa-miR-9718 | miRWalk |
| *CAV1* | hsa-miR-10396a-5p | miRWalk |
| *CAV1* | hsa-miR-10398-5p | miRWalk |
| *CAV1* | hsa-miR-10398-3p | miRWalk |
| *CAV1* | hsa-miR-10400-5p | miRWalk |
| *CAV1* | hsa-miR-10401-5p | miRWalk |
| *CAV1* | hsa-miR-10396b-5p | miRWalk |
| *CAV1* | hsa-miR-10524-5p | miRWalk |
| *CAV1* | hsa-miR-10526-3p | miRWalk |
| *CAV1* | hsa-miR-11181-3p | miRWalk |
| *CAV1* | hsa-miR-11399 | miRWalk |
| *CAV1* | hsa-miR-6529-5p | miRWalk |
| *CAV1* | hsa-miR-12114 | miRWalk |
| *CAV1* | hsa-miR-12117 | miRWalk |
| *CAV1* | hsa-miR-12128 | miRWalk |
| *CAV1* | hsa-miR-12133 | miRWalk |
| *CAV1* | hsa-miR-19b-2-5p | miRWalk |
| *CAV1* | hsa-miR-92a-2-5p | miRWalk |
| *CAV1* | hsa-miR-208a-5p | miRWalk |
| *CAV1* | hsa-miR-186-3p | miRWalk |
| *CAV1* | hsa-miR-671-5p | miRWalk |
| *CAV1* | hsa-miR-3192-5p | miRWalk |
| *CAV1* | hsa-miR-3196 | miRWalk |
| *CAV1* | hsa-miR-4314 | miRWalk |
| *CAV1* | hsa-miR-4281 | miRWalk |
| *CAV1* | hsa-miR-3922-5p | miRWalk |
| *CAV1* | hsa-miR-4447 | miRWalk |
| *CAV1* | hsa-miR-4489 | miRWalk |
| *CAV1* | hsa-miR-4647 | miRWalk |
| *CAV1* | hsa-miR-4687-5p | miRWalk |
| *CAV1* | hsa-miR-4728-5p | miRWalk |
| *CAV1* | hsa-miR-4763-3p | miRWalk |
| *CAV1* | hsa-miR-4776-3p | miRWalk |
| *CAV1* | hsa-miR-6086 | miRWalk |
| *CAV1* | hsa-miR-6746-5p | miRWalk |
| *CAV1* | hsa-miR-6814-3p | miRWalk |
| *CAV1* | hsa-miR-6855-3p | miRWalk |
| *CAV1* | hsa-miR-6890-3p | miRWalk |
| *CAV1* | hsa-miR-8085 | miRWalk |
| *CAV1* | hsa-miR-12116 | miRWalk |
| *CAV1* | hsa-miR-92a-3p | miRWalk |
| *CAV1* | hsa-miR-324-3p | miRWalk |
| *CAV1* | hsa-miR-642a-5p | miRWalk |
| *CAV1* | hsa-miR-1913 | miRWalk |
| *CAV1* | hsa-miR-4321 | miRWalk |
| *CAV1* | hsa-miR-4498 | miRWalk |
| *CAV1* | hsa-miR-4667-3p | miRWalk |
| *CAV1* | hsa-miR-4708-5p | miRWalk |
| *CAV1* | hsa-miR-5196-5p | miRWalk |
| *CAV1* | hsa-miR-5584-3p | miRWalk |
| *CAV1* | hsa-miR-6749-3p | miRWalk |
| *CAV1* | hsa-miR-6756-3p | miRWalk |
| *CAV1* | hsa-miR-6764-5p | miRWalk |
| *CAV1* | hsa-miR-6782-3p | miRWalk |
| *CAV1* | hsa-miR-6788-3p | miRWalk |
| *CAV1* | hsa-miR-6805-3p | miRWalk |
| *CAV1* | hsa-miR-6806-5p | miRWalk |
| *CAV1* | hsa-miR-6815-3p | miRWalk |
| *CAV1* | hsa-miR-6819-3p | miRWalk |
| *CAV1* | hsa-miR-6823-3p | miRWalk |
| *CAV1* | hsa-miR-6840-5p | miRWalk |
| *CAV1* | hsa-miR-6846-5p | miRWalk |
| *CAV1* | hsa-miR-6769b-3p | miRWalk |
| *CAV1* | hsa-miR-6864-3p | miRWalk |
| *CAV1* | hsa-miR-6889-3p | miRWalk |
| *CAV1* | hsa-miR-1226-5p | miRWalk |
| *CAV1* | hsa-miR-1538 | miRWalk |
| *CAV1* | hsa-miR-1268b | miRWalk |
| *CAV1* | hsa-miR-4429 | miRWalk |
