# Supplementary material for: Integrated bioinformatics analysis for the identification of idiopathic pulmonary fibrosis–related genes and potential therapeutic drugs
Source: BMC Pulm Med. 2023 Oct 4;23:373. doi: 10.1186/s12890-023-02678-z (PMC10552267; doi:10.1186/s12890-023-02678-z)
Supplement: Supplementary file 1 — Additional file 1: Table S1. The analyze network results of 1640 DEGs. Table S2. GO terms of the 18 hub genes. Table S3. KEGG pathways of the 18 hub genes. Table S4. Target microRNAs of SPP1 based on five online miRNA databases. Table S5. Target microRNAs of VEGFA based on five online miRNA databases. Table S6. Target microRNAs of COL1A1 based on five online miRNA databases. Table S7. Target microRNAs of CAV1 based on five online miRNA databases. Table S8. Target microRNAs of PECAM1 based on five online miRNA databases. Table S9. Target microRNAs of BMP4 based on five online miRNA databases. Table S10. Target microRNAs of FYN based on five online miRNA databases. Table S11. Traditional Chinese medicine prediction results of COL1A1. Table S12. Traditional Chinese medicine prediction results of VEGFA. Table S13. Traditional Chinese medicine prediction results of SPP1. [file 12890_2023_2678_MOESM1_ESM.zip › Supplementary Tables/Supplementary Table8.docx]

**Table S8 Target microRNAs of *PECAM1* based on five online miRNA databases**

| Gene Symbol | microRNA | Database |
| --- | --- | --- |
| *PECAM1* | hsa-miR-6124 | mirDIP |
| *PECAM1* | hsa-miR-874-3p | mirDIP |
| *PECAM1* | hsa-miR-5699-3p | mirDIP |
| *PECAM1* | hsa-miR-4662a-5p | mirDIP |
| *PECAM1* | hsa-miR-1233-5p | mirDIP |
| *PECAM1* | hsa-miR-6880-5p | mirDIP |
| *PECAM1* | hsa-miR-6165 | mirDIP |
| *PECAM1* | hsa-miR-6847-3p | mirDIP |
| *PECAM1* | hsa-miR-1271-3p | mirDIP |
| *PECAM1* | hsa-miR-6855-5p | mirDIP |
| *PECAM1* | hsa-miR-383-3p | mirDIP |
| *PECAM1* | hsa-miR-597-3p | mirDIP |
| *PECAM1* | hsa-miR-6819-3p | mirDIP |
| *PECAM1* | hsa-miR-6858-3p | mirDIP |
| *PECAM1* | hsa-miR-6877-3p | mirDIP |
| *PECAM1* | hsa-miR-6804-5p | mirDIP |
| *PECAM1* | hsa-miR-6816-3p | mirDIP |
| *PECAM1* | hsa-miR-134-3p | mirDIP |
| *PECAM1* | hsa-miR-933 | mirDIP |
| *PECAM1* | hsa-miR-9851-3p | mirDIP |
| *PECAM1* | hsa-miR-6084 | mirDIP |
| *PECAM1* | hsa-miR-10399-3p | mirDIP |
| *PECAM1* | hsa-miR-718 | mirDIP |
| *PECAM1* | None | ENCORI |
| *PECAM1* | None | TargetScan |
| *PECAM1* | None | DIANA-micro T |
| *PECAM1* | hsa-miR-4420 | miRWalk |
| *PECAM1* | hsa-miR-4447 | miRWalk |
| *PECAM1* | hsa-miR-129-1-3p | miRWalk |
| *PECAM1* | hsa-miR-132-3p | miRWalk |
| *PECAM1* | hsa-miR-129-2-3p | miRWalk |
| *PECAM1* | hsa-miR-29c-5p | miRWalk |
| *PECAM1* | hsa-miR-422a | miRWalk |
| *PECAM1* | hsa-miR-574-5p | miRWalk |
| *PECAM1* | hsa-miR-646 | miRWalk |
| *PECAM1* | hsa-miR-1228-5p | miRWalk |
| *PECAM1* | hsa-miR-2682-3p | miRWalk |
| *PECAM1* | hsa-miR-3161 | miRWalk |
| *PECAM1* | hsa-miR-4274 | miRWalk |
| *PECAM1* | hsa-miR-4430 | miRWalk |
| *PECAM1* | hsa-miR-4716-3p | miRWalk |
| *PECAM1* | hsa-miR-4733-5p | miRWalk |
| *PECAM1* | hsa-miR-4743-5p | miRWalk |
| *PECAM1* | hsa-miR-6880-5p | miRWalk |
| *PECAM1* | hsa-miR-6881-5p | miRWalk |
| *PECAM1* | hsa-miR-8087 | miRWalk |
| *PECAM1* | hsa-miR-548d-5p | miRWalk |
| *PECAM1* | hsa-miR-889-5p | miRWalk |
| *PECAM1* | hsa-miR-302a-3p | miRWalk |
| *PECAM1* | hsa-miR-1285-3p | miRWalk |
| *PECAM1* | hsa-miR-3141 | miRWalk |
| *PECAM1* | hsa-miR-6070 | miRWalk |
| *PECAM1* | hsa-miR-6763-5p | miRWalk |
| *PECAM1* | hsa-miR-7112-3p | miRWalk |
| *PECAM1* | hsa-miR-7113-5p | miRWalk |
| *PECAM1* | hsa-miR-10397-5p | miRWalk |
| *PECAM1* | hsa-miR-602 | miRWalk |
| *PECAM1* | hsa-miR-7107-5p | miRWalk |
| *PECAM1* | hsa-miR-6826-3p | miRWalk |
| *PECAM1* | hsa-miR-1249-3p | miRWalk |
| *PECAM1* | hsa-miR-1972 | miRWalk |
| *PECAM1* | hsa-miR-1260b | miRWalk |
| *PECAM1* | hsa-miR-4294 | miRWalk |
| *PECAM1* | hsa-miR-4259 | miRWalk |
| *PECAM1* | hsa-miR-4327 | miRWalk |
| *PECAM1* | hsa-miR-6134 | miRWalk |
| *PECAM1* | hsa-miR-6782-5p | miRWalk |
| *PECAM1* | hsa-miR-6820-5p | miRWalk |
| *PECAM1* | hsa-miR-6822-3p | miRWalk |
| *PECAM1* | hsa-miR-6856-3p | miRWalk |
| *PECAM1* | hsa-miR-7110-3p | miRWalk |
| *PECAM1* | hsa-miR-7150 | miRWalk |
| *PECAM1* | hsa-miR-9898 | miRWalk |
| *PECAM1* | hsa-miR-5682 | miRWalk |
| *PECAM1* | hsa-miR-6743-3p | miRWalk |
| *PECAM1* | hsa-miR-4510 | miRWalk |
| *PECAM1* | hsa-let-7c-5p | miRWalk |
| *PECAM1* | hsa-miR-18a-3p | miRWalk |
| *PECAM1* | hsa-miR-19b-1-5p | miRWalk |
| *PECAM1* | hsa-miR-19b-2-5p | miRWalk |
| *PECAM1* | hsa-miR-22-5p | miRWalk |
| *PECAM1* | hsa-miR-23a-3p | miRWalk |
| *PECAM1* | hsa-miR-24-2-5p | miRWalk |
| *PECAM1* | hsa-miR-25-3p | miRWalk |
| *PECAM1* | hsa-miR-32-3p | miRWalk |
| *PECAM1* | hsa-miR-33a-5p | miRWalk |
| *PECAM1* | hsa-miR-92a-1-5p | miRWalk |
| *PECAM1* | hsa-miR-92a-3p | miRWalk |
| *PECAM1* | hsa-miR-99a-3p | miRWalk |
| *PECAM1* | hsa-miR-29b-1-5p | miRWalk |
| *PECAM1* | hsa-miR-29b-2-5p | miRWalk |
| *PECAM1* | hsa-miR-103a-1-5p | miRWalk |
| *PECAM1* | hsa-miR-105-3p | miRWalk |
| *PECAM1* | hsa-miR-106a-3p | miRWalk |
| *PECAM1* | hsa-miR-208a-5p | miRWalk |
| *PECAM1* | hsa-miR-139-5p | miRWalk |
| *PECAM1* | hsa-miR-7-1-3p | miRWalk |
| *PECAM1* | hsa-miR-181a-5p | miRWalk |
| *PECAM1* | hsa-miR-181a-2-3p | miRWalk |
| *PECAM1* | hsa-miR-181c-3p | miRWalk |
| *PECAM1* | hsa-miR-187-5p | miRWalk |
| *PECAM1* | hsa-miR-187-3p | miRWalk |
| *PECAM1* | hsa-miR-203a-5p | miRWalk |
| *PECAM1* | hsa-miR-204-3p | miRWalk |
| *PECAM1* | hsa-miR-210-3p | miRWalk |
| *PECAM1* | hsa-miR-218-5p | miRWalk |
| *PECAM1* | hsa-miR-219a-1-3p | miRWalk |
| *PECAM1* | hsa-let-7i-3p | miRWalk |
| *PECAM1* | hsa-miR-23b-5p | miRWalk |
| *PECAM1* | hsa-miR-30b-3p | miRWalk |
| *PECAM1* | hsa-miR-124-5p | miRWalk |
| *PECAM1* | hsa-miR-124-3p | miRWalk |
| *PECAM1* | hsa-miR-125b-1-3p | miRWalk |
| *PECAM1* | hsa-miR-128-1-5p | miRWalk |
| *PECAM1* | hsa-miR-130a-5p | miRWalk |
| *PECAM1* | hsa-miR-135a-3p | miRWalk |
| *PECAM1* | hsa-miR-135a-2-3p | miRWalk |
| *PECAM1* | hsa-miR-145-5p | miRWalk |
| *PECAM1* | hsa-miR-145-3p | miRWalk |
| *PECAM1* | hsa-miR-152-3p | miRWalk |
| *PECAM1* | hsa-miR-125b-2-3p | miRWalk |
| *PECAM1* | hsa-miR-134-3p | miRWalk |
| *PECAM1* | hsa-miR-149-5p | miRWalk |
| *PECAM1* | hsa-miR-149-3p | miRWalk |
| *PECAM1* | hsa-miR-150-5p | miRWalk |
| *PECAM1* | hsa-miR-185-5p | miRWalk |
| *PECAM1* | hsa-miR-185-3p | miRWalk |
| *PECAM1* | hsa-miR-188-5p | miRWalk |
| *PECAM1* | hsa-miR-193a-5p | miRWalk |
| *PECAM1* | hsa-miR-206 | miRWalk |
| *PECAM1* | hsa-miR-320a-5p | miRWalk |
| *PECAM1* | hsa-miR-194-3p | miRWalk |
| *PECAM1* | hsa-miR-30c-1-3p | miRWalk |
| *PECAM1* | hsa-miR-34b-5p | miRWalk |
| *PECAM1* | hsa-miR-299-3p | miRWalk |
| *PECAM1* | hsa-miR-99b-3p | miRWalk |
| *PECAM1* | hsa-miR-296-3p | miRWalk |
| *PECAM1* | hsa-miR-361-3p | miRWalk |
| *PECAM1* | hsa-miR-362-3p | miRWalk |
| *PECAM1* | hsa-miR-363-3p | miRWalk |
| *PECAM1* | hsa-miR-365b-5p | miRWalk |
| *PECAM1* | hsa-miR-302d-3p | miRWalk |
| *PECAM1* | hsa-miR-367-3p | miRWalk |
| *PECAM1* | hsa-miR-370-5p | miRWalk |
| *PECAM1* | hsa-miR-371a-5p | miRWalk |
| *PECAM1* | hsa-miR-373-5p | miRWalk |
| *PECAM1* | hsa-miR-378a-5p | miRWalk |
| *PECAM1* | hsa-miR-378a-3p | miRWalk |
| *PECAM1* | hsa-miR-380-5p | miRWalk |
| *PECAM1* | hsa-miR-323a-5p | miRWalk |
| *PECAM1* | hsa-miR-331-5p | miRWalk |
| *PECAM1* | hsa-miR-324-3p | miRWalk |
| *PECAM1* | hsa-miR-338-3p | miRWalk |
| *PECAM1* | hsa-miR-133b | miRWalk |
| *PECAM1* | hsa-miR-345-5p | miRWalk |
| *PECAM1* | hsa-miR-345-3p | miRWalk |
| *PECAM1* | hsa-miR-196b-5p | miRWalk |
| *PECAM1* | hsa-miR-423-3p | miRWalk |
| *PECAM1* | hsa-miR-425-3p | miRWalk |
| *PECAM1* | hsa-miR-18b-5p | miRWalk |
| *PECAM1* | hsa-miR-433-5p | miRWalk |
| *PECAM1* | hsa-miR-433-3p | miRWalk |
| *PECAM1* | hsa-miR-329-5p | miRWalk |
| *PECAM1* | hsa-miR-412-3p | miRWalk |
| *PECAM1* | hsa-miR-410-5p | miRWalk |
| *PECAM1* | hsa-miR-376b-5p | miRWalk |
| *PECAM1* | hsa-miR-483-3p | miRWalk |
| *PECAM1* | hsa-miR-485-5p | miRWalk |
| *PECAM1* | hsa-miR-511-5p | miRWalk |
| *PECAM1* | hsa-miR-202-3p | miRWalk |
| *PECAM1* | hsa-miR-495-5p | miRWalk |
| *PECAM1* | hsa-miR-181d-3p | miRWalk |
| *PECAM1* | hsa-miR-512-5p | miRWalk |
| *PECAM1* | hsa-miR-515-3p | miRWalk |
| *PECAM1* | hsa-miR-519e-5p | miRWalk |
| *PECAM1* | hsa-miR-520f-5p | miRWalk |
| *PECAM1* | hsa-miR-519c-5p | miRWalk |
| *PECAM1* | hsa-miR-520a-5p | miRWalk |
| *PECAM1* | hsa-miR-519b-5p | miRWalk |
| *PECAM1* | hsa-miR-519b-3p | miRWalk |
| *PECAM1* | hsa-miR-523-5p | miRWalk |
| *PECAM1* | hsa-miR-518f-5p | miRWalk |
| *PECAM1* | hsa-miR-518f-3p | miRWalk |
| *PECAM1* | hsa-miR-520b-3p | miRWalk |
| *PECAM1* | hsa-miR-518c-5p | miRWalk |
| *PECAM1* | hsa-miR-517-5p | miRWalk |
| *PECAM1* | hsa-miR-519d-3p | miRWalk |
| *PECAM1* | hsa-miR-520d-5p | miRWalk |
| *PECAM1* | hsa-miR-518e-5p | miRWalk |
| *PECAM1* | hsa-miR-518a-5p | miRWalk |
| *PECAM1* | hsa-miR-522-5p | miRWalk |
| *PECAM1* | hsa-miR-519a-5p | miRWalk |
| *PECAM1* | hsa-miR-527 | miRWalk |
| *PECAM1* | hsa-miR-500a-3p | miRWalk |
| *PECAM1* | hsa-miR-502-5p | miRWalk |
| *PECAM1* | hsa-miR-502-3p | miRWalk |
| *PECAM1* | hsa-miR-503-3p | miRWalk |
| *PECAM1* | hsa-miR-504-3p | miRWalk |
| *PECAM1* | hsa-miR-506-5p | miRWalk |
| *PECAM1* | hsa-miR-506-3p | miRWalk |
| *PECAM1* | hsa-miR-509-3p | miRWalk |
| *PECAM1* | hsa-miR-510-5p | miRWalk |
| *PECAM1* | hsa-miR-532-5p | miRWalk |
| *PECAM1* | hsa-miR-455-5p | miRWalk |
| *PECAM1* | hsa-miR-455-3p | miRWalk |
| *PECAM1* | hsa-miR-487b-5p | miRWalk |
| *PECAM1* | hsa-miR-551a | miRWalk |
| *PECAM1* | hsa-miR-552-5p | miRWalk |
| *PECAM1* | hsa-miR-92b-5p | miRWalk |
| *PECAM1* | hsa-miR-555 | miRWalk |
| *PECAM1* | hsa-miR-564 | miRWalk |
| *PECAM1* | hsa-miR-567 | miRWalk |
| *PECAM1* | hsa-miR-571 | miRWalk |
| *PECAM1* | hsa-miR-578 | miRWalk |
| *PECAM1* | hsa-miR-583 | miRWalk |
| *PECAM1* | hsa-miR-584-5p | miRWalk |
| *PECAM1* | hsa-miR-588 | miRWalk |
| *PECAM1* | hsa-miR-589-5p | miRWalk |
| *PECAM1* | hsa-miR-550a-5p | miRWalk |
| *PECAM1* | hsa-miR-550a-3p | miRWalk |
| *PECAM1* | hsa-miR-593-5p | miRWalk |
| *PECAM1* | hsa-miR-593-3p | miRWalk |
| *PECAM1* | hsa-miR-595 | miRWalk |
| *PECAM1* | hsa-miR-597-3p | miRWalk |
| *PECAM1* | hsa-miR-598-5p | miRWalk |
| *PECAM1* | hsa-miR-601 | miRWalk |
| *PECAM1* | hsa-miR-604 | miRWalk |
| *PECAM1* | hsa-miR-605-3p | miRWalk |
| *PECAM1* | hsa-miR-609 | miRWalk |
| *PECAM1* | hsa-miR-611 | miRWalk |
| *PECAM1* | hsa-miR-613 | miRWalk |
| *PECAM1* | hsa-miR-615-5p | miRWalk |
| *PECAM1* | hsa-miR-617 | miRWalk |
| *PECAM1* | hsa-miR-622 | miRWalk |
| *PECAM1* | hsa-miR-627-3p | miRWalk |
| *PECAM1* | hsa-miR-629-3p | miRWalk |
| *PECAM1* | hsa-miR-632 | miRWalk |
| *PECAM1* | hsa-miR-635 | miRWalk |
| *PECAM1* | hsa-miR-636 | miRWalk |
| *PECAM1* | hsa-miR-637 | miRWalk |
| *PECAM1* | hsa-miR-639 | miRWalk |
| *PECAM1* | hsa-miR-642a-3p | miRWalk |
| *PECAM1* | hsa-miR-645 | miRWalk |
| *PECAM1* | hsa-miR-647 | miRWalk |
| *PECAM1* | hsa-miR-649 | miRWalk |
| *PECAM1* | hsa-miR-650 | miRWalk |
| *PECAM1* | hsa-miR-656-5p | miRWalk |
| *PECAM1* | hsa-miR-657 | miRWalk |
| *PECAM1* | hsa-miR-550a-3-5p | miRWalk |
| *PECAM1* | hsa-miR-767-5p | miRWalk |
| *PECAM1* | hsa-miR-320b | miRWalk |
| *PECAM1* | hsa-miR-1271-5p | miRWalk |
| *PECAM1* | hsa-miR-1301-3p | miRWalk |
| *PECAM1* | hsa-miR-454-5p | miRWalk |
| *PECAM1* | hsa-miR-1185-5p | miRWalk |
| *PECAM1* | hsa-miR-1185-2-3p | miRWalk |
| *PECAM1* | hsa-miR-449c-3p | miRWalk |
| *PECAM1* | hsa-miR-1185-1-3p | miRWalk |
| *PECAM1* | hsa-miR-762 | miRWalk |
| *PECAM1* | hsa-miR-1298-3p | miRWalk |
| *PECAM1* | hsa-miR-2113 | miRWalk |
| *PECAM1* | hsa-miR-761 | miRWalk |
| *PECAM1* | hsa-miR-764 | miRWalk |
| *PECAM1* | hsa-miR-874-3p | miRWalk |
| *PECAM1* | hsa-miR-541-3p | miRWalk |
| *PECAM1* | hsa-miR-876-3p | miRWalk |
| *PECAM1* | hsa-miR-147b-3p | miRWalk |
| *PECAM1* | hsa-miR-744-5p | miRWalk |
| *PECAM1* | hsa-miR-885-3p | miRWalk |
| *PECAM1* | hsa-miR-887-5p | miRWalk |
| *PECAM1* | hsa-miR-665 | miRWalk |
| *PECAM1* | hsa-miR-873-3p | miRWalk |
| *PECAM1* | hsa-miR-374b-3p | miRWalk |
| *PECAM1* | hsa-miR-760 | miRWalk |
| *PECAM1* | hsa-miR-301b-3p | miRWalk |
| *PECAM1* | hsa-miR-208b-5p | miRWalk |
| *PECAM1* | hsa-miR-933 | miRWalk |
| *PECAM1* | hsa-miR-934 | miRWalk |
| *PECAM1* | hsa-miR-936 | miRWalk |
| *PECAM1* | hsa-miR-937-5p | miRWalk |
| *PECAM1* | hsa-miR-939-5p | miRWalk |
| *PECAM1* | hsa-miR-297 | miRWalk |
| *PECAM1* | hsa-miR-1178-5p | miRWalk |
| *PECAM1* | hsa-miR-1178-3p | miRWalk |
| *PECAM1* | hsa-miR-1180-5p | miRWalk |
| *PECAM1* | hsa-miR-1181 | miRWalk |
| *PECAM1* | hsa-miR-1225-5p | miRWalk |
| *PECAM1* | hsa-miR-1226-3p | miRWalk |
| *PECAM1* | hsa-miR-1228-3p | miRWalk |
| *PECAM1* | hsa-miR-1229-3p | miRWalk |
| *PECAM1* | hsa-miR-1231 | miRWalk |
| *PECAM1* | hsa-miR-1233-5p | miRWalk |
| *PECAM1* | hsa-miR-1234-3p | miRWalk |
| *PECAM1* | hsa-miR-1238-5p | miRWalk |
| *PECAM1* | hsa-miR-1200 | miRWalk |
| *PECAM1* | hsa-miR-1202 | miRWalk |
| *PECAM1* | hsa-miR-1204 | miRWalk |
| *PECAM1* | hsa-miR-1285-5p | miRWalk |
| *PECAM1* | hsa-miR-1287-5p | miRWalk |
| *PECAM1* | hsa-miR-1291 | miRWalk |
| *PECAM1* | hsa-miR-1304-5p | miRWalk |
| *PECAM1* | hsa-miR-1304-3p | miRWalk |
| *PECAM1* | hsa-miR-1249-5p | miRWalk |
| *PECAM1* | hsa-miR-1251-3p | miRWalk |
| *PECAM1* | hsa-miR-1253 | miRWalk |
| *PECAM1* | hsa-miR-1261 | miRWalk |
| *PECAM1* | hsa-miR-1266-5p | miRWalk |
| *PECAM1* | hsa-miR-1268a | miRWalk |
| *PECAM1* | hsa-miR-1269a | miRWalk |
| *PECAM1* | hsa-miR-1270 | miRWalk |
| *PECAM1* | hsa-miR-1272 | miRWalk |
| *PECAM1* | hsa-miR-1288-3p | miRWalk |
| *PECAM1* | hsa-miR-664a-5p | miRWalk |
| *PECAM1* | hsa-miR-1306-3p | miRWalk |
| *PECAM1* | hsa-miR-1469 | miRWalk |
| *PECAM1* | hsa-miR-1908-5p | miRWalk |
| *PECAM1* | hsa-miR-1908-3p | miRWalk |
| *PECAM1* | hsa-miR-1909-5p | miRWalk |
| *PECAM1* | hsa-miR-1910-3p | miRWalk |
| *PECAM1* | hsa-miR-1912-5p | miRWalk |
| *PECAM1* | hsa-miR-1915-5p | miRWalk |
| *PECAM1* | hsa-miR-1915-3p | miRWalk |
| *PECAM1* | hsa-miR-2114-5p | miRWalk |
| *PECAM1* | hsa-miR-2116-5p | miRWalk |
| *PECAM1* | hsa-miR-2276-3p | miRWalk |
| *PECAM1* | hsa-miR-2277-5p | miRWalk |
| *PECAM1* | hsa-miR-2277-3p | miRWalk |
| *PECAM1* | hsa-miR-718 | miRWalk |
| *PECAM1* | hsa-miR-3116 | miRWalk |
| *PECAM1* | hsa-miR-3120-5p | miRWalk |
| *PECAM1* | hsa-miR-3120-3p | miRWalk |
| *PECAM1* | hsa-miR-3121-5p | miRWalk |
| *PECAM1* | hsa-miR-3122 | miRWalk |
| *PECAM1* | hsa-miR-548s | miRWalk |
| *PECAM1* | hsa-miR-3126-5p | miRWalk |
| *PECAM1* | hsa-miR-3127-3p | miRWalk |
| *PECAM1* | hsa-miR-3130-3p | miRWalk |
| *PECAM1* | hsa-miR-3131 | miRWalk |
| *PECAM1* | hsa-miR-3135a | miRWalk |
| *PECAM1* | hsa-miR-3137 | miRWalk |
| *PECAM1* | hsa-miR-3140-3p | miRWalk |
| *PECAM1* | hsa-miR-548t-5p | miRWalk |
| *PECAM1* | hsa-miR-3144-3p | miRWalk |
| *PECAM1* | hsa-miR-1273c | miRWalk |
| *PECAM1* | hsa-miR-3148 | miRWalk |
| *PECAM1* | hsa-miR-3150a-5p | miRWalk |
| *PECAM1* | hsa-miR-3150a-3p | miRWalk |
| *PECAM1* | hsa-miR-3151-5p | miRWalk |
| *PECAM1* | hsa-miR-3152-5p | miRWalk |
| *PECAM1* | hsa-miR-3154 | miRWalk |
| *PECAM1* | hsa-miR-3156-3p | miRWalk |
| *PECAM1* | hsa-miR-3157-3p | miRWalk |
| *PECAM1* | hsa-miR-3158-5p | miRWalk |
| *PECAM1* | hsa-miR-3160-5p | miRWalk |
| *PECAM1* | hsa-miR-3160-3p | miRWalk |
| *PECAM1* | hsa-miR-3162-5p | miRWalk |
| *PECAM1* | hsa-miR-3173-5p | miRWalk |
| *PECAM1* | hsa-miR-3173-3p | miRWalk |
| *PECAM1* | hsa-miR-3177-5p | miRWalk |
| *PECAM1* | hsa-miR-3184-5p | miRWalk |
| *PECAM1* | hsa-miR-3184-3p | miRWalk |
| *PECAM1* | hsa-miR-3185 | miRWalk |
| *PECAM1* | hsa-miR-3188 | miRWalk |
| *PECAM1* | hsa-miR-3189-5p | miRWalk |
| *PECAM1* | hsa-miR-3190-5p | miRWalk |
| *PECAM1* | hsa-miR-3191-3p | miRWalk |
| *PECAM1* | hsa-miR-3192-5p | miRWalk |
| *PECAM1* | hsa-miR-3194-5p | miRWalk |
| *PECAM1* | hsa-miR-3194-3p | miRWalk |
| *PECAM1* | hsa-miR-3196 | miRWalk |
| *PECAM1* | hsa-miR-514b-5p | miRWalk |
| *PECAM1* | hsa-miR-3202 | miRWalk |
| *PECAM1* | hsa-miR-4297 | miRWalk |
| *PECAM1* | hsa-miR-4293 | miRWalk |
| *PECAM1* | hsa-miR-4301 | miRWalk |
| *PECAM1* | hsa-miR-4298 | miRWalk |
| *PECAM1* | hsa-miR-4309 | miRWalk |
| *PECAM1* | hsa-miR-4308 | miRWalk |
| *PECAM1* | hsa-miR-4312 | miRWalk |
| *PECAM1* | hsa-miR-4313 | miRWalk |
| *PECAM1* | hsa-miR-4322 | miRWalk |
| *PECAM1* | hsa-miR-4323 | miRWalk |
| *PECAM1* | hsa-miR-4258 | miRWalk |
| *PECAM1* | hsa-miR-4260 | miRWalk |
| *PECAM1* | hsa-miR-4252 | miRWalk |
| *PECAM1* | hsa-miR-4266 | miRWalk |
| *PECAM1* | hsa-miR-4267 | miRWalk |
| *PECAM1* | hsa-miR-2355-5p | miRWalk |
| *PECAM1* | hsa-miR-2355-3p | miRWalk |
| *PECAM1* | hsa-miR-4269 | miRWalk |
| *PECAM1* | hsa-miR-4263 | miRWalk |
| *PECAM1* | hsa-miR-4270 | miRWalk |
| *PECAM1* | hsa-miR-4281 | miRWalk |
| *PECAM1* | hsa-miR-4278 | miRWalk |
| *PECAM1* | hsa-miR-4280 | miRWalk |
| *PECAM1* | hsa-miR-500b-5p | miRWalk |
| *PECAM1* | hsa-miR-3613-5p | miRWalk |
| *PECAM1* | hsa-miR-3614-5p | miRWalk |
| *PECAM1* | hsa-miR-3614-3p | miRWalk |
| *PECAM1* | hsa-miR-3616-3p | miRWalk |
| *PECAM1* | hsa-miR-3619-5p | miRWalk |
| *PECAM1* | hsa-miR-3620-5p | miRWalk |
| *PECAM1* | hsa-miR-3620-3p | miRWalk |
| *PECAM1* | hsa-miR-3621 | miRWalk |
| *PECAM1* | hsa-miR-3622b-5p | miRWalk |
| *PECAM1* | hsa-miR-3649 | miRWalk |
| *PECAM1* | hsa-miR-3654 | miRWalk |
| *PECAM1* | hsa-miR-3655 | miRWalk |
| *PECAM1* | hsa-miR-3663-5p | miRWalk |
| *PECAM1* | hsa-miR-3664-5p | miRWalk |
| *PECAM1* | hsa-miR-3665 | miRWalk |
| *PECAM1* | hsa-miR-3670 | miRWalk |
| *PECAM1* | hsa-miR-3675-5p | miRWalk |
| *PECAM1* | hsa-miR-3675-3p | miRWalk |
| *PECAM1* | hsa-miR-3677-5p | miRWalk |
| *PECAM1* | hsa-miR-3678-3p | miRWalk |
| *PECAM1* | hsa-miR-3679-5p | miRWalk |
| *PECAM1* | hsa-miR-3680-3p | miRWalk |
| *PECAM1* | hsa-miR-3685 | miRWalk |
| *PECAM1* | hsa-miR-3688-5p | miRWalk |
| *PECAM1* | hsa-miR-3690 | miRWalk |
| *PECAM1* | hsa-miR-3713 | miRWalk |
| *PECAM1* | hsa-miR-3180 | miRWalk |
| *PECAM1* | hsa-miR-3907 | miRWalk |
| *PECAM1* | hsa-miR-3689b-3p | miRWalk |
| *PECAM1* | hsa-miR-3913-5p | miRWalk |
| *PECAM1* | hsa-miR-3916 | miRWalk |
| *PECAM1* | hsa-miR-3919 | miRWalk |
| *PECAM1* | hsa-miR-3150b-3p | miRWalk |
| *PECAM1* | hsa-miR-3922-5p | miRWalk |
| *PECAM1* | hsa-miR-3922-3p | miRWalk |
| *PECAM1* | hsa-miR-3925-5p | miRWalk |
| *PECAM1* | hsa-miR-3934-5p | miRWalk |
| *PECAM1* | hsa-miR-3934-3p | miRWalk |
| *PECAM1* | hsa-miR-3936 | miRWalk |
| *PECAM1* | hsa-miR-3937 | miRWalk |
| *PECAM1* | hsa-miR-550b-2-5p | miRWalk |
| *PECAM1* | hsa-miR-550b-3p | miRWalk |
| *PECAM1* | hsa-miR-1268b | miRWalk |
| *PECAM1* | hsa-miR-378e | miRWalk |
| *PECAM1* | hsa-miR-4421 | miRWalk |
| *PECAM1* | hsa-miR-4423-5p | miRWalk |
| *PECAM1* | hsa-miR-378g | miRWalk |
| *PECAM1* | hsa-miR-4428 | miRWalk |
| *PECAM1* | hsa-miR-4429 | miRWalk |
| *PECAM1* | hsa-miR-4433a-3p | miRWalk |
| *PECAM1* | hsa-miR-4439 | miRWalk |
| *PECAM1* | hsa-miR-4440 | miRWalk |
| *PECAM1* | hsa-miR-4442 | miRWalk |
| *PECAM1* | hsa-miR-4443 | miRWalk |
| *PECAM1* | hsa-miR-4445-5p | miRWalk |
| *PECAM1* | hsa-miR-4446-5p | miRWalk |
| *PECAM1* | hsa-miR-4446-3p | miRWalk |
| *PECAM1* | hsa-miR-4448 | miRWalk |
| *PECAM1* | hsa-miR-4451 | miRWalk |
| *PECAM1* | hsa-miR-4452 | miRWalk |
| *PECAM1* | hsa-miR-4453 | miRWalk |
| *PECAM1* | hsa-miR-4458 | miRWalk |
| *PECAM1* | hsa-miR-378h | miRWalk |
| *PECAM1* | hsa-miR-4463 | miRWalk |
| *PECAM1* | hsa-miR-4465 | miRWalk |
| *PECAM1* | hsa-miR-4466 | miRWalk |
| *PECAM1* | hsa-miR-4469 | miRWalk |
| *PECAM1* | hsa-miR-4470 | miRWalk |
| *PECAM1* | hsa-miR-4472 | miRWalk |
| *PECAM1* | hsa-miR-4474-3p | miRWalk |
| *PECAM1* | hsa-miR-4475 | miRWalk |
| *PECAM1* | hsa-miR-3689c | miRWalk |
| *PECAM1* | hsa-miR-3689d | miRWalk |
| *PECAM1* | hsa-miR-4481 | miRWalk |
| *PECAM1* | hsa-miR-4484 | miRWalk |
| *PECAM1* | hsa-miR-4485-5p | miRWalk |
| *PECAM1* | hsa-miR-4487 | miRWalk |
| *PECAM1* | hsa-miR-4488 | miRWalk |
| *PECAM1* | hsa-miR-4489 | miRWalk |
| *PECAM1* | hsa-miR-4491 | miRWalk |
| *PECAM1* | hsa-miR-4492 | miRWalk |
| *PECAM1* | hsa-miR-4494 | miRWalk |
| *PECAM1* | hsa-miR-4498 | miRWalk |
| *PECAM1* | hsa-miR-2392 | miRWalk |
| *PECAM1* | hsa-miR-4507 | miRWalk |
| *PECAM1* | hsa-miR-4513 | miRWalk |
| *PECAM1* | hsa-miR-4514 | miRWalk |
| *PECAM1* | hsa-miR-4520-5p | miRWalk |
| *PECAM1* | hsa-miR-4521 | miRWalk |
| *PECAM1* | hsa-miR-1269b | miRWalk |
| *PECAM1* | hsa-miR-4522 | miRWalk |
| *PECAM1* | hsa-miR-4524a-5p | miRWalk |
| *PECAM1* | hsa-miR-4524a-3p | miRWalk |
| *PECAM1* | hsa-miR-4525 | miRWalk |
| *PECAM1* | hsa-miR-4527 | miRWalk |
| *PECAM1* | hsa-miR-4533 | miRWalk |
| *PECAM1* | hsa-miR-4535 | miRWalk |
| *PECAM1* | hsa-miR-1587 | miRWalk |
| *PECAM1* | hsa-miR-4537 | miRWalk |
| *PECAM1* | hsa-miR-3960 | miRWalk |
| *PECAM1* | hsa-miR-4633-5p | miRWalk |
| *PECAM1* | hsa-miR-4633-3p | miRWalk |
| *PECAM1* | hsa-miR-4640-5p | miRWalk |
| *PECAM1* | hsa-miR-4640-3p | miRWalk |
| *PECAM1* | hsa-miR-4644 | miRWalk |
| *PECAM1* | hsa-miR-4645-3p | miRWalk |
| *PECAM1* | hsa-miR-4646-3p | miRWalk |
| *PECAM1* | hsa-miR-4647 | miRWalk |
| *PECAM1* | hsa-miR-4649-3p | miRWalk |
| *PECAM1* | hsa-miR-4650-5p | miRWalk |
| *PECAM1* | hsa-miR-4651 | miRWalk |
| *PECAM1* | hsa-miR-4652-5p | miRWalk |
| *PECAM1* | hsa-miR-4656 | miRWalk |
| *PECAM1* | hsa-miR-4658 | miRWalk |
| *PECAM1* | hsa-miR-4659a-3p | miRWalk |
| *PECAM1* | hsa-miR-4661-5p | miRWalk |
| *PECAM1* | hsa-miR-4661-3p | miRWalk |
| *PECAM1* | hsa-miR-4662a-5p | miRWalk |
| *PECAM1* | hsa-miR-4664-5p | miRWalk |
| *PECAM1* | hsa-miR-4664-3p | miRWalk |
| *PECAM1* | hsa-miR-4665-5p | miRWalk |
| *PECAM1* | hsa-miR-4667-5p | miRWalk |
| *PECAM1* | hsa-miR-4676-5p | miRWalk |
| *PECAM1* | hsa-miR-4676-3p | miRWalk |
| *PECAM1* | hsa-miR-4677-3p | miRWalk |
| *PECAM1* | hsa-miR-4679 | miRWalk |
| *PECAM1* | hsa-miR-4681 | miRWalk |
| *PECAM1* | hsa-miR-4685-3p | miRWalk |
| *PECAM1* | hsa-miR-4686 | miRWalk |
| *PECAM1* | hsa-miR-4687-3p | miRWalk |
| *PECAM1* | hsa-miR-1343-5p | miRWalk |
| *PECAM1* | hsa-miR-4688 | miRWalk |
| *PECAM1* | hsa-miR-4689 | miRWalk |
| *PECAM1* | hsa-miR-4695-3p | miRWalk |
| *PECAM1* | hsa-miR-4700-5p | miRWalk |
| *PECAM1* | hsa-miR-4700-3p | miRWalk |
| *PECAM1* | hsa-miR-4701-3p | miRWalk |
| *PECAM1* | hsa-miR-4708-5p | miRWalk |
| *PECAM1* | hsa-miR-203b-5p | miRWalk |
| *PECAM1* | hsa-miR-4710 | miRWalk |
| *PECAM1* | hsa-miR-4711-5p | miRWalk |
| *PECAM1* | hsa-miR-4714-5p | miRWalk |
| *PECAM1* | hsa-miR-4715-5p | miRWalk |
| *PECAM1* | hsa-miR-4715-3p | miRWalk |
| *PECAM1* | hsa-miR-4717-3p | miRWalk |
| *PECAM1* | hsa-miR-4722-5p | miRWalk |
| *PECAM1* | hsa-miR-4723-3p | miRWalk |
| *PECAM1* | hsa-miR-4727-5p | miRWalk |
| *PECAM1* | hsa-miR-4727-3p | miRWalk |
| *PECAM1* | hsa-miR-4730 | miRWalk |
| *PECAM1* | hsa-miR-4731-5p | miRWalk |
| *PECAM1* | hsa-miR-4732-5p | miRWalk |
| *PECAM1* | hsa-miR-4732-3p | miRWalk |
| *PECAM1* | hsa-miR-4734 | miRWalk |
| *PECAM1* | hsa-miR-4736 | miRWalk |
| *PECAM1* | hsa-miR-3064-5p | miRWalk |
| *PECAM1* | hsa-miR-4738-3p | miRWalk |
| *PECAM1* | hsa-miR-4739 | miRWalk |
| *PECAM1* | hsa-miR-4740-5p | miRWalk |
| *PECAM1* | hsa-miR-4740-3p | miRWalk |
| *PECAM1* | hsa-miR-4742-3p | miRWalk |
| *PECAM1* | hsa-miR-4745-3p | miRWalk |
| *PECAM1* | hsa-miR-4746-5p | miRWalk |
| *PECAM1* | hsa-miR-4747-5p | miRWalk |
| *PECAM1* | hsa-miR-4748 | miRWalk |
| *PECAM1* | hsa-miR-4749-5p | miRWalk |
| *PECAM1* | hsa-miR-4749-3p | miRWalk |
| *PECAM1* | hsa-miR-4750-5p | miRWalk |
| *PECAM1* | hsa-miR-4754 | miRWalk |
| *PECAM1* | hsa-miR-4755-3p | miRWalk |
| *PECAM1* | hsa-miR-4758-3p | miRWalk |
| *PECAM1* | hsa-miR-4761-5p | miRWalk |
| *PECAM1* | hsa-miR-4761-3p | miRWalk |
| *PECAM1* | hsa-miR-4766-5p | miRWalk |
| *PECAM1* | hsa-miR-4767 | miRWalk |
| *PECAM1* | hsa-miR-4768-5p | miRWalk |
| *PECAM1* | hsa-miR-4769-5p | miRWalk |
| *PECAM1* | hsa-miR-4772-3p | miRWalk |
| *PECAM1* | hsa-miR-4776-3p | miRWalk |
| *PECAM1* | hsa-miR-4778-3p | miRWalk |
| *PECAM1* | hsa-miR-4779 | miRWalk |
| *PECAM1* | hsa-miR-4780 | miRWalk |
| *PECAM1* | hsa-miR-4436b-5p | miRWalk |
| *PECAM1* | hsa-miR-4436b-3p | miRWalk |
| *PECAM1* | hsa-miR-4783-5p | miRWalk |
| *PECAM1* | hsa-miR-4783-3p | miRWalk |
| *PECAM1* | hsa-miR-4784 | miRWalk |
| *PECAM1* | hsa-miR-4785 | miRWalk |
| *PECAM1* | hsa-miR-1245b-5p | miRWalk |
| *PECAM1* | hsa-miR-4787-5p | miRWalk |
| *PECAM1* | hsa-miR-4788 | miRWalk |
| *PECAM1* | hsa-miR-4795-5p | miRWalk |
| *PECAM1* | hsa-miR-4796-5p | miRWalk |
| *PECAM1* | hsa-miR-4802-5p | miRWalk |
| *PECAM1* | hsa-miR-5002-5p | miRWalk |
| *PECAM1* | hsa-miR-5002-3p | miRWalk |
| *PECAM1* | hsa-miR-5004-5p | miRWalk |
| *PECAM1* | hsa-miR-5004-3p | miRWalk |
| *PECAM1* | hsa-miR-5006-5p | miRWalk |
| *PECAM1* | hsa-miR-5007-5p | miRWalk |
| *PECAM1* | hsa-miR-5008-5p | miRWalk |
| *PECAM1* | hsa-miR-5008-3p | miRWalk |
| *PECAM1* | hsa-miR-5087 | miRWalk |
| *PECAM1* | hsa-miR-5088-5p | miRWalk |
| *PECAM1* | hsa-miR-5088-3p | miRWalk |
| *PECAM1* | hsa-miR-5089-5p | miRWalk |
| *PECAM1* | hsa-miR-5089-3p | miRWalk |
| *PECAM1* | hsa-miR-5090 | miRWalk |
| *PECAM1* | hsa-miR-5093 | miRWalk |
| *PECAM1* | hsa-miR-5187-5p | miRWalk |
| *PECAM1* | hsa-miR-5189-3p | miRWalk |
| *PECAM1* | hsa-miR-5193 | miRWalk |
| *PECAM1* | hsa-miR-5194 | miRWalk |
| *PECAM1* | hsa-miR-5195-5p | miRWalk |
| *PECAM1* | hsa-miR-5196-3p | miRWalk |
| *PECAM1* | hsa-miR-4524b-3p | miRWalk |
| *PECAM1* | hsa-miR-5571-3p | miRWalk |
| *PECAM1* | hsa-miR-664b-5p | miRWalk |
| *PECAM1* | hsa-miR-664b-3p | miRWalk |
| *PECAM1* | hsa-miR-5584-3p | miRWalk |
| *PECAM1* | hsa-miR-5586-3p | miRWalk |
| *PECAM1* | hsa-miR-548au-3p | miRWalk |
| *PECAM1* | hsa-miR-1295b-5p | miRWalk |
| *PECAM1* | hsa-miR-5588-3p | miRWalk |
| *PECAM1* | hsa-miR-5589-5p | miRWalk |
| *PECAM1* | hsa-miR-5589-3p | miRWalk |
| *PECAM1* | hsa-miR-5591-3p | miRWalk |
| *PECAM1* | hsa-miR-5684 | miRWalk |
| *PECAM1* | hsa-miR-5681b | miRWalk |
| *PECAM1* | hsa-miR-5689 | miRWalk |
| *PECAM1* | hsa-miR-5705 | miRWalk |
| *PECAM1* | hsa-miR-5708 | miRWalk |
| *PECAM1* | hsa-miR-5787 | miRWalk |
| *PECAM1* | hsa-miR-1199-5p | miRWalk |
| *PECAM1* | hsa-miR-6071 | miRWalk |
| *PECAM1* | hsa-miR-6074 | miRWalk |
| *PECAM1* | hsa-miR-6075 | miRWalk |
| *PECAM1* | hsa-miR-6076 | miRWalk |
| *PECAM1* | hsa-miR-6077 | miRWalk |
| *PECAM1* | hsa-miR-6078 | miRWalk |
| *PECAM1* | hsa-miR-6079 | miRWalk |
| *PECAM1* | hsa-miR-6080 | miRWalk |
| *PECAM1* | hsa-miR-6084 | miRWalk |
| *PECAM1* | hsa-miR-6085 | miRWalk |
| *PECAM1* | hsa-miR-6086 | miRWalk |
| *PECAM1* | hsa-miR-6088 | miRWalk |
| *PECAM1* | hsa-miR-6090 | miRWalk |
| *PECAM1* | hsa-miR-6124 | miRWalk |
| *PECAM1* | hsa-miR-6126 | miRWalk |
| *PECAM1* | hsa-miR-6129 | miRWalk |
| *PECAM1* | hsa-miR-6132 | miRWalk |
| *PECAM1* | hsa-miR-6133 | miRWalk |
| *PECAM1* | hsa-miR-6165 | miRWalk |
| *PECAM1* | hsa-miR-6500-5p | miRWalk |
| *PECAM1* | hsa-miR-6501-3p | miRWalk |
| *PECAM1* | hsa-miR-6503-3p | miRWalk |
| *PECAM1* | hsa-miR-6505-5p | miRWalk |
| *PECAM1* | hsa-miR-6507-3p | miRWalk |
| *PECAM1* | hsa-miR-6509-3p | miRWalk |
| *PECAM1* | hsa-miR-6512-5p | miRWalk |
| *PECAM1* | hsa-miR-6513-5p | miRWalk |
| *PECAM1* | hsa-miR-6514-3p | miRWalk |
| *PECAM1* | hsa-miR-6515-5p | miRWalk |
| *PECAM1* | hsa-miR-6715b-5p | miRWalk |
| *PECAM1* | hsa-miR-6715b-3p | miRWalk |
| *PECAM1* | hsa-miR-6720-5p | miRWalk |
| *PECAM1* | hsa-miR-6722-5p | miRWalk |
| *PECAM1* | hsa-miR-6726-5p | miRWalk |
| *PECAM1* | hsa-miR-6727-5p | miRWalk |
| *PECAM1* | hsa-miR-6729-5p | miRWalk |
| *PECAM1* | hsa-miR-6729-3p | miRWalk |
| *PECAM1* | hsa-miR-6730-5p | miRWalk |
| *PECAM1* | hsa-miR-6730-3p | miRWalk |
| *PECAM1* | hsa-miR-6731-5p | miRWalk |
| *PECAM1* | hsa-miR-6731-3p | miRWalk |
| *PECAM1* | hsa-miR-6732-5p | miRWalk |
| *PECAM1* | hsa-miR-6733-3p | miRWalk |
| *PECAM1* | hsa-miR-6736-5p | miRWalk |
| *PECAM1* | hsa-miR-6736-3p | miRWalk |
| *PECAM1* | hsa-miR-6737-5p | miRWalk |
| *PECAM1* | hsa-miR-6740-5p | miRWalk |
| *PECAM1* | hsa-miR-6740-3p | miRWalk |
| *PECAM1* | hsa-miR-6741-5p | miRWalk |
| *PECAM1* | hsa-miR-6741-3p | miRWalk |
| *PECAM1* | hsa-miR-6742-3p | miRWalk |
| *PECAM1* | hsa-miR-6746-5p | miRWalk |
| *PECAM1* | hsa-miR-6746-3p | miRWalk |
| *PECAM1* | hsa-miR-6747-5p | miRWalk |
| *PECAM1* | hsa-miR-6748-5p | miRWalk |
| *PECAM1* | hsa-miR-6748-3p | miRWalk |
| *PECAM1* | hsa-miR-6749-5p | miRWalk |
| *PECAM1* | hsa-miR-6751-5p | miRWalk |
| *PECAM1* | hsa-miR-6751-3p | miRWalk |
| *PECAM1* | hsa-miR-6752-5p | miRWalk |
| *PECAM1* | hsa-miR-6754-5p | miRWalk |
| *PECAM1* | hsa-miR-6754-3p | miRWalk |
| *PECAM1* | hsa-miR-6755-5p | miRWalk |
| *PECAM1* | hsa-miR-6757-5p | miRWalk |
| *PECAM1* | hsa-miR-6758-5p | miRWalk |
| *PECAM1* | hsa-miR-6758-3p | miRWalk |
| *PECAM1* | hsa-miR-6759-5p | miRWalk |
| *PECAM1* | hsa-miR-6760-5p | miRWalk |
| *PECAM1* | hsa-miR-6761-5p | miRWalk |
| *PECAM1* | hsa-miR-6761-3p | miRWalk |
| *PECAM1* | hsa-miR-6764-5p | miRWalk |
| *PECAM1* | hsa-miR-6764-3p | miRWalk |
| *PECAM1* | hsa-miR-6765-5p | miRWalk |
| *PECAM1* | hsa-miR-6766-5p | miRWalk |
| *PECAM1* | hsa-miR-6766-3p | miRWalk |
| *PECAM1* | hsa-miR-6767-5p | miRWalk |
| *PECAM1* | hsa-miR-6767-3p | miRWalk |
| *PECAM1* | hsa-miR-6769a-5p | miRWalk |
| *PECAM1* | hsa-miR-6769a-3p | miRWalk |
| *PECAM1* | hsa-miR-6772-5p | miRWalk |
| *PECAM1* | hsa-miR-6772-3p | miRWalk |
| *PECAM1* | hsa-miR-6773-5p | miRWalk |
| *PECAM1* | hsa-miR-6776-5p | miRWalk |
| *PECAM1* | hsa-miR-6778-5p | miRWalk |
| *PECAM1* | hsa-miR-6779-5p | miRWalk |
| *PECAM1* | hsa-miR-6779-3p | miRWalk |
| *PECAM1* | hsa-miR-6780a-5p | miRWalk |
| *PECAM1* | hsa-miR-6781-5p | miRWalk |
| *PECAM1* | hsa-miR-6781-3p | miRWalk |
| *PECAM1* | hsa-miR-6783-5p | miRWalk |
| *PECAM1* | hsa-miR-6783-3p | miRWalk |
| *PECAM1* | hsa-miR-6784-3p | miRWalk |
| *PECAM1* | hsa-miR-6785-3p | miRWalk |
| *PECAM1* | hsa-miR-6786-3p | miRWalk |
| *PECAM1* | hsa-miR-6787-5p | miRWalk |
| *PECAM1* | hsa-miR-6788-5p | miRWalk |
| *PECAM1* | hsa-miR-6789-5p | miRWalk |
| *PECAM1* | hsa-miR-6789-3p | miRWalk |
| *PECAM1* | hsa-miR-6790-3p | miRWalk |
| *PECAM1* | hsa-miR-6791-3p | miRWalk |
| *PECAM1* | hsa-miR-6792-3p | miRWalk |
| *PECAM1* | hsa-miR-6793-3p | miRWalk |
| *PECAM1* | hsa-miR-6795-3p | miRWalk |
| *PECAM1* | hsa-miR-6800-3p | miRWalk |
| *PECAM1* | hsa-miR-6802-5p | miRWalk |
| *PECAM1* | hsa-miR-6803-3p | miRWalk |
| *PECAM1* | hsa-miR-6804-5p | miRWalk |
| *PECAM1* | hsa-miR-6804-3p | miRWalk |
| *PECAM1* | hsa-miR-6805-5p | miRWalk |
| *PECAM1* | hsa-miR-6805-3p | miRWalk |
| *PECAM1* | hsa-miR-6806-3p | miRWalk |
| *PECAM1* | hsa-miR-6807-3p | miRWalk |
| *PECAM1* | hsa-miR-6808-5p | miRWalk |
| *PECAM1* | hsa-miR-6809-5p | miRWalk |
| *PECAM1* | hsa-miR-6809-3p | miRWalk |
| *PECAM1* | hsa-miR-6810-5p | miRWalk |
| *PECAM1* | hsa-miR-6810-3p | miRWalk |
| *PECAM1* | hsa-miR-6811-3p | miRWalk |
| *PECAM1* | hsa-miR-6812-5p | miRWalk |
| *PECAM1* | hsa-miR-6812-3p | miRWalk |
| *PECAM1* | hsa-miR-6813-5p | miRWalk |
| *PECAM1* | hsa-miR-6815-3p | miRWalk |
| *PECAM1* | hsa-miR-6816-5p | miRWalk |
| *PECAM1* | hsa-miR-6817-5p | miRWalk |
| *PECAM1* | hsa-miR-6819-3p | miRWalk |
| *PECAM1* | hsa-miR-6822-5p | miRWalk |
| *PECAM1* | hsa-miR-6823-3p | miRWalk |
| *PECAM1* | hsa-miR-6824-5p | miRWalk |
| *PECAM1* | hsa-miR-6824-3p | miRWalk |
| *PECAM1* | hsa-miR-6827-5p | miRWalk |
| *PECAM1* | hsa-miR-6829-5p | miRWalk |
| *PECAM1* | hsa-miR-6830-5p | miRWalk |
| *PECAM1* | hsa-miR-6831-5p | miRWalk |
| *PECAM1* | hsa-miR-6831-3p | miRWalk |
| *PECAM1* | hsa-miR-6832-5p | miRWalk |
| *PECAM1* | hsa-miR-6834-5p | miRWalk |
| *PECAM1* | hsa-miR-6835-3p | miRWalk |
| *PECAM1* | hsa-miR-6780b-3p | miRWalk |
| *PECAM1* | hsa-miR-6836-5p | miRWalk |
| *PECAM1* | hsa-miR-6837-5p | miRWalk |
| *PECAM1* | hsa-miR-6837-3p | miRWalk |
| *PECAM1* | hsa-miR-6838-5p | miRWalk |
| *PECAM1* | hsa-miR-6838-3p | miRWalk |
| *PECAM1* | hsa-miR-6840-3p | miRWalk |
| *PECAM1* | hsa-miR-6841-5p | miRWalk |
| *PECAM1* | hsa-miR-6842-5p | miRWalk |
| *PECAM1* | hsa-miR-6842-3p | miRWalk |
| *PECAM1* | hsa-miR-6845-3p | miRWalk |
| *PECAM1* | hsa-miR-6847-5p | miRWalk |
| *PECAM1* | hsa-miR-6847-3p | miRWalk |
| *PECAM1* | hsa-miR-6849-3p | miRWalk |
| *PECAM1* | hsa-miR-6850-3p | miRWalk |
| *PECAM1* | hsa-miR-6853-3p | miRWalk |
| *PECAM1* | hsa-miR-6854-3p | miRWalk |
| *PECAM1* | hsa-miR-6855-5p | miRWalk |
| *PECAM1* | hsa-miR-6857-5p | miRWalk |
| *PECAM1* | hsa-miR-6857-3p | miRWalk |
| *PECAM1* | hsa-miR-6858-5p | miRWalk |
| *PECAM1* | hsa-miR-6858-3p | miRWalk |
| *PECAM1* | hsa-miR-6859-5p | miRWalk |
| *PECAM1* | hsa-miR-6859-3p | miRWalk |
| *PECAM1* | hsa-miR-6769b-3p | miRWalk |
| *PECAM1* | hsa-miR-6860 | miRWalk |
| *PECAM1* | hsa-miR-6861-5p | miRWalk |
| *PECAM1* | hsa-miR-6861-3p | miRWalk |
| *PECAM1* | hsa-miR-6862-5p | miRWalk |
| *PECAM1* | hsa-miR-6865-3p | miRWalk |
| *PECAM1* | hsa-miR-6867-5p | miRWalk |
| *PECAM1* | hsa-miR-6869-5p | miRWalk |
| *PECAM1* | hsa-miR-6869-3p | miRWalk |
| *PECAM1* | hsa-miR-6870-5p | miRWalk |
| *PECAM1* | hsa-miR-6871-3p | miRWalk |
| *PECAM1* | hsa-miR-6875-5p | miRWalk |
| *PECAM1* | hsa-miR-6877-5p | miRWalk |
| *PECAM1* | hsa-miR-6877-3p | miRWalk |
| *PECAM1* | hsa-miR-6878-3p | miRWalk |
| *PECAM1* | hsa-miR-6879-5p | miRWalk |
| *PECAM1* | hsa-miR-6880-3p | miRWalk |
| *PECAM1* | hsa-miR-6882-3p | miRWalk |
| *PECAM1* | hsa-miR-6883-5p | miRWalk |
| *PECAM1* | hsa-miR-6883-3p | miRWalk |
| *PECAM1* | hsa-miR-6884-5p | miRWalk |
| *PECAM1* | hsa-miR-6885-3p | miRWalk |
| *PECAM1* | hsa-miR-6886-5p | miRWalk |
| *PECAM1* | hsa-miR-6887-5p | miRWalk |
| *PECAM1* | hsa-miR-6887-3p | miRWalk |
| *PECAM1* | hsa-miR-6889-5p | miRWalk |
| *PECAM1* | hsa-miR-6890-5p | miRWalk |
| *PECAM1* | hsa-miR-6891-5p | miRWalk |
| *PECAM1* | hsa-miR-6892-5p | miRWalk |
| *PECAM1* | hsa-miR-6893-3p | miRWalk |
| *PECAM1* | hsa-miR-6895-5p | miRWalk |
| *PECAM1* | hsa-miR-7106-5p | miRWalk |
| *PECAM1* | hsa-miR-7106-3p | miRWalk |
| *PECAM1* | hsa-miR-7109-3p | miRWalk |
| *PECAM1* | hsa-miR-7110-5p | miRWalk |
| *PECAM1* | hsa-miR-7111-3p | miRWalk |
| *PECAM1* | hsa-miR-7112-5p | miRWalk |
| *PECAM1* | hsa-miR-7114-5p | miRWalk |
| *PECAM1* | hsa-miR-7151-5p | miRWalk |
| *PECAM1* | hsa-miR-7151-3p | miRWalk |
| *PECAM1* | hsa-miR-7152-3p | miRWalk |
| *PECAM1* | hsa-miR-7153-3p | miRWalk |
| *PECAM1* | hsa-miR-7154-3p | miRWalk |
| *PECAM1* | hsa-miR-7155-5p | miRWalk |
| *PECAM1* | hsa-miR-7156-3p | miRWalk |
| *PECAM1* | hsa-miR-7157-3p | miRWalk |
| *PECAM1* | hsa-miR-7158-5p | miRWalk |
| *PECAM1* | hsa-miR-7159-5p | miRWalk |
| *PECAM1* | hsa-miR-7843-3p | miRWalk |
| *PECAM1* | hsa-miR-4433b-3p | miRWalk |
| *PECAM1* | hsa-miR-1273h-5p | miRWalk |
| *PECAM1* | hsa-miR-7845-5p | miRWalk |
| *PECAM1* | hsa-miR-7847-3p | miRWalk |
| *PECAM1* | hsa-miR-7848-3p | miRWalk |
| *PECAM1* | hsa-miR-7851-3p | miRWalk |
| *PECAM1* | hsa-miR-7854-3p | miRWalk |
| *PECAM1* | hsa-miR-7855-5p | miRWalk |
| *PECAM1* | hsa-miR-7856-5p | miRWalk |
| *PECAM1* | hsa-miR-8055 | miRWalk |
| *PECAM1* | hsa-miR-8057 | miRWalk |
| *PECAM1* | hsa-miR-8064 | miRWalk |
| *PECAM1* | hsa-miR-8068 | miRWalk |
| *PECAM1* | hsa-miR-8073 | miRWalk |
| *PECAM1* | hsa-miR-8074 | miRWalk |
| *PECAM1* | hsa-miR-8079 | miRWalk |
| *PECAM1* | hsa-miR-8083 | miRWalk |
| *PECAM1* | hsa-miR-8085 | miRWalk |
| *PECAM1* | hsa-miR-8086 | miRWalk |
| *PECAM1* | hsa-miR-8089 | miRWalk |
| *PECAM1* | hsa-miR-9899 | miRWalk |
| *PECAM1* | hsa-miR-1843 | miRWalk |
| *PECAM1* | hsa-miR-9986 | miRWalk |
| *PECAM1* | hsa-miR-10392-5p | miRWalk |
| *PECAM1* | hsa-miR-10398-5p | miRWalk |
| *PECAM1* | hsa-miR-10398-3p | miRWalk |
| *PECAM1* | hsa-miR-10400-3p | miRWalk |
| *PECAM1* | hsa-miR-10401-5p | miRWalk |
| *PECAM1* | hsa-miR-10526-3p | miRWalk |
| *PECAM1* | hsa-miR-11400 | miRWalk |
| *PECAM1* | hsa-miR-11401 | miRWalk |
| *PECAM1* | hsa-miR-3085-5p | miRWalk |
| *PECAM1* | hsa-miR-6529-5p | miRWalk |
| *PECAM1* | hsa-miR-12117 | miRWalk |
| *PECAM1* | hsa-miR-12118 | miRWalk |
| *PECAM1* | hsa-miR-12119 | miRWalk |
| *PECAM1* | hsa-miR-12120 | miRWalk |
| *PECAM1* | hsa-miR-12124 | miRWalk |
| *PECAM1* | hsa-miR-12126 | miRWalk |
| *PECAM1* | hsa-miR-12127 | miRWalk |
| *PECAM1* | hsa-miR-12128 | miRWalk |
| *PECAM1* | hsa-miR-12133 | miRWalk |
